# The long noncoding RNA landscape of neuroendocrine prostate cancer and its clinical implications

**DOI:** 10.1093/gigascience/giy050

**Published:** 2018-05-10

**Authors:** Varune Rohan Ramnarine, Mohammed Alshalalfa, Fan Mo, Noushin Nabavi, Nicholas Erho, Mandeep Takhar, Robert Shukin, Sonal Brahmbhatt, Alexander Gawronski, Maxim Kobelev, Mannan Nouri, Dong Lin, Harrison Tsai, Tamara L Lotan, R Jefferey Karnes, Mark A Rubin, Amina Zoubeidi, Martin E Gleave, Cenk Sahinalp, Alexander W Wyatt, Stanislav V Volik, Himisha Beltran, Elai Davicioni, Yuzhuo Wang, Colin C Collins

**Affiliations:** 1 Vancouver Prostate Centre & Department of Urologic Sciences, University of British Columbia, Vancouver, BC, Canada; 2 GenomeDx Biosciences Inc., Vancouver, BC, Canada; 3 Department of Computer Science, Simon Fraser University, Burnaby, BC, Canada; 4 Department of Experimental Therapeutics, BC Cancer Agency, Vancouver, BC, Canada; 5 Department of Pathology, Johns Hopkins School of Medicine, Baltimore, MD, USA; 6 Department of Urology, Mayo Clinic College of Medicine, Rochester, MN, USA; 7 Department of Pathology and Laboratory Medicine, Weill Cornell Cancer Center, Weill Cornell Medical College, New York, NY, USA; 8 Department of Computer Science, Indiana University, Bloomington, IN, USA; 9 Department of Medicine, Weill Cornell Cancer Center, Weill Cornell Medical College, New York, NY, USA

**Keywords:** neuroendocrine prostate cancer, transdifferentiation, small cell carcinoma, long non-coding RNA

## Abstract

**Background:**

Treatment-induced neuroendocrine prostate cancer (tNEPC) is an aggressive variant of late-stage metastatic castrate-resistant prostate cancer that commonly arises through neuroendocrine transdifferentiation (NEtD). Treatment options are limited, ineffective, and, for most patients, result in death in less than a year. We previously developed a first-in-field patient-derived xenograft (PDX) model of NEtD. Longitudinal deep transcriptome profiling of this model enabled monitoring of dynamic transcriptional changes during NEtD and in the context of androgen deprivation. Long non-coding RNA (lncRNA) are implicated in cancer where they can control gene regulation. Until now, the expression of lncRNAs during NEtD and their clinical associations were unexplored.

**Results:**

We implemented a next-generation sequence analysis pipeline that can detect transcripts at low expression levels and built a genome-wide catalogue (n = 37,749) of lncRNAs. We applied this pipeline to 927 clinical samples and our high-fidelity NEtD model LTL331 and identified 821 lncRNAs in NEPC. Among these are 122 lncRNAs that robustly distinguish NEPC from prostate adenocarcinoma (AD) patient tumours. The highest expressed lncRNAs within this signature are H19, LINC00617, and SSTR5-AS1. Another 742 are associated with the NEtD process and fall into four distinct patterns of expression (NEtD lncRNA Class I, II, III, and IV) in our PDX model and clinical samples. Each class has significant (z-scores >2) and unique enrichment for transcription factor binding site (TFBS) motifs in their sequences. Enriched TFBS include (1) TP53 and BRN1 in Class I, (2) ELF5, SPIC, and HOXD1 in Class II, (3) SPDEF in Class III, (4) HSF1 and FOXA1 in Class IV, and (5) TWIST1 when merging Class III with IV. Common TFBS in all NEtD lncRNA were also identified and include E2F, REST, PAX5, PAX9, and STAF. Interrogation of the top deregulated candidates (n = 100) in radical prostatectomy adenocarcinoma samples with long-term follow-up (median 18 years) revealed significant clinicopathological associations. Specifically, we identified 25 that are associated with rapid metastasis following androgen deprivation therapy (ADT). Two of these lncRNAs (SSTR5-AS1 and LINC00514) stratified patients undergoing ADT based on patient outcome.

**Discussion:**

To date, a comprehensive characterization of the dynamic landscape of lncRNAs during the NEtD process has not been performed. A temporal analysis of the PDX-based NEtD model has for the first time provided this dynamic landscape. TFBS analysis identified NEPC-related TF motifs present within the NEtD lncRNA sequences, suggesting functional roles for these lncRNAs in NEPC pathogenesis. Furthermore, select NEtD lncRNAs appear to be associated with metastasis and patients receiving ADT. Treatment-related metastasis is a clinical consequence of NEPC tumours. Top candidate lncRNAs FENDRR, H19, LINC00514, LINC00617, and SSTR5-AS1 identified in this study are implicated in the development of NEPC. We present here for the first time a genome-wide catalogue of NEtD lncRNAs that characterize the transdifferentiation process and a robust NEPC lncRNA patient expression signature. To accomplish this, we carried out the largest integrative study that applied a PDX NEtD model to clinical samples. These NEtD and NEPC lncRNAs are strong candidates for clinical biomarkers and therapeutic targets and warrant further investigation.

## Introduction

Prostate cancer (PCa) is the most common cancer affecting men and is the third highest cause of cancer death in developed countries globally [1]. Advances in detection and treatment for PCa have translated to many men being successfully treated by surgery and/or radiation. Concomitantly, androgen deprivation therapy (ADT) has resulted in significant survival gains for men with metastatic PCa. Commonly administered therapeutics include Enzalutamide, Bicalutamide, and Abiraterone [2]. These drugs inhibit the androgen signaling axis, a growth and differentiation-inducing pathway mediated by the androgen receptor (AR). Despite these successes, with the steady accumulation of facilitating genomic and epigenomic aberrations, a more aggressive tumour capable of growing in castrate levels of testosterone can develop [3], termed castration-resistant prostate cancer (CRPC). Three main classes of treatment resistance to AR-targeted therapies exist, falling into two broad categories associated with AR signaling [4]. The majority of CRPC reactivate the AR signaling axis (AR^+^ CRPC). However, some tumour cells leverage their inherent plasticity and progress to an AR-negative state (AR^−^ CRPC), circumventing AR dependence. AR^−^ CRPC is highly heterogeneous, but a major established aggressive subtype is neuroendocrine prostate cancer (NEPC) [5]. NEPC is pathologically and clinically similar to small cell carcinoma of the prostate (SCPC), which has been defined as a distinct morphological subtype of PCa with neuroendocrine differentiation [6]. Xenograft NEPC models have shown expression of a dominant and irreversible neuronal-like phenotype [7] where conventional CRPC therapies are ineffective. Platinum-based chemotherapy is only transiently effective, resulting in poor overall survival [8] with most patients surviving ∼7 months [9]. Molecular pathology markers include expression of chromogranin A (CHGA), synaptophysin (SYP), neuron-specific enolase (NSE) [10], cell-surface marker CEACAM5 [11], and negative (or low) levels of AR and AR-regulated genes such as PSA [7]. NEPC can arise *de novo* but much more commonly occurs as a consequence of ADT via an adaptive process termed neuroendocrine transdifferentiation (NEtD) [7, 12] and frequently metastasizes to visceral organs [13]. Predisposing aberrations for NEtD include loss of RB1 [14], TP53 [15], mutation of Trp53 [16], and/or PTEN inactivation [17, 18]. Emerging data suggest drivers include splice factor SRRM4 [19–21], master neural transcription factor BRN2 [22], and forkhead box A1 (FOXA1) [23]. NEPC tumours have been characterized with (1) gains in MYCN and AURKA [5]; (2) overexpression of PEG10 [24], HP1α [25], N-Myc [26, 27], SOX2 [28], and SOX11 [18]; (3) down-regulation of PHF8, KDM3A [29, 30], REST [31], and SPEDF [32]; and lastly (4) disease dependency on GPX4 [33]. Discoveries such as these continue to define the protein-coding transcriptome of NEPC. The process of transdifferentiation, however, is highly complex and likely involves multiple layers of genetic and epigenetic regulation.

Dysregulation of long non-coding RNAs (lncRNAs) could provide an additional mechanism for the gene expression alterations that occur during NEtD. LncRNAs are broadly defined as large (>200 nucleotides/nt) RNA transcripts, with the most abundant subtypes classified as antisense RNAs, pseudogenes, and long intergenic noncoding RNAs (lincRNA) [34]. They are implicated in a variety of diseases, and their association with cancer progression is reported through mechanisms such as remodeling of chromatin, transcriptional co-activation or repression, modulation of protein activity, post-transcriptional regulation, or as decoy elements [35–37]. LncRNAs form an important regulatory layer in global gene expression and, as such, alterations of lncRNAs in cancer are identified as one of the driving forces for tumorigenesis [38, 39], cancer progression, and metastasis [40, 41]. More specifically in PCa, lncRNAs have been reported to play critical roles at every stage, including the transformation of normal prostate cells to prostate intraepithelial neoplastic cells, the development of localized tumours, and finally progression to advanced metastatic disease [42]. These roles in initiation and progression are due to aberrant lncRNA expression, which changes the balance of protein-coding genes involved in processes such as proliferation and apoptosis, thereby facilitating cellular transformation.

We recently developed a first-in-field transplantable patient-derived xenograft (PDX) model of NEtD: a treatment-naïve adenocarcinoma (LTL331) that upon host castration initially regresses (LTL331–8 and 12 week) but then rapidly relapses as terminally differentiated NEPC (LTL331R) [7]. In our previous study using this model, we demonstrated a lack of evidence for NEPC cells before host castration and the conservation of genome characteristics pre- and post-castration, strongly suggesting a phase transition or state change from adenocarcinoma to NEPC [24]. With this model we have identified protein-coding transcripts such as PEG10 [24], SRRM4 [19], and HP1α [25] that are active in the phase transition and validated the discovery of BRN2 [22]. In addition to these, our model has led to the identification of candidate biomarkers and therapeutic targets for NEPC, including the DEK proto-oncogene [43] and epigenetic regulators CBX2 and EZH2 [44] (members of the polycomb group family of transcriptional repressors). We now report the comprehensive characterization of lncRNAs in our NEtD model. In the current study, we used the longitudinal genomic profiling of our PDX-based NEtD model focusing on lncRNA transcripts. We hypothesized that lncRNA expression across the “time series” would associate with the development of NEPC. Our objective was to comprehensively characterize the dynamic lncRNA landscape of NEtD and NEPC, identify putative functional motifs within lncRNA sequences, determine the clinical relevance of lncRNA expression, and identify associated clinicopathological features. To accomplish this, we implemented a sequence analysis pipeline optimized for the detection of lncRNAs, identified a clinical signature that can robustly distinguish NEPC from adenocarcinoma (AD) tumours, and identified four NEtD-associated lncRNA expression profiles. We also identified significant enrichment of well-known transcription factor motifs within the lncRNA sequences. Lastly, we observed that a subset of these lncRNAs is associated with rapid metastasis in treated patients and can stratify tumours based on patient outcome. We present here for the first time a comprehensive landscape of NEPC lncRNAs and their clinical associations.

## Results

### Comprehensive catalogue of lncRNAs in NEPC

**Figure 1:**
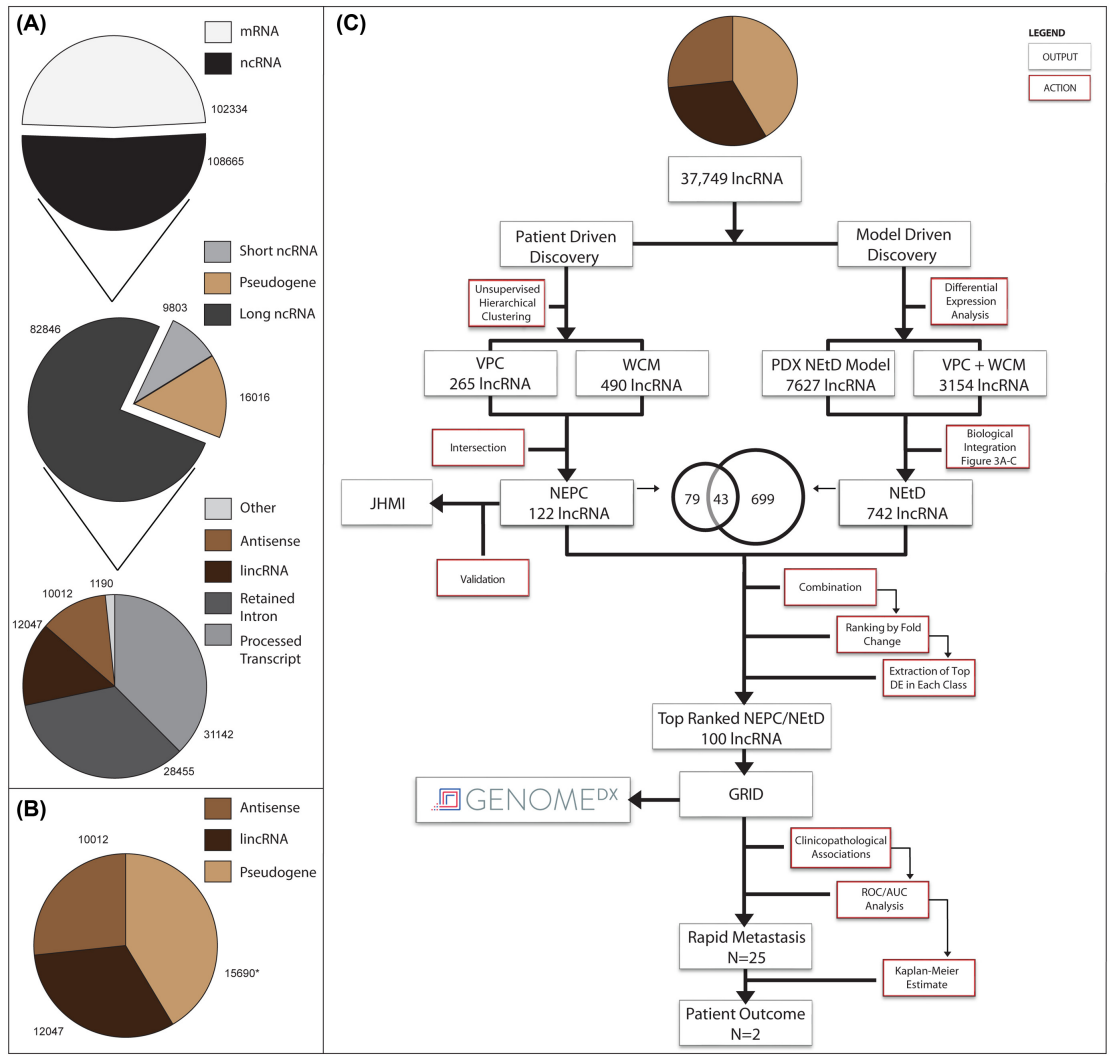
Transcriptome composition and study design. A) Proportions and totals of transcripts detected using our sequence analysis pipeline. Transcripts were separated into protein coding (mRNA) or non-coding RNA (ncRNA) and as defined by Ensembl's core biotypes as either mRNA, lncRNA, short ncRNA, or pseudogene. Within lncRNA, there exist seven classes, including processed transcripts, retained intron, lincRNA, antisense, sense intronic, sense overlapping, and three prime overlapping ncRNA (the last three labelled as “other”). Transcript totals are denoted around each pie slice. B) The three transcript classes used in this study due to their ability to separate AD and NEPC tumours, which collectively totalled 37,749 lncRNAs. *The pseudogene total was the combination of eight pseudogene subclasses and collectively referred to as pseudogene here. These subclasses include processed pseudogene, unprocessed pseudogene, transcribed unprocessed pseudogene, transcribed processed pseudogene, translated processed pseudogene, polymorphic pseudogene, unitary pseudogene, and pseudogene. These lncRNAs formed the basis for all down-stream analysis and C) the studies project workflow and study design. AUC, area under the curve; GRID, GenomeDx Decipher GRID database; JHSM, Johns Hopkins School of Medicine; PDX, patient derived xenograft; ROC, receiver-operating characteristic. See Table 2 for cohort clinical features and compositions.

To identify lncRNAs involved in NEPC, we performed next-generation polyadenylated RNA sequencing on the PDX CRPC models and patient samples. We implemented a sequence analysis pipeline composed primarily of algorithms from the Tuxedo suite of analysis tools [45]. Typically, lncRNAs are expressed at low levels, so the pipeline was augmented to include windowed-adaptive quality control corrections (see Methods and Supplementary Figs S1 and S2) that increase the ability to detect low abundance transcripts. We applied this pipeline to all PDX (n = 10) and clinical specimens (n = 117) acquired from the Vancouver Prostate Centre (VPC) and Weill Cornell Medicine (WCM) (Tables 1 and 2). Using a *quasi de novo* mapping strategy combined with amalgamating all sample transcriptome assemblies, we identified 210,999 annotated transcripts spanning 38 Ensembl transcript classes. Defined by Ensembl's core biotypes, transcripts are classified as either protein-coding RNAs, lncRNAs, short ncRNAs, or pseudogenes, which totaled 102,334 (48%), 82,846 (39%), 9,803 (5%),and 16,016 (8%), respectively (Fig. 1A, pie chart 1–2). Within lncRNA, seven subclasses exist: processed transcripts (n = 31,142), retained intron (n = 28,455), lincRNA (n = 12,047), antisense (10,012), sense intronic (n = 821), sense overlapping (n = 340), and three prime overlapping ncRNA (n = 29) (Fig. 1A, pie chart 3; due to their small totals, sense intronic, sense overlapping, and three prime overlapping ncRNAs are labeled as “Other”). Despite pseudogenes not being included within Ensembl's lncRNA classes (listed above), they are by definition considered under the umbrella of lncRNA [34].

**Table 1:** Xenograft model systems used in the study

			Resistance				Molecular Characteristics										
Name	Model System Name	Source	Phenotype	AN	TE	EZ	BI	AR	PSA	SYP	SPINK1	ERG	TMPRSS2-ERG	PTEN	PTEN GENE	p53	RB
LTL313B	313	Primary PCa	AD	-	-	-	-	+	+	-	-	+	+	-	-/-	M	WT
LTL313BR	313	LTL313B	CRPC	-	+	+	+	+	+	-	-	+	+	-	-/-	M	WT
LTL418	418	Primary PCa	AD	-	-	-	-	+	+	-	+	-	-	+	+/+	WT	WT
LTL418BR	418	LTL418B	CRPC	-	+	-	-	+	+	-	--------------------------Same as parental xenograft above--------------						
LTL331-3	331	Primary PCa	AD	-	-	-	-	+	+	-	-	+	+	-	-/-	M	M
LTL331-7	331	Primary PCa	AD	-	-	-	-	+	+	-	-	+	+	-	-/-	M	M
LTL331-5-8 week	331	LTL331-5	AD	-	-	-	-	+	-	-	------------------------Same as parental xenograft above---------------						
LTL331-5-12 week	331	LTL331-5	AD	-	-	-	-	+	-	-							
LTL331-3-R	331	LTL331-3	NEPC	-	+	-	-	-	-	+	-	-	+	-	-/-	M	M
LTL331-7-R	331	LTL313-3-R	NEPC	-	+	-	-	-	-	+	-	-	+	-	-/-	M	M

AR^+^ and AR^−^ CRPC xenograft model samples and their associated molecular characteristics. AD, adenocarcinoma; AN, grows in the absence of androgen; BI, resistant to Bicalutamide; CRPC, castration-resistant prostate cancer; EZ, resistant to Enzalutamide; M, mutation present; NEPC, neuroendocrine prostate cancer; TE, grows in the absence of supplemented testosterone; WT, wild type.

**Table 2:** Clinical samples used in the study

				Treatment Status				Gleason Grade			Clinical End Points				
Institute	Cohort Name	Clinical Group	Total	Naive	NHT	ADT	RT	-6	7	8+	BCR	MET	PCSM	+RMET+ADT	-RMET+ADT
VPC	VPC	AD-NAIVE	56	56	0	0	0	23	0	33	_	_	_	_	_
VPC	VPC	AD-NHT	14	0	14	0	0	0	0	14	_	_	_	_	_
VPC	VPC	NEPC^a^	5	0	1	5	0	0	0	5	_	_	_	_	_
VPC	VPC	CRPC	5	3	2	5	0	1	1	3	_	_	_	_	_
WCM	RUBIN	NEPC	7	_	_	_	_	NA	NA	NA	_	_	_	_	_
WCM	RUBIN	AD	30	_	_	_	_	2	23	5	_	_	_	_	_
JHSM	LOTAN	AD2	17	_	_	_	_	0	0	12	_	_	_	_	_
JHSM	LOTAN	NEPC^b^	16	_	_	_	_	NA	NA	NA	_	_	_	_	_
GRID	MCI	AD	545	0	0	124	54	63	271	211	388	212	132	11	113
GRID	MCII	AD	232	0	0	77	24	18	117	97	124	75	34	19	24
		**Total**	**927**	**59**	**17**	**211**	**78**	**107**	**412**	**380**	**512**	**287**	**166**	**30**	**137**

Patient samples and their associated clinical variables including treatment status, Gleason grading, and clinical endpoints. AD, adenocarcinoma; ADT, androgen deprivation therapy; BCR, biochemical recurrence; CRPC, castration-resistant prostate cancer; MET, adenocarcinoma metastasis; NAÏVE, adenocarcinoma naïvely treated; NEPC, neuroendocrine prostate cancer; NHT, adenocarcinoma with neoadjuvant treatment; PCSM, prostate cancer specific mortality; +RMET+ADT, ADT-treated rapid metastasis (<36 months) with at least 10 years of clinical follow-up; -RMET+ADT, ADT treated no metastasis with at least 10 years of clinical follow-up.

a Patient overlap exists across different sites of metastasis.

b Contains a subset of mixed histology tumours (see Methods for breakdown). Cells with a dash are clinical features that are unknown for cohort.

For each of the eight lncRNA subclasses and their corresponding transcripts, we performed unsupervised hierarchical clustering (UHC) and principle component analysis (PCA) on the VPC and WCM cohorts (see Methods: Statistical analysis). Five were incapable of distinctly separating NEPC and AD clinical samples due to insufficient transcript counts, incorrect transcript classification, or in general poor transcript annotation. The remaining three subclasses were capable of separating NEPC and AD (Supplementary Figs S3 and S4) and became the focus of all downstream analysis. These three lncRNA subclasses, antisense (n = 10,012), pseudogenes (n = 15,690), and lincRNAs (n = 12,047),are collectively referred to as lncRNAs here on in (n = 37,749 transcripts in total; Fig. 1B). It should be noted that immunoglobulin and T cell receptor genes (n = 326) were removed from the pseudogene transcript total. We explored these lncRNAs in our samples through two analytical workflows (model-based discovery and patient-based discovery), which we later merged for clinicopathological analysis. The outline presented in this figure represents the study's overall workflow (Fig. 1C).

### A lncRNA expression signature for NEPC

Recently it has been shown that AR^−^ and AR^+^ CRPC share substantial genomic overlap yet display significant epigenetic differences [32]. Here, we hypothesized that the lncRNA transcriptome would similarly show unique and common expression alterations between AR^+^ and AR^−^ CRPC (unexplored to date). To investigate this, we used the AR^+/−^ CRPC xenograft models (Table 1) to identify changes occurring temporally within the same tumour pre- and post-castration. Once castrated the three AD models (LTL313, LTL418, and LTL331) progress to either AR^+^ CRPC (LTL313BR and LTL418BR) or AR^−^ CRPC/NEPC (LTL331R). This allowed for the identification and quantification of differentially expressed transcripts between pre- and post-CRPC. We integrated this data with patient tumour data having matched clinical information to ensure the results were clinically relevant and to remove any model-based bias. As we suspected, of all lncRNAs altered between pre- and post-CRPC (>2 fold, *P* < 0.05), only 8% (n = 300) were commonly deregulated in both CPRC subtypes. The remaining transcripts (n = 2,669) displayed unique changes in the AR^+^ or AR^−^ CRPC subtype (Supplementary Table S2 and Supplementary Fig. S5). These data support the notion that AR^+^ and AR^−^ CRPC contain largely distinct lncRNA landscapes.

**Figure 2:**
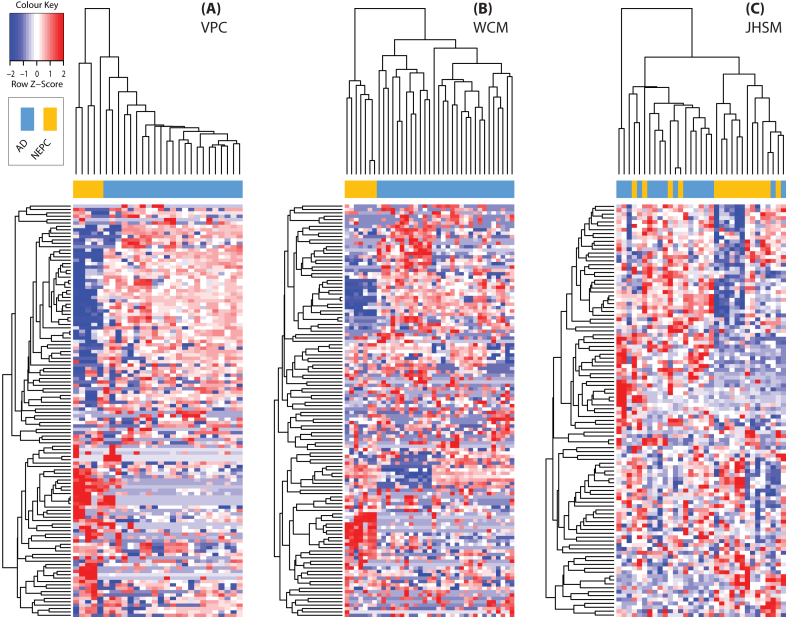
NEPC lncRNA expression signature and clinical classifier. Unsupervised hierarchical clustering of the 122 identified lncRNAs from (A) VPC and (B) WCM cohorts. Validation of this signature was shown in the (C) JHSM cohort. Samples (columns) are labelled as adenocarcinomas (blue) or neuroendocrine (yellow) tumours. See Supplementary Fig. S3 for row/lncRNA labels for each plot.

LncRNA expression may be useful as additional biomarkers beyond those currently used in the diagnosis of NEPC (i.e., CGHA, SYP, and NSE). Moreover, an lncRNA expression signature would strongly support the involvement of lncRNAs in NEPC at a molecular and cellular level. These lncRNAs would be candidates for mechanisms in the activation of a developmental pathway and/or plasticity involving previously identified protein-coding genes (PEG10, HP1α, NMYC, SOX2, SRRM4, REST, BRN2, etc.) in NEPC/NEtD. Conversely, since some of these genes (NMYC, SOX2, BRN2, and SRRM4) are well-studied transcription or splicing factors, NEPC lncRNA could be under their regulation. To build an lncRNA expression signature for NEPC, we selected the top fifth percentile of transcripts based on standard deviations of expression for the VPC and WCM cohorts independently and performed UHC. All uncharacterized transcripts (i.e., RP##-######.#, AC######.#, etc.) were removed from the analysis at this point. This produced 265 and 490 NEPC lncRNAs in the VPC and WCM cohorts, respectively. Taking the intersection of these lists and then repeating UHC generated an expression signature of 122 lncRNAs (Supplementary Table S5) that distinctly segregated NEPC from AD tumours (Fig. 2A and B). To assess the robustness of this signature, we validated it on an external clinical cohort of tumours (n = 33 ; Table 2) from Johns Hopkins School of Medicine (JHSM). These tumours contained 17 AD and 16 NEPC samples and were profiled on the Human Exon array 1.0 ST platform (see Methods) compared with the sequenced discovery cohorts. Using the same approach (UHC), a clear separation of NEPC and AD was observed (Fig. 2C). Observing consistent results across different technologies, institutes, and clinical samples further strengthens the robust nature of the NEPC signature. To our knowledge, this is the first report of lncRNAs exhibiting a unique, unbiased expression classifier capable of segregating NEPC and AD patient samples.

Some lncRNA from the patient-derived signature have been previously reported as altered in other cancer types. These lncRNAs include MALAT1 (alias NEAT2), PCA4 (aliases GDEP, PCAN1, or PCAT4), DSCAM-AS1, and SNHG12. MALAT1 is one of the most well-characterized and studied lncRNAs in cancer and has been identified as a regulator of metastasis and cell migration, a prognostic marker, and a transcriptional regulator of alternative splicing in lung cancer [46]. PCA4 has been identified as a prostate and retinal specific transcript [47] and frequently mutated in PCa [48]. DSCAM-AS1 mediates tumour progression and tamoxifen resistance in breast cancer through interacting protein hnRNPL [49]. SNHG12 is induced by c-MYC and regulates cell proliferation, apoptosis, and migration in triple negative breast cancer [50]. We were interested in identifying the most highly expressed lncRNAs in the signature. Therefore we ranked each according to their fold changes when compared with AD samples, required concordance in fold changes across both of the cohorts, and >10-fold change in magnitude. H19, LINC00617 (alias TUNA/TUNAR), NKX2–1-AS1, and SSTR5-AS1 were the only four that fit these thresholds and each with previous reports in cancer. Of note, H19 is the most studied among the four lncRNAs and is implicated in numerous cancer types [51]. It is involved in proliferation and both differentiation processes related to metastasis, epithelial-to-mesenchymal transition (EMT), and mesenchymal-to-epithelial transition (MET) [52]. LINC00617 in breast cancer regulates EMT, cancer progression, and metastasis through activation of the transcription of SOX2 [53]. SSTR5-AS1 has not been functionally characterized, but its sense form SSTR5 has and is a biomarker for neuroendocrine tumours [54]. In fact, recently it has been used to evaluate SSTR-targeted therapy for neuroendocrine tumours in circulating tumour cells [55], and its use in patient management is being tested in a Phase IV clinical trial (NCT02075606). Overall, the identification of the NEPC lncRNA expression signature has provided a previously unexplored component of the NEPC transcriptome, revealed candidate NEPC biomarkers, and associations to NEPC biology.

### Distinct expression profiles of lncRNAs are associated with NEtD

**Figure 3:**
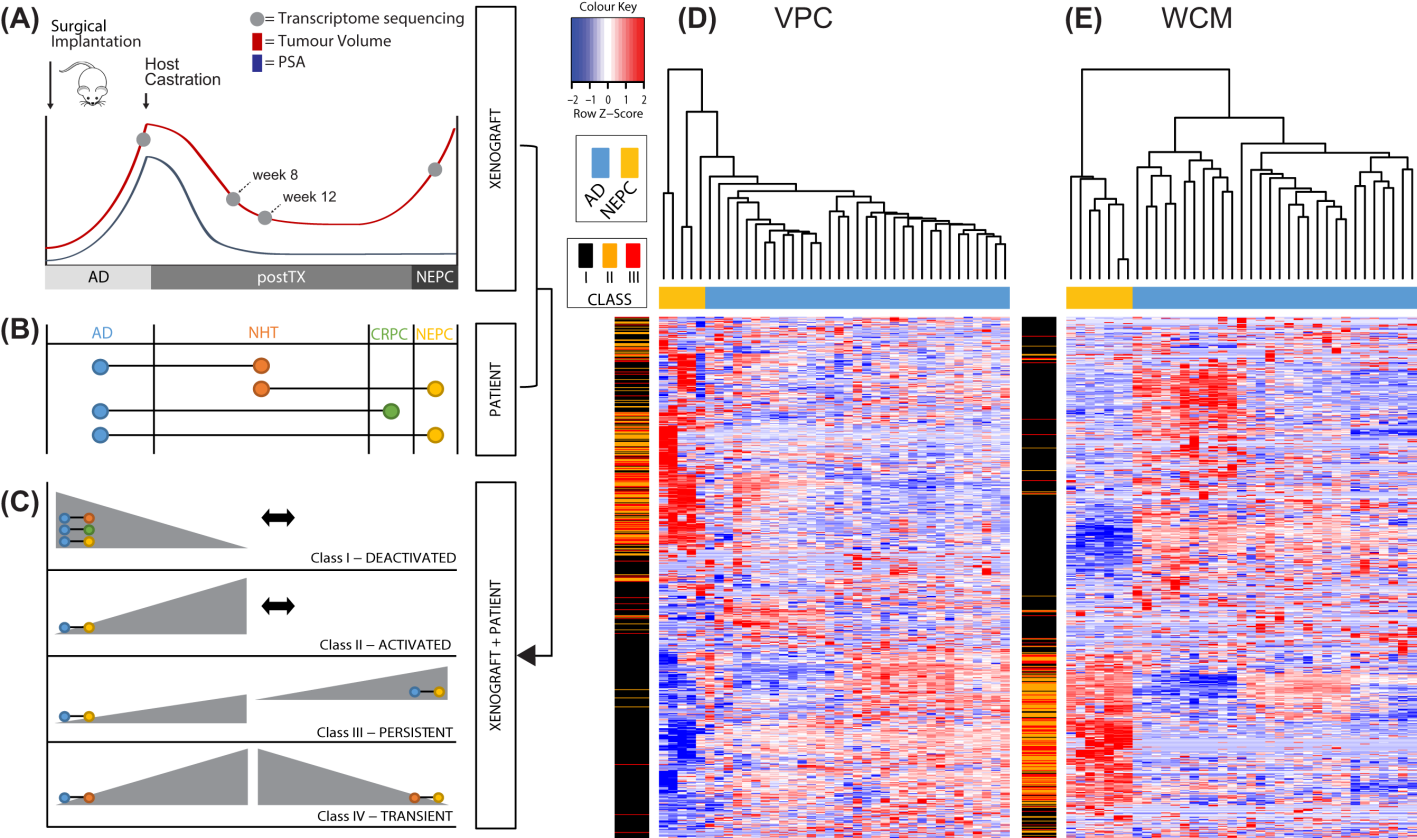
Xenograft model of neuroendocrine transdifferentiation, phenotype-driven data integration, and NEtD-associated lncRNAs. A) Schematic depicting the time points at which xenograft tumours were collected along the transdifferentiation of AD to NEPC (adapted from Akamatsu et al., 2015 [24]). B) Phenotypes for clinical samples (coloured circles) that align to various time points from above xenograft model and group-wise comparisons (black connector bars) analyzed for clinical samples. C) Four isolated expression profiles (grey triangles) from select time points in A (light grey circles) with appropriate clinical group-wise comparisons overlaid and integrated. Unsupervised hierarchical clustering with NEtD lncRNAs (Class I, Deactivated: black bars; Class II, Activated: orange bars; and Class III, Persistent: red bars) identified from integration outlined in (C). Distinct clusters of AD and NEPC clinical samples are observed in the (D) VPC and (E) WCM cohorts. Class IV, Transient lncRNAs were excluded from the clustering due to the lack of clinical samples that would represent this intermediate state.

A major goal of this study was to characterize the lncRNA landscape during the dynamic phase transition from AD to NEPC using our unique PDX model LTL331 [7] (Fig. 3A). To accomplish this, we sequenced six samples of our PDX NEtD model representing three primary time points along disease progression: two samples from each terminal point (AD and NEPC) and two samples post-castration (postTX). Time points 8- and 12-week post-castration were selected to represent postTX due to tumour volume and serum PSA levels reaching nadir (Table 1 and Fig. 3A). We identified and quantified all lncRNA transcripts that were altered across the time series and defined four patterns of transcript expression: (a) continuous decline in expression (Class I, Deactivated, n = 1,613); (b) increasing expression from either AD to postTX or postTX to NEPC (Class II, Activated, n = 4,281); (c) continuous increased expression (Class III, Persistent, n = 1,054); and (d) maximum expression at postTX (Class IV, Transient, n = 2,668)(total n = 7,627;Fig. 3A). The NEtD model and postTX state represents a biological process that currently is not characterized as a clinical entity but offers invaluable insight into the transcriptome of transdifferentiating AD cells.

To determine the clinical relevance of Class (I–IV) lncRNAs, we integrated patient samples (VPC and WCM, Table 2 - column “Clinical group”) with time points in our model (see Fig. 3B for alignment of time points to patient groups). Terminal time points were appropriately aligned to AD and NEPC samples. However, due to the lack of clinical specimens undergoing NEtD, we hypothesized that neoadjuvant hormone therapy (NHT) given to AD patients may exhibit characteristics of the postTX state. The transcriptomes from these patients have been shown to display the effects of therapy response and more specifically androgen depletion [56]. In fact, neuroendocrine differentiation has been shown to increase after only three months of NHT in a retrospective analysis of 103 radical prostatectomy specimens [57]. These early events are the specific alterations we sought to isolate from the postTX time points of our PDX model. We also postulated that a subset of Class I (down-regulated in our PDX model) would be up-regulated in the (AR^+^) CRPC clinical samples due to reactivation of the AR signalling axis in classical CRPC [56, 58–60]. Based on this model-to-patient data integration, the following patient group-wise comparisons were performed: (a) NEPC vs AD, (b) NEPC vs NHT, (c) CRPC vs AD, (d) NHT vs NAÏVE (untreated AD), and (e) NHT vs NAÏVE in combination with NEPC vs NHT. This produced 1,927;713; 975; 1,045; and 117 transcripts, respectively (>2 fold with *P* < 0.05; total n = 3,154;Fig. 3B; Supplementary Table S4). Integrating these results with the PDX NEtD model transcripts above led to 475, 222, 84, and 45 lncRNAs identified within Class I (Deactivated), Class II (Activated), Class III (Persistent), and Class IV (Transient), respectively (total n = 742; Fig. 3C). Unsupervised hierarchical clustering of Class I-III within WCM (Fig. 3D) and VPC (Fig. 3E) cohorts exhibited (as expected) a distinct separation of AD and NEPC tumours and a distinct separation between lncRNAs in Class I-III (rows of heat map). Class IV transcripts were excluded from this illustration due to their lack of altered expression between AD and NEPC clinical samples. Collectively these 742 NEtD lncRNAs are associated with the pathogenesis of tNEPC.

**Figure 4:**
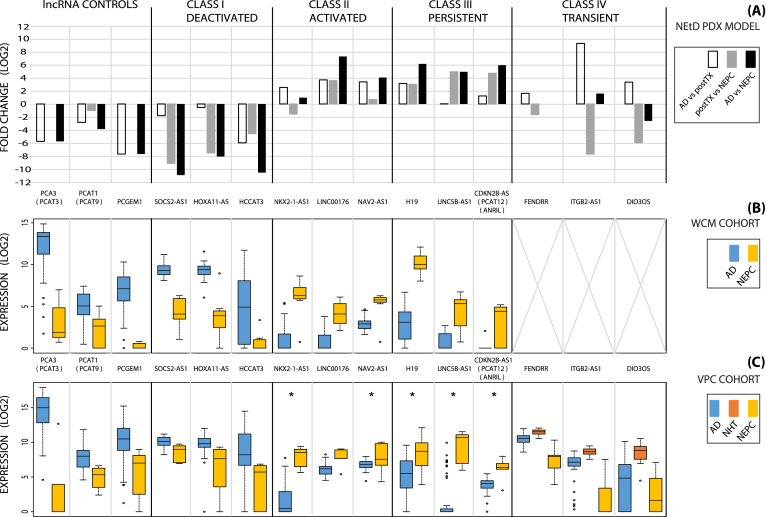
Select NEtD lncRNAs that exemplify each expression pattern are shown from the (A) NEtD PDX LTL331 model, (B) WCM cohort, and (C) VPC cohort. The expression for NEtD lncRNAs within Class IV, Transient was only identified through the VPC cohort due to the presence of NHT samples, which were not present within the WCM cohort. All boxplots showed significant separation (*P* < 0.05) between groups based on a standard Student's *t*-test with the exception of * lncRNAs.

Prominent examples identified by this biological integration of our NEtD model (Fig. 4A), WCM cohort (Fig. 4B), and VPC cohort (Fig. 4C) illustrate each of these NEtD-defining transcript classes. PCA3, PCAT1, and PCGEM1 were selected as controls for this study due to their elevated expression in PCa and high level of characterization. As expected, their expression patterns followed the trend in the PCa and NEPC samples (Fig. 4A–C, NEtD controls; *P* < 0.001). SOCS2-AS1 and HOXA11-AS are select examples that characterize the NEtD lncRNA Class I Deactivated (Fig. 4A–C, Deactivated; *P* < 0.01). HOXA11-AS, associated with the cell cycle through E2F1 [61], has been seen to promote gastric cancer proliferation and invasion (with EZH2) and can act as a “molecular sponge” for EZH2 by absorbing (via direct interaction) miR-1297 [62]. SOCS2-AS1 is another lncRNA in this class that has been identified as an AR-regulated transcript [63] and further supports our hypothesis of AR-regulated lncRNAs within NEtD Class I Deactivated. NKX2–1-AS1 exemplifies the NEtD Class II Activated (Fig. 4A–C, Activated; *P* < 0.05) and has been previously seen to characterize lung cancer subtypes AD and squamous [64]. CDKN2B-AS1 (alias ANRIL) and H19 are prime illustrations for persistently expressed NEtD Class III (Fig. 4A–C, Persistent; *P* < 0.05). Both of these lncRNAs have been identified across a number of cancer studies (H19 [51], ANRIL [65, 66]);however, depending on the cancer type, each has functioned as a tumour suppressor (i.e., ANRIL deactivating tumour suppressors CDKN2A/B in cis by three different epigenetic mechanisms [67–69]) and as an oncogene (i.e., H19 acts as a sponge for FOXM1 by absorbing miR-342–3p [70]). Two demonstrations for transiently expressed NEtD lncRNA Class IV are FENDRR and CASC15 (Fig. 4A–C, Transient; *P* < 0.01). These lncRNAs are well studied in cancer: FENDRR for its prognostic value and its involvement in gastric cancer metastasis [71] and CASC15 for its regulation of SOX4 in RUNX1-rearranged leukemia [72] and harboring a risk SNP for susceptibility of neuroblastoma [73]. CASC15 has also been identified as a mediator of neural growth and differentiation [74], which we believe could be occurring in our NEtD model based on the data presented here. Each of these lncRNAs are among the top candidates identified in this study and a focus of our future research and functionalization. Taken together, these NEtD lncRNAs (n = 742) characterize the transdifferentiation that occurs post-castration and is associated with tNEPC.

### NEtD lncRNAs are enriched with distinct transcription factor binding motifs

LncRNAs are not translated and carry out their functions post-transcription in their secondary or tertiary RNA form. This is unlike protein-coding transcripts that function in their post-translational form. Thus, identifying sequence motifs within lncRNAs could identify interacting transcripts or proteins that provide clues to function. Enrichment of transcription factor (TF) binding sites (TFBS) was determined by calculating z-scores for overrepresentation of motifs present in the NEtD lncRNA Classes (I–IV) against their genomic background (Supplementary Tables S6–S17 and Methods: Genomatix overrepresented TFBS). We also integrated each of these class-specific enrichment results to identify unique TFBS for each NEtD Class (Supplementary Tables S18 and S19). All TF and TFBS descriptions, family classifications, and annotation is described in Supplementary Table 25.

In NEtD Class I we identified 33 significant and uniquely enriched TFBS (Supplementary Tables S6, S18, and S20). Interesting results included binding motifs for TP53, scratch family transcriptional repressor 2 (SCRT2), and POU Class III homeobox 3 (POU3F3) (z-scores = 4.02, 4.27, and 2.41, respectively). TP53 often absent in NEPC, could be an activating TF for many of these Deactivated lncRNAs, and suggests an apoptosis or cell cycle arrest role is present here. Scratch family transcriptional repressor 2 has been linked as a neural-specific Snail family transcriptional repressor and critical for neuronal differentiation [75]. Similar to REST, this TF is likely causing the down-regulation of a subset of these lncRNAs. Lastly, POU3F3/BRN1 (a member of the POU family of TFs, as is BRN2) is involved in the development of the nervous system, expressed in small cell lung cancer cells (which has pathological overlaps with NEPC), and involved in proneural/neuroendocrine differentiation [76]. Considering this and the significant enrichment of these TF motifs, this suggests a role in proliferation and differentiation in NEtD Class I.

Performing TFBS enrichment analysis in NEtD Class II and III identified 12 and 15 significant and distinct TFBS motifs, respectively (Supplementary Tables S7, S8, S18, and S20). Interestingly, both classes had significant enrichment for at least one ETS and HOX family member, suggesting overlapping functional roles for their respective lncRNAs. For Class II this included ELF5, SPIC, and HOXD1 (z-scores = 2.17, 2.43, and 2.22, respectively) and for Class III, PDEF (alias SPDEF) and HOX/PBX (z-scores = 3.03 and 2.71, respectively). Members of the ETS family fused to TMPRSS2 is the most frequent genomic alteration in PCa; therefore the prevalence of their motifs in these classes is not surprising. While the ETS fusion transcript is relatively more specific to PCa vs NEPC, ETS TFs on their own are involved in a wide variety of functions, including cellular differentiation and angiogenesis. In fact, recently SPDEF was found to be down-regulated in metastatic NEPC due to DNA methylation [32] and was also significantly down-regulated in treated vs untreated high-risk PCa patients [77]. Conversely, the HOX family has never been linked to PCa or NEPC for that matter, and so this result was unexpected. In neuroblastoma, however, the HOX genes have been linked to differentiating cells [78] and specifically HOXD1 identified here (as well as HOXC6 and HOXD8) are associated with differentiation towards a neuronal phenotype [79].

Performing TFBS enrichment analysis in NEtD Class IV identified enrichment of 17 distinct TFBS motifs (Supplementary Tables S9, S18, and S20). Class IV transcripts are only expressed during treatment (castration) response. Interestingly, heat shock TFs HSF1 (z-score = 2.06) and HSF2 (z-score = 3.87) were within these results. Heat shock proteins (HSPs) are expressed at low levels under normal conditions, up-regulated by cellular stress, and function as molecular chaperones to control client protein stability and function. Their candidacy as therapeutic targets has been well studied in PCa [80] and AR^+^ CRPC [81]. In breast cancer, HSF1 specifically induces a cancer stem cell phenotype *in vitro* [82]. In PCa, HSPs bind dihydrotestosterone to the AR and enhance AR-mediated transcription. One of the functions of lncRNAs is to facilitate this type of mechanism. For example, LINC00152/CYTOR (identified within this class) binds and recruits EZH2 to its target promoters p15 and p21 in gastric cancer [83] and IL24 in lung cancer [84] and thereby causes repression of their expression. Considering the transient expression of the lncRNAs in this class, this data suggest a subset may be stress response mediators via HSPs. Lastly, FOXA1 showed a significant enrichment (z-score = 2.8) in this class. Recently, FOXA1 loss was identified as a driver of NEtD [23], which leads to AR reprogramming [85] and EMT through direct regulation of SLUG expression [86]. This suggests that some of the lncRNAs in this class could have a functional role in maintaining cellular identity when under the control of FOXA1.

With FOXA1 as one of the characterizing TFBS in Class IV, we sought to explore the persistently expressed (Class III) in conjunction with the transiently expressed transcripts (Class IV). We hypothesized that subsets of these lncRNAs have mechanistic involvement in the transdifferentiation process. To investigate this we repeated the TFBS enrichment analysis on Class III and IV together and identified six significantly enriched TFBS (Supplementary Tables S10, S18, and S20). Confirming our hypothesis was the presence of TWIST1 (z-score = 4.01), an essential member of the EMT transcriptional reprogramming factors [87]. Interestingly, TWIST1 and AURKA have very recently been seen to form a feedback loop promoting metastasis and highly aggressive phenotypes in pancreatic carcinoma [88], and TWIST1 is a marker for EMT in neuroendocrine tumours [89, 90]. Concerning PCa, it has been identified as AR regulated (and repressed via NKX3–1), whereas in the absence of AR it is up-regulated and present in metastatic disease [91].

We further investigated global functional characteristics across all NEtD lncRNAs. Specifically, we wanted to identify TFBS that were significantly enriched and common across all classes. Due to the high number of lncRNAs (n = 2,147), we decided to perform this analysis at the TF family level; therefore for each class and the full lncRNA set, we repeated the motif enrichment analysis and integrated all of their results (Supplementary Tables S12–S17, S19, and S20). We identified 62 significantly common TFBS families (z-score = >2;Supplementary Table S20). Not surprising were families involving cell cycle regulation, cyclin B2/CCNB2 and the E2F family (z-scores = 40.66 and 67.36, respectively). We also observed both the ETS (z-score = 9.9) and REST (z-score = 20.52) families of TFs, which reaffirmed our hypothesis that these lncRNAs are involved in tumour progression and neuronal pathways. Surprising was the presence of two PAX families, PAX5 (z-score = 10.15) and PAX9 (z-score = 18.18). The PAX family is known to regulate lineage specification and progenitor cell maintenance. In developmental biology, PAX5 is involved in B-cell differentiation and PAX9 in neural crest development. PAX5 has been observed as overexpressed in other neuroendocrine tumours [92, 93], overexpressed in neuroblastoma [9,4], and shown to positively regulate c-Met transcription in small cell lung cancer [95]. In lung NETs, PAX6 is prognostic for aggressiveness [96]. Their role in NEPC is yet to be characterized; however, evidence here supports their global involvement in NEtD and lncRNA function. Lastly, the selenocysteine tRNA activating factor (STAF, z-score = 15.18) was very intriguing to us. A recent Nature study by Schreiber et al. suggested that treatment resistance in NEtD of PCa depends on a druggable lipid-peroxidase pathway that protects against ferroptosis (a non-apoptotic form of cell death) [33]. The increased lipid metabolism creates a dependency on GPX4, which prevents ferroptosis. GPX4 is a selenocysteine-containing enzyme and 1 of only 25 proteins with this rare amino acid in the entire human genome. The data presented here suggest that some of these lncRNAs may be involved in the selenocysteine pathway via STAF and in selenoprotein biosynthesis of molecules (i.e., GPX4). Identifying and targeting these lncRNAs could be a path for upstream inhibition of GPX4 up-regulation and therefore allow cell death in these resistant cells to occur naturally by ferroptosis. Comprehensive *in vitro* experimentation would need to be carried out to confirm this therapeutic avenue.

### NEtD lncRNAs contain NEPC-related TFBS

It is now well established that complex cellular reprogramming occurs during NEtD, and master regulators such as REST [31], BRN2 [22], SOX2 [28], and SOX11 [18] have been identified as key TFs involved in this process. Identification of well-known TFBS such as these would test our current hypotheses on the functional involvement of individual lncRNAs in NEtD pathogenesis (Supplementary Tables S21–S24). TFBS identification was carried out using MatInspector [97–99] (see Methods: Genomatix Matbase and MatInspector) on each of the NEtD Class for select TFs. As before, all TF and TFBS descriptions, family classifications, and annotation is described in Supplementary Table 25.

With the dominance of AR-regulated genes in AD, the lack of expression that defines Class I NEtD lncRNAs is likely caused by the absence of androgen (post-castration), and therefore these lncRNAs are putatively AR-regulated. To test this, we searched for androgen and glucocorticoid response elements (ARE and GRE, respectively). The results showed that 107 lncRNAs contained ARE and/or GRE motifs, of which 16 contained only an ARE motif, 49 contained only a GRE motif, and 21 contained both ARE and GRE motifs (Supplementary Table S21). To further test and support our AR-regulated lncRNA hypothesis, we explored all previously reported AR-regulated lncRNAs. Currently, the following 17 lncRNAs have been identified with experimental evidence: PCGEM1 [100], PlncRNA-1/CBR3-AS1 [101], PCAT-18 [102], PCAT29 [103], SOCS2-AS1 [63], RP1–45I4.2 [104], SUZ12P1 [104], SNHG5 [104], LINC01138 [104], SNHG1 [104], KLKP1 [104, 105], LINC00969 [104], LINC-PINT [104], TUG1 [104], MIR17HG [104], POTEF-AS1 [106], and CTBP1-AS1 [107]). Of these, 4 (PCAT29, SUZ12P1, SNHG1, and CTBP1-AS1) were not within our pipeline lncRNA class annotation, and 8 of 13 (61%) were represented in NEtD Class I Deactivated lncRNAs (PCGEM1, PlincRNA-1, PCAT-18, SOCS-AS1, KLKP1, LINC00969, LINC-PINT, and POTEF-AS1). Due to our integrative study design (Fig. 3A–C), the remaining 5 did not move forward in the analysis. However, removing the integrative steps, down-regulation of these lncRNAs did occur in either our model or patient samples independently. Overall, of the 13 lncRNA annotated by our pipeline and reported as AR regulated, all overlapped in this study.

Due to the elevated pattern of expression that defines Class II and III NEtD lncRNAs, we hypothesized that a subset of these lncRNAs are constituents of the neuronal phenotype present in NEPC. To test this hypothesis, we analyzed these lncRNAs for the presence/absence of the following select TFs known to activate this cell type: APOU Class III homeobox 2 (POU3F2), also known as BRN2, and RE1 silencing transcription factor (REST). Activation of BRN2 and deactivation of REST are involved in neuronal differentiation and regulation of neurogenesis, respectively. Again, using the MatInspector algorithm, we identified 11, 22, and 21 lncRNAs in Class II or III with TFBS for BRN2, REST, or both, respectively (Supplementary Table S22). Taken together, this evidence supports involvement for a subset of these lncRNAs to neuronal function/pathways in NEtD.

**Figure 5:**
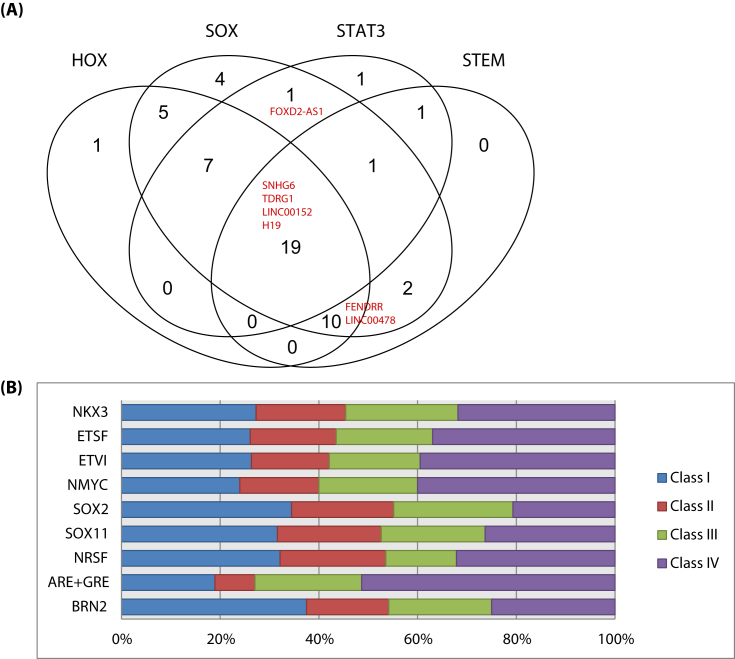
TFBS Venn diagram and distribution plots. A) Common and unique TFBS for HOX, SOX, STAT3, and STEM families of transcription factors within Class III and IV of the NEtD lncRNAs. B) Distribution of TFBS for known NEPC-involved TFs within NEtD Class lncRNAs.

To further support the hypothesis of mechanistic involvement for the NEtD process in Classes III and IV, we expected TFBS related to plasticity and stemness to be present. Therefore, we used MatInspector to identify binding motifs for members of the following well-studied cellular differentiation TF families: HOX [108], SOX, STAT3 [109], and “STEM” (STEM members are defined by Matbase and include POU5F1/OCT4, SALL4B, SOX2, NANOG, and TCF7L1). We observed 42, 49, 30, and 33 lncRNAs with TFBS for HOX, SOX, STAT3, and STEM genes, respectively (Supplementary Table S23). In fact, some lncRNA had TFBS within more than one of these TFs (Fig. 5A). Previous studies have linked 6/7 of these (highlighted in Fig. 5A) to various components of EMT and/or cellular plasticity. FENDRR (antisense lncRNA to FOXF1) regulates gastric cancer metastasis via fibronectin1 [71]. FOXD2-AS1 regulates EMT and Notch signaling to promote colorectal cancer [110]. H19 has been identified as a mediator of breast cancer plasticity during EMT and its reverse process MET [111], as well as having a role in stemness in prostate cells [112]. LINC00152 is involved in EMT (combined with cell migration and invasion) in gastric cancer [113]. LINC00478 (alias MONC) interferes with hematopoietic lineage decisions and enhances proliferation of immature progenitor cells in acute megakaryoblastic leukemia [114]. Lastly, again in gastric cancer, lncRNA SNHG6 has been seen to promote cell proliferation and EMT [115]. Based on these data, Class III and IV lncRNAs could have a role in developing a cellular “plastic” state during NEtD.

To test the involvement of known NEPC-involved TFs in all NEtD lncRNA, we searched for BRN2, ARE/GRE, REST, SOX11, SOX2, NMYC, ETVI, ETS, and NKX3 motifs (Supplementary Table S24). Since each of the classes had different sizes, this would influence the distribution and presence/absence of these motifs, so we extracted the top 25 lncRNA within each class (n = 100 NEtD lncRNA), ranked by their magnitude of fold change. Observing the distribution of these TFs separated by NEtD class revealed an interesting pattern (Fig. 5B). TFs SOX2, SOX11, and REST had a relatively more balanced distribution across each class compared to NKX3, ETSF, ETVI, and NMYC, which showed a preference to binding persistent and transiently expressed lncRNA. Interestingly, more than 50% of ARE/GRE motifs were present in transiently expressed lncRNA vs relatively few in Class I Deactivated and Class II Activated. Conversely, BRN2 motifs were relatively more present in Class I and II. These patterns suggest a time-dependent or cellular phase-dependent usage of TFs post-castration and during the NEtD process.

### NEPC and NEtD lncRNAs identify putative NEPC subtypes

To corroborate the lncRNA expression in an external NEPC (extNEPC) cohort [32], we visualized NEtD lncRNA Classes II-IV and the up-regulated NEPC lncRNA expression, including genomic profiles (copy number and mutation), through an OncoPrint schematic. The cohort consisted of 44 NEPC specimens (largest published to date) from 30 patients that were classified based on their histomorphology [6]. Due to the exome sequencing performed on this cohort, not all lncRNAs were represented/detectable in this sequencing profiling. We also plotted previously reported NEPC predisposing genes, oncogenes, drivers, and the TFs we identify above to provide “transcriptome context” for the altered lncRNAs (Supplementary Figs S6 and S7). From the 58 NEPC and 243 NEtD lncRNAs represented in the extNEPC exome sequencing profiling, 43% (25/58) and 27% (66/243) showed altered expression in 2–34% of NEPC patients, respectively (Supplementary Figs S6, S8–S11).

Surprisingly, these testable lncRNAs (58 from the NEPC lncRNAs and 66 from the NEtD lncRNA) in combination with known oncogenes/tumour suppressors/transcription factors (Supplementary Fig. S7) resulted in identifying three distinct subsets of NEPC patients within the extNEPC cohort. Group 1 had relatively higher mutation frequencies, higher ploidy, mixed tumour sites, and mixed pathological classifications. Group 2 had a relatively low mutation frequency and low ploidy, derived mostly from pelvic masses and with pathological classification D (large-cell neuroendocrine carcinoma). Group 3 tumours, however, were mostly derived from the prostate with a pathological classification B, and likely primary (*de novo*) NEPC samples where NEtD has not occurred. Of note, copy number loss or mutations in TP53 and RB1 were present in 60% of patients (26/44), spread across the cohort, and did not appear to be associated with a particular group (Supplementary Fig. S7). The three groups could be revealing an lncRNA expression signature that is specific for tumour site, degree of genomic mutations (SNPs or CNVs), and pathological classification. However, it is important to note this is an observational result requiring statistical validation in a larger cohort. The specificity of these genomic and lncRNA transcriptome profiles would need to be explored across a variety of metastatic sites and NEPC pathologies to validate these three novel NEPC molecular subtypes.

### NEPC and NEtD lncRNAs are associated with treatment-related metastasis

Prognostic and predictive biomarkers for NEtD and NEPC are in dire need since ADT is not effective for a cancer that has undergone NEtD and thus circumvents the AR signalling axis. We examined if the NEtD (n = 742) and NEPC (n = 122) lncRNAs are associated with NEPC related clinical outcomes in patients with primary prostatic adenocarcinoma. To accomplish this, we explored the candidates in two cohorts from the Mayo Clinic (MCI [116] and MCII [117]) from the Decipher GRID database (GRID) (n = 777, Table 2). We could not perform this analysis within VPC/WCM cohorts due to their small sample sizes and short-term clinical follow-up. The GRID cohorts represent tumours primarily with adverse pathology (i.e., high grade/stage) and long-term follow-up for treatment and outcomes (median 18 years). From these cohorts, a subset (n = 211) received adjuvant ADT post-radical prostatectomy (RP). To determine the most clinically relevant lncRNA transcripts, we first ranked the NEtD/NEPC lncRNAs within their respective classes and selected the top deregulated from each. The ranking was performed based on fold changes observed within the clinical groups (see Methods). This produced 100 top-ranking NEtD/NEPC lncRNAs that we investigated within the GRID cohorts (Fig. 1C and Supplementary Table S26). We validated 11 of these (2 from each NEtD Class and 3 from the NEPC lncRNA signature) by quantitative real-time PCR to confirm expression changes identified in the model and clinical samples (Supplementary Fig. S12). Due to the difference in profiling platforms between GRID (Affymetrix microarray) and VPC/WCM cohorts (Illumina Sequencing), it was necessary to remap the GRID microarray probes (see Methods) that aligned within NEtD/NEPC lncRNA sequenced regions. This resulted in 81/100 being present and quantifiable on the microarray platform.

**Figure 6:**
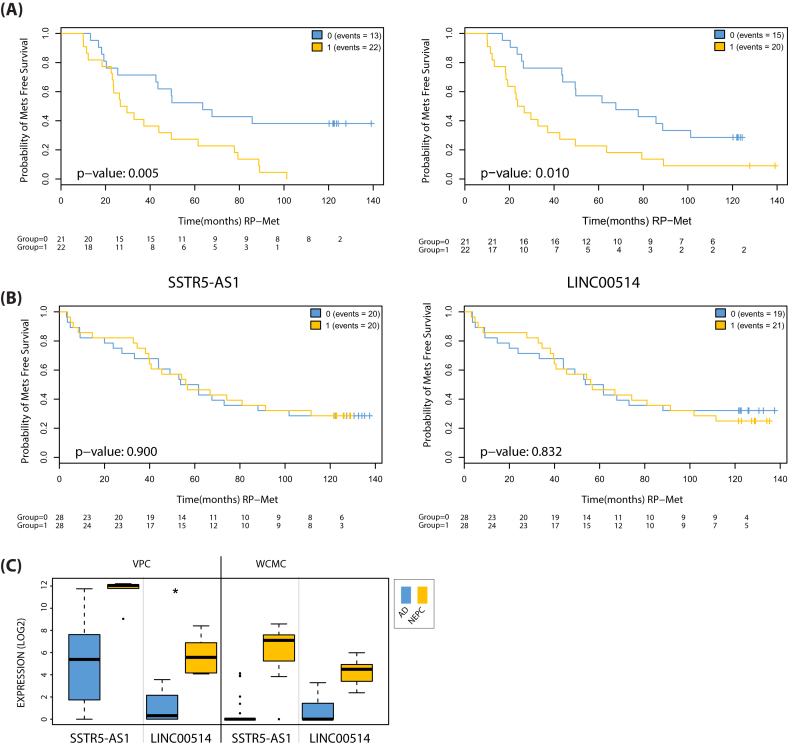
Kaplan-Meier estimates and expression for SSTR5-AS1 and LINC00514. Kaplan-Meier estimates for metastasis-free survival in the MCII cohort comparing low (blue lines) and high (yellow lines) expression (split by median) in (A) treated patients that received post-prostatectomy adjuvant ADT for SSTR5-AS1 (left) and LINC00514 (right) and (B) patients not receiving ADT treatment. C) Box plot expression for the top two NEPC lncRNA candidates (SSTR5-AS1 and LINC00514) within the VPC and WCM cohorts.

A characteristic of NEPC patients in the clinic is the occurrence of rapid metastasis following treatment [118], and so we first tested the lncRNAs' ability to predict rapid metastasis post-ADT. We performed receiver-operating characteristic (ROC) analysis to compare the sensitivity and specificity of predicting rapid metastasis (within 36 months) for each lncRNA. We then calculated the area under the curve (AUC) for each lncRNA ROC in both cohorts using probe set region expression summarized across the full lncRNA transcript (Supplementary Table S26). This identified eight lncRNAs with the highest scores: NR2F1-AS1, LINC00654, FENDRR, PCAT2, and NKX2–1-AS1 in MCI (AUC >0.70) and LINC00478, LINC00173, and LINC00514 in MCII (AUC > 0.70). These lncRNAs serve as candidates for predicting rapid metastasis in patients receiving ADT. Selecting all NEtD/NEPC lncRNAs with AUC >0.65 (n = 25), we performed survival analysis to ascertain their ability to separate patients for metastasis as an outcome and end-point. Specifically, we calculated Kaplan-Meier estimates for metastatic disease progression stratified by median expression in ADT-treated samples of the MCII cohort. The expression of two NEtD/NEPC lncRNA transcripts (SSTR5-AS1 and LINC00514) was able to separate patients more likely to develop metastatic disease from those that did not (*P* = 0.005 and *P* = 0.010, respectively; Fig. 6A). To increase our confidence that the results were associated with treatment status, we generated Kaplan-Meier estimates for these transcripts in untreated patients from the same cohort; neither showed significant separation in their performance (*P* = 0.905 and *P* = 0.832, respectively; Fig. 6B). Expression for SSTR5-AS1 and LINC00514 in the VPC and WCM cohorts illustrate their distinct and elevated expression in NEPC vs. AD patient samples (Fig. 6C). These results suggest a strong association between treatment status and increased probability of metastatic disease in patients with differential expression of these lncRNAs. This, together with results from the NEtD model and NEPC clinical samples, implicate SSTR5-AS1 and LINC00514 in NEPC and these lncRNAs serve as strong candidates as predictive biomarkers for metastatic disease post-RP following ADT.

One of the mechanisms observed with lncRNAs is direct RNA-RNA interaction with mRNA, resulting in regulation of their expression (activation or repression). This type of investigation is computationally intensive, and there are limited algorithms available to identify putative mRNA targets genome-wide. However, a method was recently published to predict lncRNA-mRNA interactions genome-wide [119], and so we sought to identify candidate mRNA transcripts interacting with SSTR5-AS1 and LINC00514. The pipeline's three core algorithms include Raccess [120] for the identification of accessible regions within the lncRNA, IntaRNA [121] to calculate nucleotide interaction energies, and RactIP [122] to predict joint secondary structures. Applying this methodology to SSTR5-AS1 and LINC00514 produced a list of predicted interacting partners for these lncRNAs (Supplementary Tables S27–S28). The top-ranked mRNAs were KDM4B and TADA3 that are predicted to hybridize and form joint structures independently with SSTR5-AS1 and LINC00514, respectively (Supplementary Figs S12 and S13). In the clinical cohorts, TADA3 is down-regulated in NEPC vs AD (>2-fold), while KDM4B is up-regulated (>5 fold); however, only the deregulation of TADA3 is statistically significant (VPC *P* = 0.003 and WCM *P* = 0.017). Both genes have NEPC associations (see Discussion), and our data suggest they are being regulated by these lncNRAs.

## Discussion

Primary NEPC arises *de novo* in 0.5% to 2% of all prostate cancer patients [123]. However, tNEPC can develop in 20–30% of metastatic castrate-resistant prostrate cancer tumours [124] and increases with disease progression [125]. The real incidence of tNEPC may be higher because of under-recognition due to tumour heterogeneity, the limited number of metastatic tumour biopsies performed, lack of uniform consensus definition based on histology or biomarker expression, and frequent misclassification as high-grade PCa (most notable in tumours with mixed histologies) [126]. NEPC can be induced *in vitro* in AR^+^ LNCaP cells in androgen-depleted culture conditions [127, 128], similarly *in vivo* [7, 129], and in patient tumours long-term ADT has increased neuroendocrine differentiation [118, 124, 130]. It is now common to observe treatment-resistant tumours with neuroendocrine features upon metastatic biopsy, and the prevailing consensus is that epithelial plasticity enables tumour adaptation in response to AR-targeted therapies [7, 9, 118, 126, 131–134]. This evidence supports the notion that tNEPC incidence through NEtD will increase as new powerful ADTs enter the clinic. There is an urgency for therapeutic strategies and clinical biomarkers defining NEtD/NEPC. Currently, the only option for patients is the short-lived effects of platinum-based chemotherapy. Optimism is on the rise, as there is an AURKA inhibitor (MLN8237) in a Phase 2 clinical trial (NCT01799278), combinatorial approaches using AURKA with PARP inhibitors under investigation [135], indirect methods that resensitize the tumour to Enzalutamide [136] or platinum-based chemotherapy [137] (Phase 2 clinical trial NCT02489903 with a Phase 3 clinical trial being planned), a SSTR4/5 analogue (Pasireotide/SOM230) in four independent clinical trials at various Phases (NCT01646684, NCT01313559, NCT01468532, and NCT01794793) with one already reporting promising clinical efficacy [138], and increased study of NEPC/NEtD in general [134, 139, 140].

In this study, we characterized the unexplored global lncRNA landscape during NEtD to provide insights into the NEPC non-coding milieu of this lethal and treatment-induced process. This required the implementation of a sequence analysis pipeline with increased sensitivity towards lower expressed transcripts, characteristic of lncRNAs. The pipeline was able to detect 37,749 lncRNA transcripts (subclassified as either lincRNA, antisense, or pseudogene) and quantify them in the two clinical cohorts (VPC and WCM). The novelty of this study lies in the use of patient samples integrated with the NEtD PDX model to detect clinically relevant lncRNAs involved in the NEtD/phase transition process. In this study, we identified 742 lncRNAs associated with NEtD and identified a robust 122 NEPC lncRNA patient signature capable of classifying NEPC from AD patient samples. The motif analysis identified significantly enriched TFBS that were unique to NEtD Classes I Deactivated (TP53 and BRN1), II Activated (ELF5, SPIC, and HOXD1), III Persistent (SPDEF and HOX), IV Transient (TP53, HSF1, HSF2, and FOXA1), and III Persistent combined with IV Transient (TWIST1). Through similar analysis, we also identified common TFBS (CCNB2, E2F, ETS, REST, PAX5, PAX9, and STAF) enriched across all of the NEtD lncRNAs. From among the 100 top-ranking lncRNA, we observe that a subset have strong clinical associations with metastatic PCa patients after receiving ADT. In previous lncRNA studies in cancer, several have been linked to malignant transformation with key roles affecting various aspects of cellular homeostasis, including proliferation, survival, migration, and genomic instability [141]. Similarly, lncRNAs identified in this study, including SSTR5-AS1 and LINC00514 with their association with poor outcome, FENDRR for its association with rapid metastasis, and H19 and LINC00617 for their concordantly high expression across both of the discovery cohorts, could be the missing links in the mechanisms causing NEtD. These five represent the top candidates discovered in this study due to this evidence but also for their characterization in other cancer types.

FENDRR is a top deregulated lncRNA in NEtD Class IV Transient and may have a role in the NEtD process. It is implicated in a lethal lung development disorder [142], lung cancer [143], within a mutational hotspot that is copy number lost in PCa [144], and can bind to PRC2 [145, 146]. PRC2 plays a significant role in tumour progression through binding of HOTAIR (a very well-studied lncRNA). Together, HOTAIR and PRC2 are involved in the control of chromatin structure and associated gene activity [147]. FENDRR may be involved in tumorigenesis like HOTAIR due to its known interaction with PRC2. A recent study showed down-regulation of FENDRR is associated with poor prognosis in gastric cancer and regulates cancer cell metastasis through fibronectin [71]. Functionally, this could be occurring in NEPC as well due to FENDRR's transient expression in the NEtD model and its association to rapid metastasis in ADT-treated PCa patients from the GRID (MCI) cohort. Another putative function of this transcript is through upregulating FOXF1, which is a protein-coding gene and the sense form for the antisense transcript FENDRR. Antisense transcripts are known to regulate their sense forms (positively or negatively). Using TANRIC, an interactive resource for the exploration of lncRNAs in large patient cohorts within 20 TCGA cancer types [148], we see that FENDRR expression is positively correlated to FOXF1 in 16 of 20 cancer types (*P* < 3.71 × 10^−9^; Supplementary Table S29). In fact, FOXF1 deletion has been seen to significantly reduce FENDRR in endothelial cells [149]. FOXF1 is also a target gene of p53 and is seen to regulate cancer cell migration and invasiveness [150]. Together these transcripts may play a transient coordinated role in NEtD through PRC2 or fibronectin.

LINC00514 is amongst the highest expressed lncRNAs in NEtD Class III Persistent. It has not been characterized. It is predicted to bind to TADA3 (Supplementary Fig. S14), potentially causing a reduction of its activity. This is intriguing because TADA3 is involved in the stabilization and activation of p53 [151, 152], and this putative interaction (LINC00514: TADA3) could be an alternative mechanism for loss of p53 activity, already known to be frequently lost in NEPC [15]. H19 and LINC00617 were two of the four highest (>10-fold) NEPC-expressed lncRNAs in this study and fortunately (unlike most lncRNAs) have both been thoroughly characterized functionally. LINC00617 is highly conserved across vertebrate genomes and is required for maintenance of pluripotency and neural differentiation in embryonic stem cells [153]. It controls this lineage commitment through RNA-binding proteins PTBP1, hnRNP-K, and Nucleolin. These RNA-binding protein complexes have been detected at promoters of NANOG, SOX2 (promoter of lineage plasticity in NEPC [28]), and FGF4 [153]. H19 has also been identified in neural differentiation of pluripotent stem cells [154] but with unknown mechanisms. With such an elevated level of expression in the clinical cohorts (∼30- to 40-fold and ∼20- to 30-fold in VPC/WCM for LINC00617 and H19, respectively), these lncRNA could be responsible for maintaining the neuronal component of NEPC through epigenetic regulation.

SSTR5-AS1 is the highest expressed lncRNA in the NEPC clinical samples when requiring expression concordance in VPC and WCM cohorts. It is an antisense transcript of SSTR5, which is a member of the superfamily of somatostatin receptors. Somatostatins are peptide hormones that regulate diverse cellular functions such as neurotransmission, cell proliferation, and endocrine signalling, as well as inhibiting the release of many hormones and other secretory proteins. The SSTR family (1–5) are markers for neuroendocrine tumours of the lung [155], with SSTR1 and SSTR5 the most dominant forms of SSTR in neuroendocrine tumours in general [54]. Interestingly, exploration within TANRIC showed a strong positive correlation in expression with SSTR5 to SSTR5-AS1 in 14 of 20 cancer types (*P* < 2.18 × 10^−15^; Supplementary Table S29). Furthermore, SSTR5 mRNA is detectable in the blood of neuroendocrine tumours of the lung [156] and could be a valuable non-invasive diagnostic marker for NEPC. In fact, clinicians utilize this biological feature in other neuroendocrine tumours (NETs) using Octreoscans to determine tumour stage and/or identification of sites of metastasis. Octreoscans, when compared with positron emission tomography (PET) scans (another commonly used approach for this), appear more sensitive in the detection of well-differentiated NETs [157]. In addition to this, therapeutically, somatostatin analogues are emerging as a promising treatment option for inoperable or metastatic NETs [158]. However, specifically in NEPC, targeting SSTR5 and/or SSTR5-AS1 for diagnostic or therapeutic purposes is in its infancy. Interestingly, SSTR5 (C terminal) is required for Rb induction and G1 cell cycle arrest [159], resulting in anti-proliferative effects. However, without Rb (known to be lost in NEPC), this function is negated. Alternatively, the interaction evidence for SSTR5-AS1 and KDM4B (Supplementary Fig. S13) provides another strong connection to NEPC biology. KDM4B is a histone demethylase and a key molecule in AR signaling and turnover [160]. In NEPC with the absence of the AR, KDM4B could interact with N-Myc instead, where it has been shown to regulate and epigenetically activate this oncogene in neuroblastoma [161]. N-Myc has been seen to drive the progression of NEPC [5, 26, 27] and recently through EZH2-mediated transcription [27]. However, another mechanism of activation could be facilitated through SSTR5-AS1 regulation. However, both of these putative functions (SSTR5-AS1: SSTR5 or SSTR5-AS1: KDM4B: N-Myc) require thorough *invitro* and *invivo* exploration to ascertain their validity.

Although multiple layers of genetic and epigenetic deregulation likely cooperate to facilitate NEtD, understanding the non-coding contribution to this multifarious process is necessary to design effective novel therapeutics. Using the five independent patient cohorts and our proven NEtD PDX LTL331 model, lncRNAs such as FENDRR, LINC00514, LINC00617, H19, SSTR5-AS1, and others identified in this study may provide more in-depth insights into NEtD and NEPC. Research identifying the relationship of these lncRNAs to other known drivers, oncogenes, and Activated pathways in NEtD is now required. This study is the first to report the lncRNA landscape of NEtD, a robust NEPC lncRNA expression clinical classifier, and provides numerous candidate biomarkers and therapeutic targets.

## Methods

### PDXs

Animal ethics, care, experiments, xenograft generation, and all protocols were carried out in accordance with the guidelines of the Canadian Council of Animal care as previously described [7]. Specific xenograft models used in this study have been previously published (protein-coding transcriptomes) by Akamatsu et al. [24] and Mo et al. [162]. In brief, six LTL331, two LTL313, and two LTL418 PDXs were raised in NOD-SCID mice (NOD.CB17-Prkdcscid/J) at the Living Tumor Laboratory [163]. Xenograft tissue was harvested after fixed lengths of time post host castration, tissue was measured, fixed for histopathological analysis, and processed for RNA analysis.

### Clinical datasets

We used five clinical cohorts from 1) WCM [5]; 2) GenomeDx Biosciences (GX) Inc. (MCI and MCII); 3) JHSM; and 4) VPC, cumulatively totalling 927 samples. For the VPC, 80 specimens were obtained from patients undergoing RP and snap frozen following a protocol approved by the Clinical Research Ethics Board of the University of British Columbia, the BC Cancer Agency, and Vancouver General Hospital pathology (depending on the sample source). All patients signed a formal consent form approved by the ethics board. A subset of the GX Decipher GRID database of clinical specimens was selected, totalling 777 patient PCa expression profiles (all from formalin-fixed parafin embedded tissue) and were obtained from two RP Mayo Clinic cohorts that have been previously described (MCI [116] and MCII [117]). JHSM samples, totalling 33 samples, were retrieved from surgical pathology and consultation files of Johns Hopkins Hospital (John Hopkins Registry) from 1999 to 2013, as previously described [164]. The 33 samples were annotated as 6 morphologically diagnosed pure SCPC samples, 12 high-risk (Gleason 9–10) AD, 10 SCPC (SC-mixed), and 5 AD (AD-mixed) from mixed histology tumours containing separate adenocarcinoma and small cell components. For this cohort, samples were dicotimized into either AD (AD and AD-mixed samples) or NEPC (SCPC and SCPC-mixed sampels) for the purposes of validating the 122 NEPC lncRNA patient signature. We also explored an externally processed cohort comprising 114 metastatic CRPC specimens, of which 44 were NEPC [32] and used in this study. Referred to in the text as the extNEPC cohort, we accessed and visualized this data through cBioPortal [165, 166] Version 1.9.0 [167]. OncoPrint schematics were generated for displaying multiple genomic alterations by heatmap for the lncRNAs. The extNEPC study samples were classified using a pathologic classification system [6] that included five catagories: “A,” usual prostate adenocarcinoma without neuroendocrine differentiation; “B,” usual prostate adenocarcinoma with neuroendocrine differentiation >20%; “C,” small-cell carcinoma; “D,” large-cell neuroendocrine carcinoma; and “E,”, mixed small-cell carcinoma–adenocarcinoma.

### Material collection and processing (VPC Cohort)

Hematoxylin and eosin (H&E) stained, formalin-fixed paraffin-embedded, and fresh frozen sections were reviewed by a pathologist to identify blocks with highest tumour content. For each frozen block used, a 5-µmslide was first taken for H&E staining; then 4 × 100-µmsections were taken for DNA and RNA isolation before a second 5-µm slide was taken for H&E staining. Each H&E slide was required to have tumour content >50% for a tumour to proceed for sequencing. RNA from 100-µmsections of snap frozen tissue was isolated using the mirVana Isolation Kit from Ambion (AM 1560). RNA sequencing was performed on Illumina HiSeq 2000 at BC Cancer Agency Michael Smith Genome Sciences Centre according to standard protocols.

### Material collection and processing (GRID and JHSM)

For GRID (MCI and MCII) and JHSM cohorts, specimen selection, RNA extraction, and microarray hybridization were performed in a Clinical Laboratory Improvement Amendments-certified laboratory facility (GenomeDx Biosciences, San Diego, CA, USA) as described previously [116, 117]. Total RNA extraction, purification, RNA amplification, and labelling were done using the Ovation WTA FFPE system (NuGen, San Carlos, CA, USA). RNA was hybridized to Human Exon 1.0 ST GeneChips (Affymetrix, Santa Clara, CA, USA). After microarray profiling, quality control was preformed using the Affymetrix Power Tools package, and probe set normalization was performed using the Single Channel Array Normalization algorithm [168].

### Quantitative Real-Time Polymerase Chain Reaction (qRT-PCR)

Primers were designed using Primer3 and checked with in silico PCR in UCSC Genome Browser (s Supplementary Table S26 for forward and reverse primer sequences). Housekeeping genes PSMB4, REEP5, and SNRPD3 were selected on the basis of high, consistent expression levels across many cell and tissue types and were used in the MiTranscriptome lncRNA study [169, 170]. Two lncRNAs from each NEtD class and three NEPC lncRNAs from among the top candidates (Supplementary Fig. S12 and Supplementary Table S26) were selected (n = 11) for qRT-PCR validation. The cDNA from the PDX LTL331 models three time points (AD, postTX, and NEPC) were used to validate the NEtD lncRNAs and a subset of the VPC clinical samples for the NEPC lncRNAs. With the rarity of clinical NEPC samples, tumour tissue and subsequent RNA were extremely limited. Due to this, only three NEPC (V73, V90, and V91) and one AD (V60) clinical sample were included in this validation. For each lncRNA and sample tested, the following experimental protocol was carried out: 1 μg of total RNA for each sample was diluted to 18 μl with water and 1 μl of random hexamers (50μM; Thermo Fisher). The mixture was heated to 65C for 5 minutes and chilled. Afterwards, 5 μl of 5x reverse transcriptase buffer, 1 μl of 10 mM dNTPs, and 1 μl of Superscript II reverse transcriptase (Thermo Fisher) were added. Each sample was then incubated at 42C for 1 hour and then at 70C for 15 minutes. Prior to use in qRT-PCR, products were diluted 10-fold with water. FastStart Essential Green Master kit from Roche (Catalogue #06 402 712 001) was used as described from their protocol for qRT-PCR reactions. In brief, 2 μl of water, 3 μl of a mixture of forward and reverse primers (each at a concentration of 10 μM),and 10 μl of the Roche Master Mix were aliquoted into each well of a 96-well plate. A mixture of 4 μl of water plus 1 μl of the diluted cDNA was then added to the appropriate wells. Expression was then quantified (as measured by Ct) through the Roche Light Cycler 96 machine. Each lncRNA/sample pair was quantified with technical replicates in triplicate. Average and standard deviation of Ct were calculated across these triplicates, and ΔCt calculated relative to house keeper gene PSMB4 (most consistent and highly expressed gene vs REEP5 and SNRPD3). Delta ΔCts were calculated relative to control samples, and fold changes were plotted using Prisms GraphPad software (Supplemental Table 12).

### RNA sequence analysis pipeline

We implemented an lncRNA sequence analysis pipeline that includes algorithms catered to the detection of known and novel transcripts (Supplementary Figs S1 and S2). Implemented in-house, this pipeline is modified and extended from the tuxedo suite of sequence analysis algorithms [45]. Once received from the sequencing centre in bam format, all sequenced model systems and patient samples were de-aligned into raw fastq format (including flagged reads) using bam2fastq and put through the following pipeline. To ensure high-quality sequence reads, libraries were trimmed using a Sickle, a windowed-adaptive approach [171]. For each read pair processed together, the algorithm determines the most optimal inner read sequence by trimming both 3’ and 5’ prime ends based on quality and length thresholds (for full description, see [172]). Bases with a quality score of <99.0% base call accuracy (corresponding to a Phred quality score of 20) were removed. Reads less than approximately two-thirds read length (30 nt in WCM and 60 nt in VPC) post-trimming were discarded. Highly repetitive sequences (>2% of library) were also discarded post-trimming using the cutadapt tool. All quality control metrics were generated and quanitified (pre- and post-trimming) using the FASTX-Toolkit and the FastQC Windows software. Reads were aligned to the Hg19 human genome build using an unspliced aligner for handling exonic reads (Bowtie–v2.2.3) in conjunction with a spliced aligner to handle reads spanning exon-exon junctions (Tophat 2.0.12). Transcriptome reconstruction using Ensembl GRCh37.75 gene tracks for each library was performed using a quasi *de novo* (genome-guided) approach (Cufflinks v2.2.1), where reads were assembled and abundances estimated using an overlap graph producing a minimal spanning network of transcripts. This version of Ensembl contained 38 transcript classes grouped by 4 core biotypes. At this stage, transcripts were also multi-read and fragment bias corrected. Transcripts with highly abundant expression were masked (e.g., rRNAs) from downstream steps to increase transcript quantification accuracy. Sample transcriptomes, the reference genome, and the transcript annotation were then meta-assembled (Cuffmerge) to produce a single annotation transcriptome model. Based on this model, transcript quantification (Cuffquant) and normalization (Cuffnorm). Geometric and FPKM normalization performed independently (Cuffnorm) corrected for uneven library sequencing depths between samples and variable transcript lengths within samples. Transcript expression displaying computational artifacts (expression values <0.1 known to occur with Cufflinks) were converted to zero values. This generated transcript expression where only lncRNAs (Ensembl and ENCODE-based) were extracted and used for all downstream analysis. All algorithms denoted in brackets are referenced and described in the Trapnell et al. Nature protocol [45]. Each cohort (VPC and WCM) was processed independently by this pipeline, and then transcriptome annotations were merged. This was accomplished using Ensembl transcript IDs combined with transcript lengths to produce unique transcript identifiers for each lncRNA across cohorts.

### RNA-RNA interaction analysis

A genome-wide analysis for SSTR5-AS1 and LINC00514 lncRNA interactions was performed using a multistep systemic approach [119]. This tool is available publicly within an online database [173] hosted by the Computational Biological Research Center at the National Institute of Advanced Industrial Science and Technology in Japan. The interaction search space included all hg19 annotated lncRNA and mRNA transcripts. This generated top-ranking interaction partners (n = 100), based on local interaction minimum free energy (Supplementary Tables S27–S28). R-chie [174, 175] was used to visualize the top-ranking predictions KDM4B and TADA3 for SSTR5-AS1 and LINC00514, respectively, using the double structure feature (Supplementary Figs S13 and S14). All bases that were not within the interaction site were predicted to form RNA secondary structure by RNAfold [176–178] selecting to enforce constrained pairing patterns for the interacting bases. Minimum free-energy structures were predicted by RNAfold on the 300nt sequences upstream and downstream of the interaction site.

### TFBS identification and enrichment analysis

All TFBS analysis was performed using Genomatix software, databases, and algorithms [179]. Three types of TF analysis were carried out in this study: (1) single lncRNA motif characterization, (2) multiple lncRNA analysis for select TFs, and (3) multiple lncRNA enrichment analysis. Prior to any of the above, lncRNA transcript(s) were submitted to the Gene2Promoter algorithm for retrieval of promoter sequences. Databases used with this algorithm included ElDorado 12–2013 and NCBI build 37 (for multiple lncRNA analysis where genomic background needed to match sequencing data) or the most recent databases ElDorado 12–2016 and GRCh38 (for single lncRNA analysis where genomic background was not relevant). Transcripts with alternative isoforms were required to have gold level (experimentally verified 5' complete transcript), silver level (transcript with 5' end confirmed by PromoterInspector prediction), or bronze level (annotated transcript, no confirmation for 5' completeness) quality for their alternative isoforms. (1) Single lncRNA motif characterization was performed using the MatInspector algorithm [97–99] with parameters “core similarity” (degree of similarity for highest conserved bases of motif) set to 1 and “matrix similarity” (degree of similarity between motif and query sequence) set to optimized as recommended by Genomatix and as described in MatInspector referenced papers above. MatInspector uses the best in field MatBase database for TFBS motif/matrix annotation, where Matrix Family Library Version 10.0 was used. (2) Multiple lncRNA analysis for select TFs was performed using MatInspector and select TF motifs (“matrix”) applied accordingly. All matrix annotation, descriptions, and matrix family definitions are listed in Supplementary Table S25. Select TF matrices (BRN2, STAT3, NKX3, NMYC, SOX2, and SOX11) and select TF matrix families (GREF [includes the androgen receptor and the closely related glucocorticoid, mineralocorticoid, and progesterone receptors], NRSF [REST], SOX, HOX, STEM, E2FF, ETSF, and ETVI1) motifs included in this study are described in Supplementary Table S25. Core and matrix similarities were again set to 1 and optimized, respectively. (3) Multiple lncRNA enrichment analysis was performed using the Overrepresented TFBS algorithm. Enrichment of matrix/matrix family was determined by Genomatix calculated z-scores (>2 or <-2), which is based on the distance from the population mean (genome or promoter sequence background) in units of the population standard deviation for query sequence/promoter. Genomatix calculates z-scores with a continuity correction using the formula z = (x-E-0.5)/S, where x is the number of found matches in the input data, E is the expected value, and S is the standard deviation. This formula is also described in the oPOSSUM algorithm [180]. A z-score < -2 or >2 can be considered statistically significant and corresponds to a *P*-value of approximately 0.05.

### Microarray to sequencing platform lift over/mapping

Affymetrix Human Exon 1.0 ST GeneChip probes were mapped to hg19 coordinates using SMALT v0.76 [181]. Probe set genomic regions (PSRs) were redefined accordingly. Exons within each lncRNA from sequencing cohorts (VPC and WCM) were integrated with PSRs to build an overlap table to determine absence/presence of lncRNA transcripts on the affymetrix microarray. R function iRanges v2.9.18 was used with method findOverlap to build the described table above. Microarray PSRs were required to be entirely within sequenced exon genome regions; otherwise they were excluded. Applying this methodology, 106 of 122 NEPC lncRNAs (87%) and 81 of 100 NEtD lncRNAs (81%) mapped to microarray PSRs for clinicopathological analysis on GRID cohorts MCI and MCII.

### Statistical analysis

For all cohorts, the programming language R v3.0 was used for statistical analysis. For VPC and WCM cohorts, unsupervised hierarchical clustering was performed with the h.clust package with Pearson correlation for distance and average linkage used. Only transcripts within the top fifth percentile based on their standard deviations were selected. The clustering and heatmaps generated were built using the heatmap.2 function. Similar clustering analysis was performed for GRID cohorts except with Euclidian distance, the ward method for linkage, and the use of the heatmap.3 function due to its advanced row/column labelling features. For all cohorts before clustering, normalized log2 expression values were standardized/scaled using a z-score that ranged from -2 to 2. For principal component analysis, the R package prcomp was used to calculate variance among transcript and sample subsets for the calculation of transcript weights and principle components. The top three components were used for visual inspection. For all clinical group-wise comparisons, a standard Student's *t-*test was applied to identify significantly differentially expressed transcripts between groups/phenotypes. Significance thresholds were implemented by enforcing a strict *P*-value cut-off of <0.05. Multiple test correction was applied to *P*-values using the Bonferroni and Hochberg method to *mathematically* minimize false discovery rate (FDR) and with a cut-off of *P*-value < 0.05. See Supplementary Table S4 for these results. To *biologically* minimize FDR, *mathematical* FDR correction was removed and instead followed the filter-down workflows in Figs 3A–C and Fig. 1C. Despite *mathematical* FDR being removed, statistical significance of *P*-value < 0.05 was still maintained during the filter-down approach using the described method in each step. See Supplementary Table S5 for these results. For ROC curves and AUC calculations, the R package “pROC” was used. Kaplan-Meier analysis was performed for determining survival outcome using the R package “survfit” with transcripts displaying below background (<0.1) expression being removed from this analysis.

### Transcript ranking

NEtD lncRNA transcripts were ranked based on fold changes observed in the clinical group-wise comparisons. For NEtD Class I Deactivated, the three group-wise comparison fold changes were used (NEPC vs AD, CRPC vs AD, and NHT vs AD), where the minimum fold change observed between the three comparisons was selected and then ranked in decreasing order. For Class II Activated and Class III Persistent transcripts, NEPC vs AD fold changes were calculated and ranked in increasing order for both VPC and WCM cohorts, where the maximum fold change between VPC and WCM was selected. For Class IV Transient transcripts, absolute fold changes for AD vs NHT and NHT vs NEPC were calculated and ranked in increasing order with the maximum fold change from either group selected. Similar ranking was performed for NEPC lncRNA transcripts, which were ordered by increasing (up-regulated transcripts in NEPC vs. AD) or decreasing (down-regulated transcripts in NEPC vs. AD) order to determine the highest- and lowest-expressed transcripts in NEPC vs AD, respectively. Concordantly expressed transcripts were required between VPC and WCM cohorts. The top 20 lncRNAs (based on fold changes from clinical samples defined above) were taken from each group. This produced 20 × 5 group (n = 100) isoforms representing 76 unique lncRNA transcripts. These represent the top NEtD/NEPC lncRNA candidates from this study (Supplementary Table S26). No pseudogenes were included in these rankings.

## Availability of supporting data

A subset of the sequenced samples (n = 70) used in this study was from previous studies with all raw sequencing data reanalyzed here using the described pipeline above. These 70 samples have been previously submitted to the European Nucleotide Archive (ENA) or NCBIs Gene Expression Omnibus. This includes the 6 NEPC PDX model samples [24] (ENA accession number PRJEB9660 and GEO accession number GSE59986), 2 CRPC PDX model samples [162] (ENA accession number PRJEB19256), 4 NEPC (VPC) samples [31, 56], 23 AD (VPC) samples [56] (ENA accession number PRJEB6530), 30 AD (WCM) samples [5], and 7 NEPC (WCM) samples [5]. The remaining unpublished sequenced samples (n = 55) have been submitted to the ENA under accession number PRJEB21092. Please see Supplementary Table S1 for a summary of sequencing and clinical information on these 125 samples. All microarray samples from GX cohorts, including 545 AD (MCI [116]) samples and 232 AD (MCII [117]) samples, are accessible through Gene Expression Omnibus accession numbers GSE46691 and GSE62116, respectively. Additional supporting data and custom code from the sequencing pipeline described above are also available from the *GigaScience* GigaDB database [182].

## Supplementary Material

GIGA-D-17-00096_Original_Submission.pdf

GIGA-D-17-00096_Revision_1.pdf

GIGA-D-17-00096_Revision_2.pdf

GIGA-D-17-00096_Revision_3.pdf

Response_to_Reviewer_Comments_Original_Submission.pdf

Response_to_Reviewer_Comments_Revision_1.pdf

Response_to_Reviewer_Comments_Revision_2.pdf

Reviewer_1_Report_(Original_Submission) -- Etienne Dardenne 6/19/2017 Reviewed

Reviewer_1_Report_(Revision_1) -- Etienne Dardenne 1/22/2018 Reviewed

Reviewer_2_Report_(Original_Submission) -- Ha Dang 6/30/2017 Reviewed

Reviewer_2_Report_(Revision_1).pdf

Reviewer_2_Report_(Revision_2) -- Ha Dang 1/28/2018 Reviewed

Supplement Files

## Additional file

**Table S1:** Discovery cohorts clinical and sequencing information.

**Table S2:** Differentially expressed AR- and AR+ CRPC lncRNA.

**Table S3:** NEPC lncRNA expression signature labels and order for Fig. 2.

**Table S4:** Clinical cohort group-wise comparisons.

**Table S5:** NEtD and NEPC lncRNA transcript annotation

**Table S6:**TFBS-Enrichment by Matrix for NEtD Class I

**Table S7:**TFBS-Enrichment by Matrix for NEtD Class II

**Table S8:** TFBS-Enrichment by Matrix for NEtD Class III

**Table S9:**TFBS-Enrichment by Matrix for NEtD Class IV

**Table S10:**TFBS-Enrichment by Matrix for NEtD Class III-IV

**Table S11:**TFBS-Enrichment by Matrix for all NEtD lncRNAs

**Table S12:**TFBS-Enrichment by Matrix Family for NEtD Class I

**Table S13:**TFBS-Enrichment by Matrix Family for NEtD Class II

**Table S14:**TFBS-Enrichment by Matrix Family for NEtD Class III

**Table S15:**TFBS-Enrichment by Matrix Family for NEtD Class IV

**Table S16:**TFBS-Enrichment by Matrix Family for NEtD Class III-IV

**Table S17:**TFBS-Enrichment by Matrix Family for all NEtD lncRNAs

**Table S18**: TFBS Overlap Table by Matrix

**Table S19:**TFBS Overlap Table by Matrix Family

**Table S20:**Unique and common TFBS in NEtD lncRNA

**Table S21:**Select TF Identification for NEtD Class I

**Table S22:**Select TF Identification for NEtD Class II-III

**Table S23:**Select TF Identification for NEtD Class III-IV

**Table S24:**Select TF Identification for all NEtD lncRNAs

**Table S25:** Genomatix Matrix and Matrix Family definitions

**Table S26:** Top-ranking NEPC and NEtD lncRNAs.

**Table S27:** SSTR5-AS1-predicted RNA (mRNA or lncRNA) interactions with associated binding energies, predicted transcript Ensembl ID, name, interaction position, and ranking.

**Table S28:** LINC00514-predicted RNA (mRNA or lncRNA) interactions with associated binding energies, predicted transcript Ensembl ID, name, interaction position, and ranking.

**Table S29:** TANRIC results for lncRNAs FENDRR and SSTR5-AS1. Spearman rank correlation for protein coding genes that are the sense forms to the above antisense transcripts. Numbers in brackets denote *P*-values. NSC, no significant correlation; NA, mRNA data was not available for this tumour type, therefor the analysis was not applicable.

**Figure S1:** The next-generation sequence analysis pipeline implemented for the detection and quantification of lncRNAs in this study. A) The nine-step lncRNA next-generation sequencing analysis pipeline with core algorithms (Bowtie2, Tophat2, Cufflinks2, Cuffmerge, Cuffquant, and Cuffnorm) implemented from the Tuxedo suite of analysis tools. Sequencing quality control metrics before and after trimming of data for sample V60 is outlined in B–F. This includes pre-trimming (A) phred quality scores and (B) percentage of each base type across read library at each base pair position. After quality control corrections were applied, V60 read library had acceptable (D) Phred quality scores (∼30 Phred Score) and (E) expected base type percentages (∼25%) for T, C, A, and G. F) All over-represented sequences that were >2% of library were removed from the V60 read library. See Methods for complete listing and version numbers for all algorithms and tools used in the sequence analysis pipeline.

**Figure S2:** Average Phred quality scores for all VPC and WCM samples before and after quality control corrections were applied.

**Figure S3:** Unsupervised hierarchal clustering (A–D) and principle component analysis (E–H) on the four major Ensembl transcript classes detected within the VPC cohort. Samples are labelled as adenocarcinomas (blue) and neuroendocrine tumours (gold).

**Figure S4:** Unsupervised hierarchal clustering (A–D) and principle component analysis (E–H) on the four major Ensembl transcript classes detected within the WCM cohort. Samples are labelled as adenocarcinomas (blue) and neuroendocrine tumours (gold).

**Figure S5:** Detected and differentially expressed lncRNAs among (A) AR^−/+^ CRPC xenograft models (B) and matched clinical samples. P, patient; X, xenograft.

**Figure S6:** NEPC and NEtD lncRNA Oncoprint Plot in the extNEPC Cohort – LEGEND. Clinical, transcriptome, and genomic annotation for samples plotted in Supplementary Figs S7–S11. All annotations were generated from cBioportal with the exception of the NEPC molecular subtype, which was assigned from this study. Transcripts denoted with 1 in superscript within Supplementary Figs S8–S11 are lncRNAs that overlap a NEPC lncRNA with a NEtD lncRNA. For example, H19 appears in Supplementary Fig. S8 (NEPC lncRNA) and supplementary 9 (NEtD lncRNA Class II).

**Figure S7:** NEPC and NEtD lncRNA Oncoprint Plot in the extNEPC Cohort - Known NEPC genes and TFs. Select NEPC oncogenes, tumour suppressor, and transcription factors that have been reported previously or within this study for transcriptomic/genomic context with Supplementary Figs S8–S11. Please see Supplementary Fig. S6 for figure legend.

**Figure S8:** NEPC and NEtD lncRNA Oncoprint Plot in the extNEPC Cohort - NEPC lncRNA. Transcripts from the NEPC lncRNA expression signature that are up-regulated (74 of 122), testable (58 of 74), and altered (25 of 58) in the extNEPC cohort. Please see Supplementary Fig. S6 for figure legend.

**Figure S9:** NEPC and NEtD lncRNA Oncoprint Plot in the extNEPC Cohort - NEtD lncRNA Class II. Transcripts from NEtD lncRNA Class II (222), testable (128 of 222), and altered (26 of 128) in the extNEPC cohort. Please see Supplementary Fig. S6 for figure legend.

**Figure S10:** NEPC and NEtD lncRNA Oncoprint Plot in the extNEPC Cohort - NEtD lncRNA Class III. Transcripts from NEtD lncRNA Class III (84), testable (79 of 84), and altered (29 of 84) in the extNEPC cohort. Please see Supplementary Fig. S6 for figure legend.

**Figure S11:** NEPC and NEtD lncRNA Oncoprint Plot in the extNEPC Cohort - NEtD lncRNA Class IV. Transcripts from NEtD lncRNA Class IV (45), testable (36 of 45), and altered (11 of 31) in the extNEPC cohort. Please see Supplementary Fig. S6 for figure legend.

**Figure S12:** Quantitative Real-Time Polymerase Chain Reactionon select NEPC and NEtD lncRNAs.

**Figure S13:** Hypothetical RNA-RNA folding structure for exon 4 of SSTR5-AS1 (top) and the 3’UTR of KDM4B (bottom). Predicted base pair binding (green arcs) along the sequence (black arrow) are displayed, included predicted interaction site (orange bars).

**Figure S14:** Hypothetical RNA-RNA folding structure for exon 4 of LINC00514 (top) and the 3’UTR of TADA3 (bottom). Predicted base pair binding (green arcs) along the sequence (black arrow) are displayed, included predicted interaction site (orange bars).
