# Supplementary material for: The long noncoding RNA landscape of neuroendocrine prostate cancer and its clinical implications
Source: Gigascience. 2018 May 10;7(6):giy050. doi: 10.1093/gigascience/giy050 (PMC6007253; doi:10.1093/gigascience/giy050)
Supplement: GIGA-D-17-00096_Original_Submission.pdf [file giy050_giga-d-17-00096_original_submission.pdf]

GigaScience

# The Long Noncoding RNA Landscape of Neuroendocrine Prostate Cancer and its Clinical Implications

--Manuscript Draft--

| | |
|---|---|
| **Manuscript Number:** | GIGA-D-17-00096 |
| **Full Title:** | The Long Noncoding RNA Landscape of Neuroendocrine Prostate Cancer and its Clinical Implications |
| **Article Type:** | Research |
| **Funding Information:** | Mitacs (CA) Accelerate PhD Fellowship Program (IT04310) — Mr Varune Rohan Ramnarine<br>Terry Fox Foundation (201012TFF) — Dr Colin C Collins<br>Prostate Cancer Team Grant (T2013-01) — Dr Colin C Collins |


**Abstract:**

BACKGROUND
Neuroendocrine prostate cancer (NEPC) is an aggressive variant of late stage prostate cancer that most commonly arises through neuroendocrine transdifferentiation (NEtD). Treatment options are limited, ineffective, and in most patients, results in death in less than a year. We recently developed a first-in-field patient-derived xenograft (PDX) model of NEtD. Longitudinal deep transcriptome profiling of this model enabled monitoring of dynamic transcriptional changes during the NEtD process and in the context of selective treatment pressure. In the current study, we focused on long non-coding RNAs (lncRNA), which have been implicated in tumorigenesis and cancer progression through gene regulation, and whose roles in NEtD remain largely unexplored.

RESULTS
We implemented a next-generation sequence analysis pipeline that can detect transcripts at low expression levels and built a genome-wide catalogue (n=50,000) of lncRNAs. We applied this pipeline to 927 clinical samples and our high fidelity NEtD model and identified 4,720 lncRNAs in NEPC. Among these were 216 lncRNAs capable of robustly classifying NEPC from prostate adenocarcinoma (AD) patient tumours. Another 2,553 were associated with the NEtD process, identified through five distinct patterns of expression in our PDX model and clinical samples. We interrogated our top deregulated candidates (n=106) in primary tumours obtained from radical prostatectomies all with long-term follow-up (median 18 years) and discovered significant clinicopathological associations. Specifically, we identified 26 that are associated with rapid metastasis following androgen deprivation therapy (ADT). Two of these lncRNAs (SSTR5-AS1 and LINC00514) significantly stratified patients undergoing ADT based on patient outcome.

DISCUSSION
A comprehensive characterization of the dynamic expression of lncRNAs during the NEtD process has not been performed to date. Temporal dissection of our PDX-based NEtD model has provided insights into lncRNAs linked to NEtD. Furthermore, select NEtD lncRNAs provide strong evidence for an association with metastasis and patients receiving ADT, which are clinical features of NEPC tumours. Candidate protein coding transcripts (KDM4B, TADA3, FOXF1, and SSTR5) have NEPC associations and are identified here as being regulated by NEtD lncRNA. In vivo and in vitro functionalization is required to validate these findings and to identify the mechanisms of action and/or interaction of these lncRNAs.

CONCLUSION
We present here for the first time a genome-wide catalog of NEtD lncRNAs that characterize the transdifferentiation process and a NEPC lncRNA expression signature. To accomplish this, we carried out the largest integrative study applying a PDX NEtD model to clinical samples performed to date. These NEtD and NEPC lncRNA represent strong candidates for clinical biomarkers and therapeutic targets,

| | |
|---|---|
| | warranting further investigation in these respective areas. |
| **Corresponding Author:** | Varune Rohan Ramnarine<br><br>CANADA |
| **Corresponding Author Secondary Information:** | |
| **Corresponding Author's Institution:** | |
| **Corresponding Author's Secondary Institution:** | |
| **First Author:** | Varune Rohan Ramnarine |
| **First Author Secondary Information:** | |
| **Order of Authors:** | Varune Rohan Ramnarine |
| | Mohammed Alshalalfa |
| | Fan Mo |
| | Dong Lin |
| | Noushin Nabavi |
| | Nicholas Erho |
| | Mandeep Takhar |
| | Hussam Al-Deen Ashab |
| | Sonal Brahmbhatt |
| | R. Jefferey Karnes |
| | Mark A Rubin |
| | Himisha Beltran |
| | Harrison Tsai |
| | Tamara L Lotan |
| | Stanislav V Volik |
| | Alexander W Wyatt |
| | Elai Davicioni |
| | Yuzhuo Wang |
| | Colin C Collins |
| **Order of Authors Secondary Information:** | |
| **Opposed Reviewers:** | |
| **Additional Information:** | |
| **Question** | **Response** |
| Are you submitting this manuscript to a special series or article collection? | No |
| **Experimental design and statistics**<br><br>Full details of the experimental design and statistical methods used should be given in the Methods section, as detailed in our Minimum Standards Reporting Checklist. Information essential to interpreting the data presented should be made available | Yes |

Powered by Editorial Manager® and ProduXion Manager® from Aries Systems Corporation

| | |
|---|---|
| in the figure legends.<br><br>Have you included all the information requested in your manuscript? | |
| **Resources**<br><br>A description of all resources used, including antibodies, cell lines, animals and software tools, with enough information to allow them to be uniquely identified, should be included in the Methods section. Authors are strongly encouraged to cite Research Resource Identifiers (RRIDs) for antibodies, model organisms and tools, where possible.<br><br>Have you included the information requested as detailed in our Minimum Standards Reporting Checklist? | Yes |
| **Availability of data and materials**<br><br>All datasets and code on which the conclusions of the paper rely must be either included in your submission or deposited in publicly available repositories (where available and ethically appropriate), referencing such data using a unique identifier in the references and in the "Availability of Data and Materials" section of your manuscript.<br><br>Have you have met the above requirement as detailed in our Minimum Standards Reporting Checklist? | Yes |

Manuscript

Click here to download Manuscript NEPC lncRNA - v5.12 - 2017.04.28.docx

# The Long Noncoding RNA Landscape of Neuroendocrine Prostate Cancer and its Clinical Implications

Varune Rohan Ramnarine[1], Mohammed Alshalalfa[2], Fan Mo[1], Dong Lin[1,3], Noushin Nabavi[1], Nicholas Erho[2], Mandeep Takhar[2], Hussam Al-Deen Ashab[2], Sonal Brahmbhatt[1], R. Jefferey Karnes[4], Mark A Rubin[5], Himisha Beltran[6], Harrison Tsai[7], Tamara L Lotan[7], Stanislav V Volik[1], Alexander W Wyatt[1], Elai Davicioni[2], Yuzhuo Wang[1,3] and Colin C Collins[1]

[1]Vancouver Prostate Centre & Department of Urologic Sciences, University of British Columbia, Vancouver, BC, Canada; [2]GenomeDx Biosciences Inc., Vancouver, BC, Canada; [3]Department of Experimental Therapeutics, BC Cancer Agency, Vancouver, BC, Canada; [4]Department of Urology, Mayo Clinic College of Medicine, Rochester, MN, USA; [5]Department of Pathology and Laboratory Medicine, Weill Cornell Cancer Center, Weill Cornell Medical College, New York, NY, USA; [6]Department of Medicine, Weill Cornell Cancer Center, Weill Cornell Medical College, New York, NY, USA; [7]Department of Pathology, Johns Hopkins School of Medicine, Baltimore, MD, USA

**Running title:** NEPC lncRNAs
**Keywords:** neuroendocrine prostate cancer, transdifferentiation, small cell carcinoma, long non-coding RNA
**Total number of figures and tables:** 3 figures and 1 table
**Total number of supplementary figures and tables:** 8 figures and 10 tables

# ABSTRACT

**Background**

Neuroendocrine prostate cancer (NEPC) is an aggressive variant of late stage prostate cancer that most commonly arises through neuroendocrine transdifferentiation (NEtD). Treatment options are limited, ineffective, and in most patients, results in death in less than a year. We recently developed a first-in-field patient-derived xenograft (PDX) model of NEtD. Longitudinal deep transcriptome profiling of this model enabled monitoring of dynamic transcriptional changes during the NEtD process and in the context of selective treatment pressure. In the current study, we focused on long non-coding RNAs (lncRNA), which have been implicated in tumorigenesis and cancer progression through gene regulation, and whose roles in NEtD remain largely unexplored.

**Results**

We implemented a next-generation sequence analysis pipeline that can detect transcripts at low expression levels and built a genome-wide catalogue (n=50,000) of lncRNAs. We applied this pipeline to 927 clinical samples and our high fidelity NEtD model and identified 4,720 lncRNAs in NEPC. Among these were 216 lncRNAs capable of robustly classifying NEPC from prostate adenocarcinoma (AD) patient tumours. Another 2,553 were associated with the NEtD process, identified through five distinct patterns of expression in our PDX model and clinical samples. We interrogated our top deregulated candidates (n=106) in primary tumours obtained from radical prostatectomies all with long-term follow-up (median 18 years) and discovered significant clinicopathological associations. Specifically, we identified 26 that are associated with rapid metastasis following androgen deprivation therapy (ADT). Two of these lncRNAs (SSTR5-AS1 and LINC00514) significantly stratified patients undergoing ADT based on patient outcome.

**Discussion**

A comprehensive characterization of the dynamic expression of lncRNAs during the NEtD process has not been performed to date. Temporal dissection of our PDX-based NEtD model has provided insights

into lncRNAs linked to NEtD. Furthermore, select NEtD lncRNAs provide strong evidence for an association with metastasis and patients receiving ADT, which are clinical features of NEPC tumours. Candidate protein coding transcripts (KDM4B, TADA3, FOXF1, and SSTR5) have NEPC associations and are identified here as being regulated by NEtD lncRNA. In *vivo* and in *vitro* functionalization is required to validate these findings and to identify the mechanisms of action and/or interaction of these lncRNAs.

**Conclusion**

We present here for the first time a genome-wide catalog of NEtD lncRNAs that characterize the transdifferentiation process and a NEPC lncRNA expression signature. To accomplish this, we carried out the largest integrative study applying a PDX NEtD model to clinical samples performed to date. These NEtD and NEPC lncRNA represent strong candidates for clinical biomarkers and therapeutic targets, warranting further investigation in these respective areas.

# Introduction

Prostate cancer (PCa) is the most common type of cancer affecting men and is estimated to be the third highest cause of cancer death in developed countries globally[1]. Advances in detection and treatment for PCa have translated to many men being 'cured' by surgery and/or radiation. Concomitantly, androgen deprivation therapy (ADT) has resulted in significant survival gains for men with metastatic PCa. This treatment inhibits the androgen signaling axis, a growth and differentiation-inducing pathway mediated by the androgen receptor (AR). Despite these successes, with the steady accumulation of facilitating genomic and epigenomic aberrations, a more aggressive tumour capable of growing in castrate levels of testosterone can develop[2] termed castration-resistant prostate cancer (CRPC). Three main classes of treatment resistance to AR-targeted therapies exist, falling into two broad categories associated to AR signaling[3]. The majority of CRPC result in the reactivation of the AR signaling axis ($AR^+$ CRPC). Commonly administered therapeutics include Enzalutamide, Bicalutamide, and Abiraterone[4]. In parallel, some tumour cells leverage the increasing genomic and transcriptomic plasticity and progress to an AR-negative state ($AR^-$ CRPC), circumventing AR dependence. $AR^-$ CRPC is highly heterogeneous, but a major established aggressive subtype is neuroendocrine prostate cancer (NEPC)[5]. NEPC, also termed small cell carcinoma of the prostate (SCPC), expresses a dominant and irreversible neuronal-like phenotype[6] where conventional CRPC therapies are ineffective. Platinum-based chemotherapy is only transiently effective, resulting in poor overall survival[7] with most patients surviving ~7 months[8]. Molecular pathology markers include expression of chromogranin A (CHGA), synaptophysin (SYP), and neuro-specific enolase (NSE)[9] and negative (or low) levels of AR and AR-regulated genes such as PSA[6]. NEPC can arise *de novo* but much more commonly occurs as a consequence of ADT via an adaptive process termed neuroendocrine transdifferentiation[6,10](NEtD) and frequently metastasizes to visceral organs[11]. Emerging data suggest predisposing aberrations include loss of RB1[12] and TP53[13], gain of MYCN and AURKA[5], mutation of Trp53[14], overexpression of PEG10[15], N-Myc[16,17], SOX2[18], while the splice factor SRRM4[19] and master neural transcription factor BRN2[20] appear to be a drivers of NEtD. Discoveries such as these are the first to define protein-coding alterations characteristic of NEPC. However, the process of transdifferentiation is highly complex, and likely involves multiple layers of genetic and epigenetic regulation.

Dysregulation of long non-coding RNAs (lncRNA), previously unexplored in NEtD and master regulators within the cell, could provide an additional mechanism for the gene expression alterations that occur during NEtD. LncRNAs are broadly defined as large (>200 bp) RNA transcripts, with the most abundant subtypes classified as antisense RNAs, pseudogenes, and long intergenic noncoding RNAs (lincRNA)[21]. They have been implicated in a variety of diseases and their association with cancer progression has

been reported through mechanisms such as remodeling of chromatin, transcriptional co-activation or repression, protein inhibition, post-transcriptional modifiers, or as decoy elements[22]. LncRNAs form an important regulatory layer in global gene regulation and as such, alterations of lncRNA expression in cancer have been identified as one of the driving forces for tumorigenesis[23]. With emphasized research focus in the non-coding field of genomics, lncRNAs have emerged as crucial players in cancer progression and metastasis[24]. More specifically in prostate cancer, lncRNAs have been reported to play critical roles at every level of PCa progression, including the transformation of normal prostate cells to prostate intraepithelial neoplastic (PIN) cells, the development of localized PCa, and finally progression to advanced metastatic disease[25]. These disease progressive roles are due to aberrant lncRNA expression, which changes the balance of protein-coding genes involved in cellular proliferation and apoptosis, thereby accelerating cellular transformation.

We recently developed a first-in-field transplantable patient-derived xenograft (PDX) model of NEtD: a treatment-naïve adenocarcinoma (LTL331) that upon host castration initially regresses, but then rapidly relapses as terminally differentiated NEPC (LTL331R)[6]. In our previous study using this model, we demonstrated that the lack of evidence for NEPC cells before AR-blockade and the conservation of genome characteristics pre- and post-treatment strongly suggested a phase transition or state change from adenocarcinoma to NEPC[15]. In the current study, we used the longitudinal genomic profiling of our PDX-based NEtD model, yet focus on lncRNA transcripts. We hypothesize that aberrant lncRNA expression exist along this 'time series' and is associated with the development of NEPC. Our objective here is to comprehensively characterize the lncRNA landscape of NEtD and to ascertain their clinical relevance and associated clinicopathological features. To accomplish this, we implemented a sequence analysis pipeline optimized for the detection of lncRNAs, identified a signature that can robustly classify NEPC from AD tumours, and identified five NEtD associated lncRNA expression profiles. Lastly, we observed that a subset of these lncRNAs was associated with rapid metastasis in treated patients and are able to stratify samples based on patient outcome. We present here for the first time a comprehensive landscape of NEtD lncRNAs and their clinical associations.

# Results

## *Catalogue of Poly(A) long non-coding RNAs in neuroendocrine prostate cancer*

To identify lncRNAs involved in the progression from AD to NEPC, we performed next-generation polyadenylated RNA sequencing on our xenograft model LTL331 of NEtD. We implemented a next-generation sequence analysis pipeline composed primarily of algorithms from the Tuxedo suite of analysis tools[26] . We augmented this pipeline to include windowed adaptive quality control corrections (see Methods and Supplementary Figure 1-2) and to increase the ability to detect low abundance transcripts. This methodology was employed because lncRNAs are typically expressed at low levels. We applied the pipeline to our xenograft models (n=10) and clinical specimens (n=117) acquired from the Vancouver Prostate Centre (VPC) and Weill Cornell Medicine (WCM) (Table 1A and 1B). Using a *quasi de novo* mapping strategy combined with amalgamating all sample transcriptome assemblies, we identified 261,740 annotated transcripts spanning 38 Ensembl transcript classes (Supplementary Table 2). Defined by Ensembl's core biotypes, transcripts they were classified as either protein-coding RNAs, long ncRNAs, short ncRNAs, or pseudogenes, which totaled 131,458 (~50%), 94,620 (~36%), 12,992 (~5%) and 22,670 (~9%), respectively (Figure 1A – Panel 1-2). Within long ncRNA, seven classes exist; processed transcripts, retained intron, lincRNA, antisense, sense intronic, sense overlapping, and 3 prime overlapping ncRNA (Figure 1A – Panel 3). Despite pseudogenes not being included within Ensembl's long ncRNA classes (listed above), they are by definition considered under the umbrella of lncRNA[21]. Only

three classes were capable of distinctly distinguishing NEPC and AD clinical samples (Supplementary Figure 3-4). For this reason, all downstream analysis focused on these three lncRNA classes (50,000 transcripts in total – Figure 1B); antisense (15,276), pseudogenes (22,297) and lincRNAs (15,276) – collectively referred to as lncRNAs here on in. We explored these lncRNAs in our samples through two alternate discovery paths, which we later merged for our clinicopathological analysis (Figure 1C). The outline presented in this figure represents the studies overall workflow.

### *Long non-coding RNA expression profiles classify neuroendocrine prostate cancer*

Recently it has been shown that $AR^-$ and $AR^+$ CRPC share substantial genomic overlap yet display significant epigenetic differences[27]. Here, we hypothesize that the lncRNA transcriptome would similarly show unique and common expression alterations between $AR^+$ and $AR^-$ CRPC (unexplored to date). To investigate this, we used our $AR^{+/-}$ CRPC xenograft models (Table 1A) to identify changes occurring temporally within the same tumour pre- and post-treatment. Once chemically castrated our three AD models (LTL313, LTL418, and LTL331) progress to either $AR^+$ CRPC (LTL313BR and LTL418BR) or $AR^-$ CRPC/NEPC (LTL331R). This allowed for the identification and quantification of differentially expressed transcripts between pre- and post-CRPC. We integrated these models to patient tumours with matched clinical information to ensure our results were clinically relevant and to remove any model-based bias. As we suspected, among all lncRNAs altered (>2 fold, p-value<0.05) between pre- and post-CRPC, only 9.3% (n=330) were commonly deregulated in both CPRC subtypes and the remaining transcripts (n=3219) showed unique changes in either AR+ or AR- CRPC (Supplementary Table 5 and Supplementary Figure 5). This data supports the notion that AR+ and AR- CRPC contain diverse lncRNA landscapes.

LncRNA expression may be useful as an additional biomarkers to those currently used in the diagnosis of NEPC (i.e. chromogranin A, synaptophysin, and neuron-specific enolase). Moreover, an lncRNA expression signature would strongly support the involvement of lncRNAs in NEPC at a molecular and cellular level. These lncRNAs would be candidates for the mechanism driving the deregulation of known protein coding genes (PEG10, N-Myc, SOX2, SRRM4, REST, etc…) in NEPC/NEtD. To build a lncRNA expression signature for NEPC, we selected the top five percentile of transcripts based on standard deviations of expression for the VPC and WCM cohorts independently and performed unsupervised hierarchical clustering (UHC). This produced 1,415 and 1,317 NEPC lncRNAs in the VPC and WCM cohorts, respectively. Taking the intersection of these lists and then repeating UHC generated an expression signature of 216 lncRNAs (Supplementary Table 4) that distinctly segregated NEPC from AD tumours (Figure 1D-E). To assess the robustness of this signature, we validated it on an external clinical cohort of tumours (n=33 – Table 1A) from Johns Hopkins School of Medicine (JHSM). These tumours contained 17 AD and 16 NEPC samples and were profiled on the Human Exon array 1.0 ST platform (see Methods) compared to our discovery cohorts. Using the same approach (UHC), a clear separation of NEPC and AD was observed (Supplementary Figure 6). To the best of our knowledge, this is the first report of lncRNAs exhibiting a unique unbiased expression profile for NEPC and robustly segregating NEPC from AD patient samples.

### *Long non-coding RNAs are associated with the progression to neuroendocrine prostate cancer*

A major goal of this study was to characterize the lncRNA landscape during the dynamic phase transition from adenocarcinoma to NEPC using our unique PDX model LTL331 (Figure 2A). We recently investigated this model and identified protein-coding transcripts such as PEG10[12] and SRRM4[13] that are active in the phase transition and appear to be novel drivers of NEtD. In addition to these, our model has led to the identification of potential biomarkers and therapeutic targets for NEPC, including the DEK

proto-oncogene [28], BRN2[20], and epigenetic regulators CBX2 and EZH2[29] (members of the polycomb group family of transcriptional repressors). However, none of our previous studies have comprehensively investigated lncRNAs within our NEtD model. Here, we further explore the intermediate time points (pre-NEPC) where lncRNA transcripts whose expression modulates in response to treatment resulting in NEPC can be identified. Pre-NEPC represents a phenotype that currently is not characterized as a clinical entity but offers invaluable insight into the transcriptome of transdifferentiating adenocarcinoma cells. We characterized the transcriptome profiles at 8- and 12-week time points (pre-NEPC) post-castration when tumour volume and serum PSA levels reach nadir (Figure 2A). To do this, we deeply sequenced six samples of our PDX NEtD model representing three primary time points in duplicate (AD, pre-NEPC, and NEPC) along the phase transition.

We identified and quantified all lncRNA transcripts that were altered across the time series and defined five patterns of transcript expression: (a) those exhibiting a continuous decline in expression (Class I – AR-regulated, n=6,449); (b) those increasing expression from either AD to pre-NEPC or pre-NEPC to NEPC (Class II – Neuronal, n=17,420); (c) those with continuous increased expression (Class III – Persistent, n=4,339); (d) those with maximum expression at pre-NEPC (Class IV – Transient, n=9,560); (e) and those with minimum expression at pre-NEPC (Class V – Reactivated, n=6,574) (Figure 2C – grey triangles). Due to the dominance of androgen receptor (AR) regulated genes in AD, transcripts in Class I following this decreasing trend are likely due to the absence of androgen (post-castration) and thus probable AR-regulated lncRNA. Conversely, transcripts in Class II with the highest expression post-castration are likely constituents of the neuronal phenotype present in NEPC. The remaining three (Class III – Persistent, Class IV – Transient, and Class V – Reactivated) are candidates for mechanistic involvement in the transdifferentiation process.

To determine the clinical relevance of these lncRNAs from our PDX NEtD model, we integrated patient samples (VPC and WCM, Table 1B – Column "Clinical Group") with time points in our model (Figure 2B). Terminal NEtD model points (AD and NEPC) were appropriately aligned to AD and NEPC clinical samples. Yet, due to the complete lack of pre-NEPC clinical speciums we hypothesized that neoadjuvant treated AD (NHT) tumors would best match with this intermediate tumour state. The transcriptomes from these patients have been shown to display the effects of therapy response and more specifically androgen depletion[30]. In fact, neuroendocrine differentiation has been shown to increase after only three months of NHT in a retrospective analysis of 103 radical prostatectomy specimens[31]. These early events are the specific alterations we sought to isolate from the pre-NEPC time points of our PDX model. We also postulated that a subset of Class I – AR-regulated transcripts (down-regulated in our xenograft model) would be up-regulated in our (AR+) CRPC clinical samples due to reactivation of the AR signalling axis in classical CRPC[30,32-34]. Based on this model-to-clinical sample integration, the following patient group-wise comparisons were performed; NEPC vs. AD, NEPC vs. NHT, CRPC vs. AD, NHT vs. NAÏVE (untreated AD) and NHT vs. NAÏVE in combination with NEPC vs. NHT. This produced 6,961, 2,378, 3,843, 5,706 and 377 transcripts (>2 fold with p-value < 0.05 – Supplementary Table 6), respectively. Taking these results and integrating them with the PDX models aberrantly expressed transcripts from above, led to the identification of 2,553 lncRNA associated to the pathogenesis of NEPC (Figure 2C). These included 1,728, 680, 268, 132, and 19 that were within the following: Class I – AR-regulated, Class II – Neuronal, Class III – Persistent, Class IV – Transient, and Class V – Reactivated, respectively. Prominent examples observed in our NEtD model (Figure 2D), WCM cohort (Figure 2E), and VPC cohort (Figure 2F) illustrate each of these NEtD defining transcript classes. Unsupervised hierarchical clustering of Class I – AR-regulated,

Class II –Neuronal, and Class III – Persistent lncRNAs within WCM (Figure 2G) and VPC (Figure 2H) cohorts exhibit a distinct separation of AD and NEPC tumours (columns of heat map) and as expected, a distinct separation between Class I – AR-regulated and Class II – Neuronal/Class III – Persistent lncRNA (rows of heat map). Class IV – Transient and Class V – Reactivated lncRNA were excluded from this illustration due to their lack of altered expression between AD and NEPC samples.

Taken together, these NEtD lncRNAs (n=2,553) characterize the transdifferentiation that occurs to adenocarcinoma cells post-castration and are associated with the pathogenesis of treatment induced and resistant NEPC. We present here a genome-wide catalogue and resource for lncRNA in NEtD that warrants further exploration and validation for therapeutic candidacy.

### *Neuroendocrine prostate cancer long non-coding RNAs are involved in treatment-associated metastasis*

To determine if our NEtD (n=2,553) and NEPC (n=216) lncRNAs are associated with clinical outcomes in patients with primary prostatic adenocarcinoma, we explored our candidates in two cohorts from the Mayo Clinic (MCI[35] and MCII[36]) from the Decipher GRID database (GRID) (n=777, Table 1B). We could not preform this analysis within VPC/WCM cohorts due to their small sample sizes and short-term clinical follow up. The GRID cohorts represent tumors primarily with adverse pathology (i.e., high grade/stage) and long-term follow up for treatment and outcomes (median 18 years). From these cohorts, a subset (n=211) received adjuvant androgen deprivation therapy (ADT) post-radical prostatectomy (RP). To determine the most clinically relevant lncRNA transcripts in primary adenocarcinoma tumors, we first ranked the NEtD/NEPC lncRNAs within their respective classes and selected the top-ranked from each. The ranking was performed based on fold changes observed within our clinical groups (see Methods). This produced 106 top ranking NEtD/NEPC lncRNA (Supplementary Table 7) that we investigated within the GRID cohorts for their ability to predict rapid metastatic development post-ADT. Due to the difference in profiling platforms between GRID (Affymetrix microarray) and VPC/WCM cohorts (Illumina Sequencing), it was necessary to remap the GRID microarray probes (see Methods) that aligned within NEtD/NEPC lncRNA sequenced regions. This resulted in 85/106 (80%) being present and quantifiable on the microarray platform.

A characteristic of NEPC patients in the clinic is the occurrence of rapid metastasis following treatment[37]. We performed receiver operating characteristic (ROC) analysis to compare the sensitivity and specificity of predicting rapid metastasis (within 36 months) for each lncRNA. For each lncRNA ROC, we calculated the area under the curve (AUC) in both cohorts using probe set region expression summarized across the full lncRNA transcript (Supplementary Table 7). This identified eight lncRNA: NR2F1-AS1, LINC00654, FENDRR, PCAT2, and NKX2-1-AS1 in MCI (AUC>0.70) and LINC00478, LINC00173, and LINC00514 in MCII (AUC>0.70) with the highest scores. These lncRNAs serve as candidates for predicting rapid metastasis in patients receiving ADT, however clinical and experimental follow-up is necessary to validate these findings. Selecting all NEtD/NEPC lncRNAs with AUC>0.65 (n=26), we performed survival analysis to ascertain their ability to separate patients for metastasis as an outcome. Specifically, we calculated Kaplan-Meier estimates for metastatic disease progression stratified by median expression in ADT-treated samples of the MCII cohort. The expression of two NEtD/NEPC lncRNA transcripts (SSTR5-AS1 and LINC00514) was able to separate patients more likely to develop metastatic disease from those that did not (p-value=0.005 and p-value=0.010, respectively – Figure 3A). To increase our confidence that the results were associated with treatment status, we generated Kaplan-Meier estimates for these transcripts in untreated patients from the same cohort, and neither showed significant separation in their performance (p-value=0.905 and p-value=0.832, respectively – Figure 3B). These results suggest that a strong association with treatment status and increased probability of metastatic disease exists

between patients with differential expression of these lncRNA. Taken together, the previous results combined with these implicate SSTR5-AS1 and LINC00514 in NEtD and serve as strong candidates as predictive biomarkers for metastatic disease post-RP following ADT.

One of the mechanisms observed with lncRNAs is direct RNA-RNA interaction with mRNA molecules, resulting in regulation of their expression (activation or repression) and function. Computationally this type of investigation is intensive, and there are limited algorithms available to identify putative mRNA targets genome-wide. Using a recently published method to predict lncRNA-mRNA interactions comprehensively[38], we sought to identify candidate mRNA transcripts interacting with SSTR5-AS1 and LINC00514. The pipeline's three core algorithms include: Raccess[39] for the identification of accessible regions within the lncRNA, IntaRNA[40] to calculate nucleotide interaction energies, and RactIP[41] to predict joint secondary structures. Applying this methodology to SSTR5-AS1 and LINC00514 produced a list of predicted interacting partners for these lncRNAs (Supplementary Table 8-9). The top-ranked mRNAs were KDM4B and TADA3 that are predicted to hybridize independently and form joint structures with SSTR5-AS1 and LINC00514 (Supplementary Figure 7-8), respectively. In our clinical cohorts, TADA3 is down-regulated in NEPC versus AD (>2 fold), while KDM4B is up-regulated (>5 fold), however only TADA3s deregulation is statistically significant (VPC p-value=0.003 and WCM p-value=0.017). Both genes have NEPC associations (see Discussion), yet experimental validation would be necessary to confirm their regulation via these lncRNAs.

# Discussion

Primary NEPC arises *de novo* in 0.5% to 2% of all prostate cancer patients[42]. However, treatment related NEPC (tNEPC) can develop in approximately 20% to 30% of mCRPC tumours[43] and increases with disease progression[44]. The real incidence of tNEPC may be higher because of under-recognition due to tumour heterogeneity, the limited number of metastatic tumour biopsies performed, lack of uniform consensus definition based on histology or biomarker expression, and frequent misclassification as high-grade PCa, most notable in tumours with mixed histologies[45]. NEPC can be induced in vitro in AR+ LNCaP cells in androgen depleted culture conditions[46,47], similarly in *vivo*[6,48], and in patient tumours long-term ADT has increased neuroendocrine differentiation[37,43,49]. It is now common to observe treatment-resistant tumours with neuroendocrine features upon metastatic biopsy, and the prevailing consensus is epithelial plasticity enables tumour adaptation in response to AR-targeted therapies[6,8,37,45,50-52]. This evidence supports the notion that NEtD incidence will increase as new powerful ADTs enter the clinic. The urgency for therapeutic strategies and clinical biomarkers defining NEPC, if not high before, will soon reach a critical state. Currently, the only option for patients is the short-lived effects of platinum-based chemotherapy. Optimism is on the rise as there is an AURKA inhibitor (MLN8237) in Phase 2 clinical trials (NCT01799278), targeting strategies under development for PEG10 (another NEPC oncogene), and increased study of NEPC in general.

In this study, we sought to characterize the unexplored genome-wide lncRNA landscape of NEtD to provide insights into the NEPC non-coding milieu of this lethal disease. This required the implementation of a sequence analysis pipeline with increased sensitivity towards lower expressed transcripts, characteristic of lncRNAs. Our pipeline was able to detect 50,000 lncRNA transcripts (subclassified as either lincRNA, antisense or pseudogene) and quantify them in our two clinical cohorts (VPC and WCM). The novelty of this study lies in the use of patient samples integrated with our PDX model to detect clinically relevant lncRNAs involved in the NEtD/phase transition process. In this study, we identified 2,553 lncRNAs associated with NEtD and observed that subsets of these have strong clinical associations

with metastatic PCa patients after receiving ADT. Furthermore, we identify a robust 216 NEPC lncRNA signature capable of classifying NEPC from AD patient samples. In previous lncRNA studies in cancer, several have been linked to malignant transformation with key roles affecting various aspects of cellular homeostasis including proliferation, survival, migration, and genomic instability[53]. Similarly, lncRNA identified in this study including SSTR5-AS1 and LINC00514 with their association with poor outcome and FENDRR with its association with rapid metastasis could be mechanistically linked to NEtD.

FENDRR is a top deregulated lncRNAs in Class IV – Transient and may have a role in the NEtD process. It has been implicated in a lethal lung development disorder[54], lung cancer[55], and can bind to PRC2[56,57]. PRC2 plays significant roles in tumour progression through binding of HOTAIR (a very well-studied lncRNA). Together, HOTAIR and PRC2 are involved in the control of chromatin structure and associated gene activity[58]. FENDRR may be involved in tumorigenesis in a manner similar to HOTAIR due to its known interaction with PRC2. A recent study showed down-regulation of FENDRR is associated with poor prognosis in gastric cancer and regulates cancer cell metastasis through fibronectin[59]. Functionally, this could be occurring in NEPC as well due to FENDRRs transient expression in our NEtD model and its association to rapid metastasis in ADT-treated PCa patients from our GRID (MCI) cohort. Another putative function of this transcript is through upregulating FOXF1, which is a protein coding gene and the sense form for the antisense transcript FENDRR. Antisense transcripts are known to regulate their sense forms (positively or negatively). Using TANRIC, an interactive resource for the exploration of lncRNA in large patient cohorts within 20 TCGA cancer types[60], we see that FENDRR expression is positively correlated to FOXF1 in 16 of 20 cancer types (p-value $< 3.71\times10^{-9}$, Supplementary Table 10). FOXF1 is a target gene of p53 and seen to regulate cancer cell migration and invasiveness[61].

SSTR5-AS1 is the highest expressed lncRNA in our NEPC clinical samples when requiring expression concordance in VPC and WCM cohorts. It is an antisense transcript of SSTR5, which is a member of the superfamily of somatostatin receptors. Somatostatins are peptide hormones that regulate diverse cellular functions such as neurotransmission, cell proliferation, and endocrine signalling, as well as inhibiting the release of many hormones and other secretory proteins. The SSTR family (1 through 5) are markers for neuroendocrine tumours of the lung (NELC)[62] with SSTR1 and SSTR5 the most dominant forms of SSTR in neuroendocrine tumours in general[63]. Interestingly, exploration within TANRIC showed a strong positive correlation in expression with SSTR5 to SSTR5-AS1 in 14 of 20 cancer types (p-value $< 2.18\times10^{-15}$, Supplementary Table 10). Furthermore, SSTR5 is detectable in the blood of NELC[64] and could be a valuable non-invasive diagnostic marker for NEPC. In fact, clinicians utilize this biological feature in other neuroendocrine tumours (NETs) using Octreoscans to determine tumour stage and/or identification of sites of metastasis. Octreoscans when compared to positron emission tomography (PET) scans (a commonly used approach for this) appears more sensitive in the detection of well-differentiated NETs[65]. In addition to this, therapeutically, somatostatin analogues are emerging a promising treatment option for inoperable or metastatic NETs[66]. However, specifically in NEPC, targeting SSTR5 and/or SSTR5-AS1 for diagnostic or therapeutic purposes has yet to be done. Interestingly, SSTR5 (C-terminal) is required for Rb induction and G1 cell cycle arrest[67], resulting in anti-proliferative effects. However, without Rb (known to be lost in NEPC), this function would be negated. Alternatively, our interaction prediction provided evidence for SSTR5-AS1 and KDM4B (Supplementary Figure 7), which provides another strong connection to NEPC biology. KDM4B is a histone demethylase shown to interact physically with N-Myc and epigenetically regulate and activate this oncogene in neuroblastoma[68]. N-Myc has been seen to drive the progression of NEPC[5,16,17] and recently through EZH2 mediated transcription[17]. Another mechanism of activation could be facilitated through SSTR5-AS1 regulation. However, both of these putative functions (SSTR5-AS1:SSTR5 or SSTR5-AS1:KDM4B:N-Myc) require thorough in *vitro* and in *vivo* exploration to ascertain their validity.

LINC00514 is amongst the highest expressed lncRNAs in Class III – Persistent. It has not been characterized. It is predicted to bind to TADA3 (Supplementary Figure 8), potentially causing a reduction of its activity. This is intriguing because TADA3 is involved in the stabilization and activation of p53[69,70] and this putative interaction (LINC00514:TADA3) could be a mechanism for loss of p53 activity, already known to be frequently lost in NEPC[13].

Although multiple layers of genetic and epigenetic deregulation likely cooperate to facilitate NEtD, understanding the non-coding contribution to this multifarious process is necessary to design effective novel therapeutics. Using our five independent patient cohorts and our proven NEtD PDX LTL331 model, lncRNAs such as FENDRR, SSTR5-AS1, LINC00514 and others identified in this study may provide deeper insights to NEtD and NEPC. Research identifying the relationship of these lncRNAs to other known drivers of NEtD is now required. Taken together, this study is the first to report the lncRNA landscape of NEtD, a robust NEPC lncRNA expression classifier, and provides numerous candidates for evaluation of biomarkers and therapeutic targets.

# METHODS

## *Patient-derived xenografts*

Six LTL331, two LTL313, and two LTL418 patient-derived xenografts were raised in NOD-SCID mice (NOD.CB17-Prkdcscid/J) exactly as previously described[6]. Xenograft tissue was harvested after fixed lengths of time post host castration, and tissue was measured, fixed for histopathological analysis, and processed for RNA analysis.

## *Clinical datasets*

We used five clinical cohorts from 1) Weill Cornell Medicine (WCM)[5]; 2) GenomeDx Biosciences (GX) Inc. (MCI and MCII); 3) Johns Hopkins School of Medicine (JHSM); and 4) Vancouver Prostate Centre (VPC), cumulatively totaling 927 samples. For the VPC, 80 specimens were obtained from patients undergoing radical prostatectomy (RP) and snap frozen following a protocol approved by the Clinical Research Ethics Board of the University of British Columbia (UBC), the BC Cancer Agency and Vancouver General Hospital pathology (depending on the sample source). All patients signed a formal consent form approved by the ethics board. A subset of the GX Decipher GRID™ database of clinical specimens was selected, totally 777 patient PCa expression profiles (all from FFPE tissue) and were obtained from two RP Mayo Clinic (MC) cohorts that have been described previously (MCI[35] and MCII[36]). JHSM samples, totally 33 samples were retrieved from surgical pathology and consultation files of Johns Hopkins Hospital (John Hopkins Registry) from 1999-2013, as previously described[71]. The 33 were annotated as 6 morphologically-diagnosed pure prostate small cell carcinoma samples (SCPC), 12 high risk (GS9-10) Adenocarcinoma (AD), 10 SCPC (SC-mixed) and 5 AD (AD-mixed) from mixed histology tumors containing separate adenocarcinoma and small cell components. For this cohort, samples were dicotimized into either AD (AD and AD-mixed samples) or NEPC (SCPC and SCPC-mixed sampels) for the purposes of validating our 216 NEPC lncRNA signature.

## *Material collection and processing (VPC Cohort)*

Hematoxylin and eosin stained FFPE and frozen sections were reviewed by a pathologist (LF) to identify blocks with highest tumor content. For each frozen block used, a 5µm slide was first taken for H&E staining; then 4x100µm sections were taken for DNA and RNA isolation before a 2nd 5µm slide was taken for H&E staining. Each H&E slide was required to have tumor content >50% for a tumor to proceed for sequencing. RNA from 100µm sections of snap-frozen tissue was isolated using the mirVana Isolation Kit from Ambion (AM 1560).

## *Material collection and processing (GRID and JHSM)*

For GRID (MCI and MCII) and JHSM cohorts, specimen selection, RNA extraction and microarray hybridization was performed for these samples in a Clinical Laboratory Improvement Amendments (CLIA)-certified laboratory facility (GenomeDx Biosciences, San Diego, CA, USA) as described previously. Total RNA extraction, purification, and RNA amplification and labelling were done using the Ovation WTA FFPE system (NuGen, San Carlos, CA). RNA was hybridized to Human Exon 1.0 ST GeneChips (Affymetrix, Santa Clara, CA). After microarray quality control using the Affymetrix Power Tools packages, probe set normalization was performed using the Single Channel Array Normalization (SCAN) algorithm[72].

### *Next-generation sequencing*

RNA sequencing was performed on Illumina HiSeq 2000 at BCCA Michael Smith Genome Sciences Centre according to standard protocols. A subset of samples (n=70) used in this study were from previous studies with all raw data reanalyzed, including the 6 LTL331 model samples[15], 4 NEPC (VPC) samples[30,73], 23 AD (VPC) samples[30], 30 AD (WCM) samples[5], 7 NEPC (WCM) samples[5] and all others (n=57) unpublished (Supplementary Table 1).

### *RNA sequence analysis pipeline*

We implemented a lncRNA sequence analysis pipeline that includes algorithms catered to the detection of known and novel transcripts (see Supplementary Figure 1A). Developed in-house, this pipeline is modified and extended from the tuxedo suite of sequence analysis algorithms[26]. Once received from the sequencing centre in bam format, all sequenced model systems and patient samples were de-aligned into raw fastq format (including flagged reads) using bam2fastq and put through the following pipeline. To ensure high-quality sequence reads, libraries were trimmed using a windowed adaptive approach (Sickle – https://github.com/ucdavis-bioinformatics/sickle). For each read pair processed together, the algorithm determines the most optimal inner read sequence by trimming both 3' and 5' prime ends based on quality and length thresholds (for full description see – http://bioinformatics.ucdavis.edu/software/). Bases with a quality score of less than 99.0% base call accuracy (corresponding to a Phred quality score of 20) were removed. Reads less than ~2/3 read length (30bp in WCM and 60bp in VPC) post-trimming were discarded. Highly repetitive sequences (>2% of library) were also discarded post-trimming using the cutadapt tool. All quality control metrics were generated (pre- and post-trimming) using the FASTX-Toolkit and the FastQC Windows software. Reads were aligned to the Hg19 human genome build using an unspliced aligner for handling exonic reads (Bowtie), in conjunction with a spliced aligner to handle reads spanning exon-exon junctions (Tophat). Transcriptome reconstruction using Ensembl GRCh37.75 gene tracks for each library was performed using a quasi de novo (genome-guided) approach (Cufflinks), where reads were assembled and abundances estimated using an overlap graph producing a minimal spanning network of transcripts. This version of Ensembl contained 38 transcript classes, grouped by four core biotypes (Supplementary Table 2). At this stage, transcripts were also multi-read and fragment bias corrected. Transcripts with highly abundant expression were masked (e.g. rRNAs) from downstream steps to increase transcript quantification accuracy. Sample transcriptomes, the reference genome, and the transcript annotation were then meta-assembled (Cuffmerge) to produce a single annotation transcriptome model. Based on this model, transcript quantification (Cuffquant) and normalization (cuffnorm) for library depth and transcript length were performed. Transcript expression displaying computational artifacts (expression values < 0.1 known to occur with Cufflinks) were converted to zero values. This generated transcript expression where only lncRNA (Ensembl and ENCODE-based) were extracted and used for all downstream analysis. All algorithms denoted in brackets are referenced and described in Trapnell et al. Nature protocol[26]. Each cohort (VPC and WCM) was processed independently by this pipeline, and then transcriptome annotations were merged. This was accomplished using Ensembl transcript IDs combined with transcript lengths to produce unique transcript identifiers for each lncRNA across cohorts.

### *RNA-RNA Interaction Analysis*

A genome-wide search for SSTR5-AS1 and LINC00514 lncRNA interactions was preformed using a multistep systemic approach[38]. This tool is available publically within an online database (http://rtools.cbrc.jp/cgi-bin/RNARNA/index.pl) hosted by the Computational Biological Research Center (CBRC) at the National Institute of Advanced Industrial Science and Technology in Japan. The interaction search space included all hg19 annotated lncRNA and mRNA transcripts. The top-ranked (n=100)

interaction partners, based on local interaction minimum free energy, was generated using this approach (Supplementary Table 8-9). To visualize the top-ranked predictions KDM4B and TADA3 for SSTR5-AS1 and LINC00514, respectively (Supplementary Figure 7-8), R-chie[74] (http://www.e-rna.org/r-chie/) was used using the double structure feature. All bases that were not within the interaction site were predicted to form RNA secondary structure by RNAfold[75,76] (http://rna.tbi.univie.ac.at/cgi-bin/RNAfold.cgi) selecting enforced constrained pairing pattern for the interacting bases. Minimum free energy (MFE) structures were predicted by RNAfold on the 300bp sequences upstream and downstream of the interaction site.

### *Microarray to sequencing platform lift over / mapping3*

Affymetrix Human Exon 1.0 ST GeneChip probes were mapped to Hg19 coordinates using SMALT v0.76 (http://www.sanger.ac.uk/resources/software/smalt/). Probe set genomic regions (PSRs) were redefined accordingly. Exons within each lncRNA from sequencing cohorts (VPC and WCM) were integrated with PSRs to build an overlap table to determine absence/presence of lncRNA transcripts on the affymetrix microarray. R function iRanges v2.9.18 was used with method findOverlap to build described table above. Microarray PSRs were required to be completely within sequenced exon genome regions for exclusion, otherwise excluded. Applying this methodology 170 of 216 NEPC lncRNA (78.7%) and 85 of 106 NEtD lncRNA (80.2%) mapped to microarray PSRs for clinicopathological analysis on GROD cohorts MCI and MCII.

### *Statistical analysis*

For all cohorts, the programming language R v3.0 was used for statistical analysis. For VPC and WCM cohorts, unsupervised hierarchical clustering was performed with the h.clust package with correlation distance and average linkage used. Only transcripts within the top five percentile based on their standard deviations were selected. The clustering and heatmaps generated were built using the heatmap.2 function.  Similar clustering analysis was performed for GRID cohorts except with Euclidian distance, the ward method for linkage, and the use of the heatmap.3 function due to its advanced row/column labelling features. For all cohorts before clustering, expression values were standardized using a z-score that ranged from -3 to 3. For principal component analysis, the R package prcomp was used to calculate variance among transcript and sample subsets for the calculation of transcript weights and principle components. The top 3 components were used for visual inspection. For all clinical group-wise comparisons, a standard student t-test was applied (VPC, WCM, and GRID) to identify differentially expressed transcripts between groups/phenotypes. Significance thresholds were implemented by enforcing a p-value cut-off of $<0.05$. For receiver-operating characteristic (ROC) curves and area under the curve (AUC) calculations the R package 'pROC' was used. Kaplan-Meier analysis was performed using the R package 'survfit' with transcripts displaying below background ($>0.1$) expression being removed from this analysis.

### *Transcript Ranking*

NEtD lncRNA transcripts were ranked based on fold changes observed in appropriate clinical group-wise comparisons. For AR-regulated transcripts, the three group-wise comparisons utilized (NEPC vs. AD, CRPC vs. AD, and NHT vs. AD) were extracted, the minimum fold change observed was selected, and ranked in decreasing order. For Neuronal and Persistent transcripts, NEPC vs. AD fold changes were calculated and ranked in increasing order for both VPC and WCM cohorts, where the maximum fold change present was selected. Similar ranking was performed for NEPC lncRNA transcripts yet were ordered in increasing and decreasing order to determine the highest/lowest expressed transcripts in

NEPC vs. AD (transcripts independently ranked in VPC and WCM). For Transient and Reactivated transcripts, absolute fold changes for AD vs. NHT and NHT vs. NEPC were calculated and ranked in increasing order with the maximum fold change from either group selected. The top 20 lncRNAs were taken from each list, producing 106 isoforms representing 79 unique lncRNA transcripts. These represent the top NEtD/NEPC lncRNA candidates from our study. No pseudogenes were included in these rankings.


## FUNDING

This work was supported by the Mitacs Accelerate PhD Fellowship Program (IT04310 to VRR) in collaboration with GenomeDx Biosciences, Terry Fox Foundation (201012TFF to CC), and Prostate Cancer Canada Team Grant (T2013-01 to CC)

## AKNOWLEDGEMENTS

We are grateful to GenomeDx bioinformaticians Mandeep Takhar for her help with GRID statistical analysis/primer code, Hussam Al-Deen Ashab for his help with IA analysis (ultimately wasn't included in the paper) but efforts and knowledge gained from the results were insightful for this work, Nicholas Erho for his efforts in mapping our sequenced data to the GRID microarray, and Mohammed Alshalalfa for his guidance and supervision on all NEPC/NEtD lncRNA clinicopathological GenomeDx analysis. We would also like to deeply thank Daniel Lai and Alex Gawronski for their advice with RNA-RNA visualization and interaction analysis algorithms. We would like to thank Faraz Hach for his manuscript insights and advice. Lastly, we are extremely grateful to Stephanie Giles Hunt for her manuscript comments, advice and support.


## COMPETING INTERESTS

The authors declare that they have no competing interests.

## FIGURE LEGENDS

**Table 1:** Model and clinical samples used in the study. (A) $AR^+$ and $AR^-$ CRPC xenograft model samples and their associated molecular characteristics. (B) Patient samples and their associated clinical variables including treatment status, Gleason grading, and clinical endpoints.

**Figure 1**: Transcriptome composition and NEPC lncRNA expression signature. (A) Proportions and totals of transcript identifiable using next-generation sequencing pipeline. Transcripts were separated into protein coding (mRNA) or non-coding RNA (ncRNA) and as defined by Ensembl's core biotypes as either mRNA, long ncRNA, short ncRNA, or pseudogene. Within long ncRNA, there exist seven classes, including processed transcripts, retained intron, lincRNA, antisense, sense intronic, sense overlapping, and 3 prime overlapping ncRNA (the last three labelled as 'other'). Transcript totals are denoted around each pie chart. (B) Three transcript classes were used in this study due to their ability to separate AD and NEPC tumours, which collectively totalled 50,000 lncRNA. These lncRNA formed the basis for all down-stream analysis and this studies project workflow (C). Unsupervised hierarchical clustering (UHC)

of 216 identified lncRNA in VPC (D) and WCM (E) cohorts. Samples (columns) are labelled as adenocarcinomas (green) or neuroendocrine (red) tumours.

**Figure 2:** Model of neuroendocrine transdifferentiation, discovery methodology, and select NEtD associated lncRNA. (A) Schematic depicting the time points at which xenograft tumours were collected along the transdifferentiation of AD to NEtD (taken and modified from Akamatsu/Wyatt et al., 2015). (B) Phenotypes that align to various time points from above xenograft model and group-wise comparisons made in clinical samples. (C) Five isolated expression profiles (grey triangles) from select time points in A (light grey circles) with appropriate clinical group-wise comparisons overlaid. Select NEtD lncRNA that exemplifies each expression profile are shown from our (D) NEtD xenograft model, (E) WCM cohort, and (F) VPC cohort. The expression for NEtD lncRNA's within Class IV - Transient and Class V – Reactivated were only identified through our VPC cohort due to the presence of NHT samples, which were not present within the WCM cohort. Unsupervised hierarchical clustering with 2402/2553 of our NEtD lncRNA (Class I – AR-Regulated, Class II – Neuronal, and Class III – Persistent) clusters AD and NEPC samples within the (G) VPC cohort and (H) WCM cohort.

**Figure 3:** Expression and Kaplan-Meier estimates for SSTR5-AS1 and LINC00514. (A) Box plot expression for our top two NEPC lncRNA candidates within the VPC and WCM cohorts. Kaplan-Meier estimates for metastasis-free survival in the MCII cohort comparing low (red lines) and high (blue lines) expression (split by median) in treated patients that received post-prostatectomy adjuvant ADT (B) for SSTR5-AS1 (left) and LINC00514 (right) and patients not receiving ADT treatment (C).

## SUPPLEMENTARY FIGURE LEGENDS

**Table 1:** Clinical information for VPC and WCM patient samples, including sequencing details

**Table 2:** Transcriptome composition and Ensembl-based transcript totals for VPC and WCM cohorts with implemented next-generation sequence analysis pipeline

**Table 3:** Transcriptome composition and Cufflinks-based transcript totals for VPC and WCM cohorts with implemented next-generation sequence analysis pipeline

**Table 4:** LncRNA transcriptome annotation information, including names, genomic coordinates, transcript class, and marked presence/absence within model and clinical samples

**Table 5:** Differentially expressed lncRNA totals within $AR^-$ and $AR^+$ CRPC model samples

**Table 6:** Clinical group-wise comparisons, including group definitions, sizes, and number of significantly differentially expressed lncRNAs

**Table 7:** Top ranking NEtD lncRNA identified in study and their associated ROC/AUC values

**Table 8:** SSTR5-AS1 predicted RNA (mRNA or lncRNA) interactions with associated binding energies, predicted transcript Ensembl ID, name, interaction position, and ranking.

**Table 9:** LINC00514 predicted RNA (mRNA or lncRNA) interactions with associated binding energies, predicted transcript Ensembl ID, name, interaction position, and ranking.

**Table 10:**TANRIC results for lncRNA FENDRR and SSTR5-AS1. Spearman rank correlation for protein coding genes that are the sense form to the above antisense transcripts. Numbers in brackets denote p-values, no significant correlation is noted by NSC, and if mRNA data was not available for this tumor type a NA was given.

**Figure 1:** The next-generation sequence analysis pipeline implemented for the detection and quantification of lncRNA in this study (A). Sequencing quality control metrics before and after trimming of data for sample 1015LT is outlined in B-F. This includes (A) Phred quality scores and (B) percentage of each base type across read library at each base pair position and (C) over-represented sequences present within the library. After quality control corrections are applied, (C) read library has acceptable Phred quality scores (~30 Phred Score) and (D) expected base type percentages (~25%). All over-represented sequences (>2% of library) are removed from read library.

**Figure 2:** Average Phred quality scores for all VPC and WCM samples, pre- and post-quality control correction.

**Figure 3:** Unsupervised hierarchal clustering (A-D) and principle component analysis (E-H) on the four major Ensembl transcript classes detected within the VPC cohort. Samples are labelled as adenocarcinomas (blue) and neuroendocrine tumours (gold)

**Figure 4:** Unsupervised hierarchal clustering (A-D) and principle component analysis (E-H) on the four major Ensembl transcript classes detected within the WCM cohort. Samples are labelled as adenocarcinomas (blue) and neuroendocrine tumours (gold)

**Figure 5:** Detected and differentially expressed lncRNAs among $AR^{-/+}$ CRPC xenograft models (A) and matched clinical samples (B)

**Figure 6:** Validation of NEPC lncRNA (n=216) expression signature/classifier within an external small cell prostate cancer cohort (n=33). Samples are labelled as small cell carcinomas (red and dark red) and adenocarcinomas (green and olive)

**Figure 7:** Hypothetical RNA-RNA folding structure for exon 4 of SSTR5-AS1 (top) and the 3'UTR of KDM4B (bottom). Predicted base pair binding (green arcs) along the sequence (black arrow) are displayed, included predicted interaction site (orange bars).

**Figure 8:** Hypothetical RNA-RNA folding structure for exon 4 of LINC00514 (top) and the 3'UTR of TADA3 (bottom). Predicted base pair binding (green arcs) along the sequence (black arrow) are displayed, included predicted interaction site (orange bars).

# REFERENCES


1 Torre, L. A. *et al.* Global cancer statistics, 2012. *CA: a cancer journal for clinicians* **65**, 87-108, doi:10.3322/caac.21262 (2015).

2 Grasso, C. S. *et al.* The mutational landscape of lethal castration-resistant prostate cancer. *Nature* **487**, 239-243, doi:10.1038/nature11125 (2012).

3 Vlachostergios, P. J., Puca, L. & Beltran, H. Emerging Variants of Castration-Resistant Prostate Cancer. *Curr Oncol Rep* **19**, 32, doi:10.1007/s11912-017-0593-6 (2017).

4 Karantanos, T. *et al.* Understanding the mechanisms of androgen deprivation resistance in prostate cancer at the molecular level. *European urology* **67**, 470-479, doi:10.1016/j.eururo.2014.09.049 (2015).

5 Beltran, H. *et al.* Molecular characterization of neuroendocrine prostate cancer and identification of new drug targets. *Cancer discovery* **1**, 487-495, doi:10.1158/2159-8290.CD-11-0130 (2011).

6 Lin, D. *et al.* High fidelity patient-derived xenografts for accelerating prostate cancer discovery and drug development. *Cancer research* **74**, 1272-1283, doi:10.1158/0008-5472.CAN-13-2921-T (2014).

7 Aparicio, A. M. *et al.* Platinum-based chemotherapy for variant castrate-resistant prostate cancer. *Clinical cancer research : an official journal of the American Association for Cancer Research* **19**, 3621-3630, doi:10.1158/1078-0432.CCR-12-3791 (2013).

8 Wang, H. T. *et al.* Neuroendocrine Prostate Cancer (NEPC) progressing from conventional prostatic adenocarcinoma: factors associated with time to development of NEPC and survival from NEPC diagnosis-a systematic review and pooled analysis. *Journal of clinical oncology : official journal of the American Society of Clinical Oncology* **32**, 3383-3390, doi:10.1200/JCO.2013.54.3553 (2014).

9 Terry, S. & Beltran, H. The many faces of neuroendocrine differentiation in prostate cancer progression. *Frontiers in oncology* **4**, 60, doi:10.3389/fonc.2014.00060 (2014).

10 Shen, R. *et al.* Transdifferentiation of cultured human prostate cancer cells to a neuroendocrine cell phenotype in a hormone-depleted medium. *Urologic oncology* **3**, 67-75 (1997).

11 Palmgren, J. S., Karavadia, S. S. & Wakefield, M. R. Unusual and underappreciated: small cell carcinoma of the prostate. *Seminars in oncology* **34**, 22-29, doi:10.1053/j.seminoncol.2006.10.026 (2007).

12 Tan, H. L. *et al.* Rb loss is characteristic of prostatic small cell neuroendocrine carcinoma. *Clinical cancer research : an official journal of the American Association for Cancer Research* **20**, 890-903, doi:10.1158/1078-0432.CCR-13-1982 (2014).

13 Chen, H. *et al.* Pathogenesis of prostatic small cell carcinoma involves the inactivation of the P53 pathway. *Endocrine-related cancer* **19**, 321-331, doi:10.1530/ERC-11-0368 (2012).

14 Ku, S. Y. *et al.* Rb1 and Trp53 cooperate to suppress prostate cancer lineage plasticity, metastasis, and antiandrogen resistance. *Science* **355**, 78-83, doi:10.1126/science.aah4199 (2017).

15 Akamatsu, S. *et al.* The Placental Gene PEG10 Promotes Progression of Neuroendocrine Prostate Cancer. *Cell reports* **12**, 922-936, doi:10.1016/j.celrep.2015.07.012 (2015).

16 Lee, J. K. *et al.* N-Myc Drives Neuroendocrine Prostate Cancer Initiated from Human Prostate Epithelial Cells. *Cancer cell* **29**, 536-547, doi:10.1016/j.ccell.2016.03.001 (2016).

17 Dardenne, E. *et al.* N-Myc Induces an EZH2-Mediated Transcriptional Program Driving Neuroendocrine Prostate Cancer. *Cancer cell* **30**, 563-577, doi:10.1016/j.ccell.2016.09.005 (2016).

18 Mu, P. *et al.* SOX2 promotes lineage plasticity and antiandrogen resistance in TP53- and RB1-deficient prostate cancer. *Science* **355**, 84-88, doi:10.1126/science.aah4307 (2017).

19 Li, Y. *et al.* SRRM4 Drives Neuroendocrine Transdifferentiation of Prostate Adenocarcinoma Under Androgen Receptor Pathway Inhibition. *European urology*, doi:10.1016/j.eururo.2016.04.028 (2016).

20 Bishop, J. L. *et al.* The Master Neural Transcription Factor BRN2 is an Androgen Receptor Suppressed Driver of Neuroendocrine Differentiation in Prostate Cancer. *Cancer discovery*, doi:10.1158/2159-8290.CD-15-1263 (2016).

21 Gibb, E. A., Brown, C. J. & Lam, W. L. The functional role of long non-coding RNA in human carcinomas. *Molecular cancer* **10**, 38, doi:10.1186/1476-4598-10-38 (2011).

22 Cheetham, S. W., Gruhl, F., Mattick, J. S. & Dinger, M. E. Long noncoding RNAs and the genetics of cancer. *British journal of cancer* **108**, 2419-2425, doi:10.1038/bjc.2013.233 (2013).

23 Gutschner, T. & Diederichs, S. The hallmarks of cancer: a long non-coding RNA point of view. *RNA biology* **9**, 703-719, doi:10.4161/rna.20481 (2012).

24 Sahu, A., Singhal, U. & Chinnaiyan, A. M. Long noncoding RNAs in cancer: from function to translation. *Trends in cancer* **1**, 93-109, doi:10.1016/j.trecan.2015.08.010 (2015).

25 Cheng, W., Zhang, Z. & Wang, J. Long noncoding RNAs: new players in prostate cancer. *Cancer letters* **339**, 8-14, doi:10.1016/j.canlet.2013.07.008 (2013).

26 Trapnell, C. *et al.* Differential gene and transcript expression analysis of RNA-seq experiments with TopHat and Cufflinks. *Nature protocols* **7**, 562-578, doi:10.1038/nprot.2012.016 (2012).

27 Beltran, H. *et al.* Divergent clonal evolution of castration-resistant neuroendocrine prostate cancer. *Nature medicine* **22**, 298-305, doi:10.1038/nm.4045 (2016).

28 Lin, D. *et al.* Identification of DEK as a potential therapeutic target for neuroendocrine prostate cancer. *Oncotarget* **6**, 1806-1820, doi:10.18632/oncotarget.2809 (2015).

29 Clermont, P. L. *et al.* Polycomb-mediated silencing in neuroendocrine prostate cancer. *Clinical epigenetics* **7**, 40, doi:10.1186/s13148-015-0074-4 (2015).

30 Wyatt, A. W. *et al.* Heterogeneity in the inter-tumor transcriptome of high risk prostate cancer. *Genome biology* **15**, 426, doi:10.1186/s13059-014-0426-y (2014).

31 Ahlgren, G. *et al.* Regressive changes and neuroendocrine differentiation in prostate cancer after neoadjuvant hormonal treatment. *The Prostate* **42**, 274-279 (2000).

32 Wolf, D. A., Herzinger, T., Hermeking, H., Blaschke, D. & Horz, W. Transcriptional and posttranscriptional regulation of human androgen receptor expression by androgen. *Molecular endocrinology* **7**, 924-936, doi:10.1210/mend.7.7.8413317 (1993).

33 Cai, C. *et al.* Androgen receptor gene expression in prostate cancer is directly suppressed by the androgen receptor through recruitment of lysine-specific demethylase 1. *Cancer cell* **20**, 457-471, doi:10.1016/j.ccr.2011.09.001 (2011).

34 Knuuttila, M. *et al.* Castration induces up-regulation of intratumoral androgen biosynthesis and androgen receptor expression in an orthotopic VCaP human prostate cancer xenograft model. *The American journal of pathology* **184**, 2163-2173, doi:10.1016/j.ajpath.2014.04.010 (2014).

35 Erho, N. *et al.* Discovery and validation of a prostate cancer genomic classifier that predicts early metastasis following radical prostatectomy. *PloS one* **8**, e66855, doi:10.1371/journal.pone.0066855 (2013).

36 Karnes, R. J. *et al.* Validation of a genomic classifier that predicts metastasis following radical prostatectomy in an at risk patient population. *The Journal of urology* **190**, 2047-2053, doi:10.1016/j.juro.2013.06.017 (2013).

37 Beltran, H. *et al.* Challenges in recognizing treatment-related neuroendocrine prostate cancer. *Journal of clinical oncology : official journal of the American Society of Clinical Oncology* **30**, e386-389, doi:10.1200/JCO.2011.41.5166 (2012).

38 Terai, G., Iwakiri, J., Kameda, T., Hamada, M. & Asai, K. Comprehensive prediction of lncRNA-RNA interactions in human transcriptome. *BMC genomics* **17 Suppl 1**, 12, doi:10.1186/s12864-015-2307-5 (2016).

39 Kiryu, H. *et al.* A detailed investigation of accessibilities around target sites of siRNAs and miRNAs. *Bioinformatics* **27**, 1788-1797, doi:10.1093/bioinformatics/btr276 (2011).

40 Busch, A., Richter, A. S. & Backofen, R. IntaRNA: efficient prediction of bacterial sRNA targets incorporating target site accessibility and seed regions. *Bioinformatics* **24**, 2849-2856, doi:10.1093/bioinformatics/btn544 (2008).

41 Kato, Y. *et al.* RactIP: fast and accurate prediction of RNA-RNA interaction using integer programming. *Bioinformatics* **26**, i460-466, doi:10.1093/bioinformatics/btq372 (2010).

42 Helpap, B., Kollermann, J. & Oehler, U. Neuroendocrine differentiation in prostatic carcinomas: histogenesis, biology, clinical relevance, and future therapeutical perspectives. *Urologia internationalis* **62**, 133-138, doi:30376 (1999).

43 Hirano, D., Okada, Y., Minei, S., Takimoto, Y. & Nemoto, N. Neuroendocrine differentiation in hormone refractory prostate cancer following androgen deprivation therapy. *European urology* **45**, 586-592; discussion 592, doi:10.1016/j.eururo.2003.11.032 (2004).

44 Berruti, A. *et al.* Chromogranin A expression in patients with hormone naive prostate cancer predicts the development of hormone refractory disease. *The Journal of urology* **178**, 838-843; quiz 1129, doi:10.1016/j.juro.2007.05.018 (2007).

45 Aggarwal, R., Zhang, T., Small, E. J. & Armstrong, A. J. Neuroendocrine prostate cancer: subtypes, biology, and clinical outcomes. *Journal of the National Comprehensive Cancer Network : JNCCN* **12**, 719-726 (2014).

46 Yuan, T. C., Veeramani, S. & Lin, M. F. Neuroendocrine-like prostate cancer cells: neuroendocrine transdifferentiation of prostate adenocarcinoma cells. *Endocrine-related cancer* **14**, 531-547, doi:10.1677/ERC-07-0061 (2007).

47 Terry, S. *et al.* Cross modulation between the androgen receptor axis and protocadherin-PC in mediating neuroendocrine transdifferentiation and therapeutic resistance of prostate cancer. *Neoplasia* **15**, 761-772 (2013).

48 Huss, W. J., Gregory, C. W. & Smith, G. J. Neuroendocrine cell differentiation in the CWR22 human prostate cancer xenograft: association with tumor cell proliferation prior to recurrence. *The Prostate* **60**, 91-97, doi:10.1002/pros.20032 (2004).

49 Vashchenko, N. & Abrahamsson, P. A. Neuroendocrine differentiation in prostate cancer: implications for new treatment modalities. *European urology* **47**, 147-155, doi:10.1016/j.eururo.2004.09.007 (2005).

50 Aparicio, A. & Tzelepi, V. Neuroendocrine (small-cell) carcinomas: why they teach us essential lessons about prostate cancer. *Oncology* **28**, 831-838 (2014).

51 Beltran, H. *et al.* Aggressive variants of castration-resistant prostate cancer. *Clinical cancer research : an official journal of the American Association for Cancer Research* **20**, 2846-2850, doi:10.1158/1078-0432.CCR-13-3309 (2014).

52 Bishop, J. L., Davies, A., Ketola, K. & Zoubeidi, A. Regulation of tumor cell plasticity by the androgen receptor in prostate cancer. *Endocrine-related cancer* **22**, R165-182, doi:10.1530/ERC-15-0137 (2015).

53 Huarte, M. The emerging role of lncRNAs in cancer. *Nature medicine* **21**, 1253-1261, doi:10.1038/nm.3981 (2015).

54 Szafranski, P. *et al.* Small noncoding differentially methylated copy-number variants, including lncRNA genes, cause a lethal lung developmental disorder. *Genome research* **23**, 23-33, doi:10.1101/gr.141887.112 (2013).

55 White, N. M. *et al.* Transcriptome sequencing reveals altered long intergenic non-coding RNAs in lung cancer. *Genome biology* **15**, 429, doi:10.1186/s13059-014-0429-8 (2014).

56 Schuettengruber, B., Chourrout, D., Vervoort, M., Leblanc, B. & Cavalli, G. Genome regulation by polycomb and trithorax proteins. *Cell* **128**, 735-745, doi:10.1016/j.cell.2007.02.009 (2007).

57 Khalil, A. M. *et al.* Many human large intergenic noncoding RNAs associate with chromatin-modifying complexes and affect gene expression. *Proceedings of the National Academy of Sciences of the United States of America* **106**, 11667-11672, doi:10.1073/pnas.0904715106 (2009).

58 Rinn, J. L. *et al.* Functional demarcation of active and silent chromatin domains in human HOX loci by noncoding RNAs. *Cell* **129**, 1311-1323, doi:10.1016/j.cell.2007.05.022 (2007).
59 Xu, T. P. *et al.* Decreased expression of the long non-coding RNA FENDRR is associated with poor prognosis in gastric cancer and FENDRR regulates gastric cancer cell metastasis by affecting fibronectin1 expression. *Journal of hematology & oncology* **7**, 63, doi:10.1186/s13045-014-0063-7 (2014).
60 Li, J. *et al.* TANRIC: An Interactive Open Platform to Explore the Function of lncRNAs in Cancer. *Cancer research* **75**, 3728-3737, doi:10.1158/0008-5472.CAN-15-0273 (2015).
61 Tamura, M. *et al.* Forkhead transcription factor FOXF1 is a novel target gene of the p53 family and regulates cancer cell migration and invasiveness. *Oncogene* **33**, 4837-4846, doi:10.1038/onc.2013.427 (2014).
62 Tsuta, K., Wistuba, II & Moran, C. A. Differential expression of somatostatin receptors 1-5 in neuroendocrine carcinoma of the lung. *Pathology, research and practice* **208**, 470-474, doi:10.1016/j.prp.2012.05.014 (2012).
63 Pisarek, H., Pawlikowski, M., Kunert-Radek, J., Kubiak, R. & Winczyk, K. SSTR1 and SSTR5 subtypes are the dominant forms of somatostatin receptor in neuroendocrine tumors. *Folia histochemica et cytobiologica* **48**, 142-147, doi:10.2478/v10042-008-0103-7 (2010).
64 Muscarella, L. A. *et al.* Gene expression of somatostatin receptor subtypes SSTR2a, SSTR3 and SSTR5 in peripheral blood of neuroendocrine lung cancer affected patients. *Cellular oncology* **34**, 435-441, doi:10.1007/s13402-011-0025-9 (2011).
65 Squires, M. H., 3rd *et al.* Octreoscan Versus FDG-PET for Neuroendocrine Tumor Staging: A Biological Approach. *Annals of surgical oncology* **22**, 2295-2301, doi:10.1245/s10434-015-4471-x (2015).
66 Narayanan, S. & Kunz, P. L. Role of Somatostatin Analogues in the Treatment of Neuroendocrine Tumors. *Hematology/oncology clinics of North America* **30**, 163-177, doi:10.1016/j.hoc.2015.09.008 (2016).
67 Sharma, K., Patel, Y. C. & Srikant, C. B. C-terminal region of human somatostatin receptor 5 is required for induction of Rb and G1 cell cycle arrest. *Molecular endocrinology* **13**, 82-90, doi:10.1210/mend.13.1.0220 (1999).
68 Yang, J. *et al.* The role of histone demethylase KDM4B in Myc signaling in neuroblastoma. *Journal of the National Cancer Institute* **107**, djv080, doi:10.1093/jnci/djv080 (2015).
69 Sekaric, P., Shamanin, V. A., Luo, J. & Androphy, E. J. hAda3 regulates p14ARF-induced p53 acetylation and senescence. *Oncogene* **26**, 6261-6268, doi:10.1038/sj.onc.1210462 (2007).
70 Wang, T. *et al.* hADA3 is required for p53 activity. *The EMBO journal* **20**, 6404-6413, doi:10.1093/emboj/20.22.6404 (2001).
71 Tsai, H. *et al.* Cyclin D1 Loss Distinguishes Prostatic Small-Cell Carcinoma from Most Prostatic Adenocarcinomas. *Clinical cancer research : an official journal of the American Association for Cancer Research* **21**, 5619-5629, doi:10.1158/1078-0432.CCR-15-0744 (2015).
72 Piccolo, S. R. *et al.* A single-sample microarray normalization method to facilitate personalized-medicine workflows. *Genomics* **100**, 337-344, doi:10.1016/j.ygeno.2012.08.003 (2012).
73 Lapuk, A. V. *et al.* From sequence to molecular pathology, and a mechanism driving the neuroendocrine phenotype in prostate cancer. *The Journal of pathology* **227**, 286-297, doi:10.1002/path.4047 (2012).
74 Lai, D., Proctor, J. R., Zhu, J. Y. & Meyer, I. M. R-CHIE: a web server and R package for visualizing RNA secondary structures. *Nucleic acids research* **40**, e95, doi:10.1093/nar/gks241 (2012).
75 Mathews, D. H. *et al.* Incorporating chemical modification constraints into a dynamic programming algorithm for prediction of RNA secondary structure. *Proceedings of the National*

*Academy of Sciences of the United States of America* **101**, 7287-7292, doi:10.1073/pnas.0401799101 (2004).

76 Gruber, A. R., Lorenz, R., Bernhart, S. H., Neubock, R. & Hofacker, I. L. The Vienna RNA websuite. *Nucleic acids research* **36**, W70-74, doi:10.1093/nar/gkn188 (2008).

Table 1A

Click here to download Table T1A.pdf

| Name | Model System | Model System Name | Source | Phenotype | Resistance | | | | MOLECULAR CHARACTERICS | | | | | | | |
|---|---|---|---|---|---|---|---|---|---|---|---|---|---|---|---|---|
| | | | | | AN | TE | EZ | BI | AR | PSA | SYP | SPINK1 | ERG | TMPRSS2-ERG | PTEN | PTEN GENE |
| LTL313B | Xenograft | 313 | Primary PCa | AD | - | - | - | - | + | + | - | - | + | + | - | -/- |
| LTL313BR | Xenograft | 313 | LTL313B | CRPC | - | + | + | + | + | + | - | - | + | + | - | -/- |
| LTL418B | Xenograft | 418 | Primary PCa | AD | - | - | - | - | + | + | - | + | - | - | + | +/+ |
| LTL418BR | Xenograft | 418 | LTL418B | CRPC | - | + | - | - | + | + | - | | | | | |
| LTL331-3 | Xenograft | 331 | Primary PCa | AD | - | - | - | - | + | + | - | - | + | + | - | -/- |
| LTL331-7 | Xenograft | 331 | Primary PCa | AD | - | - | - | - | + | + | - | - | + | + | - | -/- |
| LTL331-5-8week | Xenograft | 331 | LTL331-5 | AD | - | - | - | - | + | - | - | | | | | |
| LTL331-5-12week | Xenograft | 331 | LTL331-5 | AD | - | - | - | - | + | - | - | | | | | |
| LTL331-3-R | Xenograft | 331 | LTL331-3 | NEPC | - | + | - | - | - | - | + | - | - | + | - | -/- |
| LTL331-7-R | Xenograft | 331 | LTL313-3-R | NEPC | - | + | - | - | - | - | + | - | - | + | - | -/- |

**LEGEND**

| | |
|---|---|
| AD | Adenocarcinoma |
| CRPC | Castration resistant prostate cancer |
| NEPC | Neuroendocrine prostate cancer |
| AN | Grows in the absense of Androgen |
| TE | Grows in the absense of Supplemented Testosterone |
| EZ | Resistant to Enzalutamide |
| BI | Resistant to Bicalutamide |
| (hatched) | Unknown |

Table 1B Click here to download Table T1B.pdf

| | | | | Treatment Status | | | | Gleason Grade | | | Clinical Charertistics End Points (For GRID Data Only) | | | | |
|---|---|---|---|---|---|---|---|---|---|---|---|---|---|---|---|
| Institute | Cohort Name | Clinical Group | TOTAL | NAIVE | NHT | ADT | RT | -6 | 7 | 8+ | BCR | MET | PCSM | +RMET+ADT | -RMET+ADT |
| VPC | VPC | AD-NAIVE | 56 | 56 | 0 | 0 | 0 | 23 | 0 | 33 | | | | | |
| VPC | VPC | AD-NHT | 14 | 0 | 14 | 0 | 0 | 0 | 0 | 14 | | | | | |
| VPC | VPC | NEPC[1] | 5 | 0 | 1 | 5 | 0 | 0 | 0 | 5 | | | | | |
| VPC | VPC | CRPC | 5 | 3 | 2 | 5 | 0 | 1 | 1 | 3 | | | | | |
| WCM | RUBIN | NEPC | 7 | | | | | NA | NA | NA | | | | | |
| WCM | RUBIN | AD | 30 | | | | | 2 | 23 | 5 | | | | | |
| JHSM | LOTAN | AD[2] | 17 | | | | | 0 | 0 | 12 | | | | | |
| JHSM | LOTAN | NEPC[2] | 16 | | | | | NA | NA | NA | | | | | |
| GRID | MCI | AD | 545 | 0 | 0 | 124 | 54 | 63 | 271 | 211 | 388 | 212 | 132 | 11 | 113 |
| GRID | MCII | AD | 232 | 0 | 0 | 77 | 24 | 18 | 117 | 97 | 124 | 75 | 34 | 19 | 24 |
| | | **TOTAL** | **927** | **59** | **17** | **211** | **78** | **107** | **412** | **380** | **512** | **287** | **166** | **30** | **137** |

**LEGEND**

| | |
|---|---|
| AD | Adenocarcinoma |
| CRPC | Castration resistant prostate cancer |
| NEPC | Neuroendocrine prostate cancer |
| NHT | Neoadjuvant Treatment |
| NAIVE | Naive Treatment |
| ADT | Androgen deprivation therapy |
| BCR | Biochemical recurrance |
| MET | Metastasis |
| PCSM | Prostate cancer specific mortality |
| +RMET+ADT | ADT treated rapid metastasis with at least 10 years of clinical followup |
| -RMET+ADT | ADT treated non-rapid metastasis with at least 10 years of clinical followup |
| 1 | Patient Overlaps Exist |
| 2 | Contains a subset of mixed histology tumours (see methods for breakdown) |
| | Unknown |

Figure 1

Click here to download Figure F1.pdf

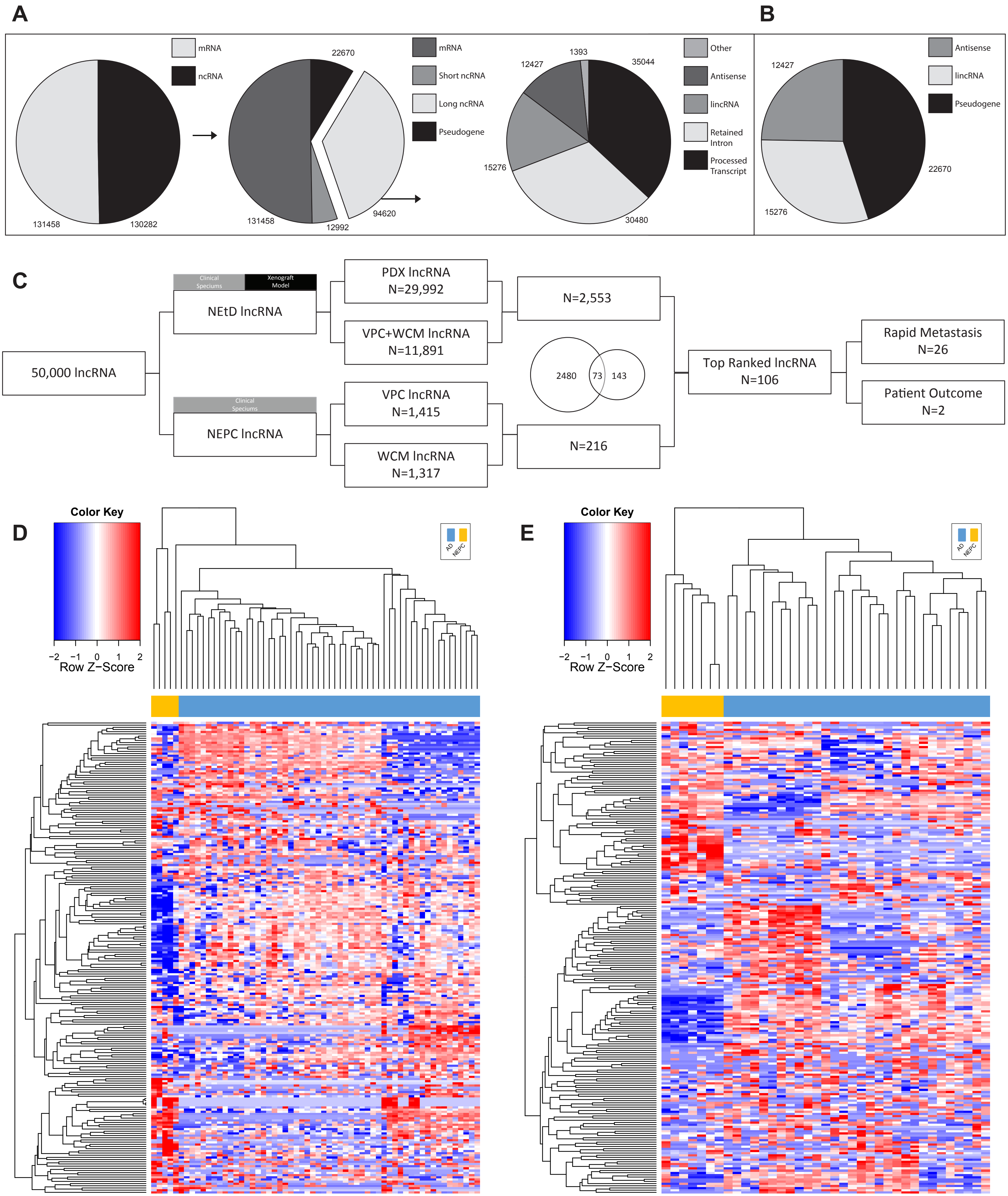

Figure 2

Click here to download Figure F2.pdf

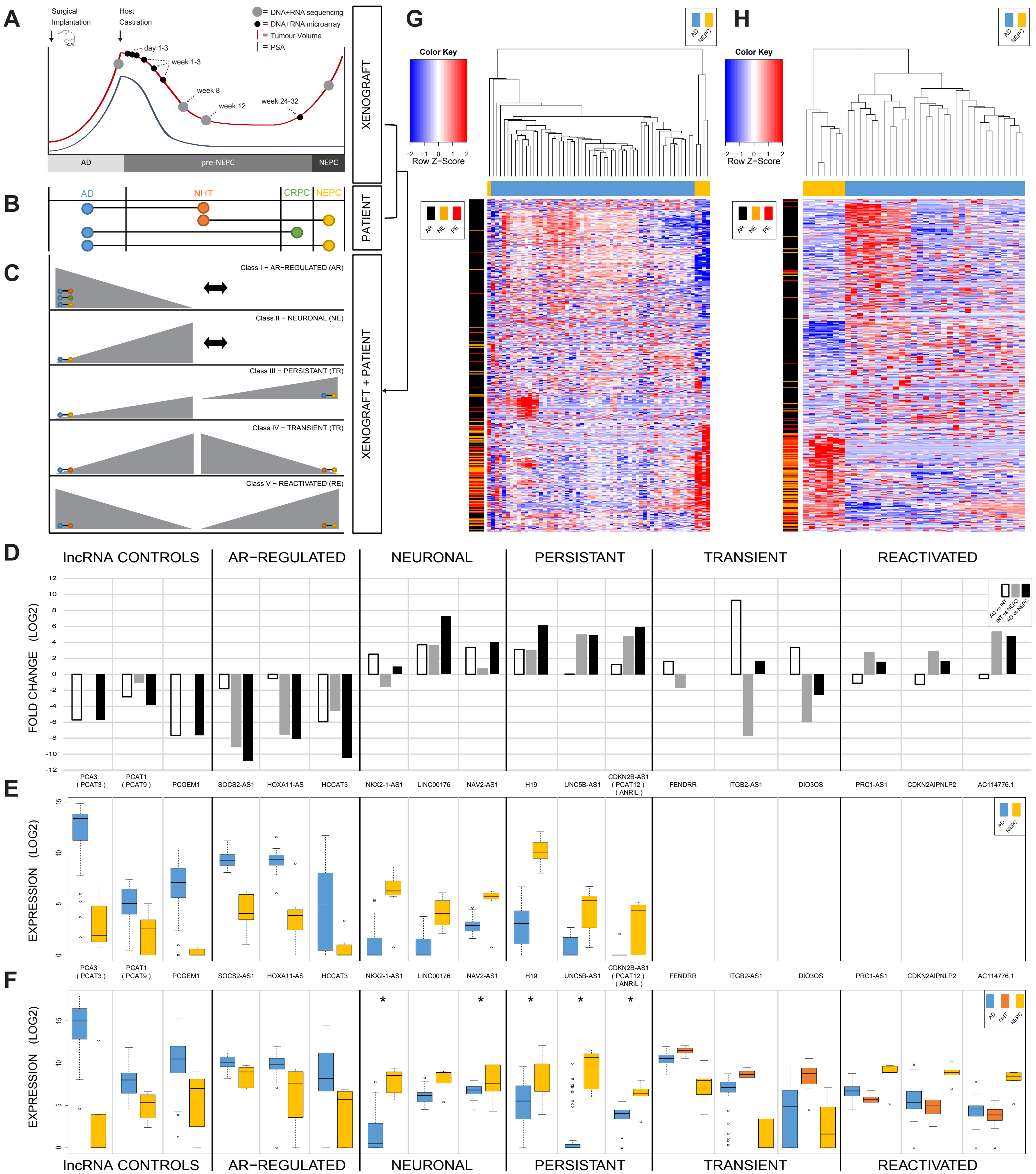

Figure 3 Click here to download Figure F3.pdf

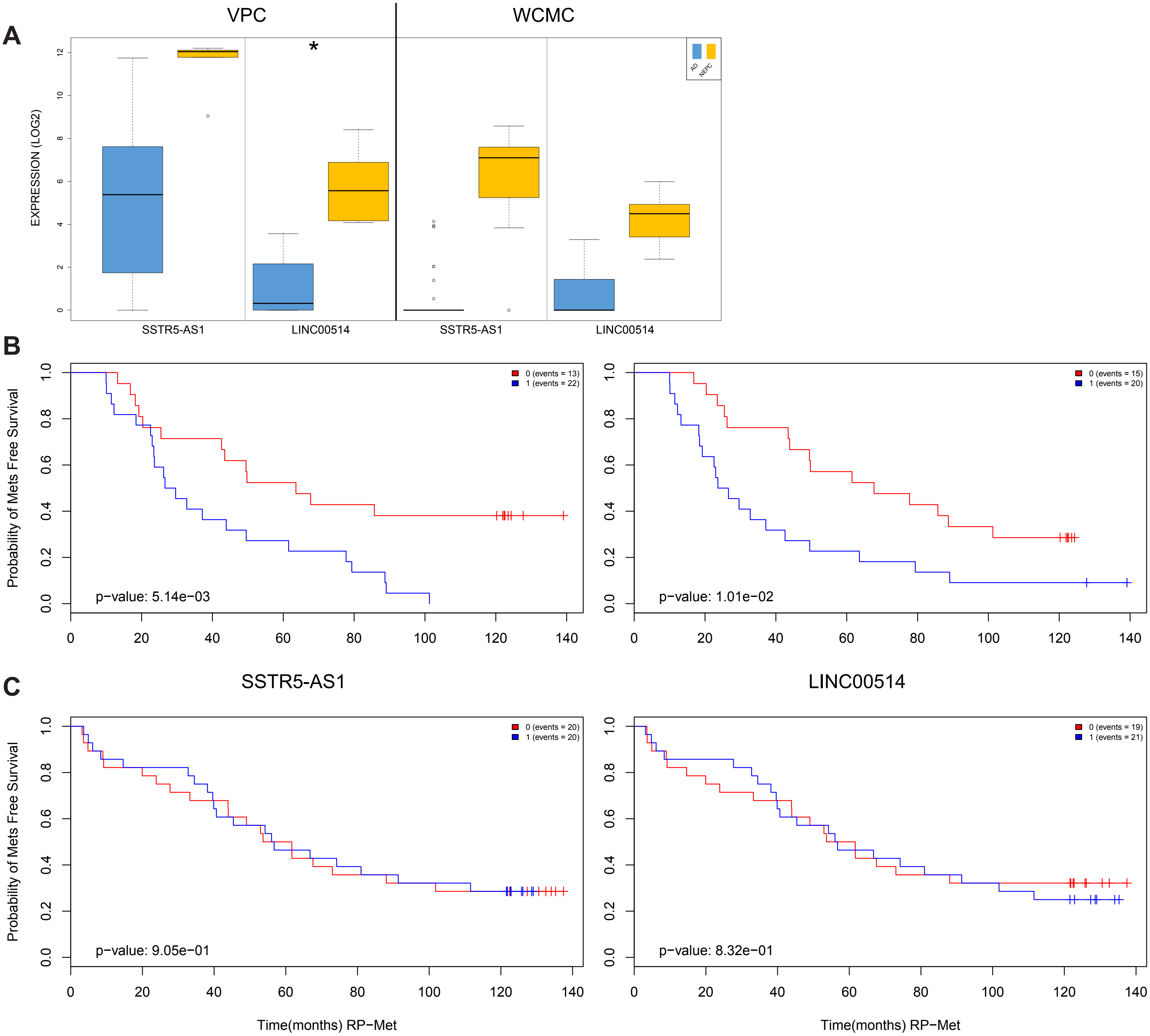

Supplementary Figure 1

Click here to access/download
**Supplementary Material**
SF1.pdf

Supplementary Figure 2

Click here to access/download
**Supplementary Material**
SF2.pdf

Supplementary Figure 3

Click here to access/download
**Supplementary Material**
SF3.pdf

Supplementary Figure 4

Click here to access/download
**Supplementary Material**
SF4.pdf

Supplementary Figure 5

Click here to access/download
**Supplementary Material**
SF5.pdf

Supplementary Figure 6

Click here to access/download
**Supplementary Material**
SF6.pdf

Supplementary Figure 7

Click here to access/download
**Supplementary Material**
SF7.pdf

Supplementary Figure 8

Click here to access/download
**Supplementary Material**
SF8.pdf

Supplementary Table 1-10

Click here to access/download
**Supplementary Material**
Supplementary Tables.xlsx

Personal Cover

Click here to download Personal Cover NEPC lncRNA - Cover Letter - 2017.04.28.docx

VANCOUVER PROSTATE CENTRE
A UBC & VGH Centre of Excellence

2660 Oak Street
Vancouver, BC
Canada V6H 3Z6

Phone: 604 875 4818
Fax: 604 875 5654
www.prostatecentre.com

Colin Collins, PhD
Professor, Department of Urologic Sciences
Senior Scientist, Vancouver Prostate Centre
University of British Columbia
ccollins@prostatecentre.com
Cell: 604-779-9287

Laurie Goodman
GigaScience

April 28th 2017

Dear Dr. Goodman,

I am pleased to submit our manuscript entitled "The Long Non-coding RNA Landscape of Neuroendocrine Prostate Cancer and their Clinical Implications" for your consideration as a Research Article for GigaScience.

In our paper we describe the first long non-coding RNA (lncRNA) genome-wide profiling of our neuroendocrine xenograft model, critical to understanding disease progression. We introduced this model in a previous study identifying the protein coding genes PEG10 and SRRM4 as novel oncogenes involved in the transdifferentiation of adenocarcinoma (NEtD) to treatment induced neuroendocrine prostate cancer (NEPC). We believe that the transdifferentiation process is complex and likely involves a number of interacting layers of epigenetic and genetic aberrations. One of those layers we focus on with this study, characterizing the unexplored lncRNA landscape throughout NEtD.

Identifying lncRNA aberrations and their cellular implications have begun to fill the holes in our understanding of tumorigenesis and thus have emerged as crucial players in cancer progression and metastasis. In our study, implementing an in-house sequence analysis pipeline capable of detecting 50,000 lncRNA, we identify 4,720 associated to NEPC. A subset of these has strong clinical or clinicopathological implications, including a robust 216-lncRNA signature present within three clinical NEPC cohorts, 2,553 that display five striking patterns of expression directly associated to NEtD, 26 that possess an association to rapid metastasis following androgen deprivation therapy (ADT), and finally our top two candidates that show significant increased likelihood to stratify patients undergoing ADT based on patient outcome.

We present here for the first time a catalog of NEtD lncRNA that we feel is best suited for the aims and scope that GigaScience encompasses. The selection of your journal was due to the foundation of our work, which surrounds genome-wide (quality-control driven) sequence analysis, novel methods for integrative transcriptomic analysis of xenograft models to clinical samples (largest study to date to preform this), and application of this methodology to provide insights into the lncRNA molecular and clinical aspects of a NEPC.

This study has not been published, nor is under consideration for publication elsewhere. All authors are fully aware of the content of this manuscript and agree with the content and its submission for publication. We will not discuss the study with the media or other journals.

Yours sincerely,

Colin Collins, PhD
