# Supplementary material for: The long noncoding RNA landscape of neuroendocrine prostate cancer and its clinical implications
Source: Gigascience. 2018 May 10;7(6):giy050. doi: 10.1093/gigascience/giy050 (PMC6007253; doi:10.1093/gigascience/giy050)
Supplement: GIGA-D-17-00096_Revision_3.pdf [file giy050_giga-d-17-00096_revision_3.pdf]

GigaScience

The Long Noncoding RNA Landscape of Neuroendocrine Prostate Cancer and its Clinical Implications

--Manuscript Draft--

**Manuscript Number:**	GIGA-D-17-00096R3
**Full Title:**	The Long Noncoding RNA Landscape of Neuroendocrine Prostate Cancer and its Clinical Implications
**Article Type:**	Research
**Funding Information:**	Mitacs (CA) Accelerate PhD Fellowship Program (IT04310) — Mr Varune Rohan Ramnarine<br>Terry Fox Foundation (201012TFF) — Dr Colin C Collins<br>Prostate Cancer Team Grant (T2013-01) — Dr Colin C Collins

Abstract:

Background
Treatment induced neuroendocrine prostate cancer (tNEPC) is an aggressive variant of late-stage metastatic castrate resistant (mCRPC) prostate cancer that commonly arises through neuroendocrine transdifferentiation (NEtD). Treatment options are limited, ineffective, and for most patients, results in death in less than a year. We previously developed a first-in-field patient-derived xenograft (PDX) model of NEtD. Longitudinal deep transcriptome profiling of this model enabled monitoring of dynamic transcriptional changes during NEtD and in the context of androgen deprivation. Long non-coding RNA (lncRNA) are implicated in cancer where they can control gene regulation. Until now the expression of lncRNAs during NEtD and their clinical associations were unexplored.

Results
We implemented a next-generation sequence analysis pipeline that can detect transcripts at low expression levels and built a genome-wide catalogue (n=37,749) of lncRNAs. We applied this pipeline to 927 clinical samples and our high fidelity NEtD model LTL331 and identified 821 lncRNAs in NEPC. Among these are 122 lncRNAs that robustly distinguish NEPC from prostate adenocarcinoma (AD) patient tumours. The highest expressed lncRNAs within this signature are H19, LINC00617, and SSTR5-AS1. Another 742 are associated with the NEtD process and fall into four distinct patterns of expression (NEtD lncRNA Class I, II, III, and IV) in our PDX model and clinical samples. Each class has significant (z-scores>2) and unique enrichment for transcription factor binding site (TFBS) motifs in their sequences. Enriched TFBS include (1) TP53 and BRN1 in Class I, (2) ELF5, SPIC, and HOXD1 in Class II, (3) SPDEF in Class III, (4) HSF1 and FOXA1 in Class IV, and (5) TWIST1 when merging Class III with IV. Common TFBS in all NEtD lncRNA were also identified and include, E2F, REST, PAX5, PAX9, and STAF. Interrogation of the top deregulated candidates (n=100) in radical prostatectomy adenocarcinoma samples with long-term follow-up (median 18 years) revealed significant clinicopathological associations. Specifically, we identified 25 that are associated with rapid metastasis following androgen deprivation therapy (ADT). Two of these lncRNAs (SSTR5-AS1 and LINC00514) stratified patients undergoing ADT based on patient outcome.

Discussion
To date, a comprehensive characterization of the dynamic landscape of lncRNAs during the NEtD process has not been performed. A temporal analysis of the PDX-based NEtD model has for the first time provided this dynamic landscape. TFBS analysis identified NEPC-related TF motifs present within the NEtD lncRNA sequences, suggesting functional roles for these lncRNAs in NEPC pathogenesis. Furthermore, select NEtD lncRNAs appear to be associated with metastasis and patients receiving ADT. Treatment-related metastasis is a clinical consequence of NEPC tumours. Top candidate lncRNAs FENDRR, H19, LINC00514, LINC00617, and SSTR5-AS1 identified in this study are implicated in the development of NEPC. We present here for the first time a genome-wide catalogue of NEtD lncRNAs that

Powered by Editorial Manager® and ProduXion Manager® from Aries Systems Corporation

	characterize the transdifferentiation process and a robust NEPC lncRNA patient expression signature. To accomplish this, we carried out the largest integrative study that applied a PDX NEtD model to clinical samples. These NEtD and NEPC lncRNAs are strong candidates for clinical biomarkers and therapeutic targets and warrant further investigation.
**Corresponding Author:**	Varune Rohan Ramnarine<br><br>CANADA
**Corresponding Author Secondary Information:**	
**Corresponding Author's Institution:**	
**Corresponding Author's Secondary Institution:**	
**First Author:**	Varune Rohan Ramnarine
**First Author Secondary Information:**	
**Order of Authors:**	Varune Rohan Ramnarine
	Mohammed Alshalalfa
	Fan Mo
	Noushin Nabavi
	Nicholas Erho
	Mandeep Takhar
	Robert Shukin
	Sonal Brahmbhatt
	Alexander Gawronski
	Maxim Kobelev
	Mannan Nouri
	Dong Lin
	Harrison Tsai
	Tamara L Lotan
	R. Jefferey Karnes
	Mark A Rubin
	Amina Zoubeidi
	Martin E Gleave
	Cenk Sahinalp
	Alexander W Wyatt
	Stanislav V Volik
	Himisha Beltran
	Elai Davicioni
	Yuzhuo Wang
	Colin C Collins
**Order of Authors Secondary Information:**	
**Response to Reviewers:**	Hello Dr. Edmunds,<br>All data issues have now been resolved. Please see attached for all final versions of

	data and our manuscript.<br>Regards<br>Rohan
**Additional Information:**	
**Question**	**Response**
Are you submitting this manuscript to a special series or article collection?	No
**Experimental design and statistics**<br><br>Full details of the experimental design and statistical methods used should be given in the Methods section, as detailed in our Minimum Standards Reporting Checklist. Information essential to interpreting the data presented should be made available in the figure legends.<br><br>Have you included all the information requested in your manuscript?	Yes
**Resources**<br><br>A description of all resources used, including antibodies, cell lines, animals and software tools, with enough information to allow them to be uniquely identified, should be included in the Methods section. Authors are strongly encouraged to cite Research Resource Identifiers (RRIDs) for antibodies, model organisms and tools, where possible.<br><br>Have you included the information requested as detailed in our Minimum Standards Reporting Checklist?	Yes
**Availability of data and materials**<br><br>All datasets and code on which the conclusions of the paper rely must be either included in your submission or deposited in publicly available repositories (where available and ethically appropriate), referencing such data using a unique identifier in the references and in the "Availability of Data and Materials" section of your manuscript.<br><br>Have you have met the above requirement as detailed in our Minimum Standards Reporting Checklist?	Yes

Manuscript
Click here to download Manuscript NEPC lncRNA - v5.18 - 2018.04.19.docx

The Long Noncoding RNA Landscape of Neuroendocrine Prostate Cancer and its Clinical Implications

Varune Rohan Ramnarine[1], Mohammed Alshalalfa[2], Fan Mo[1], Noushin Nabavi[1], Nicholas Erho[2], Mandeep Takhar[2], Robert Shukin[1], Sonal Brahmbhatt[1], Alexander Gawronski[3], Maxim Kobelev[1], Mannan Nouri[1], Dong Lin[1,4], Harrison Tsai[5], Tamara L Lotan[5], R. Jefferey Karnes[6], Mark A Rubin[7], Amina Zoubeidi[1], Martin E Gleave[1], Cenk Sahinalp[1,8], Alexander W Wyatt[1], Stanislav V Volik[1], Himisha Beltran[9], Elai Davicioni[2], Yuzhuo Wang[1,4], and Colin C Collins[1]

[1]Vancouver Prostate Centre & Department of Urologic Sciences, University of British Columbia, Vancouver, BC, Canada; [2]GenomeDx Biosciences Inc., Vancouver, BC, Canada; [3]Department of Computer Science, Simon Fraser University, Burnaby, BC, Canada; [4]Department of Experimental Therapeutics, BC Cancer Agency, Vancouver, BC, Canada; [5]Department of Pathology, Johns Hopkins School of Medicine, Baltimore, MD, USA; [6]Department of Urology, Mayo Clinic College of Medicine, Rochester, MN, USA; [7]Department of Pathology and Laboratory Medicine, Weill Cornell Cancer Center, Weill Cornell Medical College, New York, NY, USA; [8]Department of Computer Science, Indiana University, Bloomington, USA; [9]Department of Medicine, Weill Cornell Cancer Center, Weill Cornell Medical College, New York, NY, USA

Running title: NEPC lncRNAs
Keywords: neuroendocrine prostate cancer, transdifferentiation, small cell carcinoma, long non-coding RNA
Total number of figures and tables: 6 figures and 2 tables
Total number of supplementary figures and tables: 14 figures and 29 tables

ABSTRACT

Background

Treatment induced neuroendocrine prostate cancer (tNEPC) is an aggressive variant of late-stage metastatic castrate resistant (mCRPC) prostate cancer that commonly arises through neuroendocrine transdifferentiation (NEtD). Treatment options are limited, ineffective, and for most patients, results in death in less than a year. We previously developed a first-in-field patient-derived xenograft (PDX) model of NEtD. Longitudinal deep transcriptome profiling of this model enabled monitoring of dynamic transcriptional changes during NEtD and in the context of androgen deprivation. Long non-coding RNA (lncRNA) are implicated in cancer where they can control gene regulation. Until now the expression of lncRNAs during NEtD and their clinical associations were unexplored.

Results

We implemented a next-generation sequence analysis pipeline that can detect transcripts at low expression levels and built a genome-wide catalogue (n=37,749) of lncRNAs. We applied this pipeline to 927 clinical samples and our high fidelity NEtD model LTL331 and identified 821 lncRNAs in NEPC. Among these are 122 lncRNAs that robustly distinguish NEPC from prostate adenocarcinoma (AD) patient tumours. The highest expressed lncRNAs within this signature are H19, LINC00617, and SSTR5-AS1. Another 742 are associated with the NEtD process and fall into four distinct patterns of expression (NEtD lncRNA Class I, II, III, and IV) in our PDX model and clinical samples. Each class has significant (z-scores>2) and unique enrichment for transcription factor binding site (TFBS) motifs in their sequences. Enriched TFBS include (1) TP53 and BRN1 in Class I, (2) ELF5, SPIC, and HOXD1 in Class II, (3) SPDEF in Class III, (4) HSF1 and FOXA1 in Class IV, and (5) TWIST1 when merging Class III with IV. Common TFBS in all NEtD

lncRNA were also identified and include, E2F, REST, PAX5, PAX9, and STAF. Interrogation of the top deregulated candidates (n=100) in radical prostatectomy adenocarcinoma samples with long-term follow-up (median 18 years) revealed significant clinicopathological associations. Specifically, we identified 25 that are associated with rapid metastasis following androgen deprivation therapy (ADT). Two of these lncRNAs (SSTR5-AS1 and LINC00514) stratified patients undergoing ADT based on patient outcome.

Discussion

To date, a comprehensive characterization of the dynamic landscape of lncRNAs during the NEtD process has not been performed. A temporal analysis of the PDX-based NEtD model has for the first time provided this dynamic landscape. TFBS analysis identified NEPC-related TF motifs present within the NEtD lncRNA sequences, suggesting functional roles for these lncRNAs in NEPC pathogenesis. Furthermore, select NEtD lncRNAs appear to be associated with metastasis and patients receiving ADT. Treatment-related metastasis is a clinical consequence of NEPC tumours. Top candidate lncRNAs FENDRR, H19, LINC00514, LINC00617, and SSTR5-AS1 identified in this study are implicated in the development of NEPC. We present here for the first time a genome-wide catalogue of NEtD lncRNAs that characterize the transdifferentiation process and a robust NEPC lncRNA patient expression signature. To accomplish this, we carried out the largest integrative study that applied a PDX NEtD model to clinical samples. These NEtD and NEPC lncRNAs are strong candidates for clinical biomarkers and therapeutic targets and warrant further investigation.

INTRODUCTION

Prostate cancer (PCa) is the most common cancer affecting men and is third highest cause of cancer death in developed countries globally[1]. Advances in detection and treatment for PCa have translated to many men being successfully treated by surgery and/or radiation. Concomitantly, androgen deprivation therapy (ADT) has resulted in significant survival gains for men with metastatic PCa. Commonly administered therapeutics include Enzalutamide, Bicalutamide, and Abiraterone[2]. These drugs inhibit the androgen signaling axis, a growth and differentiation-inducing pathway mediated by the androgen receptor (AR). Despite these successes, with the steady accumulation of facilitating genomic and epigenomic aberrations, a more aggressive tumour capable of growing in castrate levels of testosterone can develop[3] termed castration-resistant prostate cancer (CRPC). Three main classes of treatment resistance to AR-targeted therapies exist, falling into two broad categories associated to AR signaling[4]. The majority of CRPC reactivate the AR signaling axis (AR^+ CRPC). However, some tumour cells leverage their inherent plasticity and progress to an AR-negative state (AR^- CRPC), circumventing AR dependence. AR^- CRPC is highly heterogeneous, but a major established aggressive subtype is neuroendocrine prostate cancer (NEPC)[5]. NEPC is pathologically and clinically similar to small cell carcinoma of the prostate (SCPC), which has been defined as a distinct morphological subtype of PCa with neuroendocrine differentiation[6]. Xenograft NEPC models have shown expression of a dominant and irreversible neuronal-like phenotype[7] where conventional CRPC therapies are ineffective. Platinum-based chemotherapy is only transiently effective, resulting in poor overall survival[8] with most patients surviving ~7 months[9]. Molecular pathology markers include expression of chromogranin A (CHGA), synaptophysin (SYP), neuro-specific enolase (NSE)[10], cell-surface marker CEACAM5[11], and negative (or low) levels of AR and AR-regulated genes such as PSA[7]. NEPC can arise *de novo* but much more commonly occurs as a consequence of ADT via an adaptive process termed neuroendocrine transdifferentiation (NEtD)[7,12] and frequently metastasizes to visceral organs[13]. Predisposing aberrations for NEtD include loss of RB1[14], TP53[15], mutation of Trp53[16] and/or PTEN inactivation[17,18]. Emerging data suggest drivers include splice factor SRRM4[19-21], master neural transcription factor BRN2[22], and FOXA1[23]. NEPC tumours have been characterized with (1) gains in MYCN and AURKA[5], (2) overexpression of PEG10[24], HP1α[25], N-Myc[26,27], SOX2[28], and SOX11[18], (3) downregulation of PHF8, KDM3A[29,30], REST[31], and SPEDF[32], and lastly (4) disease dependency on GPX4[33]. Discoveries such

as these continue to define the protein-coding transcriptome of NEPC. The process of transdifferentiation however, is highly complex and likely involves multiple layers of genetic and epigenetic regulation.

Dysregulation of long non-coding RNAs (lncRNAs) could provide an additional mechanism for the gene expression alterations that occur during NEtD. LncRNAs are broadly defined as large (>200 nucleotides/nt) RNA transcripts, with the most abundant subtypes classified as antisense RNAs, pseudogenes, and long intergenic noncoding RNAs (lincRNA)[34]. They are implicated in a variety of diseases, and their association with cancer progression is reported through mechanisms such as remodeling of chromatin, transcriptional co-activation or repression, modulation of protein activity, post-transcriptional regulation, or as decoy elements[35-37]. LncRNAs form an important regulatory layer in global gene expression and as such, alterations of lncRNAs in cancer is identified as one of the driving forces for tumorigenesis[38,39], cancer progression, and metastasis[40,41]. More specifically in PCa, lncRNAs have been reported to play critical roles at every stage including, the transformation of normal prostate cells to prostate intraepithelial neoplastic (PIN) cells, the development of localized tumours, and finally progression to advanced metastatic disease[42]. These roles in initiation and progression are due to aberrant lncRNA expression, which changes the balance of protein-coding genes involved in processes such as proliferation and apoptosis, thereby facilitating cellular transformation.

We recently developed a first-in-field transplantable patient-derived xenograft (PDX) model of NEtD: a treatment-naïve adenocarcinoma (LTL331) that upon host castration initially regresses (LTL331-8 and 12 week), but then rapidly relapses as terminally differentiated NEPC (LTL331R)[7]. In our previous study using this model, we demonstrated a lack of evidence for NEPC cells before host castration and the conservation of genome characteristics pre- and post-castration strongly suggesting a phase transition or state change from adenocarcinoma to NEPC[24]. With this model we have identified protein-coding transcripts such as PEG10[24], SRRM4[19], and HP1α[25] that are active in the phase transition and validated the discovery of BRN2[22]. In addition to these, our model has led to the identification of potential biomarkers and therapeutic targets for NEPC, including the DEK proto-oncogene[43] and epigenetic regulators CBX2 and EZH2[44] (members of the polycomb group family of transcriptional repressors). We now report the comprehensive characterization of lncRNAs in our NEtD model. In the current study, we used the longitudinal genomic profiling of our PDX-based NEtD model focusing on lncRNA transcripts. We hypothesized that lncRNA expression across the 'time series' would associate with the development of NEPC. Our objective was to comprehensively characterize the dynamic lncRNA landscape of NEtD and NEPC, identify putative functional motifs within lncRNA sequences, determine the clinical relevance of lncRNA expression, and identify associated clinicopathological features. To accomplish this, we implemented a sequence analysis pipeline optimized for the detection of lncRNAs, identified a clinical signature that can robustly distinguish NEPC from AD tumours, and identified four NEtD associated lncRNA expression profiles. We also identified significant enrichment of well-known transcription factor motifs within the lncRNA sequences. Lastly, we observed that a subset of these lncRNAs are associated with rapid metastasis in treated patients and can stratify tumours based on patient outcome. We present here for the first time a comprehensive landscape of NEPC lncRNAs and their clinical associations.

RESULTS

Comprehensive catalogue of long non-coding RNAs in neuroendocrine prostate cancer

To identify lncRNAs involved in NEPC, we performed next-generation polyadenylated RNA sequencing on the PDX CRPC models and patient samples. We implemented a sequence analysis pipeline composed primarily of algorithms from the Tuxedo suite of analysis tools[45]. Typically, lncRNAs are expressed at low

levels, so the pipeline was augmented to include windowed-adaptive quality control corrections (see Methods and Supplementary Figure 1-2) that increase the ability to detect low abundance transcripts. We applied this pipeline to each PDX (n=10) and clinical specimens (n=117) acquired from the Vancouver Prostate Centre (VPC) and Weill Cornell Medicine (WCM) (Table 1-2). Using a *quasi de novo* mapping strategy combined with amalgamating all sample transcriptome assemblies, we identified 210,999 annotated transcripts spanning 38 Ensembl transcript classes. Defined by Ensembl's core biotypes, transcripts are classified as either protein-coding RNAs, long ncRNAs, short ncRNAs, or pseudogenes, which totaled 102,334 (48%), 82,846 (39%), 9,803 (5%) and 16,016 (8%), respectively (Figure 1A – Pie Chart 1-2). Within long ncRNA, seven subclasses exist; processed transcripts (n=31,142), retained intron (n=28,455), lincRNA (n=12,047), antisense (10,012), sense intronic (n=821), sense overlapping (n=340), and 3 prime overlapping ncRNA (n=29) (Figure 1A – Pie Chart 3 – Due to their small totals sense intronic, sense overlapping, and 3 prime overlapping ncRNAs are labeled as "Other"). Despite pseudogenes not being included within Ensembl's long ncRNA classes (listed above), they are by definition considered under the umbrella of lncRNA[34].

For each of the eight lncRNA subclasses and their corresponding transcripts, we performed unsupervised hierarchical clustering (UHC) and principle component analysis (PCA) on the VPC and WCM cohorts (see methods – statistical analysis). Five were incapable of distinctly separating NEPC and AD clinical samples due to insufficient transcript counts, incorrect transcript classification, or in general poor transcript annotation. The remaining three subclasses were capable of separating NEPC and AD (Supplementary Figure 3-4) and became the focus of all downstream analysis. These three lncRNA subclasses, antisense (n=10,012), pseudogenes (n=15,690), and lincRNAs (n=12,047) are collectively referred to as lncRNAs here on in (n=37,749 transcripts in total – Figure 1B). It should be noted that immunoglobulin and T cell receptor genes (n=326) were removed from the pseudogene transcript total. We explored these lncRNAs in our samples through two analytical workflows (model-based discovery and patient-based discovery), which we later merged for clinicopathological analysis. The outline presented in this figure represents the study's overall workflow (Figure 1C).

Long non-coding RNA expression profiles classify neuroendocrine prostate cancer

Recently it has been shown that AR$^-$ and AR$^+$ CRPC share substantial genomic overlap yet display significant epigenetic differences[32]. Here, we hypothesized that the lncRNA transcriptome would similarly show unique and common expression alterations between AR$^+$ and AR$^-$ CRPC (unexplored to date). To investigate this, we used the AR$^{+/-}$ CRPC xenograft models (Table 1) to identify changes occurring temporally within the same tumour pre- and post-castration. Once castrated the three AD models (LTL313, LTL418, and LTL331) progress to either AR$^+$ CRPC (LTL313BR and LTL418BR) or AR$^-$ CRPC/NEPC (LTL331R). This allowed for the identification and quantification of differentially expressed transcripts between pre- and post-CRPC. We integrated this data with patient tumour data having matched clinical information to ensure the results were clinically relevant and to remove any model-based bias. As we suspected, of all lncRNAs altered between pre- and post-CRPC (>2 fold, p-value<0.05), only 8% (n=300) were commonly deregulated in both CPRC subtypes. The remaining transcripts (n=2669) displayed unique changes in the AR$^+$ or AR$^-$ CRPC subtype (Supplementary Table 2 and Supplementary Figure 5). This data supports the notion that AR$^+$ and AR$^-$ CRPC contain largely distinct lncRNA landscapes.

LncRNA expression may be useful as additional biomarkers beyond those currently used in the diagnosis of NEPC (i.e., CGHA, SYP, and NSE). Moreover, a lncRNA expression signature would strongly support the involvement of lncRNAs in NEPC at a molecular and cellular level. These lncRNAs would be candidates for mechanisms in the activation of a developmental pathway and/or plasticity involving previously identified protein-coding genes (PEG10, HP1α, NMYC, SOX2, SRRM4, REST, BRN2, etc…) in NEPC/NEtD. Conversely,

since some of these genes (NMYC, SOX2, BRN2, and SRRM4) are well-studied transcription or splicing factors, NEPC lncRNA could be under their regulation. To build a lncRNA expression signature for NEPC, we selected the top 5 th percentile of transcripts based on standard deviations of expression for the VPC and WCM cohorts independently and performed UHC. All uncharacterized transcripts (i.e. RP##-######.#, AC######.#, etc.) were removed from the analysis at this point. This produced 265 and 490 NEPC lncRNAs in the VPC and WCM cohorts, respectively. Taking the intersection of these lists and then repeating UHC generated an expression signature of 122 lncRNAs (Supplementary Table 5) that distinctly segregated NEPC from AD tumours (Figure 2A-B). To assess the robustness of this signature, we validated it on an external clinical cohort of tumours (n=33 – Table 2) from Johns Hopkins School of Medicine (JHSM). These tumours contained 17 AD and 16 NEPC samples and were profiled on the Human Exon array 1.0 ST platform (see Methods) compared to the sequenced discovery cohorts. Using the same approach (UHC), a clear separation of NEPC and AD was observed (Figure 2C). Observing consistent results across different technologies, platforms, institutes, and clinical samples further strengthen the robust nature of the NEPC signature. To our knowledge, this is the first report of lncRNAs exhibiting a unique, unbiased expression profile capable of segregating NEPC and from AD patient samples.

Some lncRNA from the patient derived signature have been previously reported as altered in other cancer types. These lncRNAs include MALAT1 (alias NEAT2), PCA4 (aliases GDEP, PCAN1, or PCAT4), DSCAM-AS1, and SNHG12. MALAT1 is one of the most well characterized and studied lncRNAs in cancer and has been identified as a regulator of metastasis and cell migration, a prognostic marker, and a transcriptional regulator of alternative splicing in lung cancer [46]. PCA4 has been identified as a prostate and retinal specific transcript [47] and frequently mutated in PCa[48]. DSCAM-AS1 mediates tumour progression and tamoxifen resistance in breast cancer through interacting protein hnRNPL[49]. SNHG12 is induced by c-MYC and regulates cell proliferation, apoptosis, and migration in triple negative breast cancer[50]. We were interested in identifying the most highly expressed lncRNAs in the signature. Therefore we ranked each according to their fold changes when compared to AD samples, required concordance in fold changes across both of the cohorts, and >10-fold change in magnitude. H19, LINC00617 (alias TUNA/TUNAR in mouse), NKX2-1-AS1, and SSTR5-AS1 were the only four that fit these thresholds and each with previous reports in cancer. Of note, H19 is the most studied among the four lncRNAs and is implicated in numerous cancer types[51]. It is involved in proliferation and both differentiation processes related to metastasis, epithelial to mesenchymal transition (EMT) and mesenchymal to epithelial transition (MET)[52]. LINC00617 in breast cancer regulates EMT, cancer progression, and metastasis through activation of the transcription of SOX2[53]. SSTR5-AS1 has not been functionally characterized, but its sense form SSTR5 has and is a biomarker for neuroendocrine tumours[54]. In fact, recently it has been used to evaluate SSTR-targeted therapy for neuroendocrine tumours in circulating tumour cells[55] and its use in patient management is being tested in a Phase IV clinical trial (NCT02075606). Overall, the identification of the NEPC lncRNA expression signature has provided a previously unexplored component of the NEPC transcriptome, revealed candidate NEPC biomarkers, and associations to NEPC biology.

Long non-coding RNAs are associated with neuroendocrine transdifferentiation

A major goal of this study was to characterize the lncRNA landscape during the dynamic phase transition from adenocarcinoma to NEPC using our unique PDX model LTL331[7] (Figure 3A). To accomplish this, we sequenced six samples of our PDX NEtD model representing three primary time points along disease progression: two samples from each terminal point (AD and NEPC) and two samples post-castration (postTX). Time points 8- and 12-week post-castration were selected to represent postTX due to tumour volume and serum PSA levels reaching nadir (Table 1 and Figure 3A). We identified and quantified all lncRNA transcripts that were altered across the time series and defined four patterns of transcript expression: (a) continuous decline in expression (Class I – Deactivated, n=1,613); (b) increasing expression

from either AD to postTX or postTX to NEPC (Class II – Activated, n=4,281); (c) continuous increased expression (Class III – Persistent, n=1,054); and (d) maximum expression at postTX (Class IV – Transient, n=2,668); (Total n=7,627, Figure 3A). The NEtD model and postTX state represents a biological process that currently is not characterized as a clinical entity but offers invaluable insight into the transcriptome of transdifferentiating AD cells.

To determine the clinical relevance of Class (I – IV) lncRNAs, we integrated patient samples (VPC and WCM, Table 2 – Column "Clinical Group") with time points in our model (see Figure 3B for alignment of time points to patient groups). Terminal time points were appropriately aligned to AD and NEPC samples. Due to the lack of clinical specimens undergoing NEtD we hypothesized that neoadjuvant hormone therapy (NHT) given to AD patients might exhibit characteristics of the postTX state. The transcriptomes from these patients have been shown to display the effects of therapy response and more specifically androgen depletion[56]. In fact, neuroendocrine differentiation has been shown to increase after only three months of NHT in a retrospective analysis of 103 radical prostatectomy specimens[57]. These early events are the specific alterations we sought to isolate from the postTX time points of our PDX model. We also postulated that a subset of Class I (down-regulated in our PDX model) would be up-regulated in the (AR^+) CRPC clinical samples due to reactivation of the AR signalling axis in classical CRPC[56,58-60]. Based on this model-to-clinic data integration, the following patient group-wise comparisons were performed: (a) NEPC vs AD, (b) NEPC vs NHT, (c) CRPC vs AD, (d) NHT vs NAÏVE (untreated AD) and (e) NHT vs NAÏVE in combination with NEPC vs NHT. This produced 1,927, 713, 975, 1,045, and 117 transcripts, respectively (>2 fold with p-value < 0.05 – Total n=3,154, Figure 3B, Supplementary Table 4). Integrating these results with the PDX NEtD model transcripts above, led to 475, 222, 84, and 45 lncRNAs identified within Class I (Deactivated), Class II (Activated), Class III (Persistent), and Class IV (Transient), respectively (Total n=742, Figure 3C). Unsupervised hierarchical clustering of Class I-III within WCM (Figure 3D) and VPC (Figure 3E) cohorts exhibit (as expected) a distinct separation of AD and NEPC tumours and a distinct separation between lncRNAs in Class I-III (rows of heat map). Class IV transcripts were excluded from this illustration due to their lack of altered expression between AD and NEPC clinical samples. Collectively these 742 NEtD lncRNAs are associated with the pathogenesis of treatment-induced NEPC.

Prominent examples identified by this biological integration of our NEtD model (Figure 4A), WCM cohort (Figure 4B), and VPC cohort (Figure 4C) illustrate each of these NEtD defining transcript classes. PCA3, PCAT1, and PCGEM1 were selected as controls for this study due to their elevated expression in PCa and high level of characterization. As expected, their expression patterns followed the trend in the PCa and NEPC samples (Figure 4A-C – NEtD Controls, p-values<0.001). SOCS2-AS1 and HOXA11-AS are select examples that characterize the deactivated NEtD lncRNA Class I (Figure 4A-C – Deactivated, p-values<0.01). HOXA11-AS, associated with the cell cycle through E2F1[61], has been seen to promote gastric cancer proliferation and invasion (with EZH2), and can act as a 'molecular sponge' for EZH2 by absorbing (via direct interaction) of miR-1297[62]. SOCS2-AS1 is another lncRNA in this class that has been identified as an AR-regulated transcript[63] and further supports our hypothesis of AR-regulated lncRNAs within deactivated NEtD Class I. NKX2-1-AS1 exemplifies the activated NEtD Class II (Figure 4A-C – Activated, p-value<0.05) and has been previously seen to characterize lung cancer subtypes AD and squamous[64]. CDKN2B-AS1 (alias ANRIL) and H19 are prime illustrations for persistently expressed NEtD Class III (Figure 4A-C – Persistent, p-values<0.05). Both of these lncRNAs have been identified across a number of cancer studies (H19[51], ANRIL[65,66]), however depending on the cancer type each have functioned as a tumour suppressor (i.e. ANRIL deactivating tumour suppressors CDKN2A/B in cis by 3 different epigenetic mechanisms[67-69]) and as an oncogene (i.e. H19 acts as a sponge for FOXM1 by absorbing miR-342-3p[70]). Two demonstrations for transiently expressed NEtD lncRNA Class IV are FENDRR and CASC15 (Figure 4A-C – Transient, p-values<0.01). These lncRNAs are well studied in cancer; FENDRR for its prognostic value

and its involvement in gastric cancer metastasis[71] and CASC15 for its regulation of SOX4 in RUNX1-rearranged leukemia[72] and it harbours a risk SNP for susceptibility of neuroblastoma[73]. CASC15 has also been identified as a mediator of neural growth and differentiation[74], which we believe could be occurring in our NEtD model based on the data presented here. Each of these lncRNAs are among the top candidates identified in this study and a focus of our future research and functionalization. Taken together, these NEtD lncRNAs (n=742) characterize the transdifferentiation that occurs post-castration and is associated with treatment-induced NEPC.

NEtD lncRNAs are enriched with distinct transcription factor binding motifs

LncRNAs are not translated and carry out their functions post-transcription in their secondary or tertiary RNA form. This is unlike protein-coding transcripts that function in their post-translational form. Thus, identifying sequence motifs within lncRNAs could identify interacting transcripts or proteins that provide clues to function. Enrichment of transcription factor (TF) binding sites (TFBS) was determined by calculating Z-scores for overrepresentation of motifs present in the NEtD lncRNA Classes (I – IV) against their genomic background (Supplementary tables 6-17 and methods – Genomatix overrepresented TFBS). We also integrated each of these class-specific enrichment results to identify unique TFBS for each NEtD Class (supplementary table 18-19).

In NEtD Class I we identified 33 significant and uniquely enriched TFBS (Supplementary 5 and 19). Interesting results included binding motifs for TP53, scratch family transcriptional repressor 2 (SCRT2), and POU Class III homeobox 3 (POU3F3) (z-scores=4.02, 4.27, and 2.41, respectively). TP53 often absent in NEPC, could be an activating TF for many of these deactivated lncRNAs, and suggests an apoptosis or cell cycle arrest role is present here. SCRT2 has been linked as a neural-specific Snail family transcriptional repressor and critical for neuronal differentiation[75]. Similar to REST, this TF is likely causing the downregulation of a subset of these lncRNAs. Lastly, POU3F3/BRN1 (a member of the POU family of TFs, as is BRN2) is involved in the development of the nervous system, expressed in small cell lung cancer (SCLC) cells (which has pathological overlaps to NEPC), and involved in proneural/neuroendocrine differentiation[76]. Considering this and the significant enrichment of these TFs, this suggests a role in proliferation and differentiation in NEtD Class I.

Performing TFBS enrichment analysis in NEtD Class II and III identified 12 and 15 significant and distinct TFBS motifs, respectively (Supplementary Table 7-8 and 20). Interestingly, both classes had significant enrichment for at least one ETS and HOX family member, suggesting overlapping functional roles for their respective lncRNAs. For Class II this included ELF5, SPIC, and HOXD1 (z-scores=2.17, 2.43, 2.22, respectively) and for Class III, PDEF (alias SPDEF) and HOX/PBX (z-scores=3.03 and 2.71, respectively). Members of the ETS family fused to TMPRSS2 is the most frequent genomic alteration in PCa, therefore the prevalence of their motifs in these classes is not surprising. While the ETS fusion transcript is relatively more specific to PCa versus NEPC, ETS TFs on their own are involved in a wide variety of functions, including cellular differentiation and angiogenesis. In fact, recently SPDEF was found to be down-regulated in metastatic NEPC due to DNA methylation[32] and was also significantly down-regulated in treated verses untreated high-risk PCa patients[77]. Conversely, the HOX family has never been linked to PCa nor NEPC for that matter, and so this result was unexpected. In neuroblastoma however, the HOX genes have been linked to differentiating cells[78] and specifically HOXD1 identified here (as well as HOXC6 and HOXD8) are associated with differentiation towards a neuronal phenotype[79].

Performing TFBS enrichment analysis in NEtD Class IV identified enrichment of 17 distinct TFBS motifs (Supplementary Table 9 and 20). Class IV transcripts are only expressed during treatment (castration) response. Interestingly, heat shock TFs HSF1 (z-score=2.06) and HSF2 (z-score=3.87) were within these

results. Heat shock proteins (HSP) are expressed at low levels under normal conditions, upregulated by cellular stress, and function as molecular chaperones to control client protein stability and function. Their candidacy as therapeutic targets have been well-studied in PCa[80] and AR^+ CRPC[81]. In breast cancer, HSF1 specifically induces a cancer stem cell phenotype *in vitro*[82]. In PCa, HSPs bind dihydrotestosterone to the AR and enhance AR-mediated transcription. One of the functions of lncRNAs is to facilitate this type of mechanism. For example, LINC00152/CYTOR (identified within this class), binds and recruits EZH2 to its target promoters p15 and p21 in gastric cancer[83], IL24 in lung cancer[84], and thereby causes repression of their expression. Considering the transient expression of the lncRNAs in this class, this data suggests a subset may be stress response mediators via HSPs. Lastly, forkhead box A1 (FOXA1) showed a significant enrichment (z-score=2.8) in this class. Recently, FOXA1 loss was identified as a driver of NEtD[23], which leads to AR reprogramming[85] and EMT through direct regulation of SLUG expression[86]. This suggests that some of the lncRNAs in this class could have a functional role in maintaining cellular identity when under the control of FOXA1.

With FOXA1 as one of the characterizing TFBS in Class IV, we sought to explore the persistently expressed transcripts (Class III) in conjunction with transiently expression (Class IV). We hypothesized that subsets of these lncRNAs have mechanistic involvement in the transdifferentiation process. To investigate this we repeated the TFBS enrichment analysis on Class III and IV together and identified 6 significantly enriched TFBS (Supplementary Table 10 and 20). Confirming our hypothesis was the presence of TWIST1 (z-score=4.01), an essential member of the EMT transcriptional reprogramming factors[87]. Interestingly, TWIST1 and AURKA have very recently been seen to form a feedback loop promoting metastasis, highly aggressive phenotypes in pancreatic carcinoma[88], and TWIST1 is a marker for EMT in neuroendocrine tumours[89,90]. Concerning PCa, it has been identified as AR-regulated (and repressed via NKX3-1), whereas in the absence of AR is up-regulated and present in metastatic disease[91].

We further investigated global functional characteristics across all NEtD lncRNAs. Specifically, we wanted to identify TFBS that were significantly enriched and common across all classes. Due to the high number of lncRNAs (n=2,147), we decided to perform this analysis at the TF family level, therefore for each class and the full lncRNA set we repeated the motif enrichment analysis and integrated all of their results (Supplementary Tables 12-17 and 19). We identified 62 significantly common TFBS (z-score=>2, Supplementary Table 20). Not surprising were families involving cell cycle regulation, cyclin B2/CCNB2 and the E2F family (z-scores=40.66 and 67.36, respectively). We also observed both the ETS (z-score=9.9) and REST (z-score=20.52) families of TFs, which reaffirmed our hypothesis that these lncRNAs are involved in tumour progression and neuronal pathways. Surprising was the presence of two PAX families, PAX5 (z-score=10.15) and PAX9 (z-score=18.18). The PAX family is known to regulate lineage specification and progenitor cell maintenance. In developmental biology, PAX5 is involved in B-cell differentiation and PAX9 in neural crest development. PAX5 has been observed as overexpressed and in other NETs[92,93], neuroblastoma[94], and shown to positively regulate c-Met transcription in SCLC[95]. In lung NETs, PAX6 is prognostic for aggressiveness[96]. Their role in NEPC is yet to be characterized, however evidence here supports their global involvement in NEtD and lncRNA function. Lastly, the selenocysteine tRNA activating factor (STAF, z-score=15.18) was very intriguing to us. A recent Nature study by Schreiber et al. suggested that treatment resistance in NEtD of PCa depends on a druggable lipid-peroxidase pathway that protects against ferroptosis (a non-apoptotic form of cell death)[33]. The increased lipid metabolism creates a dependency on GPX4, which prevents ferroptosis. GPX4 is a selenocysteine-containing enzyme and one of only 25 proteins with this rare amino acid in the entire human genome. The data suggest that some of these lncRNAs may be involved in the selenocysteine pathway via STAF and involved in selenoprotein biosynthesis of molecules (i.e. GPX4). Identifying and targeting these lncRNAs could be an avenue for upstream inhibition of GPX4 up-regulation and therefore allow cell death in these resistant cells to occur

naturally by ferroptosis. Comprehensive *in vitro* experimentation would need to be carried out to confirm this therapeutic avenue.

NEtD lncRNAs contain NEPC-related Transcription Factor Binding Sites

It is now well established that complex cellular reprogramming occurs during NEtD and master regulators such as REST[31], BRN2[22], SOX2[28], and SOX11[18] have been identified as key TFs involved in this process. Identification of well-known TFBS such as these would test our current hypotheses on the functional involvement of individual lncRNAs in NEtD pathogenesis (Supplementary Tables 20-23). TFBS identification was carried out using MatInspector[97-99] (see methods – Genomatix Matbase and MatInspector) on each of the NEtD Class for select TFs.

With the dominance of AR-regulated genes in AD, the lack of expression observed in Class I are likely caused by the absence of androgen (post-castration) and are therefore candidate AR-regulated lncRNAs. To test this, we searched for androgen and glucocorticoid response elements (ARE and GRE, respectively). The results showed that 107 lncRNAs contained ARE and/or GRE motifs, of which 16 contained only an ARE motif, 49 contained only a GRE motif, and 21 contained both ARE and GRE motifs (Supplementary Table 21). To further test and support our AR-regulated lncRNA hypothesis we explored all previously reported AR-regulated lncRNA. Currently, the following 17 lncRNAs have been identified with experimental evidence: PCGEM1[100], PlncRNA-1/CBR3-AS1[101], PCAT-18[102], PCAT29[103], SOCS2-AS1[63], RP1-45I4.2[104], SUZ12P1[104], SNHG5[104], LINC01138[104], SNHG1[104], KLKP1[104,105], LINC00969[104], LINC-PINT[104], TUG1[104], MIR17HG[104], POTEF-AS1[106], and CTBP1-AS1[107]). Of these, 4 (PCAT29, SUZ12P1, SNHG1, and CTBP1-AS1) were not within our pipelines lncRNA class annotation, and 8 of 13 (61%) were represented in NEtD Class I deactivated lncRNAs (PCGEM1, PlincRNA-1, PCAT-18, SOCS-AS1, KLKP1, LINC00969, LINC-PINT, and POTEF-AS1). Due to our integrative study design (Figure 3A-C), the remaining 5 did not move forward in the analysis. However, removing the integrative steps, down-regulation of these lncRNAs did occur in either our model or patient samples independently. Overall, of the 13 lncRNA annotated by our pipeline and reported as AR-regulated, all overlapped in this study.

Due to their activating pattern of expression, we hypothesized that a subset of lncRNAs in these classes are constituents of the neuronal phenotype present in NEPC. To test this hypothesis, we analyzed these lncRNAs for the presence/absence of select TFs known to induce NEtD; POU Class III homeobox 2 (POU3F2) – also known as BRN2 and RE1 silencing transcription factor (REST). Activation of BRN2 and deactivation of REST are involved in neuronal differentiation and regulation of neurogenesis, respectively. Again, using the MatInspector algorithm we identified 11, 22, and 21 lncRNAs in Class II or III with TFBS for BRN2, REST, or both, respectively (Supplementary Table 22). Taken together, this evidence supports involvement for a subset of these lncRNAs to neuronal function/pathways in NEtD.

To further support the hypothesis of mechanistic involvement for the NEtD process in Classes III and IV, we expected TFBS related to plasticity and stemness to be present. Therefore, we used MatInspector to identify binding motifs for members of the following well-studied cellular differentiation TF families: HOX[108], SOX, STAT3[109], and 'STEM' (STEM members are defined by Matbase and include POU5F1/OCT4, SALL4B, SOX2, NANOG, and TCF7L1). We observed 42, 49, 30, and 33 lncRNAs with TFBS for HOX, SOX, STAT3, and STEM genes, respectively (Supplementary Table 23). In fact, some lncRNA had TFBS within more than one of these TFs (Figure 5A). Previous studies have linked 6/7 of these (highlighted in Figure 5A) to various components of EMT and/or cellular plasticity. FENDRR (antisense lncRNA to FOXF1) regulates gastric cancer metastasis via fibronectin1[71]. FOXD2-AS1 regulates EMT and Notch signaling to promote colorectal cancer[110]. H19 has been identified as a mediator of breast cancer plasticity during EMT and its reverse process mesenchymal-to-epithelial transition (MET)[111], as well as having a role in stemness

in prostate cells[112]. LINC00152 is involved in EMT (combined with cell migration and invasion) in gastric cancer[113]. LINC00478 (alias MONC) interferes with hematopoietic lineage decisions and enhances proliferation of immature progenitor cells in acute megakaryoblastic leukemia[114]. Lastly, again in gastric cancer, lncRNA SNHG6 has been seen to promote cell proliferation and EMT[115]. Based on this data, Class III and IV lncRNAs could have a role in developing a cellular 'plastic' state during NEtD.

To test the involvement of known NEPC-involved TFs in all NEtD lncRNA, we searched for BRN2, ARE/GRE, REST, SOX11, SOX2, NMYC, ETVI, ETS, and NKX3 motifs (Supplementary Table 24). Since each of the classes had different sizes this would influence the distribution and presence/absence of these motifs, so we extracted the top 25 lncRNA within each class (n=100 NEtD lncRNA), ranked by their magnitude of fold change. Observing the distribution of these TF separated by NEtD class revealed an interesting pattern (Figure 5B). TFs SOX2, SOX11, and REST had a relatively more balanced distribution across each class compared to NKX3, ETSF, ETVI, and NMYC, which showed a preference to binding persistent and transiently expressed lncRNA. Interestingly, over 50% of ARE/GRE motifs were present in transiently expressed lncRNA versus relatively few in Class I deactivated and Class II activated. Conversely, BRN2 motifs were relatively more present in Class I and II. These patterns suggest a time-dependent or cellular phase-dependent usage of TFs post-castration and during the NEtD process.

NEPC and NEtD lncRNAs identify putative NEPC subtypes

To corroborate the lncRNA expression in an external NEPC (extNEPC) cohort[32], we visualized NEtD lncRNA Classes II-IV and the up-regulated NEPC lncRNA expression including genomic profiles (copy number and mutation) through an OncoPrint schematic. The cohort consisted of 44 NEPC specimens (largest published to date) from 30 patients that were classified based on their histomorphology[6]. Due to the exome-sequencing performed on this cohort, not all lncRNAs were represented/detectable in this sequencing profiling. We also plotted previously reported NEPC predisposing genes, oncogenes, drivers, and the TFs we identify above to provide "transcriptome context" for the altered lncRNAs (Supplementary Figure 6-7). From the 58 NEPC and 243 NEtD lncRNAs represented in the extNEPC exome sequencing profiling, 43% (25/58) and 27% (66/243) showed altered expression in 2-34% of NEPC patients, respectively (Supplementary Figure 6, 8-11).

Surprisingly, these testable lncRNAs (58 from the NEPC lncRNAs and 66 from the NEtD lncRNA) in combination with known oncogenes/tumour suppressors/transcription factors (supplementary figure 7) resulted in identifying three distinct subsets of NEPC patients within the extNEPC cohort. Group 1 had relatively higher mutation frequencies, higher ploidy, mixed tumour sites, and mixed pathological classifications. Group 2 had a relatively low mutation frequency, low ploidy, derived mostly from pelvic masses, and with pathological classification D (large-cell neuroendocrine carcinoma). Whereas, Group 3 tumours were mostly derived from the prostate with a pathological classification B, and likely primary (*de novo*) NEPC samples where NEtD has not occurred. Of note, copy number loss or mutations in TP53 and RB1 were present in 60% of patients (26/44), spread across the cohort, and did not appear to be associated with a particular group (Supplementary Figure 7). The three groups could be revealing an lncRNA expression signature that is specific for tumour site, degree of genomic mutations (SNPs or CNVs), and pathological classification. However, it is important to note this is an observational result requiring statistical validation in a larger cohort. The specificity of these genomic and lncRNA transcriptome profiles would need to be explored across a variety of metastatic sites and NEPC pathologies to validate these three novel NEPC molecular subtypes.

NEPC and NEtD lncRNAs are associated with treatment-related metastasis

Prognostic and predictive biomarkers for NEtD and NEPC are in dire need since ADT is not effective for a cancer that has undergone NEtD and thus circumventing the AR signalling axis. We examined if the NEtD (n=742) and NEPC (n=122) lncRNAs are associated with NEPC related clinical outcomes in patients with primary prostatic adenocarcinoma. To accomplish this, we explored the candidates in two cohorts from the Mayo Clinic (MCI[116] and MCII[117]) from the Decipher GRID database (GRID) (n=777, Table 2). We could not perform this analysis within VPC/WCM cohorts due to their small sample sizes and short-term clinical follow up. The GRID cohorts represent tumours primarily with adverse pathology (i.e., high grade/stage) and long-term follow up for treatment and outcomes (median 18 years). From these cohorts, a subset (n=211) received adjuvant ADT post-radical prostatectomy (RP). To determine the most clinically relevant lncRNA transcripts, we first ranked the NEtD/NEPC lncRNAs within their respective classes and selected the top deregulated from each. The ranking was performed based on fold changes observed within the clinical groups (see Methods). This produced 100 top ranking NEtD/NEPC lncRNAs that we investigated within the GRID cohorts (Figure 1C and Supplementary Table 26). We validated 11 of these (2 from each NEtD Class and 3 from the NEPC lncRNA signature) by quantitative real-time PCR to confirm expression changes identified in the model and clinical samples (Supplementary Figure 12). Due to the difference in profiling platforms between GRID (Affymetrix microarray) and VPC/WCM cohorts (Illumina Sequencing), it was necessary to remap the GRID microarray probes (see Methods) that aligned within NEtD/NEPC lncRNA sequenced regions. This resulted in 81/100 being present and quantifiable on the microarray platform.

A characteristic of NEPC patients in the clinic is the occurrence of rapid metastasis following treatment[118], and so we first tested the lncRNAs ability to predict rapid metastasis post-ADT. We performed receiver-operating characteristic (ROC) analysis to compare the sensitivity and specificity of predicting rapid metastasis (within 36 months) for each lncRNA. We then calculated the area under the curve (AUC) for each lncRNA ROC, in both cohorts using probe set region expression summarized across the full lncRNA transcript (Supplementary Table 26). This identified eight lncRNAs: NR2F1-AS1, LINC00654, FENDRR, PCAT2, and NKX2-1-AS1 in MCI (AUC>0.70) and LINC00478, LINC00173, and LINC00514 in MCII (AUC>0.70) with the highest scores. These lncRNAs serve as candidates for predicting rapid metastasis in patients receiving ADT. Selecting all NEtD/NEPC lncRNAs with AUC>0.65 (n=25), we performed survival analysis to ascertain their ability to separate patients for metastasis as an outcome and end-point. Specifically, we calculated Kaplan-Meier estimates for metastatic disease progression stratified by median expression in ADT-treated samples of the MCII cohort. The expression of two NEtD/NEPC lncRNA transcripts (SSTR5-AS1 and LINC00514) was able to separate patients more likely to develop metastatic disease from those that did not (p-value=0.005 and p-value=0.010, respectively – Figure 6A). To increase our confidence that the results are associated with treatment status, we generated Kaplan-Meier estimates for these transcripts in untreated patients from the same cohort, and neither showed significant separation in their performance (p-value=0.905 and p-value=0.832, respectively – Figure 6B). Expression for SSTR5-AS1 and LINC00514 in the VPC and WCM cohorts illustrates their distinct and elevated expression in NEPC vs. AD patient samples (Figure 6C). These results suggest a strong association between treatment status and increased probability of metastatic disease in patients with differential expression of these lncRNAs. This, together with results from the NEtD model and NEPC clinical samples, implicates SSTR5-AS1 and LINC00514 in NEPC and serve as strong candidates as predictive biomarkers for metastatic disease post-RP following ADT.

One of the mechanisms observed with lncRNAs is direct RNA-RNA interaction with mRNA, resulting in regulation of their expression (activation or repression). This type of investigation is computationally intensive, and there are limited algorithms available to identify putative mRNA targets genome-wide.

However, a method was recently published to predict lncRNA-mRNA interactions genome-wide[119], and so we sought to identify candidate mRNA transcripts interacting with SSTR5-AS1 and LINC00514. The pipeline's three core algorithms include Raccess[120] for the identification of accessible regions within the lncRNA, IntaRNA[121] to calculate nucleotide interaction energies, and RactIP[122] to predict joint secondary structures. Applying this methodology to SSTR5-AS1 and LINC00514 produced a list of predicted interacting partners for these lncRNAs (Supplementary Table 27-28). The top-ranked mRNAs were KDM4B and TADA3 that are predicted to hybridize independently and form joint structures with SSTR5-AS1 and LINC00514, respectively (Supplementary Figure 12-13). In the clinical cohorts, TADA3 is down-regulated in NEPC versus AD (>2 fold), while KDM4B is up-regulated (>5 fold), however only the deregulation of TADA3 is statistically significant (VPC p-value=0.003 and WCM p-value=0.017). Both genes have NEPC associations (see Discussion) and our data suggest they are being regulated by these lncNRAs.

DISCUSSION

Primary NEPC arises *de novo* in 0.5% to 2% of all prostate cancer patients[123]. However, treatment-induced NEPC (tNEPC) can develop in 20-30% of mCRPC tumours[124] and increases with disease progression[125]. The real incidence of tNEPC may be higher because of under-recognition due to tumour heterogeneity, the limited number of metastatic tumour biopsies performed, lack of uniform consensus definition based on histology or biomarker expression, and frequent misclassification as high-grade PCa (most notable in tumours with mixed histologies)[126]. NEPC can be induced *in vitro* in AR^+ LNCaP cells in androgen-depleted culture conditions[127,128], similarly *in vivo*[7,129], and in patient tumours long-term ADT has increased neuroendocrine differentiation[118,124,130]. It is now common to observe treatment-resistant tumours with neuroendocrine features upon metastatic biopsy, and the prevailing consensus is that epithelial plasticity enables tumour adaptation in response to AR-targeted therapies[7,9,118,126,131-134]. This evidence supports the notion that tNEPC incidence through NEtD will increase as new powerful ADTs enter the clinic. There is an urgency for therapeutic strategies and clinical biomarkers defining NEtD/NEPC. Currently, the only option for patients is the short-lived effects of platinum-based chemotherapy. Optimism is on the rise as there is an AURKA inhibitor (MLN8237) in a Phase 2 clinical trial (NCT01799278), combinational approaches using AURKA with PARP inhibitors under investigation[135], indirect methods that resensitize the tumour to Enzalutamide[136] or platinum-based chemotherapy[137] (Phase 2 clinical trial NCT02489903 with a Phase 3 clinical trial being planned), a SSTR4/5 analogue (Pasireotide/SOM230) in four independent clinical trials at various Phases (NCT01646684, NCT01313559, NCT01468532 and NCT01794793) with one already reporting promising clinical efficacy[138], and increased study of NEPC/NEtD in general[134,139,140].

In this study, we characterized the unexplored global lncRNA landscape during NEtD to provide insights into the NEPC non-coding milieu of this lethal and treatment-induced process. This required the implementation of a sequence analysis pipeline with increased sensitivity towards lower expressed transcripts, characteristic of lncRNAs. The pipeline was able to detect 37,749 lncRNA transcripts (subclassified as either lincRNA, antisense or pseudogene) and quantify them in the two clinical cohorts (VPC and WCM). The novelty of this study lies in the use of patient samples integrated with the NEtD PDX model to detect clinically relevant lncRNAs involved in the NEtD/phase transition process. In this study, we identified 742 lncRNAs associated with NEtD and identified a robust 122 NEPC lncRNA patient signature capable of classifying NEPC from AD patient samples. The motif analysis identified significantly enriched TFBS that were unique to NEtD Class I (TP53 and BRN1), II (ELF5, SPIC, and HOXD1), III (SPDEF and HOX), IV (TP53, HSF1, HSF2, and FOXA1), and III combined with IV (TWIST1). Through similar analysis, we also identified common TFBS (CCNB2, E2F, ETS, REST, PAX5, PAX9, STAF) enriched across all of the NEtD lncRNAs. From among the 100 top ranking lncRNA, we observe that a subset have strong clinical

associations with metastatic PCa patients after receiving ADT. In previous lncRNA studies in cancer, several have been linked to malignant transformation with key roles affecting various aspects of cellular homeostasis including proliferation, survival, migration, and genomic instability[141]. Similarly, lncRNAs identified in this study including SSTR5-AS1 and LINC00514 with their association with poor outcome, FENDRR for its association with rapid metastasis, and H19 and LINC00617 for their concordantly high expression across both of the discovery cohorts, could be the missing links in the mechanisms causing NEtD. These five represent the top candidates discovered in this study due to this evidence but also for their characterization in other cancer types.

FENDRR is a top deregulated lncRNA in NEtD Class IV Transient and may have a role in the NEtD process. It is implicated in a lethal lung development disorder[142], lung cancer[143], within a mutational hotspot and a copy number lost region of the PCa genome[144], and can bind to PRC2[145,146]. PRC2 plays a significant role in tumour progression through binding of HOTAIR (a very well-studied lncRNA). Together, HOTAIR and PRC2 are involved in the control of chromatin structure and associated gene activity[147]. FENDRR may be involved in tumorigenesis like HOTAIR due to its known interaction with PRC2. A recent study showed down-regulation of FENDRR is associated with poor prognosis in gastric cancer and regulates cancer cell metastasis through fibronectin[71]. Functionally, this could be occurring in NEPC as well due to FENDRRs transient expression in the NEtD model and its association to rapid metastasis in ADT-treated PCa patients from the GRID (MCI) cohort. Another putative function of this transcript is through upregulating FOXF1, which is a protein-coding gene and the sense form for the antisense transcript FENDRR. Antisense transcripts are known to regulate their sense forms (positively or negatively). Using TANRIC, an interactive resource for the exploration of lncRNAs in large patient cohorts within 20 TCGA cancer types[148], we see that FENDRR expression is positively correlated to FOXF1 in 16 of 20 cancer types (p-value $< 3.71 \times 10^{-9}$, Supplementary Table 29). In fact, FOXF1 deletion has been seen to reduce FENDRR in endothelial cells significantly[149]. FOXF1 is also a target gene of p53 and seen to regulate cancer cell migration and invasiveness[150]. Together these transcripts may play a transient coordinated role in NEtD through PRC2 or fibronectin.

LINC00514 is amongst the highest expressed lncRNAs in NEtD Class III. It has not been characterized. It is predicted to bind to TADA3 (Supplementary Figure 14), potentially causing a reduction of its activity. This is intriguing because TADA3 is involved in the stabilization and activation of p53[151,152] and this putative interaction (LINC00514:TADA3) could be an alternative mechanism for loss of p53 activity, already known to be frequently lost in NEPC[15]. H19 and LINC00617 were two of the four highest (>10 fold) NEPC expressed lncRNAs in this study and fortunately (unlike most lncRNAs) have both been thoroughly characterized functionally. LINC00617 is highly conserved across vertebrate genomes, required for maintenance of pluripotency, and neural differentiation in embryonic stem cells[153]. It controls this lineage commitment through RNA-binding proteins (RBPs) PTBP1, hnRNP-K, and Nucleolin where these RBP complexes have been detected at promoters of NANOG, SOX2 (promoter of lineage plasticity in NEPC[28]), and FGF4[153]. H19 has also been identified in neural differentiation of pluripotent stem cells[154] but with unknown mechanisms. With such an elevated level of expression in the clinical cohorts (~30-40 and ~20-30 fold in VPC/WCM for LINC00617 and H19, respectively) these lncRNA could be responsible for maintaining the neuronal component of NEPC through epigenetic regulation.

SSTR5-AS1 is the highest expressed lncRNA in the NEPC clinical samples when requiring expression concordance in VPC and WCM cohorts. It is an antisense transcript of SSTR5, which is a member of the superfamily of somatostatin receptors. Somatostatins are peptide hormones that regulate diverse cellular functions such as neurotransmission, cell proliferation, and endocrine signalling, as well as inhibiting the release of many hormones and other secretory proteins. The SSTR family (1 through 5) are markers for

neuroendocrine tumours of the lung (NELC)[155] with SSTR1 and SSTR5 the most dominant forms of SSTR in neuroendocrine tumours in general[54]. Interestingly, exploration within TANRIC showed a strong positive correlation in expression with SSTR5 to SSTR5-AS1 in 14 of 20 cancer types (p-value < 2.18×10^{-15}, Supplementary Table 29). Furthermore, SSTR5 mRNA is detectable in the blood of NELC[156] and could be a valuable non-invasive diagnostic marker for NEPC. In fact, clinicians utilize this biological feature in other neuroendocrine tumours (NETs) using Octreoscans to determine tumour stage and/or identification of sites of metastasis. Octreoscans, when compared to positron emission tomography (PET) scans (a commonly used approach for this), appears more sensitive in the detection of well-differentiated NETs[157]. In addition to this, therapeutically, somatostatin analogues are emerging as a promising treatment option for inoperable or metastatic NETs[158]. However, specifically in NEPC, targeting SSTR5 and/or SSTR5-AS1 for diagnostic or therapeutic purposes is in its infancy. Interestingly, SSTR5 (C-terminal) is required for Rb induction and G1 cell cycle arrest[159], resulting in anti-proliferative effects. However, without Rb (known to be lost in NEPC), this function would be negated. Alternatively, the interaction evidence for SSTR5-AS1 and KDM4B (Supplementary Figure 13) provides another strong connection to NEPC biology. KDM4B is a histone demethylase and a key molecule in AR signaling and turnover[160]. In NEPC with the absence of the AR, KDM4B could interact with N-Myc instead, where it has been shown to regulate and epigenetically activate this oncogene in neuroblastoma[161]. N-Myc has been seen to drive the progression of NEPC[5,26,27] and recently through EZH2 mediated transcription[27]. Another mechanism of activation could be facilitated through SSTR5-AS1 regulation. However, both of these putative functions (SSTR5-AS1:SSTR5 or SSTR5-AS1:KDM4B:N-Myc) require thorough in *vitro* and in *vivo* exploration to ascertain their validity.

Although multiple layers of genetic and epigenetic deregulation likely cooperate to facilitate NEtD, understanding the non-coding contribution to this multifarious process is necessary to design effective novel therapeutics. Using the five independent patient cohorts and our proven NEtD PDX LTL331 model, lncRNAs such as FENDRR, LINC00514, LINC00617, H19, SSTR5-AS1 and others identified in this study may provide more in-depth insights to NEtD and NEPC. Research identifying the relationship of these lncRNAs to other known drivers, oncogenes, and activated pathways in NEtD is now required. This study is the first to report the lncRNA landscape of NEtD, a robust NEPC lncRNA expression clinical classifier, and provides numerous candidates for evaluation of biomarkers and therapeutic targets.

METHODS

Patient-derived xenografts

Animal ethics, care, experiments, xenograft generation, and all protocols were carried out in accordance with the guidelines of the Canadian Council of Animal care as previously described[7]. Specific xenograft models used in this study have been previously published (protein-coding transcriptomes) by Akamatsu et al.[24] and Mo et al.[162]. In brief, six LTL331, two LTL313, and two LTL418 patient-derived xenografts were raised in NOD-SCID mice (NOD.CB17-Prkdcscid/J) at the Living Tumor Laboratory (www.livingtumorlab.ca). Xenograft tissue was harvested after fixed lengths of time post host castration, tissue was measured, fixed for histopathological analysis, and processed for RNA analysis.

Clinical datasets

We used five clinical cohorts from 1) Weill Cornell Medicine (WCM)[5]; 2) GenomeDx Biosciences (GX) Inc. (MCI and MCII); 3) Johns Hopkins School of Medicine (JHSM); and 4) Vancouver Prostate Centre (VPC), cumulatively totalling 927 samples. For the VPC, 80 specimens were obtained from patients undergoing radical prostatectomy (RP) and snap frozen following a protocol approved by the Clinical Research Ethics Board of the University of British Columbia (UBC), the BC Cancer Agency, and Vancouver General Hospital

pathology (depending on the sample source). All patients signed a formal consent form approved by the ethics board. A subset of the GX Decipher GRID™ database of clinical specimens was selected, totalling 777 patient PCa expression profiles (all from FFPE tissue) and were obtained from two RP Mayo Clinic (MC) cohorts that have been previously described (MCI[116] and MCII[117]). JHSM samples, totalling 33 samples were retrieved from surgical pathology and consultation files of Johns Hopkins Hospital (John Hopkins Registry) from 1999-2013, as previously described[163]. The 33 were annotated as 6 morphologically-diagnosed pure prostate small cell carcinoma samples (SCPC), 12 high risk (Gleason 9-10) Adenocarcinoma (AD), 10 SCPC (SC-mixed) and 5 AD (AD-mixed) from mixed histology tumours containing separate adenocarcinoma and small cell components. For this cohort, samples were dicotimized into either AD (AD and AD-mixed samples) or NEPC (SCPC and SCPC-mixed sampels) for the purposes of validating the 122 NEPC lncRNA patient signature. We also explored an externally processed cohort comprising 114 metastatic CRPC specimens, of which 44 were NEPC[32] and used in this study. Referred in text as the extNEPC cohort, we accessed and visualized this data through cBioPortal[164,165] Version 1.9.0 (www.cbioportal.org). OncoPrint schematics were generated for displaying multiple genomic alterations by heatmap for the lncRNAs. The extNEPC study samples were classified using a pathologic classification system[6] that included five catagories: 'A', usual prostate adenocarcinoma without neuroendocrine differentiation; 'B', usual prostate adenocarcinoma with neuroendocrine differentiation > 20%; 'C', small-cell carcinoma; 'D', large-cell neuroendocrine carcinoma; and 'E', mixed small-cell carcinoma–adenocarcinoma.

Material collection and processing (VPC Cohort)

Hematoxylin and eosin (H&E) stained, formalin-fixed paraffin-embedded (FFPE), and fresh frozen sections were reviewed by a pathologist to identify blocks with highest tumour content. For each frozen block used, a 5µm slide was first taken for H&E staining; then 4x100µm sections were taken for DNA and RNA isolation before a 2nd 5µm slide was taken for H&E staining. Each H&E slide was required to have tumour content >50% for a tumour to proceed for sequencing. RNA from 100µm sections of snap frozen tissue were isolated using the mirVana Isolation Kit from Ambion (AM 1560). RNA sequencing was performed on Illumina HiSeq 2000 at BC Cancer Agency Michael Smith Genome Sciences Centre according to standard protocols.

Material collection and processing (GRID and JHSM)

For GRID (MCI and MCII) and JHSM cohorts, specimen selection, RNA extraction, and microarray hybridization was performed in a Clinical Laboratory Improvement Amendments (CLIA)-certified laboratory facility (GenomeDx Biosciences, San Diego, CA, USA) as described previously[116,117]. Total RNA extraction, purification, RNA amplification, and labelling were done using the Ovation WTA FFPE system (NuGen, San Carlos, CA). RNA was hybridized to Human Exon 1.0 ST GeneChips (Affymetrix, Santa Clara, CA). After microarray profiling, quality control was preformed using the Affymetrix Power Tools package, and probe set normalization was performed using the Single Channel Array Normalization (SCAN) algorithm[166].

Quantification Real Time Polymerase Chain Reaction (qRT-PCR)

Primers were designed using Primer3 and checked with in silico PCR in UCSC Genome Browser (See supplementary Table 26 for forward and reverse primer sequences). Housekeeping genes PSMB4, REEP5, and SNRPD3 were selected on the basis of high, consistent expression levels across many cell and tissue types, and used in the MiTranscriptome lncRNA study[167,168]. Two lncRNAs from each NEtD class and three NEPC lncRNAs from among the top candidates (Supplementary Figure 12 and Supplementary Table 26) were selected (n=11) for quantitative real time polymerase chain reaction (qRT-PCR) validation. The cDNA from the PDX LTL331 models three time points (AD, postTX, and NEPC) were used to validate the NEtD

lncRNAs and a subset of the VPC clinical samples for the NEPC lncRNAs. With the rarity of clinical NEPC samples, tumour tissue and subsequent RNA was extremely limited. Due to this, only three NEPC (V73, V90, and V91) and one AD (V60) clinical sample were included in this validation. For each lncRNA and sample tested, the following experimental protocol was carried out: one microgram of total RNA for each sample was diluted to 18ul with water and 1ul of random hexamers (50uM; Thermo Fisher). The mixture was heated to 65C for five minutes and chilled. Afterwards, 5ul of 5X reverse transcriptase buffer, 1ul of 10mM dNTP's, and 1ul of Superscript II reverse transcriptase (Thermo Fisher) were added. Each sample was then incubated at 42C for 1 hour and then at 70C for 15 minutes. Prior to use in qRT-PCR products were diluted 10-fold with water. FastStart Essential Green Master kit from Roche (Catalogue #06 402 712 001) was used as described from their protocol for qRT-PCR reactions. In brief, 2ul of water, 3ul of a mixture of forward and reverse primers (each at a concentration of 10uM) and 10ul of the Roche Master Mix was aliquoted into each well of a 96-well plate. A mixture of 4ul of water plus 1ul of the diluted cDNA was then added to the appropriate wells. Expression was then quantified (as measured by Ct) through the Roche LightCycler 96 machine. Each lncRNA/sample pair was quantified with technical replicates in triplicate. Average and standard deviation of Ct were calculated across these triplicates, and delta Ct calculated relative to house keeper gene PSMB4 (most consistent and highly expressed gene vs REEP5 and SNRPD3). Delta delta Ct's were calculated relative to control samples and fold changes were plotted using Prisms GraphPad software (Supplemental Table 12).

RNA sequence analysis pipeline

We implemented a lncRNA sequence analysis pipeline that includes algorithms catered to the detection of known and novel transcripts (Supplementary Figure 1-2). Developed in-house, this pipeline is modified and extended from the tuxedo suite of sequence analysis algorithms[45]. Once received from the sequencing centre in bam format, all sequenced model systems and patient samples were de-aligned into raw fastq format (including flagged reads) using bam2fastq and put through the following pipeline. To ensure high-quality sequence reads, libraries were trimmed using a windowed-adaptive approach (Sickle – https://github.com/ucdavis-bioinformatics/sickle). For each read pair processed together, the algorithm determines the most optimal inner read sequence by trimming both 3' and 5' prime ends based on quality and length thresholds (for full description see – http://bioinformatics.ucdavis.edu/software/). Bases with a quality score of less than 99.0% base call accuracy (corresponding to a Phred quality score of 20) were removed. Reads less than ~2/3 read length (30nt in WCM and 60nt in VPC) post-trimming were discarded. Highly repetitive sequences (>2% of library) were also discarded post-trimming using the cutadapt tool. All quality control metrics were generated and quanitified (pre- and post-trimming) using the FASTX-Toolkit and the FastQC Windows software. Reads were aligned to the Hg19 human genome build using an unspliced aligner for handling exonic reads (Bowtie - v2.2.3), in conjunction with a spliced aligner to handle reads spanning exon-exon junctions (Tophat – 2.0.12). Transcriptome reconstruction using Ensembl GRCh37.75 gene tracks for each library was performed using a quasi *de novo* (genome-guided) approach (Cufflinks – v2.2.1), where reads were assembled and abundances estimated using an overlap graph producing a minimal spanning network of transcripts. This version of Ensembl contained 38 transcript classes, grouped by 4 core biotypes. At this stage, transcripts were also multi-read and fragment bias corrected. Transcripts with highly abundant expression were masked (e.g. rRNAs) from downstream steps to increase transcript quantification accuracy. Sample transcriptomes, the reference genome, and the transcript annotation were then meta-assembled (Cuffmerge) to produce a single annotation transcriptome model. Based on this model, transcript quantification (Cuffquant) and normalization (Cuffnorm) for library depth and transcript length were performed. Transcript expression displaying computational artifacts (expression values < 0.1 known to occur with Cufflinks) were converted to zero values. This generated transcript expression where only lncRNAs (Ensembl and ENCODE-based) were extracted and used for all downstream analysis. All algorithms denoted in brackets are referenced and

described in Trapnell et al. Nature protocol[45]. Each cohort (VPC and WCM) was processed independently by this pipeline, and then transcriptome annotations were merged. This was accomplished using Ensembl transcript IDs combined with transcript lengths to produce unique transcript identifiers for each lncRNA across cohorts.

RNA-RNA Interaction Analysis

A genome-wide search for SSTR5-AS1 and LINC00514 lncRNA interactions was performed using a multistep systemic approach[119]. This tool is available publicly within an online database (http://rtools.cbrc.jp/cgi-bin/RNARNA/index.pl) hosted by the Computational Biological Research Center (CBRC) at the National Institute of Advanced Industrial Science and Technology in Japan. The interaction search space included all hg19 annotated lncRNA and mRNA transcripts. This generated top-ranking interaction partners (n=100), based on local interaction minimum free energy (Supplementary Table 27-28). R-chie[169] (http://www.e-rna.org/r-chie/) was used to visualize the top-ranking predictions KDM4B and TADA3 for SSTR5-AS1 and LINC00514, respectively using the double structure feature (Supplementary Figure 13-14). All bases that were not within the interaction site were predicted to form RNA secondary structure by RNAfold[170,171] (http://rna.tbi.univie.ac.at/cgi-bin/RNAfold.cgi) selecting enforced constrained pairing pattern for the interacting bases. Minimum free energy structures were predicted by RNAfold on the 300nt sequences upstream and downstream of the interaction site.

Transcription factor binding site identification and enrichment analysis

All transcription factor (TF) binding site (TFBS) analysis was performed using Genomatix software, databases, and algorithms (www.genomatix.de). Three types of TF analysis were carried out in this study: (1) Single lncRNA motif characterization, (2) Multiple lncRNA analysis for select TFs and (3) Multiple lncRNA enrichment analysis. Prior to any of the above, lncRNA transcript(s) were submitted to the Gene2Promoter algorithm for retrieval of promoter sequences. Databases used with this algorithm included ElDorado 12-2013 and NCBI build 37 (for multiple lncRNA analysis where genomic background needed to match sequencing data) or the most recent databases ElDorado 12-2016 and GRCh38 (for single lncRNA analysis where genomic background was not relevant). Transcripts with alternative isoforms were required to have gold level (experimentally verified 5' complete transcript), silver level (transcript with 5' end confirmed by PromoterInspector prediction), or bronze level (annotated transcript, no confirmation for 5' completeness) quality for their alternative isoforms. (1) Single lncRNA motif characterization was performed using the MatInspector algorithm[97-99] with parameters ‘core similarity’ (degree of similarity for highest conserved bases of motif) set to 1 and ‘matrix similarity’ (degree of similarity between motif and query sequence) set to optimized as recommended by Genomatix and as described in MatInspector referenced papers above. MatInspector uses the best in field MatBase database for TFBS motif/matrix annotation, where Matrix Family Library Version 10.0 was used. (2) Multiple lncRNA analysis for select TFs was performed using MatInspector and select TF motifs (‘matrix’) applied accordingly. All matrix annotation, descriptions, and matrix family definitions are listed in Supplementary Table 25. Select TF matrices: BRN2, STAT3, NKX3, NMYC, SOX2, and SOX11 and select TF matrix families: GREF (includes the androgen receptor and the closely related glucocorticoid, mineralocorticoid and progesterone receptors), NRSF (REST), SOX, HOX, STEM, E2FF, ETSF, and ETVI1 motifs included in this study are described in Supplementary Table 25. Core and matrix similarities were again set to 1 and optimized, respectively. (3) Multiple lncRNA enrichment analysis was performed using the Overrepresented TFBS algorithm. Enrichment of matrix/matrix family was determined by Genomatix calculated Z-scores (greater than 2 or less than -2), which is based on the distance from the population mean (genome or promoter sequence background) in units of the population standard deviation for query sequence/promoter. Genomatix calculates Z-scores with a continuity correction using the formula $z = (x-E-0.5)/S$, where x is the number of found matches in the input data, E is the expected value and S is the standard deviation. This formula

is also described in the oPOSSUM algorithm[172]. A Z-score below -2 or above 2 can be considered statistically significant and corresponds to a p-value of approximately 0.05.

Microarray to sequencing platform lift over / mapping

Affymetrix Human Exon 1.0 ST GeneChip probes were mapped to Hg19 coordinates using SMALT v0.76 (http://www.sanger.ac.uk/resources/software/smalt/). Probe set genomic regions (PSRs) were redefined accordingly. Exons within each lncRNA from sequencing cohorts (VPC and WCM) were integrated with PSRs to build an overlap table to determine absence/presence of lncRNA transcripts on the affymetrix microarray. R function iRanges v2.9.18 was used with method findOverlap to build the described table above. Microarray PSRs were required to be entirely within sequenced exon genome regions, otherwise they were excluded. Applying this methodology, 106 of 122 NEPC lncRNAs (87%) and 81 of 100 NEtD lncRNAs (81%) mapped to microarray PSRs for clinicopathological analysis on GRID cohorts MCI and MCII.

Statistical analysis

For all cohorts, the programming language R v3.0 was used for statistical analysis. For VPC and WCM cohorts, unsupervised hierarchical clustering was performed with the h.clust package with Pearson correlation for distance and average linkage used. Only transcripts within the top 5th percentile based on their standard deviations were selected. The clustering and heatmaps generated were built using the heatmap.2 function. Similar clustering analysis was performed for GRID cohorts except with Euclidian distance, the ward method for linkage, and the use of the heatmap.3 function due to its advanced row/column labelling features. For all cohorts before clustering, normalized log2 expression values were standardized/scaled using a Z-score that ranged from -2 to 2. For principal component analysis, the R package prcomp was used to calculate variance among transcript and sample subsets for the calculation of transcript weights and principle components. The top 3 components were used for visual inspection. For all clinical group-wise comparisons, a standard student t-test was applied to identify significantly differentially expressed transcripts between groups/phenotypes. Significance thresholds were implemented by enforcing a strict p-value cut-off of <0.05. Multiple test correction was applied to p-values using the Bonferroni and Hochberg method to *mathematically* minimize false discovery rate (FDR) and with a cut-off of p-value < 0.05. See supplementary table 4 for these results. To *biologically* minimize FDR, *mathematical* FDR correction was removed and instead followed the filter-down workflows in Figure 3A-C and Figure 1C. Despite *mathematical* FDR being removed, statistical significance of p-value < 0.05 was still maintained during the filter-down approach using the described method in each step. See supplementary table 5 for these results. For receiver-operating characteristic (ROC) curves and area under the curve (AUC) calculations, the R package 'pROC' was used. Kaplan-Meier analysis was performed for determining survival outcome using the R package 'survfit' with transcripts displaying below background (<0.1) expression being removed from this analysis.

Transcript Ranking

NEtD lncRNA transcripts were ranked based on fold changes observed in the clinical group-wise comparisons. For NEtD Class I Deactivated, the three group-wise comparison fold changes were used (NEPC vs. AD, CRPC vs. AD, and NHT vs. AD), where the minimum fold change observed between the 3 comparisons was selected and then ranked in decreasing order. For Class II Activated and Class III Persistent transcripts, NEPC vs. AD fold changes were calculated and ranked in increasing order for both VPC and WCM cohorts, where the maximum fold change between VPC and WCM was selected. For Class IV Transient transcripts, absolute fold changes for AD vs. NHT and NHT vs. NEPC were calculated and ranked in increasing order with the maximum fold change from either group was selected. Similar ranking was performed for NEPC lncRNA transcripts and were ordered in increasing/decreasing order to

determine the highest/lowest-expressed transcripts in NEPC vs. AD. Concordantly expressed transcripts were required between VPC and WCM cohorts. The top 20 lncRNAs (based on fold changes from clinical samples defined above) were taken from each group. This produced 20 x 5 groups (n=100) isoforms representing 76 unique lncRNA transcripts. These represent the top NEtD/NEPC lncRNA candidates from this study (Supplementary Table 26). No pseudogenes were included in these rankings.

AVAILABILITY OF SUPPORTING DATA

A subset of the sequenced samples (n=70) used in this study were from previous studies with all raw sequencing data reanalyzed here using the described pipeline above. They have been previously submitted to the European Nucleotide Archive (ENA) or NCBIs Gene Expression Omnibus (GEO). This includes the 6 NEPC PDX model samples[24] (ENA accession number PRJEB9660 and GEO accession number GSE59986), 2 CRPC PDX model samples[162] (ENA accession number PRJEB19256), 4 NEPC (VPC) samples[31,56], 23 AD (VPC) samples[56] (ENA accession number PRJEB6530), 30 AD (WCM) samples[5] and 7 NEPC (WCM) samples[5]. The remaining unpublished sequenced samples (n=55) have been submitted to the ENA under accession number PRJEB21092. Please see Supplementary Table 1 for a summary of sequencing and clinical information on these 125 samples. All microarray samples from GX cohorts, including 545 AD (MCI[116]) samples and 232 AD (MCII[117]) samples, are accessible through GEO accession numbers GSE46691 and GSE62116, respectively. Additional supporting data and custom code from the sequencing pipeline described above is also available from the *GigaScience* GigaDB database[173].

FUNDING

This work was supported by the Mitacs Accelerate PhD Fellowship Program (IT04310 to VRR) in collaboration with GenomeDx Biosciences, Terry Fox Foundation (201012TFF to CC), and Prostate Cancer Canada Team Grant (T2013-01 to CC).

AKNOWLEDGEMENTS

We are grateful to the following GenomeDx bioinformaticians: Mandeep Takhar for her help with GRID statistical analysis/primer code, Hussam Al-Deen Ashab for his help with IA analysis (ultimately wasn't included in the paper) but effort and knowledge gained from the results were insightful for this work, Nicholas Erho for his effort in mapping our sequenced data to the GRID microarray, mentoring, and constant support, and Mohammed Alshalalfa for his guidance and supervision on all NEPC/NEtD lncRNA clinicopathological GenomeDx analysis. We would also like to deeply thank Daniel Lai and Alex Gawronski for their advice with RNA-RNA visualization and interaction analysis algorithms. We would like to thank Faraz Hach for his manuscript insights and advice. Lastly, we are extremely grateful to Stephanie Giles Ramnarine for her manuscript comments, advice, and support.

COMPETING INTERESTS

The authors declare that they have no competing interests.

FIGURE LEGENDS

Table 1: Model samples used in the study. AR^+ and AR^- CRPC xenograft model samples and their associated molecular characteristics. AD, Adenocarcinoma; AN, Grows in the absence of androgen; BI, Resistant to

Bicalutamide; CRPC, Castration resistant prostate cancer; EZ, Resistant to Enzalutamide; M, Mutation present; NEPC, Neuroendocrine prostate cancer; TE, Grows in the absence of supplemented testosterone; WT, Wild type.

Table 2: Clinical samples used in the study. Patient samples and their associated clinical variables including treatment status, Gleason grading, and clinical endpoints. AD, Adenocarcinoma; ADT, Androgen deprivation therapy; BCR, Biochemical recurrence; CRPC, Castration resistant prostate cancer; MET, Adenocarcinoma metastasis; NAÏVE, Adenocarcinoma naïvely treated; NEPC, Neuroendocrine prostate cancer; NHT, Adenocarcinoma with neoadjuvant treatment; PCSM, Prostate cancer specific mortality; +RMET+ADT, ADT treated rapid metastasis (<36 months) with at least 10 years of clinical follow-up; -RMET+ADT, ADT treated no metastasis with at least 10 years of clinical follow-up; [1]Patient overlap exists across different sites of metastasis; [2]Contains a subset of mixed histology tumours (see Methods for breakdown); Hashed cells are clinical features that are unknown for cohort.

Figure 1: Transcriptome composition and Study Design. (A) Proportions and totals of transcripts detected using our next-generation sequencing pipeline. Transcripts were separated into protein coding (mRNA) or non-coding RNA (ncRNA) and as defined by Ensembl's core biotypes as either mRNA, long ncRNA, short ncRNA, or pseudogene. Within long ncRNA, there exist seven classes, including processed transcripts, retained intron, lincRNA, antisense, sense intronic, sense overlapping, and 3 prime overlapping ncRNA (the last three labelled as 'other'). Transcript totals are denoted around each pie slice. (B) The three transcript classes used in this study due to their ability to separate AD and NEPC tumours, which collectively totalled 37,749 lncRNAs. *The pseudogene total was the combination of eight pseudogene subclasses and collectively referred to as pseudogene here. These subclasses include processed pseudogene, unprocessed pseudogene, transcribed unprocessed pseudogene, transcribed processed pseudogene, translated processed pseudogene, polymorphic pseudogene, unitary pseudogene, and pseudogene. These lncRNAs formed the basis for all down-stream analysis and (C) the studies project workflow and study design. AUC, Area under the curve; GRID, GenomeDx Decipher GRID database; JHSM, Johns Hopkins School of Medicine; NEPC, Neuroendocrine prostate cancer; NEtD, Neuroendocrine transdifferentiation; PDX, patient derived xenograft; ROC, Receiver-operating characteristic; VPC, Vancouver Prostate Centre cohort; WCM, Weill Cornell Medicine cohort. See Table 2 for cohort clinical features and compositions.

Figure 2: NEPC lncRNA expression signature and clinical classifier - Unsupervised hierarchical clustering of the 122 identified lncRNAs from (A) VPC and (B) WCM cohorts. Validation of this signature was shown in the (C) JHSM cohort. Samples (columns) are labelled as adenocarcinomas (blue) or neuroendocrine (yellow) tumours. See supplementary figure 3 for row/lncRNA labels for each plot.

Figure 3: Xenograft model of neuroendocrine transdifferentiation, phenotype-driven integration, and NEtD associated lncRNAs. (A) Schematic depicting the time points at which xenograft tumours were collected along the transdifferentiation of AD to NEPC (adapted from Akamatsu et al., 2015[24]). (B) Phenotypes that align to various time points from above xenograft model and group-wise comparisons analyzed for clinical samples. (C) Four isolated expression profiles (grey triangles) from select time points in A (light grey circles) with appropriate clinical group-wise comparisons overlaid and integrated. Unsupervised hierarchical clustering with NEtD lncRNAs (Class I – Deactivated – Black bars, Class II – Activated – Orange bars, and Class III – Persistent – Red bars) identified from integration outlined in (C). Distinct clusters of AD and NEPC clinical samples are observed in the (D) VPC and (E) WCM cohorts. Class IV – Transient lncRNAs were excluded from the clustering due to the lack of clinical samples that would represent this intermediate state.

Figure 4: Select NEtD lncRNAs that exemplify each expression pattern are shown from the (A) NEtD PDX LTL331 model, (B) WCM, and (C) VPC cohorts. The expression for NEtD lncRNAs within Class IV – Transient were only identified through the VPC cohort due to the presence of NHT samples, which were not present

within the WCM cohort. All boxplots showed significant separation (p-value < 0.05) between groups based on a standard student t-test with the exception of * lncRNAs.

Figure 5: Transcription Factor Binding Site (TFBS) Venn Diagram and Distribution plots. (A) Common and unique TFBS for HOX, SOX, STAT3, and STEM families of transcription factors within Class III and IV of the NEtD lncRNAs. (B) Distribution of TFBS for known NEPC-involved TFs within NEtD Class lncRNAs.

Figure 6: Kaplan-Meier estimates and expression for SSTR5-AS1 and LINC00514. Kaplan-Meier estimates for metastasis-free survival in the MCII cohort comparing low (blue lines) and high (yellow lines) expression (split by median) in (A) treated patients that received post-prostatectomy adjuvant ADT for SSTR5-AS1 (left) and LINC00514 (right) and (B) patients not receiving ADT treatment. (C) Box plot expression for the top two NEPC lncRNA candidates (SSTR5-AS1 and LINC00514) within the VPC and WCM cohorts.

SUPPLEMENTARY DATA LEGENDS

Table 1: Discovery cohorts clinical and sequencing information

Table 2: Differentially expressed AR- and AR+ CRPC lncRNA

Table 3: NEPC lncRNA expression signature labels and order for Figure 2

Table 4: Clinical cohort group-wise comparisons

Table 5: NEtD and NEPC lncRNA Annotation

Table 6: Genomatix – Enrichment by Matrix NEtD – Class I

Table 7: Genomatix – Enrichment by Matrix NEtD – Class II

Table 8: Genomatix – Enrichment by Matrix NEtD – Class III

Table 9: Genomatix – Enrichment by Matrix NEtD – Class IV

Table 10: Genomatix – Enrichment by Matrix NEtD – Class III-IV

Table 11: Genomatix – Enrichment by Matrix NEtD – All

Table 12: Genomatix – Enrichment by Matrix Family NEtD – Class I

Table 13: Genomatix – Enrichment by Matrix Family NEtD – Class II

Table 14: Genomatix – Enrichment by Matrix Family NEtD – Class III

Table 15: Genomatix – Enrichment by Matrix Family NEtD – Class IV

Table 16: Genomatix – Enrichment by Matrix Family NEtD – Class III-IV

Table 17: Genomatix – Enrichment by Matrix Family NEtD – All

Table 18: Genomatix – Overlap table by Matrix

Table 19: Genomatix – Overlap table by Matrix Family

Table 20: Uniquely and Commonly Enriched TFBS in NEtD lncRNA

Table 21: Genomatix – Select TF Identification – NEtD Class I

Table 22: Genomatix – Select TF Identification – NEtD Class II-III

Table 23: Genomatix – Select TF Identification – NEtD Class III-IV

Table 24: Genomatix – Select TF Identification – NEtD Full Set

Table 25: Genomatix Matrix Descriptions

Table 26: Top-ranking NEPC and NEtD lncRNAs

Table 27: SSTR5-AS1 predicted RNA (mRNA or lncRNA) interactions with associated binding energies, predicted transcript Ensembl ID, name, interaction position, and ranking.

Table 28: LINC00514 predicted RNA (mRNA or lncRNA) interactions with associated binding energies, predicted transcript Ensembl ID, name, interaction position, and ranking.

Table 29: TANRIC results for lncRNAs FENDRR and SSTR5-AS1. Spearman rank correlation for protein coding genes that are the sense forms to the above antisense transcripts. Numbers in brackets denote p-values. NSC, no significant correlation; NA, mRNA data was not available for this tumour type, therefor the analysis was not applicable.

Figure 1: The next-generation sequence analysis pipeline implemented for the detection and quantification of lncRNAs in this study. (A) The 9-step lncRNA next-generation sequencing analysis pipeline with core algorithms (Bowtie2, Tophat2, Cufflinks2, Cuffmerge, Cuffquant and Cuffnorm) implemented from the Tuxedo suite of analysis tools. Sequencing quality control metrics before and after trimming of data for sample V60 is outlined in B-F. This includes pre-trimming (A) phred quality scores and (B) percentage of each base type across read library at each base pair position. After quality control corrections are applied V60 read library has acceptable (D) Phred quality scores (~30 Phred Score) and (E) expected base type percentages (~25%) for T, C, A, and G. (F) All over-represented sequences that are >2% of library is removed from the V60 read library. See Methods for complete listing and version numbers for all algorithms and tools used in the sequence analysis pipeline.

Figure 2: Average Phred quality scores for all VPC and WCM samples, pre- and post-quality control correction.

Figure 3: Unsupervised hierarchal clustering (A-D) and principle component analysis (E-H) on the four major Ensembl transcript classes detected within the VPC cohort. Samples are labelled as adenocarcinomas (blue) and neuroendocrine tumours (gold)

Figure 4: Unsupervised hierarchal clustering (A-D) and principle component analysis (E-H) on the four major Ensembl transcript classes detected within the WCM cohort. Samples are labelled as adenocarcinomas (blue) and neuroendocrine tumours (gold)

Figure 5: Detected and differentially expressed lncRNAs among (A) $AR^{-/+}$ CRPC xenograft models (B) and matched clinical samples. X, xenograft; P, patient.

Figure 6: NEPC and NEtD lncRNA Oncoprint Plot in the extNEPC Cohort – LEGEND. Clinical, transcriptome, and genomic annotation for samples plotted in supplementary figures 7-11. All annotation was generated from cBioportal with the exception of the NEPC molecular subtype, which was assigned from this study. Transcripts denoted with 1 in superscript within supplementary figures 8-11 are lncRNAs that overlap a NEPC lncRNA with a NEtD lncRNA. For example, H19 appears in supplementary figure 8 (NEPC lncRNA) and supplementary 9 (NEtD lncRNA Class II).

Figure 7: NEPC and NEtD lncRNA Oncoprint Plot in the extNEPC Cohort – Known NEPC genes and TFs. Select NEPC oncogenes, tumour suppressor, and transcription factors that have been reported previously or within this study for transcriptomic/genomic context with supplementary figures 8-11. Please see supplementary figure 6 for figure legend.

Figure 8: NEPC and NEtD lncRNA Oncoprint Plot in the extNEPC Cohort – NEPC lncRNA. Transcripts from the NEPC lncRNA expression signature that are up-regulated (74 of 122), testable (58 of 74), and altered (25 of 58) in the extNEPC cohort. Please see supplementary figure 6 for figure legend.

Figure 9: NEPC and NEtD lncRNA Oncoprint Plot in the extNEPC Cohort – NEtD lncRNA Class II. Transcripts from NEtD lncRNA Class II (222), testable (128 of 222), and altered (26 of 128) in the extNEPC cohort. Please see supplementary figure 6 for figure legend.

Figure 10: NEPC and NEtD lncRNA Oncoprint Plot in the extNEPC Cohort – NEtD lncRNA Class III. Transcripts from NEtD lncRNA Class III (84), testable (79 of 84), and altered (29 of 84) in the extNEPC cohort. Please see supplementary figure 6 for figure legend.

Figure 11: NEPC and NEtD lncRNA Oncoprint Plot in the extNEPC Cohort – NEtD lncRNA Class IV. Transcripts from NEtD lncRNA Class IV (45), testable (36 of 45), and altered (11 of 31) in the extNEPC cohort. Please see supplementary figure 6 for figure legend.

Figure 12: Quantitative real-time polymerase chain reaction on select NEPC and NEtD lncRNAs

Figure 13: Hypothetical RNA-RNA folding structure for exon 4 of SSTR5-AS1 (top) and the 3'UTR of KDM4B (bottom). Predicted base pair binding (green arcs) along the sequence (black arrow) are displayed, included predicted interaction site (orange bars).

Figure 14: Hypothetical RNA-RNA folding structure for exon 4 of LINC00514 (top) and the 3'UTR of TADA3 (bottom). Predicted base pair binding (green arcs) along the sequence (black arrow) are displayed, included predicted interaction site (orange bars).

REFERENCES

1 Torre, L. A. *et al.* Global cancer statistics, 2012. *CA: a cancer journal for clinicians* **65**, 87-108, doi:10.3322/caac.21262 (2015).

2 Karantanos, T. *et al.* Understanding the mechanisms of androgen deprivation resistance in prostate cancer at the molecular level. *European urology* **67**, 470-479, doi:10.1016/j.eururo.2014.09.049 (2015).

3 Grasso, C. S. *et al.* The mutational landscape of lethal castration-resistant prostate cancer. *Nature* **487**, 239-243, doi:10.1038/nature11125 (2012).

4 Vlachostergios, P. J., Puca, L. & Beltran, H. Emerging Variants of Castration-Resistant Prostate Cancer. *Curr Oncol Rep* **19**, 32, doi:10.1007/s11912-017-0593-6 (2017).

5 Beltran, H. *et al.* Molecular characterization of neuroendocrine prostate cancer and identification of new drug targets. *Cancer discovery* **1**, 487-495, doi:10.1158/2159-8290.CD-11-0130 (2011).

6 Epstein, J. I. *et al.* Proposed morphologic classification of prostate cancer with neuroendocrine differentiation. *Am J Surg Pathol* **38**, 756-767, doi:10.1097/PAS.0000000000000208 (2014).

7 Lin, D. *et al.* High fidelity patient-derived xenografts for accelerating prostate cancer discovery and drug development. *Cancer research* **74**, 1272-1283, doi:10.1158/0008-5472.CAN-13-2921-T (2014).

8 Aparicio, A. M. *et al.* Platinum-based chemotherapy for variant castrate-resistant prostate cancer. *Clinical cancer research : an official journal of the American Association for Cancer Research* **19**, 3621-3630, doi:10.1158/1078-0432.CCR-12-3791 (2013).

9 Wang, H. T. *et al.* Neuroendocrine Prostate Cancer (NEPC) progressing from conventional prostatic adenocarcinoma: factors associated with time to development of NEPC and survival from NEPC diagnosis-a systematic review and pooled analysis. *Journal of clinical oncology : official journal of the American Society of Clinical Oncology* **32**, 3383-3390, doi:10.1200/JCO.2013.54.3553 (2014).

10 Terry, S. & Beltran, H. The many faces of neuroendocrine differentiation in prostate cancer progression. *Frontiers in oncology* **4**, 60, doi:10.3389/fonc.2014.00060 (2014).

11 Lee, J. K. *et al.* Systemic surfaceome profiling identifies target antigens for immune-based therapy in subtypes of advanced prostate cancer. *Proceedings of the National Academy of Sciences of the United States of America*, doi:10.1073/pnas.1802354115 (2018).

12 Shen, R. *et al.* Transdifferentiation of cultured human prostate cancer cells to a neuroendocrine cell phenotype in a hormone-depleted medium. *Urologic oncology* **3**, 67-75 (1997).

13 Palmgren, J. S., Karavadia, S. S. & Wakefield, M. R. Unusual and underappreciated: small cell carcinoma of the prostate. *Seminars in oncology* **34**, 22-29, doi:10.1053/j.seminoncol.2006.10.026 (2007).

14 Tan, H. L. *et al.* Rb loss is characteristic of prostatic small cell neuroendocrine carcinoma. *Clinical cancer research : an official journal of the American Association for Cancer Research* **20**, 890-903, doi:10.1158/1078-0432.CCR-13-1982 (2014).

15 Chen, H. *et al.* Pathogenesis of prostatic small cell carcinoma involves the inactivation of the P53 pathway. *Endocrine-related cancer* **19**, 321-331, doi:10.1530/ERC-11-0368 (2012).

16 Ku, S. Y. *et al.* Rb1 and Trp53 cooperate to suppress prostate cancer lineage plasticity, metastasis, and antiandrogen resistance. *Science* **355**, 78-83, doi:10.1126/science.aah4199 (2017).

17 Ham, W. S. *et al.* Pathological effects of prostate cancer correlate with neuroendocrine differentiation and PTEN expression after bicalutamide monotherapy. *The Journal of urology* **182**, 1378-1384, doi:10.1016/j.juro.2009.06.025 (2009).
18 Zou, M. *et al.* Transdifferentiation as a Mechanism of Treatment Resistance in a Mouse Model of Castration-Resistant Prostate Cancer. *Cancer discovery* **7**, 736-749, doi:10.1158/2159-8290.CD-16-1174 (2017).
19 Li, Y. *et al.* SRRM4 Drives Neuroendocrine Transdifferentiation of Prostate Adenocarcinoma Under Androgen Receptor Pathway Inhibition. *European urology*, doi:10.1016/j.eururo.2016.04.028 (2016).
20 Li, Y. *et al.* Establishment of a neuroendocrine prostate cancer model driven by the RNA splicing factor SRRM4. *Oncotarget* **8**, 66878-66888, doi:10.18632/oncotarget.19916 (2017).
21 Zhang, X. *et al.* SRRM4 Expression and the Loss of REST Activity May Promote the Emergence of the Neuroendocrine Phenotype in Castration-Resistant Prostate Cancer. *Clinical cancer research : an official journal of the American Association for Cancer Research* **21**, 4698-4708, doi:10.1158/1078-0432.CCR-15-0157 (2015).
22 Bishop, J. L. *et al.* The Master Neural Transcription Factor BRN2 is an Androgen Receptor Suppressed Driver of Neuroendocrine Differentiation in Prostate Cancer. *Cancer discovery*, doi:10.1158/2159-8290.CD-15-1263 (2016).
23 Kim, J. *et al.* FOXA1 inhibits prostate cancer neuroendocrine differentiation. *Oncogene* **36**, 4072-4080, doi:10.1038/onc.2017.50 (2017).
24 Akamatsu, S. *et al.* The Placental Gene PEG10 Promotes Progression of Neuroendocrine Prostate Cancer. *Cell reports* **12**, 922-936, doi:10.1016/j.celrep.2015.07.012 (2015).
25 Ci, X. *et al.* Heterochromatin protein 1alpha mediates development and aggressiveness of neuroendocrine prostate cancer. *Cancer research*, doi:10.1158/0008-5472.CAN-17-3677 (2018).
26 Lee, J. K. *et al.* N-Myc Drives Neuroendocrine Prostate Cancer Initiated from Human Prostate Epithelial Cells. *Cancer cell* **29**, 536-547, doi:10.1016/j.ccell.2016.03.001 (2016).
27 Dardenne, E. *et al.* N-Myc Induces an EZH2-Mediated Transcriptional Program Driving Neuroendocrine Prostate Cancer. *Cancer cell* **30**, 563-577, doi:10.1016/j.ccell.2016.09.005 (2016).
28 Mu, P. *et al.* SOX2 promotes lineage plasticity and antiandrogen resistance in TP53- and RB1-deficient prostate cancer. *Science* **355**, 84-88, doi:10.1126/science.aah4307 (2017).
29 Maina, P. K. *et al.* c-MYC drives histone demethylase PHF8 during neuroendocrine differentiation and in castration-resistant prostate cancer. *Oncotarget* **7**, 75585-75602, doi:10.18632/oncotarget.12310 (2016).
30 Maina, P. K. *et al.* Histone demethylase PHF8 regulates hypoxia signaling through HIF1alpha and H3K4me3. *Biochim Biophys Acta* **1860**, 1002-1012, doi:10.1016/j.bbagrm.2017.07.005 (2017).
31 Lapuk, A. V. *et al.* From sequence to molecular pathology, and a mechanism driving the neuroendocrine phenotype in prostate cancer. *The Journal of pathology* **227**, 286-297, doi:10.1002/path.4047 (2012).
32 Beltran, H. *et al.* Divergent clonal evolution of castration-resistant neuroendocrine prostate cancer. *Nature medicine* **22**, 298-305, doi:10.1038/nm.4045 (2016).
33 Viswanathan, V. S. *et al.* Dependency of a therapy-resistant state of cancer cells on a lipid peroxidase pathway. *Nature* **547**, 453-457, doi:10.1038/nature23007 (2017).
34 Gibb, E. A., Brown, C. J. & Lam, W. L. The functional role of long non-coding RNA in human carcinomas. *Molecular cancer* **10**, 38, doi:10.1186/1476-4598-10-38 (2011).
35 Cheetham, S. W., Gruhl, F., Mattick, J. S. & Dinger, M. E. Long noncoding RNAs and the genetics of cancer. *British journal of cancer* **108**, 2419-2425, doi:10.1038/bjc.2013.233 (2013).
36 Marchese, F. P., Raimondi, I. & Huarte, M. The multidimensional mechanisms of long noncoding RNA function. *Genome biology* **18**, 206, doi:10.1186/s13059-017-1348-2 (2017).

37 Sun, W., Yang, Y., Xu, C. & Guo, J. Regulatory mechanisms of long noncoding RNAs on gene expression in cancers. *Cancer Genet* **216-217**, 105-110, doi:10.1016/j.cancergen.2017.06.003 (2017).

38 Gutschner, T. & Diederichs, S. The hallmarks of cancer: a long non-coding RNA point of view. *RNA biology* **9**, 703-719, doi:10.4161/rna.20481 (2012).

39 Kondo, Y., Shinjo, K. & Katsushima, K. Long non-coding RNAs as an epigenetic regulator in human cancers. *Cancer Sci* **108**, 1927-1933, doi:10.1111/cas.13342 (2017).

40 Sahu, A., Singhal, U. & Chinnaiyan, A. M. Long noncoding RNAs in cancer: from function to translation. *Trends in cancer* **1**, 93-109, doi:10.1016/j.trecan.2015.08.010 (2015).

41 Bhan, A., Soleimani, M. & Mandal, S. S. Long Noncoding RNA and Cancer: A New Paradigm. *Cancer research* **77**, 3965-3981, doi:10.1158/0008-5472.CAN-16-2634 (2017).

42 Cheng, W., Zhang, Z. & Wang, J. Long noncoding RNAs: new players in prostate cancer. *Cancer letters* **339**, 8-14, doi:10.1016/j.canlet.2013.07.008 (2013).

43 Lin, D. *et al.* Identification of DEK as a potential therapeutic target for neuroendocrine prostate cancer. *Oncotarget* **6**, 1806-1820, doi:10.18632/oncotarget.2809 (2015).

44 Clermont, P. L. *et al.* Polycomb-mediated silencing in neuroendocrine prostate cancer. *Clinical epigenetics* **7**, 40, doi:10.1186/s13148-015-0074-4 (2015).

45 Trapnell, C. *et al.* Differential gene and transcript expression analysis of RNA-seq experiments with TopHat and Cufflinks. *Nature protocols* **7**, 562-578, doi:10.1038/nprot.2012.016 (2012).

46 Gutschner, T., Hammerle, M. & Diederichs, S. MALAT1 -- a paradigm for long noncoding RNA function in cancer. *J Mol Med (Berl)* **91**, 791-801, doi:10.1007/s00109-013-1028-y (2013).

47 Cross, D. S. & Burmester, J. K. Functional characterization of the GDEP promoter and three enhancer elements in retinoblastoma and prostate cell lines. *Med Oncol* **25**, 40-49, doi:10.1007/s12032-007-0038-4 (2008).

48 Reding, D. J. *et al.* Identification of a gene frequently mutated in prostate tumors. *Med Oncol* **18**, 179-187, doi:10.1385/MO:18:3:179 (2001).

49 Niknafs, Y. S. *et al.* The lncRNA landscape of breast cancer reveals a role for DSCAM-AS1 in breast cancer progression. *Nat Commun* **7**, 12791, doi:10.1038/ncomms12791 (2016).

50 Wang, O. *et al.* C-MYC-induced upregulation of lncRNA SNHG12 regulates cell proliferation, apoptosis and migration in triple-negative breast cancer. *Am J Transl Res* **9**, 533-545 (2017).

51 Chen, T., Yang, P. & He, Z. Y. Long non-coding RNA H19 can predict a poor prognosis and lymph node metastasis: a meta-analysis in human cancer. *Minerva Med* **107**, 251-258, doi:R10Y2016N04A0251 [pii] (2016).

52 Raveh, E., Matouk, I. J., Gilon, M. & Hochberg, A. The H19 Long non-coding RNA in cancer initiation, progression and metastasis - a proposed unifying theory. *Molecular cancer* **14**, 184, doi:10.1186/s12943-015-0458-2 (2015).

53 Li, H. *et al.* Long noncoding RNA linc00617 exhibits oncogenic activity in breast cancer. *Molecular carcinogenesis* **56**, 3-17, doi:10.1002/mc.22338 (2017).

54 Pisarek, H., Pawlikowski, M., Kunert-Radek, J., Kubiak, R. & Winczyk, K. SSTR1 and SSTR5 subtypes are the dominant forms of somatostatin receptor in neuroendocrine tumors. *Folia histochemica et cytobiologica* **48**, 142-147, doi:10.2478/v10042-008-0103-7 (2010).

55 Childs, A. *et al.* Expression of somatostatin receptors 2 and 5 in circulating tumour cells from patients with neuroendocrine tumours. *British journal of cancer* **115**, 1540-1547, doi:10.1038/bjc.2016.377 (2016).

56 Wyatt, A. W. *et al.* Heterogeneity in the inter-tumor transcriptome of high risk prostate cancer. *Genome biology* **15**, 426, doi:10.1186/s13059-014-0426-y (2014).

57 Ahlgren, G. *et al.* Regressive changes and neuroendocrine differentiation in prostate cancer after neoadjuvant hormonal treatment. *The Prostate* **42**, 274-279 (2000).

58 Wolf, D. A., Herzinger, T., Hermeking, H., Blaschke, D. & Horz, W. Transcriptional and posttranscriptional regulation of human androgen receptor expression by androgen. *Molecular endocrinology* **7**, 924-936, doi:10.1210/mend.7.7.8413317 (1993).

59 Cai, C. *et al.* Androgen receptor gene expression in prostate cancer is directly suppressed by the androgen receptor through recruitment of lysine-specific demethylase 1. *Cancer cell* **20**, 457-471, doi:10.1016/j.ccr.2011.09.001 (2011).

60 Knuuttila, M. *et al.* Castration induces up-regulation of intratumoral androgen biosynthesis and androgen receptor expression in an orthotopic VCaP human prostate cancer xenograft model. *The American journal of pathology* **184**, 2163-2173, doi:10.1016/j.ajpath.2014.04.010 (2014).

61 Wang, Q. *et al.* A novel cell cycle-associated lncRNA, HOXA11-AS, is transcribed from the 5-prime end of the HOXA transcript and is a biomarker of progression in glioma. *Cancer letters* **373**, 251-259, doi:10.1016/j.canlet.2016.01.039 (2016).

62 Sun, M. *et al.* LncRNA HOXA11-AS Promotes Proliferation and Invasion of Gastric Cancer by Scaffolding the Chromatin Modification Factors PRC2, LSD1, and DNMT1. *Cancer research* **76**, 6299-6310, doi:10.1158/0008-5472.CAN-16-0356 (2016).

63 Misawa, A., Takayama, K., Urano, T. & Inoue, S. Androgen-induced Long Noncoding RNA (lncRNA) SOCS2-AS1 Promotes Cell Growth and Inhibits Apoptosis in Prostate Cancer Cells. *J Biol Chem* **291**, 17861-17880, doi:10.1074/jbc.M116.718536 (2016).

64 Zhao, W., Luo, J. & Jiao, S. Comprehensive characterization of cancer subtype associated long non-coding RNAs and their clinical implications. *Sci Rep* **4**, 6591, doi:10.1038/srep06591 (2014).

65 Li, Z., Yu, X. & Shen, J. ANRIL: a pivotal tumor suppressor long non-coding RNA in human cancers. *Tumour Biol* **37**, 5657-5661, doi:10.1007/s13277-016-4808-5 (2016).

66 Aguilo, F., Zhou, M. M. & Walsh, M. J. Long noncoding RNA, polycomb, and the ghosts haunting INK4b-ARF-INK4a expression. *Cancer research* **71**, 5365-5369, doi:10.1158/0008-5472.CAN-10-4379 (2011).

67 Kotake, Y. *et al.* Long non-coding RNA ANRIL is required for the PRC2 recruitment to and silencing of p15(INK4B) tumor suppressor gene. *Oncogene* **30**, 1956-1962, doi:10.1038/onc.2010.568 (2011).

68 Yap, K. L. *et al.* Molecular interplay of the noncoding RNA ANRIL and methylated histone H3 lysine 27 by polycomb CBX7 in transcriptional silencing of INK4a. *Molecular cell* **38**, 662-674, doi:10.1016/j.molcel.2010.03.021 (2010).

69 Yu, W. *et al.* Epigenetic silencing of tumour suppressor gene p15 by its antisense RNA. *Nature* **451**, 202-206, doi:10.1038/nature06468 (2008).

70 Wang, S. H. *et al.* Long non-coding RNA H19 regulates FOXM1 expression by competitively binding endogenous miR-342-3p in gallbladder cancer. *J Exp Clin Cancer Res* **35**, 160, doi:10.1186/s13046-016-0436-6 (2016).

71 Xu, T. P. *et al.* Decreased expression of the long non-coding RNA FENDRR is associated with poor prognosis in gastric cancer and FENDRR regulates gastric cancer cell metastasis by affecting fibronectin1 expression. *Journal of hematology & oncology* **7**, 63, doi:10.1186/s13045-014-0063-7 (2014).

72 Fernando, T. R. *et al.* The lncRNA CASC15 regulates SOX4 expression in RUNX1-rearranged acute leukemia. *Molecular cancer* **16**, 126, doi:10.1186/s12943-017-0692-x (2017).

73 Diskin, S. J. *et al.* Common variation at 6q16 within HACE1 and LIN28B influences susceptibility to neuroblastoma. *Nat Genet* **44**, 1126-1130, doi:10.1038/ng.2387 (2012).

74 Russell, M. R. *et al.* CASC15-S Is a Tumor Suppressor lncRNA at the 6p22 Neuroblastoma Susceptibility Locus. *Cancer research* **75**, 3155-3166, doi:10.1158/0008-5472.CAN-14-3613 (2015).

75 Nakakura, E. K. *et al.* Mammalian Scratch: a neural-specific Snail family transcriptional repressor. *Proceedings of the National Academy of Sciences of the United States of America* **98**, 4010-4015, doi:10.1073/pnas.051014098 (2001).

76 Ishii, J. *et al.* Class III/IV POU transcription factors expressed in small cell lung cancer cells are involved in proneural/neuroendocrine differentiation. *Pathol Int* **64**, 415-422, doi:10.1111/pin.12198 (2014).

77 Beltran, H. *et al.* Impact of therapy on genomics and transcriptomics in high-risk prostate cancer treated with neoadjuvant docetaxel and androgen deprivation therapy. *Clinical cancer research : an official journal of the American Association for Cancer Research*, doi:10.1158/1078-0432.CCR-17-1034 (2017).

78 Manohar, C. F., Furtado, M. R., Salwen, H. R. & Cohn, S. L. Hox gene expression in differentiating human neuroblastoma cells. *Biochem Mol Biol Int* **30**, 733-741 (1993).

79 Manohar, C. F., Salwen, H. R., Furtado, M. R. & Cohn, S. L. Up-regulation of HOXC6, HOXD1, and HOXD8 homeobox gene expression in human neuroblastoma cells following chemical induction of differentiation. *Tumour Biol* **17**, 34-47 (1996).

80 Hessenkemper, W. & Baniahmad, A. Targeting heat shock proteins in prostate cancer. *Curr Med Chem* **20**, 2731-2740, doi:CMC-EPUB-20130315-19 [pii] (2013).

81 Azad, A. A., Zoubeidi, A., Gleave, M. E. & Chi, K. N. Targeting heat shock proteins in metastatic castration-resistant prostate cancer. *Nature reviews. Urology* **12**, 26-36, doi:10.1038/nrurol.2014.320 (2015).

82 Wang, B., Lee, C. W., Witt, A., Thakkar, A. & Ince, T. A. Heat shock factor 1 induces cancer stem cell phenotype in breast cancer cell lines. *Breast Cancer Res Treat* **153**, 57-66, doi:10.1007/s10549-015-3521-1 (2015).

83 Chen, W. M. *et al.* Long intergenic non-coding RNA 00152 promotes tumor cell cycle progression by binding to EZH2 and repressing p15 and p21 in gastric cancer. *Oncotarget* **7**, 9773-9787, doi:10.18632/oncotarget.6949 (2016).

84 Chen, Q. N. *et al.* Long intergenic non-coding RNA 00152 promotes lung adenocarcinoma proliferation via interacting with EZH2 and repressing IL24 expression. *Molecular cancer* **16**, 17, doi:10.1186/s12943-017-0581-3 (2017).

85 Jin, H. J., Zhao, J. C., Wu, L., Kim, J. & Yu, J. Cooperativity and equilibrium with FOXA1 define the androgen receptor transcriptional program. *Nat Commun* **5**, 3972, doi:10.1038/ncomms4972 (2014).

86 Jin, H. J., Zhao, J. C., Ogden, I., Bergan, R. C. & Yu, J. Androgen receptor-independent function of FoxA1 in prostate cancer metastasis. *Cancer research* **73**, 3725-3736, doi:10.1158/0008-5472.CAN-12-3468 (2013).

87 Yang, J. *et al.* Twist, a master regulator of morphogenesis, plays an essential role in tumor metastasis. *Cell* **117**, 927-939, doi:10.1016/j.cell.2004.06.006 (2004).

88 Wang, J. *et al.* The Aurora-A-Twist1 axis promotes highly aggressive phenotypes in pancreatic carcinoma. *J Cell Sci* **130**, 1078-1093, doi:10.1242/jcs.196790 (2017).

89 Galvan, J. A. *et al.* Epithelial-mesenchymal transition markers in the differential diagnosis of gastroenteropancreatic neuroendocrine tumors. *Am J Clin Pathol* **140**, 61-72, doi:10.1309/AJCPIV40ISTBXRAX (2013).

90 Fendrich, V. *et al.* Epithelial-mesenchymal transition is a critical step in tumorgenesis of pancreatic neuroendocrine tumors. *Cancers (Basel)* **4**, 281-294, doi:10.3390/cancers4010281 (2012).

91 Eide, T., Ramberg, H., Glackin, C., Tindall, D. & Tasken, K. A. TWIST1, A novel androgen-regulated gene, is a target for NKX3-1 in prostate cancer cells. *Cancer Cell Int* **13**, 4, doi:10.1186/1475-2867-13-4 (2013).

92 Ainechi, S. *et al.* Paired Box 5 (PAX5) Expression in Poorly Differentiated Neuroendocrine Carcinoma of the Gastrointestinal and Pancreatobiliary Tract: Diagnostic and Potentially Therapeutic Implications. *Appl Immunohistochem Mol Morphol*, doi:10.1097/PAI.0000000000000473 (2016).

93 Song, J. *et al.* Expression patterns of PAX5, c-Met, and paxillin in neuroendocrine tumors of the lung. *Arch Pathol Lab Med* **134**, 1702-1705, doi:10.1043/2009-0664-OAR1.1 (2010).

94 Czapiewski, P. *et al.* Expression pattern of ISL-1, TTF-1 and PAX5 in olfactory neuroblastoma. *Pol J Pathol* **67**, 130-135, doi:28055 [pii] (2016).

95 Kanteti, R. *et al.* PAX5 is expressed in small-cell lung cancer and positively regulates c-Met transcription. *Lab Invest* **89**, 301-314, doi:10.1038/labinvest.2008.168 (2009).

96 Walter, R. F. *et al.* SOX4, SOX11 and PAX6 mRNA expression was identified as a (prognostic) marker for the aggressiveness of neuroendocrine tumors of the lung by using next-generation expression analysis (NanoString). *Future Oncol* **11**, 1027-1036, doi:10.2217/fon.15.18 (2015).

97 Quandt, K., Frech, K., Karas, H., Wingender, E. & Werner, T. MatInd and MatInspector: new fast and versatile tools for detection of consensus matches in nucleotide sequence data. *Nucleic acids research* **23**, 4878-4884, doi:5s0483 [pii] (1995).

98 Cartharius, K. *et al.* MatInspector and beyond: promoter analysis based on transcription factor binding sites. *Bioinformatics* **21**, 2933-2942, doi:10.1093/bioinformatics/bti473 (2005).

99 Markoff, A. *Analytical tools for DNA, genes and genomes : nuts & bolts*. 1st edn, (DNA Press, 2005).

100 Yang, L. *et al.* lncRNA-dependent mechanisms of androgen-receptor-regulated gene activation programs. *Nature* **500**, 598-602, doi:10.1038/nature12451 (2013).

101 Cui, Z. *et al.* The prostate cancer-up-regulated long noncoding RNA PlncRNA-1 modulates apoptosis and proliferation through reciprocal regulation of androgen receptor. *Urologic oncology* **31**, 1117-1123, doi:10.1016/j.urolonc.2011.11.030 (2013).

102 Crea, F. *et al.* Identification of a long non-coding RNA as a novel biomarker and potential therapeutic target for metastatic prostate cancer. *Oncotarget* (2014).

103 Malik, R. *et al.* The lncRNA PCAT29 Inhibits Oncogenic Phenotypes in Prostate Cancer. *Molecular cancer research : MCR*, doi:10.1158/1541-7786.MCR-14-0257 (2014).

104 Wan, X. *et al.* Identification of androgen-responsive lncRNAs as diagnostic and prognostic markers for prostate cancer. *Oncotarget*, doi:10.18632/oncotarget.11391 (2016).

105 Lu, W. *et al.* KLK31P is a novel androgen regulated and transcribed pseudogene of kallikreins that is expressed at lower levels in prostate cancer cells than in normal prostate cells. *The Prostate* **66**, 936-944, doi:10.1002/pros.20382 (2006).

106 Misawa, A. *et al.* Androgen-induced lncRNA POTEF-AS1 regulates apoptosis-related pathway to facilitate cell survival in prostate cancer cells. *Cancer science* **108**, 373-379, doi:10.1111/cas.13151 (2017).

107 Takayama, K. *et al.* Androgen-responsive long noncoding RNA CTBP1-AS promotes prostate cancer. *The EMBO journal* **32**, 1665-1680, doi:10.1038/emboj.2013.99 (2013).

108 Seifert, A., Werheid, D. F., Knapp, S. M. & Tobiasch, E. Role of Hox genes in stem cell differentiation. *World J Stem Cells* **7**, 583-595, doi:10.4252/wjsc.v7.i3.583 (2015).

109 Cho, K. H. *et al.* STAT3 mediates TGF-beta1-induced TWIST1 expression and prostate cancer invasion. *Cancer letters* **336**, 167-173, doi:10.1016/j.canlet.2013.04.024 (2013).

110 Yang, X., Duan, B. & Zhou, X. Long non-coding RNA FOXD2-AS1 functions as a tumor promoter in colorectal cancer by regulating EMT and Notch signaling pathway. *Eur Rev Med Pharmacol Sci* **21**, 3586-3591, doi:13269 [pii] (2017).

111 Zhou, W. *et al.* The lncRNA H19 mediates breast cancer cell plasticity during EMT and MET plasticity by differentially sponging miR-200b/c and let-7b. *Sci Signal* **10**, doi:10.1126/scisignal.aak9557 (2017).

112 Bauderlique-Le Roy, H. *et al.* Enrichment of Human Stem-Like Prostate Cells with s-SHIP Promoter Activity Uncovers a Role in Stemness for the Long Noncoding RNA H19. *Stem Cells Dev* **24**, 1252-1262, doi:10.1089/scd.2014.0386 (2015).

113 Zhao, J. *et al.* Long non-coding RNA Linc00152 is involved in cell cycle arrest, apoptosis, epithelial to mesenchymal transition, cell migration and invasion in gastric cancer. *Cell Cycle* **14**, 3112-3123, doi:10.1080/15384101.2015.1078034 (2015).

114 Emmrich, S. *et al.* LincRNAs MONC and MIR100HG act as oncogenes in acute megakaryoblastic leukemia. *Molecular cancer* **13**, 171, doi:10.1186/1476-4598-13-171 (2014).

115 Yan, K., Tian, J., Shi, W., Xia, H. & Zhu, Y. LncRNA SNHG6 is Associated with Poor Prognosis of Gastric Cancer and Promotes Cell Proliferation and EMT through Epigenetically Silencing p27 and Sponging miR-101-3p. *Cell Physiol Biochem* **42**, 999-1012, doi:10.1159/000478682 (2017).

116 Erho, N. *et al.* Discovery and validation of a prostate cancer genomic classifier that predicts early metastasis following radical prostatectomy. *PloS one* **8**, e66855, doi:10.1371/journal.pone.0066855 (2013).

117 Karnes, R. J. *et al.* Validation of a genomic classifier that predicts metastasis following radical prostatectomy in an at risk patient population. *The Journal of urology* **190**, 2047-2053, doi:10.1016/j.juro.2013.06.017 (2013).

118 Beltran, H. *et al.* Challenges in recognizing treatment-related neuroendocrine prostate cancer. *Journal of clinical oncology : official journal of the American Society of Clinical Oncology* **30**, e386-389, doi:10.1200/JCO.2011.41.5166 (2012).

119 Terai, G., Iwakiri, J., Kameda, T., Hamada, M. & Asai, K. Comprehensive prediction of lncRNA-RNA interactions in human transcriptome. *BMC genomics* **17 Suppl 1**, 12, doi:10.1186/s12864-015-2307-5 (2016).

120 Kiryu, H. *et al.* A detailed investigation of accessibilities around target sites of siRNAs and miRNAs. *Bioinformatics* **27**, 1788-1797, doi:10.1093/bioinformatics/btr276 (2011).

121 Busch, A., Richter, A. S. & Backofen, R. IntaRNA: efficient prediction of bacterial sRNA targets incorporating target site accessibility and seed regions. *Bioinformatics* **24**, 2849-2856, doi:10.1093/bioinformatics/btn544 (2008).

122 Kato, Y. *et al.* RactIP: fast and accurate prediction of RNA-RNA interaction using integer programming. *Bioinformatics* **26**, i460-466, doi:10.1093/bioinformatics/btq372 (2010).

123 Helpap, B., Kollermann, J. & Oehler, U. Neuroendocrine differentiation in prostatic carcinomas: histogenesis, biology, clinical relevance, and future therapeutical perspectives. *Urologia internationalis* **62**, 133-138, doi:30376 (1999).

124 Hirano, D., Okada, Y., Minei, S., Takimoto, Y. & Nemoto, N. Neuroendocrine differentiation in hormone refractory prostate cancer following androgen deprivation therapy. *European urology* **45**, 586-592; discussion 592, doi:10.1016/j.eururo.2003.11.032 (2004).

125 Berruti, A. *et al.* Chromogranin A expression in patients with hormone naive prostate cancer predicts the development of hormone refractory disease. *The Journal of urology* **178**, 838-843; quiz 1129, doi:10.1016/j.juro.2007.05.018 (2007).

126 Aggarwal, R., Zhang, T., Small, E. J. & Armstrong, A. J. Neuroendocrine prostate cancer: subtypes, biology, and clinical outcomes. *Journal of the National Comprehensive Cancer Network : JNCCN* **12**, 719-726 (2014).

127 Yuan, T. C., Veeramani, S. & Lin, M. F. Neuroendocrine-like prostate cancer cells: neuroendocrine transdifferentiation of prostate adenocarcinoma cells. *Endocrine-related cancer* **14**, 531-547, doi:10.1677/ERC-07-0061 (2007).

128 Terry, S. *et al.* Cross modulation between the androgen receptor axis and protocadherin-PC in mediating neuroendocrine transdifferentiation and therapeutic resistance of prostate cancer. *Neoplasia* **15**, 761-772 (2013).

129 Huss, W. J., Gregory, C. W. & Smith, G. J. Neuroendocrine cell differentiation in the CWR22 human prostate cancer xenograft: association with tumor cell proliferation prior to recurrence. *The Prostate* **60**, 91-97, doi:10.1002/pros.20032 (2004).

130 Vashchenko, N. & Abrahamsson, P. A. Neuroendocrine differentiation in prostate cancer: implications for new treatment modalities. *European urology* **47**, 147-155, doi:10.1016/j.eururo.2004.09.007 (2005).

131 Aparicio, A. & Tzelepi, V. Neuroendocrine (small-cell) carcinomas: why they teach us essential lessons about prostate cancer. *Oncology* **28**, 831-838 (2014).

132 Beltran, H. *et al.* Aggressive variants of castration-resistant prostate cancer. *Clinical cancer research : an official journal of the American Association for Cancer Research* **20**, 2846-2850, doi:10.1158/1078-0432.CCR-13-3309 (2014).

133 Bishop, J. L., Davies, A., Ketola, K. & Zoubeidi, A. Regulation of tumor cell plasticity by the androgen receptor in prostate cancer. *Endocrine-related cancer* **22**, R165-182, doi:10.1530/ERC-15-0137 (2015).

134 Davies, A. H., Beltran, H. & Zoubeidi, A. Cellular plasticity and the neuroendocrine phenotype in prostate cancer. *Nature reviews. Urology*, doi:10.1038/nrurol.2018.22 (2018).

135 Zhang, W. *et al.* Targeting the MYCN-PARP-DNA Damage Response Pathway inNeuroendocrine Prostate Cancer. *Clinical cancer research : an official journal of the American Association for Cancer Research*, doi:10.1158/1078-0432.CCR-17-1872 (2017).

136 Wang, C. *et al.* Blocking the Feedback Loop between Neuroendocrine Differentiation and Macrophages Improves the Therapeutic Effects of Enzalutamide (MDV3100) on Prostate Cancer. *Clinical cancer research : an official journal of the American Association for Cancer Research*, doi:10.1158/1078-0432.CCR-17-2446 (2017).

137 Brzezniak, C., Oronsky, B. & Aggarwal, R. A Complete Metabolic Response of Metastatic Castration-resistant Neuroendocrine Carcinoma of the Prostate After Treatment with RRx-001 and Reintroduced Platinum Doublets. *European urology*, doi:10.1016/j.eururo.2017.09.010 (2017).

138 Thakur, M. K. *et al.* Phase I Trial of the Combination of Docetaxel, Prednisone, and Pasireotide in Metastatic Castrate-Resistant Prostate Cancer. *Clinical genitourinary cancer*, doi:10.1016/j.clgc.2018.01.019 (2018).

139 Akamatsu, S., Inoue, T., Ogawa, O. & Gleave, M. E. Clinical and molecular features of treatment-related neuroendocrine prostate cancer. *International journal of urology : official journal of the Japanese Urological Association*, doi:10.1111/iju.13526 (2018).

140 Stone, L. Prostate cancer: A novel mechanism of neuroendocrine transdifferentiation. *Nature reviews. Urology*, doi:10.1038/nrurol.2018.40 (2018).

141 Huarte, M. The emerging role of lncRNAs in cancer. *Nature medicine* **21**, 1253-1261, doi:10.1038/nm.3981 (2015).

142 Szafranski, P. *et al.* Small noncoding differentially methylated copy-number variants, including lncRNA genes, cause a lethal lung developmental disorder. *Genome research* **23**, 23-33, doi:10.1101/gr.141887.112 (2013).

143 White, N. M. *et al.* Transcriptome sequencing reveals altered long intergenic non-coding RNAs in lung cancer. *Genome biology* **15**, 429, doi:10.1186/s13059-014-0429-8 (2014).

144 Camacho, N. *et al.* Appraising the relevance of DNA copy number loss and gain in prostate cancer using whole genome DNA sequence data. *PLoS genetics* **13**, e1007001, doi:10.1371/journal.pgen.1007001 (2017).

145 Schuettengruber, B., Chourrout, D., Vervoort, M., Leblanc, B. & Cavalli, G. Genome regulation by polycomb and trithorax proteins. *Cell* **128**, 735-745, doi:10.1016/j.cell.2007.02.009 (2007).

146 Khalil, A. M. *et al.* Many human large intergenic noncoding RNAs associate with chromatin-modifying complexes and affect gene expression. *Proceedings of the National Academy of Sciences of the United States of America* **106**, 11667-11672, doi:10.1073/pnas.0904715106 (2009).

147 Rinn, J. L. *et al.* Functional demarcation of active and silent chromatin domains in human HOX loci by noncoding RNAs. *Cell* **129**, 1311-1323, doi:10.1016/j.cell.2007.05.022 (2007).

148 Li, J. *et al.* TANRIC: An Interactive Open Platform to Explore the Function of lncRNAs in Cancer. *Cancer research* **75**, 3728-3737, doi:10.1158/0008-5472.CAN-15-0273 (2015).

149 Ren, X. *et al.* FOXF1 transcription factor is required for formation of embryonic vasculature by regulating VEGF signaling in endothelial cells. *Circulation research* **115**, 709-720, doi:10.1161/CIRCRESAHA.115.304382 (2014).

150 Tamura, M. *et al.* Forkhead transcription factor FOXF1 is a novel target gene of the p53 family and regulates cancer cell migration and invasiveness. *Oncogene* **33**, 4837-4846, doi:10.1038/onc.2013.427 (2014).

151 Sekaric, P., Shamanin, V. A., Luo, J. & Androphy, E. J. hAda3 regulates p14ARF-induced p53 acetylation and senescence. *Oncogene* **26**, 6261-6268, doi:10.1038/sj.onc.1210462 (2007).

152 Wang, T. *et al.* hADA3 is required for p53 activity. *The EMBO journal* **20**, 6404-6413, doi:10.1093/emboj/20.22.6404 (2001).

153 Lin, N. *et al.* An evolutionarily conserved long noncoding RNA TUNA controls pluripotency and neural lineage commitment. *Molecular cell* **53**, 1005-1019, doi:10.1016/j.molcel.2014.01.021 (2014).

154 Lee, H. J. *et al.* Epigenetic alteration of imprinted genes during neural differentiation of germline-derived pluripotent stem cells. *Epigenetics* **11**, 177-183, doi:10.1080/15592294.2016.1146852 (2016).

155 Tsuta, K., Wistuba, II & Moran, C. A. Differential expression of somatostatin receptors 1-5 in neuroendocrine carcinoma of the lung. *Pathology, research and practice* **208**, 470-474, doi:10.1016/j.prp.2012.05.014 (2012).

156 Muscarella, L. A. *et al.* Gene expression of somatostatin receptor subtypes SSTR2a, SSTR3 and SSTR5 in peripheral blood of neuroendocrine lung cancer affected patients. *Cellular oncology* **34**, 435-441, doi:10.1007/s13402-011-0025-9 (2011).

157 Squires, M. H., 3rd *et al.* Octreoscan Versus FDG-PET for Neuroendocrine Tumor Staging: A Biological Approach. *Annals of surgical oncology* **22**, 2295-2301, doi:10.1245/s10434-015-4471-x (2015).

158 Narayanan, S. & Kunz, P. L. Role of Somatostatin Analogues in the Treatment of Neuroendocrine Tumors. *Hematology/oncology clinics of North America* **30**, 163-177, doi:10.1016/j.hoc.2015.09.008 (2016).

159 Sharma, K., Patel, Y. C. & Srikant, C. B. C-terminal region of human somatostatin receptor 5 is required for induction of Rb and G1 cell cycle arrest. *Molecular endocrinology* **13**, 82-90, doi:10.1210/mend.13.1.0220 (1999).

160 Coffey, K. *et al.* The lysine demethylase, KDM4B, is a key molecule in androgen receptor signalling and turnover. *Nucleic acids research* **41**, 4433-4446, doi:10.1093/nar/gkt106 (2013).

161 Yang, J. *et al.* The role of histone demethylase KDM4B in Myc signaling in neuroblastoma. *Journal of the National Cancer Institute* **107**, djv080, doi:10.1093/jnci/djv080 (2015).

162 Mo, F. *et al.* Stromal Gene Expression is Predictive for Metastatic Primary Prostate Cancer. *European urology*, doi:10.1016/j.eururo.2017.02.038 (2017).

163 Tsai, H. *et al.* Cyclin D1 Loss Distinguishes Prostatic Small-Cell Carcinoma from Most Prostatic Adenocarcinomas. *Clinical cancer research : an official journal of the American Association for Cancer Research* **21**, 5619-5629, doi:10.1158/1078-0432.CCR-15-0744 (2015).

164 Gao, J. *et al.* Integrative analysis of complex cancer genomics and clinical profiles using the cBioPortal. *Sci Signal* **6**, pl1, doi:10.1126/scisignal.2004088 (2013).

165 Cerami, E. *et al.* The cBio cancer genomics portal: an open platform for exploring multidimensional cancer genomics data. *Cancer discovery* **2**, 401-404, doi:10.1158/2159-8290.CD-12-0095 (2012).

166 Piccolo, S. R. *et al.* A single-sample microarray normalization method to facilitate personalized-medicine workflows. *Genomics* **100**, 337-344, doi:10.1016/j.ygeno.2012.08.003 (2012).

167 Iyer, M. K. *et al.* The landscape of long noncoding RNAs in the human transcriptome. *Nat Genet* **47**, 199-208, doi:10.1038/ng.3192 (2015).

168 Eisenberg, E. & Levanon, E. Y. Human housekeeping genes, revisited. *Trends Genet* **29**, 569-574, doi:10.1016/j.tig.2013.05.010 (2013).

169 Lai, D., Proctor, J. R., Zhu, J. Y. & Meyer, I. M. R-CHIE: a web server and R package for visualizing RNA secondary structures. *Nucleic acids research* **40**, e95, doi:10.1093/nar/gks241 (2012).

170 Mathews, D. H. *et al.* Incorporating chemical modification constraints into a dynamic programming algorithm for prediction of RNA secondary structure. *Proceedings of the National Academy of Sciences of the United States of America* **101**, 7287-7292, doi:10.1073/pnas.0401799101 (2004).

171 Gruber, A. R., Lorenz, R., Bernhart, S. H., Neubock, R. & Hofacker, I. L. The Vienna RNA websuite. *Nucleic acids research* **36**, W70-74, doi:10.1093/nar/gkn188 (2008).

172 Ho Sui, S. J. *et al.* oPOSSUM: identification of over-represented transcription factor binding sites in co-expressed genes. *Nucleic acids research* **33**, 3154-3164, doi:10.1093/nar/gki624 (2005).

173 Ramnarine VR; Alshalalfa, M; Mo F; Nabavi, N; Erho, N; Takhar, M; Shukin, R; Brahmbhatt, S; Nouri, M; Lin, D; Tsai, H; Lotan, TL; Karnes, RJ; Rubin, MA; Kobelev, M; Zoubeidi, A; Gleave, ME; Volik, SV; Wyatt, AW; Beltran, H; Davicioni, E; Wang, Y; Collins, CC. Supporting data for "The Long Noncoding RNA Landscape of Neuroendocrine Prostate Cancer and its Clinical Implications" (2018) GigaScience Database. http://doi.org/10.5524/100443

TABLE 1

Name	Model System	Model System Name	Source	Phenotype	Resistance				MOLECULAR CHARACTERICS									
					AN	TE	EZ	BI	AR	PSA	SYP	SPINK1	ERG	TMPRSS2-ERG	PTEN	PTEN GENE	p53	RB
LTL313B	Xenograft	313	Primary PCa	AD	-	-	-	-	+	+	-	-	+	+	-	-/-	M	WT
LTL313BR	Xenograft	313	LTL313B	CRPC	-	+	+	+	+	+	-	-	+	+	-	-/-	M	WT
LTL418	Xenograft	418	Primary PCa	AD	-	-	-	-	+	+	-	+	-	-	+	+/+	WT	WT
LTL418BR	Xenograft	418	LTL418B	CRPC	-	+	-	-	+	+	-	--------------- Same as parental xenograft above ---------------						
LTL331-3	Xenograft	331	Primary PCa	AD	-	-	-	-	+	+	-	-	+	+	-	-/-	M	M
LTL331-7	Xenograft	331	Primary PCa	AD	-	-	-	-	+	+	-	-	+	+	-	-/-	M	M
LTL331-5-8week	Xenograft	331	LTL331-5	AD	-	-	-	-	+	-	-	--------------- Same as parental xenograft above ---------------						
LTL331-5-12week	Xenograft	331	LTL331-5	AD	-	-	-	-	+	-	-							
LTL331-3-R	Xenograft	331	LTL331-3	NEPC	-	+	-	-	-	-	+	-	-	+	-	-/-	M	M
LTL331-7-R	Xenograft	331	LTL313-3-R	NEPC	-	+	-	-	-	-	+	-	-	+	-	-/-	M	M

 Click here to download Table T2.xlsx

TABLE 2

				Treatment Status				Clinical Charertistics							
								Gleason Grade			End Points (For GRID Data Only)				
**Institute**	**Cohort Name**	**Clinical Group**	**TOTAL**	**NAIVE**	**NHT**	**ADT**	**RT**	**-6**	**7**	**8+**	**BCR**	**MET**	**PCSM**	**+RMET+ADT**	**-RMET+ADT**
VPC	VPC	AD-NAIVE	56	56	0	0	0	23	0	33					
VPC	VPC	AD-NHT	14	0	14	0	0	0	0	14					
VPC	VPC	NEPC[1]	5	0	1	5	0	0	0	5					
VPC	VPC	CRPC	5	3	2	5	0	1	1	3					
WCM	RUBIN	NEPC	7					NA	NA	NA					
WCM	RUBIN	AD	30					2	23	5					
JHSM	LOTAN	AD[2]	17					0	0	12					
JHSM	LOTAN	NEPC[2]	16					NA	NA	NA					
GRID	MCI	AD	545	0	0	124	54	63	271	211	388	212	132	11	113
GRID	MCII	AD	232	0	0	77	24	18	117	97	124	75	34	19	24
		**TOTAL**	**927**	**59**	**17**	**211**	**78**	**107**	**412**	**380**	**512**	**287**	**166**	**30**	**137**

Figure 1

Click here to download Figure F1.pdf

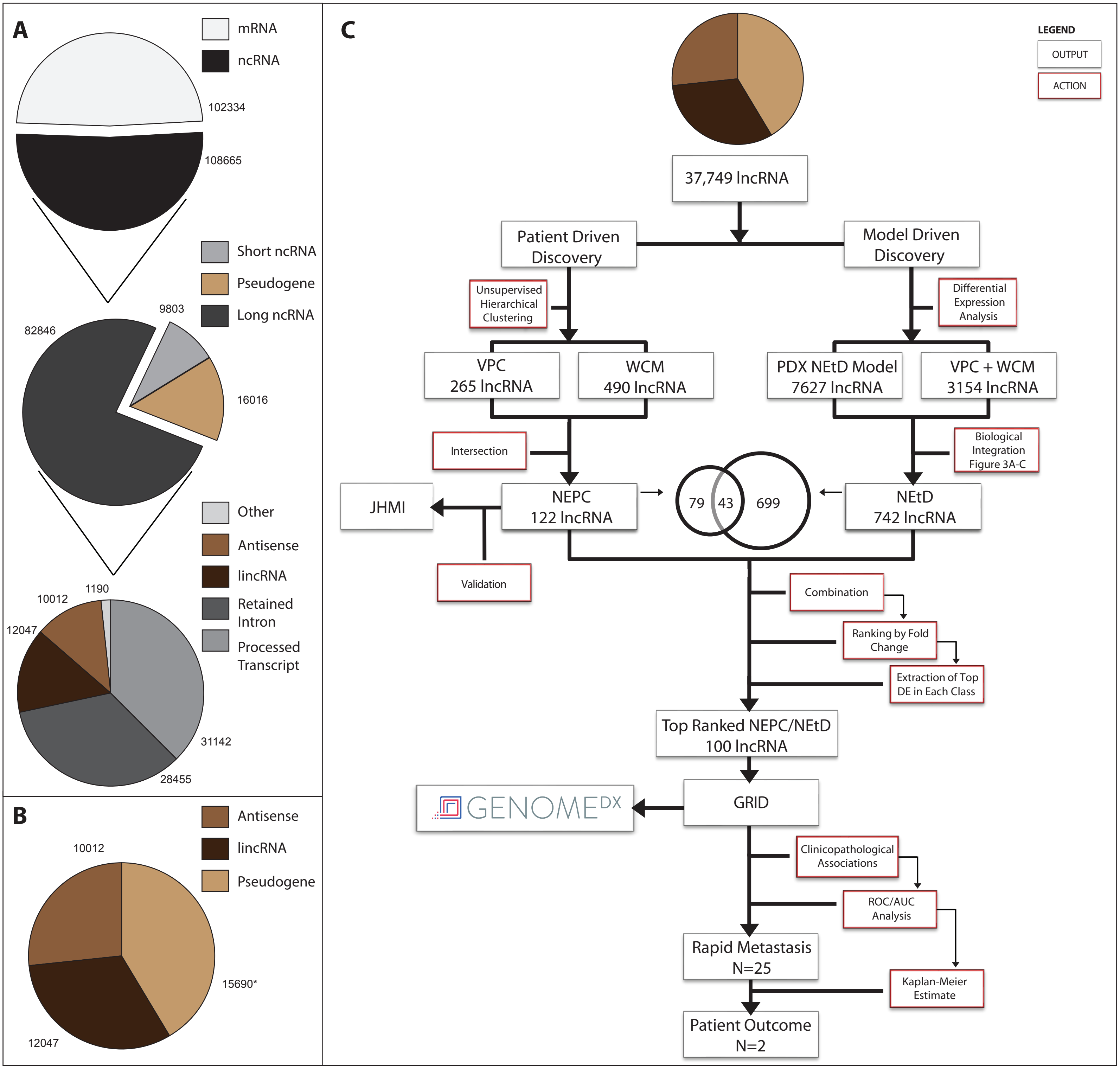

Figure 2

Click here to download Figure F2.pdf

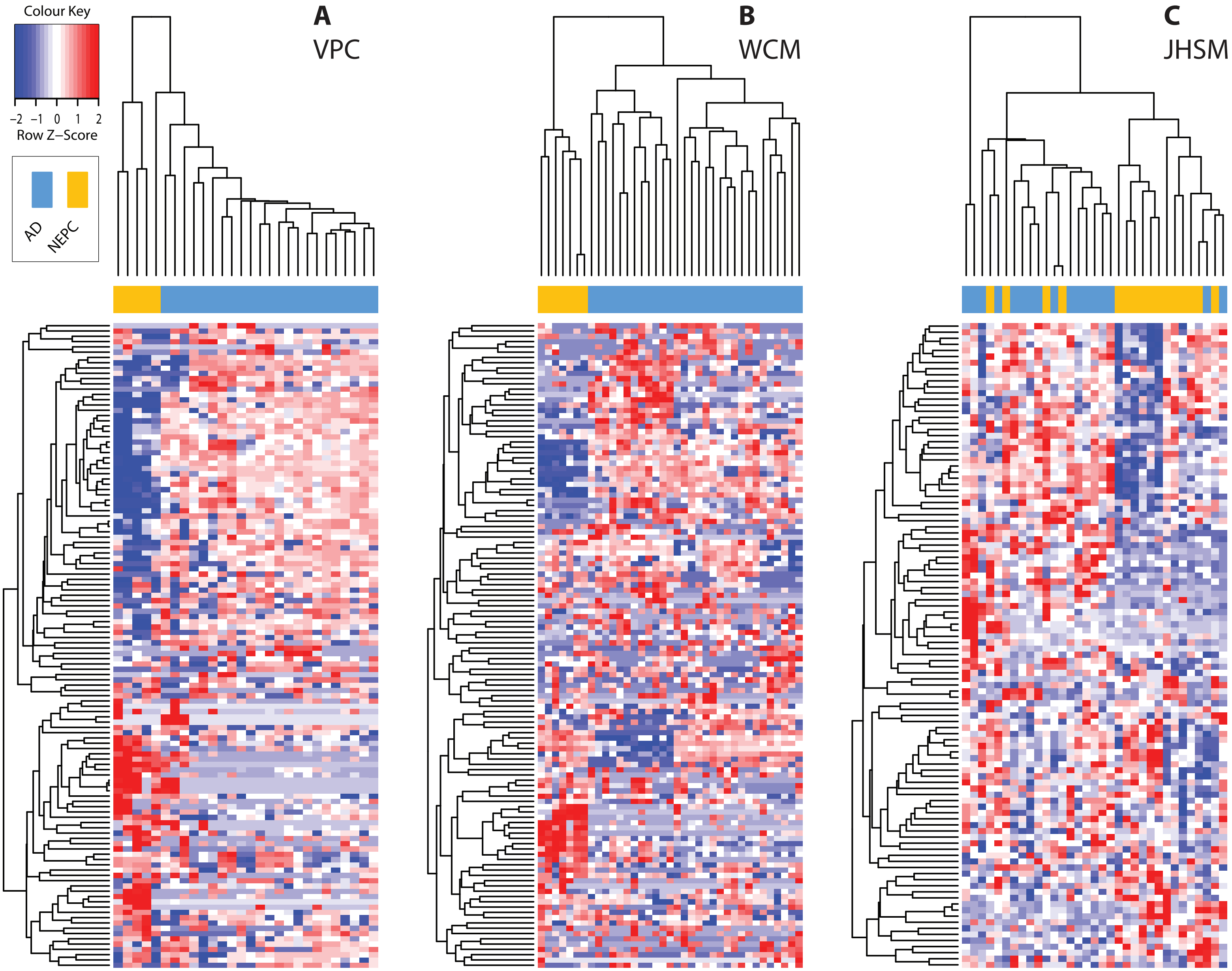

Figure 3

Click here to download Figure F3.pdf

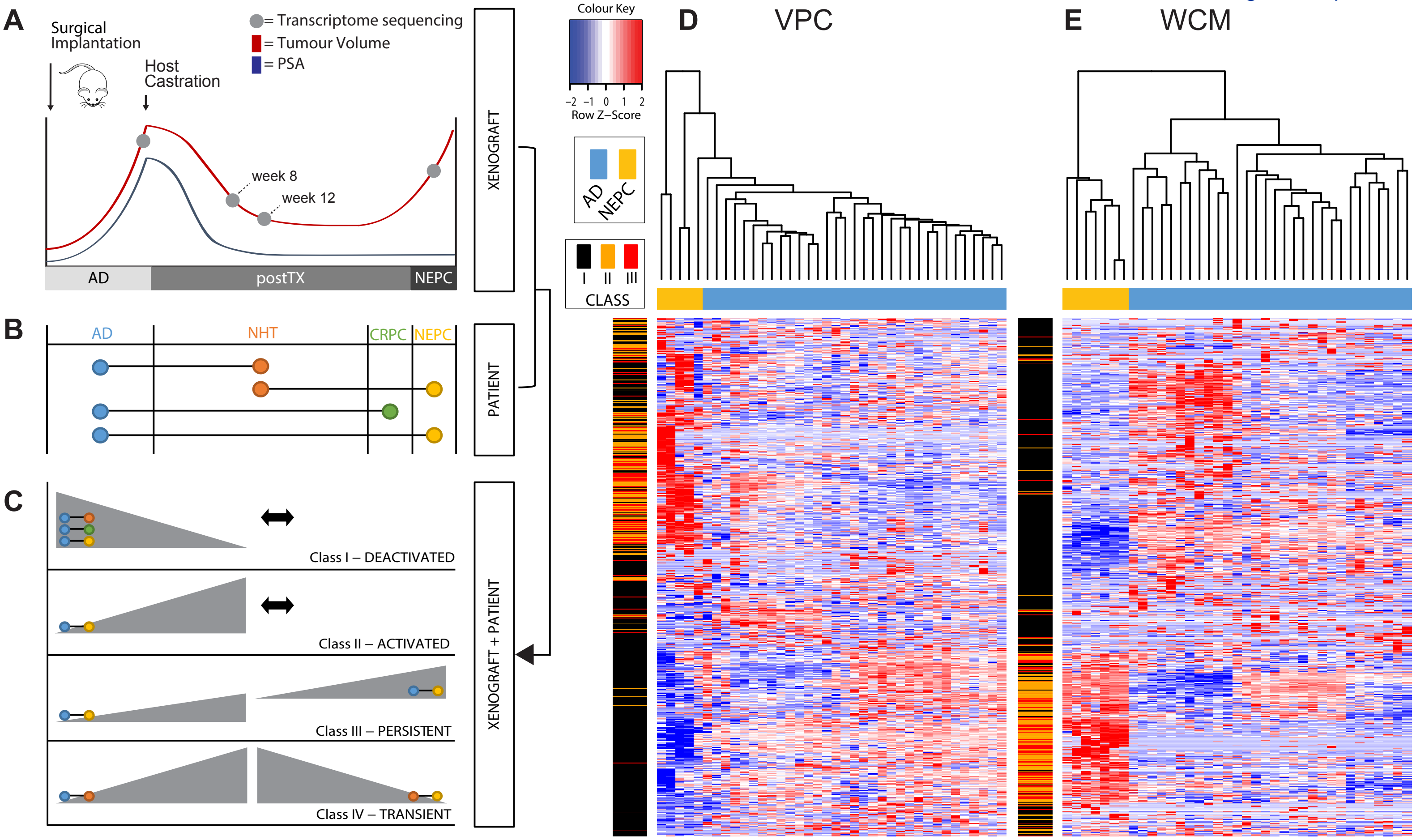

Figure 4

Click here to download Figure F4.pdf

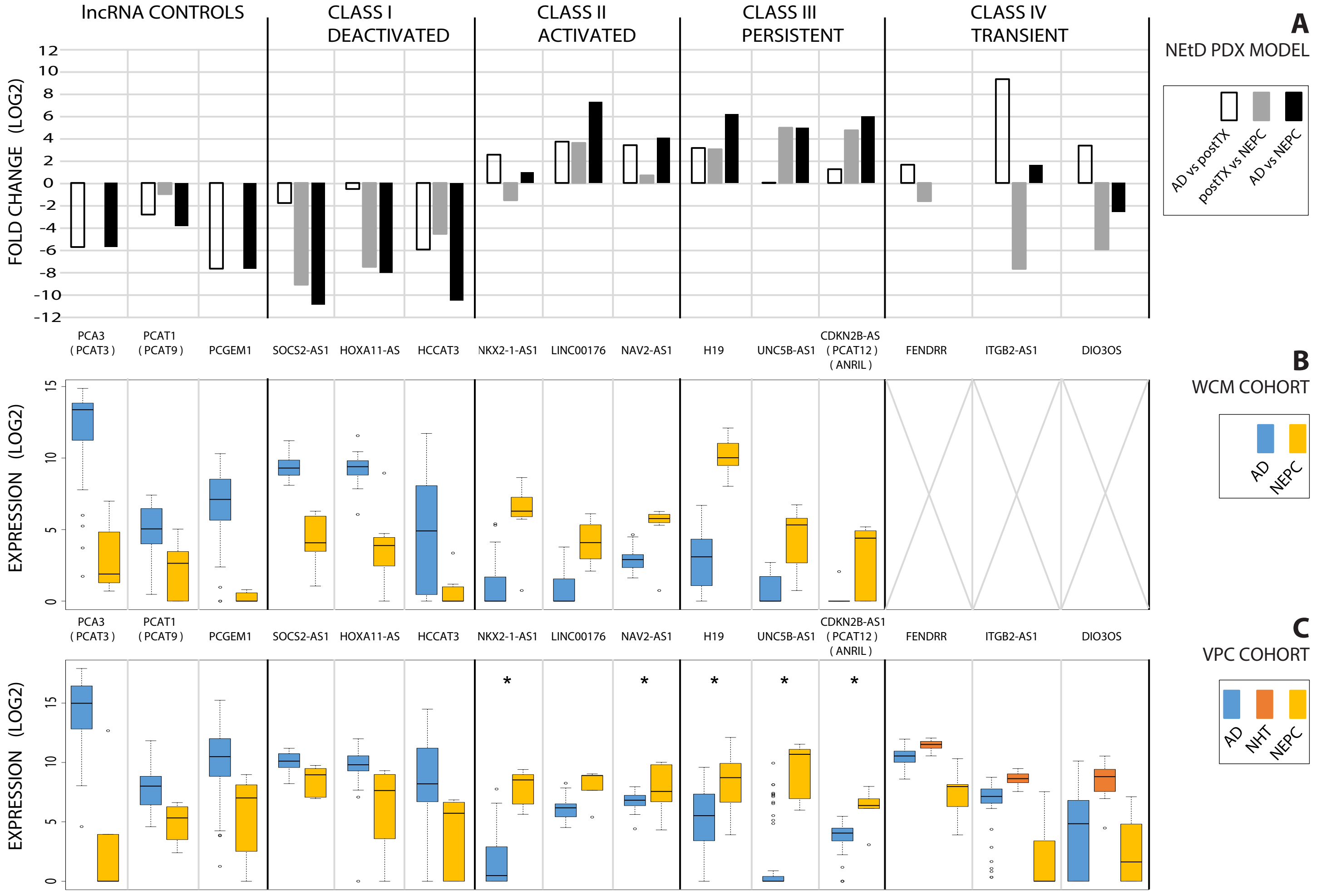

Figure 5

A

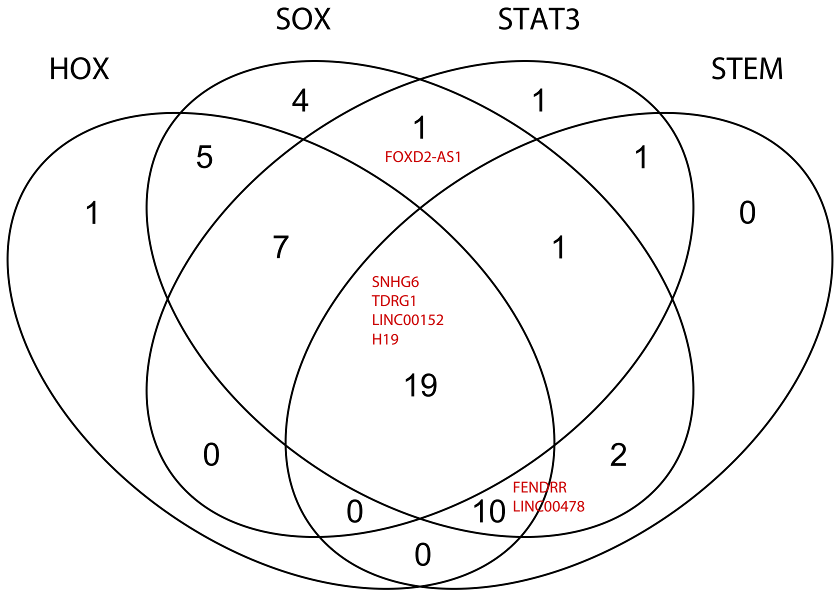
HOX
SOX
STAT3
STEM
4
1
1
5
FOXD2-AS1
1
1
0
7
1
SNHG6
TDRG1
LINC00152
H19
19
0
2
FENDRR
LINC00478
0
10
0

B

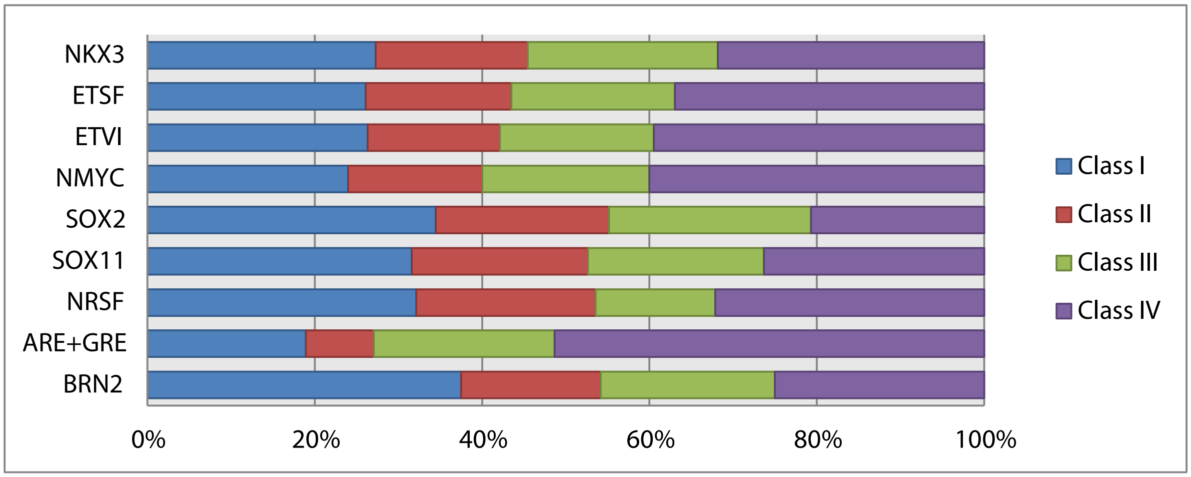
NKX3
ETSF
ETVI
NMYC
SOX2
SOX11
NRSF
ARE+GRE
BRN2
0%
20%
40%
60%
80%
100%
Class I
Class II
Class III
Class IV

Figure 6

Click here to download Figure F6.pdf

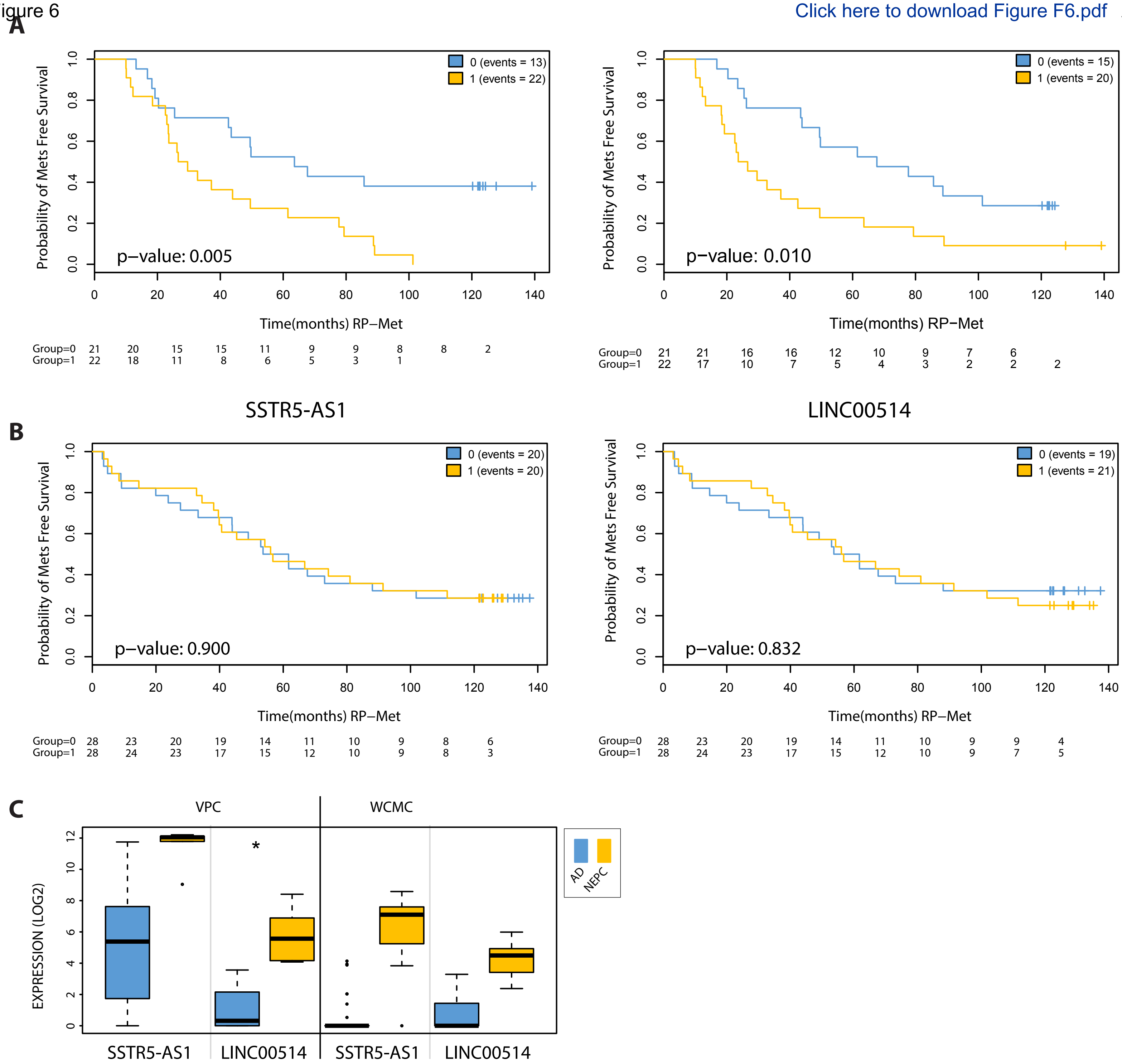

Supplementary Figure 1

Click here to access/download
Supplementary Material
SF1.pdf

Supplementary Figure 2

Click here to access/download
Supplementary Material
SF2.pdf

Supplementary Figure 3

Click here to access/download
Supplementary Material
SF3.pdf

Supplementary Figure 4

Click here to access/download
Supplementary Material
SF4.pdf

Supplementary Figure 5

Click here to access/download
Supplementary Material
SF5.pdf

Supplementary Figure 6

Click here to access/download
Supplementary Material
SF6.pdf

Supplementary Figure 7

Click here to access/download
Supplementary Material
SF7.pdf

Supplementary Figure 8

Click here to access/download
Supplementary Material
SF8.pdf

Supplementary Figure 9

Click here to access/download
Supplementary Material
SF9.pdf

Supplementary Figure 10

Click here to access/download
Supplementary Material
SF10.pdf

Supplementary Figure 11

Click here to access/download
Supplementary Material
SF11.pdf

Supplementary Figure 12

Click here to access/download

Supplementary Material

SF12.pdf

Supplementary Figure 13

Click here to access/download
Supplementary Material
SF13.pdf

Supplementary Figure 14

Click here to access/download
Supplementary Material
SF14.pdf

Supplementary Tables 1 - 28

Click here to access/download
Supplementary Material
ST1-28.xlsx
