# Supplementary material for: The long noncoding RNA landscape of neuroendocrine prostate cancer and its clinical implications
Source: Gigascience. 2018 May 10;7(6):giy050. doi: 10.1093/gigascience/giy050 (PMC6007253; doi:10.1093/gigascience/giy050)
Supplement: Supplement Files [file giy050_supplement_files.zip › SF1.pdf]

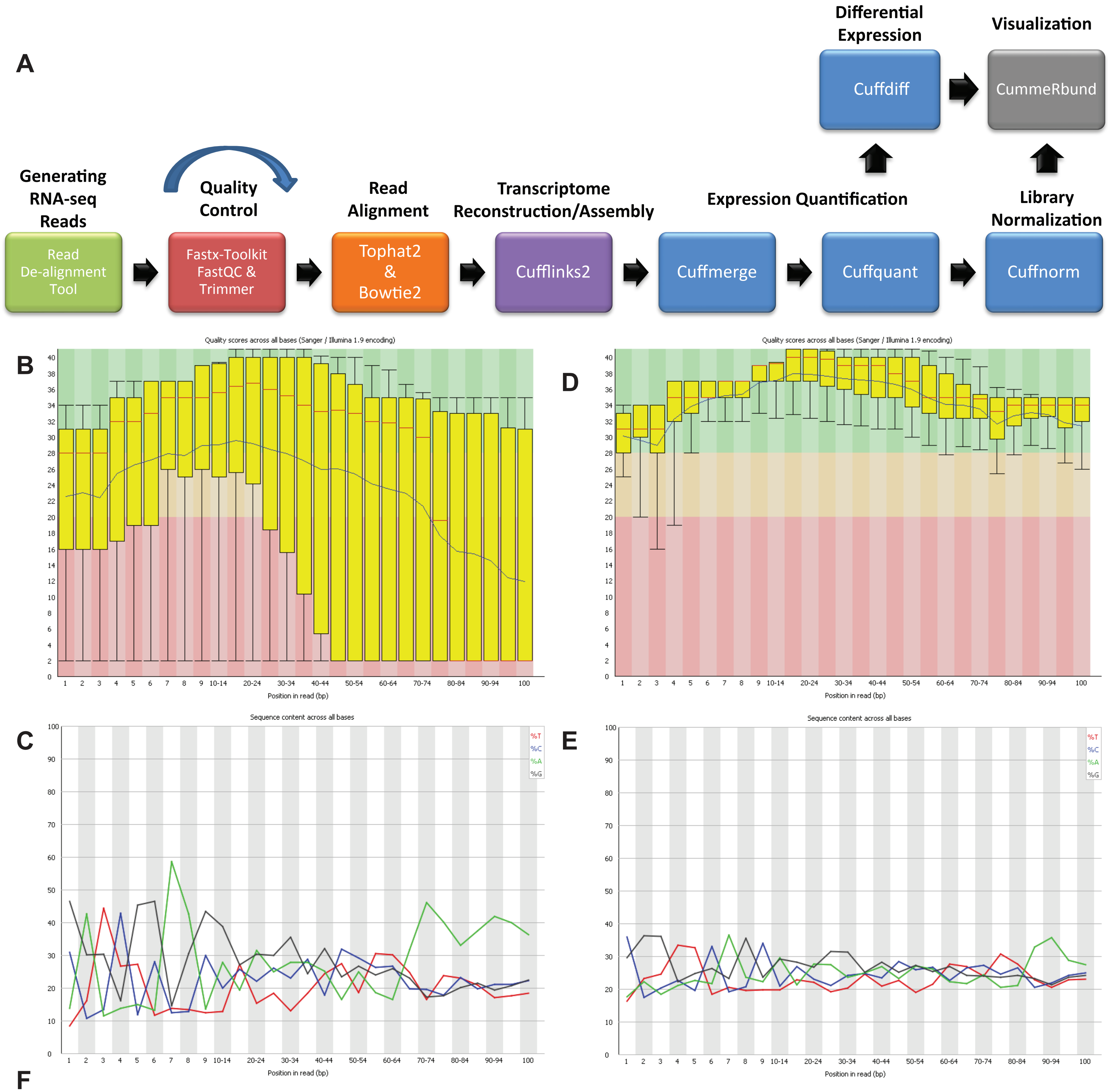

F

Sequence	Count	Percentage	Possible Source
GATCGGAAGAGCGGTTCAGCAGGAATGCCGAGACCGACAGTGATCTCGTA	60786256	15.311652466318707	Illumina Paired End PCR Primer 2 (97% over 37bp)
CGGTTCAGCAGGAATGCCGAGATCGGAAGAGCGGTTCAGCAGGAATGCCG	22125941	5.573376966699054	Illumina Paired End PCR Primer 2 (100% over 31bp)
GAGCGGAAGAGCGGTTCAGCAGGAATGCCGAGACCGACAGTGATCTCGTA	1619516	0.40794527887426735	Illumina Paired End PCR Primer 2 (97% over 37bp)
GATCGGAAGAGCGGTTCAGCAGGAATGCCGAGACCGTGACCAATCTCGTA	1272543	0.3205450943457779	Illumina Paired End PCR Primer 2 (97% over 36bp)
GATCGGAAGAGCGGTTCAGCAGGAATGCCGAGACCGGCCAATATCTCGTA	1057985	0.26649936516205563	Illumina Paired End PCR Primer 2 (97% over 36bp)
CGGAAGAGCGGTTCAGCAGGAATGCCGAGACCGACAGTGATCTCGTATGC	542971	0.13677077350000852	Illumina Paired End PCR Primer 2 (97% over 34bp)
CGGTTCAGCAGGAATGCCGAGACCGACAGTGATCTCGTATGCCGTCTTCT	414121	0.10431431787811325	Illumina Paired End PCR Primer 2 (96% over 26bp)
