# Supplementary material for: The long noncoding RNA landscape of neuroendocrine prostate cancer and its clinical implications
Source: Gigascience. 2018 May 10;7(6):giy050. doi: 10.1093/gigascience/giy050 (PMC6007253; doi:10.1093/gigascience/giy050)
Supplement: Supplement Files [file giy050_supplement_files.zip › SF11.pdf]

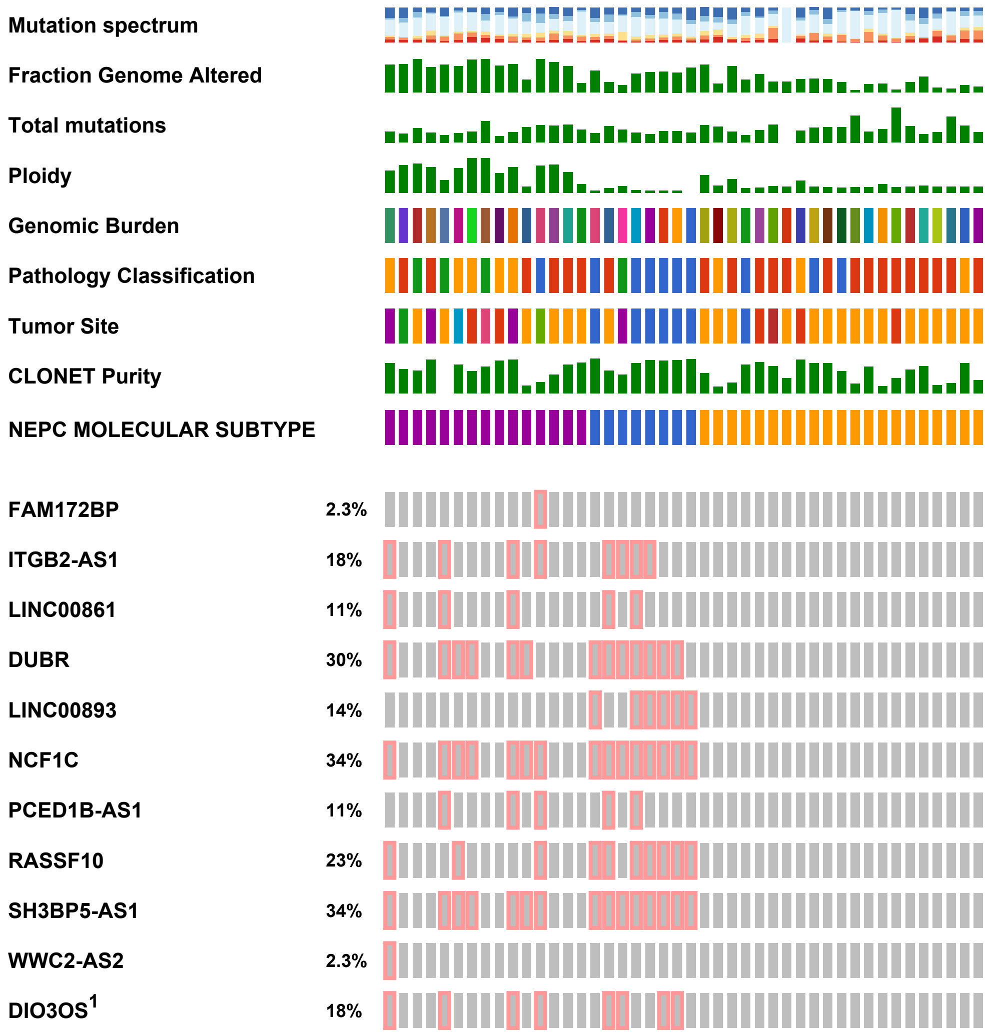


| | TOTALS | PERCENTAGE % |
|---|---|---|
| NEtD lncRNA - Class IV | 45 | 100 |
| extNEPC Detectable | 36 | 80 |
| extNEPC Detectable AND Altered[2] | 11 | 31 |
| extNEPC Detectable AND Unaltered | 25 | 69 |

[1]Overlapped NEtD lncRNA with NEPC lncRNA

[2]Displayed in plot above
