# Supplementary material for: The long noncoding RNA landscape of neuroendocrine prostate cancer and its clinical implications
Source: Gigascience. 2018 May 10;7(6):giy050. doi: 10.1093/gigascience/giy050 (PMC6007253; doi:10.1093/gigascience/giy050)
Supplement: Supplement Files [file giy050_supplement_files.zip › SF12.pdf]

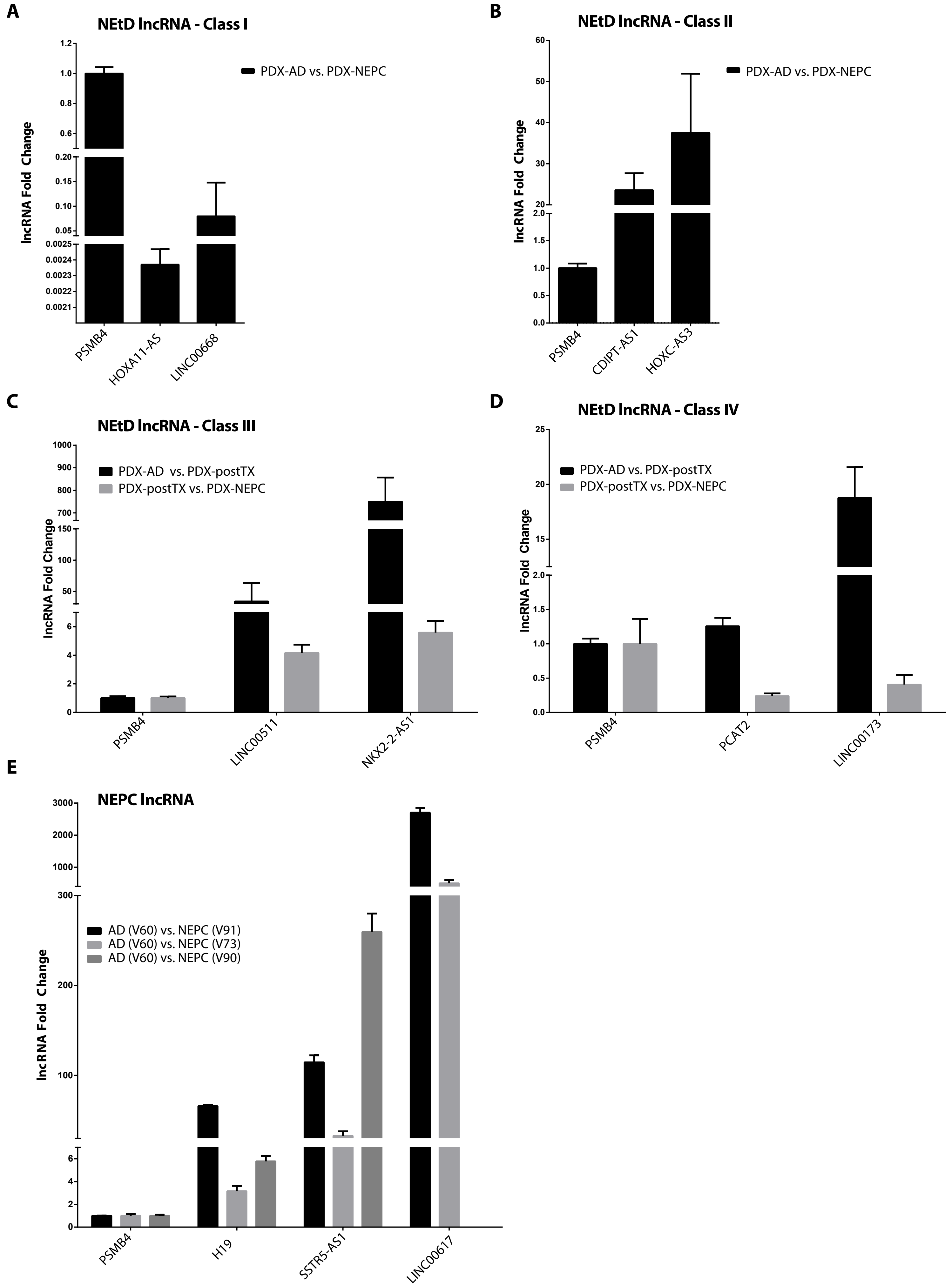

A
NEtD lncRNA - Class I
PDX-AD vs. PDX-NEPC
lncRNA Fold Change
1.2
1.0
0.8
0.6
0.20
0.15
0.10
0.05
0.0025
0.0024
0.0023
0.0022
0.0021
PSMB4
HOXA11-AS
LINC00668
B
NEtD lncRNA - Class II
PDX-AD vs. PDX-NEPC
lncRNA Fold Change
60
50
40
30
20
2.0
1.5
1.0
0.5
0.0
PSMB4
CDIPT-AS1
HOXC-AS3
C
NEtD lncRNA - Class III
PDX-AD vs. PDX-postTX
PDX-postTX vs. PDX-NEPC
lncRNA Fold Change
1000
900
800
700
150
100
50
6
4
2
0
PSMB4
LINC00511
NKX2-2-AS1
D
NEtD lncRNA - Class IV
PDX-AD vs. PDX-postTX
PDX-postTX vs. PDX-NEPC
lncRNA Fold Change
25
20
15
2.0
1.5
1.0
0.5
0.0
PSMB4
PCAT2
LINC00173
E
NEPC lncRNA
AD (V60) vs. NEPC (V91)
AD (V60) vs. NEPC (V73)
AD (V60) vs. NEPC (V90)
lncRNA Fold Change
3000
2000
1000
300
200
100
6
4
2
0
PSMB4
H19
SSTR5-AS1
LINC00617
