# Supplementary material for: The long noncoding RNA landscape of neuroendocrine prostate cancer and its clinical implications
Source: Gigascience. 2018 May 10;7(6):giy050. doi: 10.1093/gigascience/giy050 (PMC6007253; doi:10.1093/gigascience/giy050)
Supplement: Supplement Files [file giy050_supplement_files.zip › SF6.pdf]

Genetic Alteration
Amplification; Deep Deletion; mRNA Upregulation; mRNA Downregulation; Truncating Mutation (putative driver); Missense Mutation (putative driver); Missense Mutation (putative passenger)

Mutation spectrum
C>A; C>G; C>T; T>A; T>C; T>G

Fraction Genome Altered
0 1

Total mutations
6 752

Ploidy
1.6 3.84

Genomic Burden
0.4463; 0.4237; 0.445; 0.3153; 0.4081; 0.3699; 0.4089; 0.0312; 0.2339; 0.2853; 0.387; 0.1657; 0.2058; 0.7753; 0.8151; 0.0933; 0.7718; 0.7924; 0.3649; 0.343; 0.6356; 0.9437; 0.7382; 0.1871; 0.9471; 0.0466; 0.0012; 0.0026; 0.2964; 0.1899; 0.2676; 0.1946; 0.0914; 0.061; 0.1614; 0.1028; 0.2368; 0.8791; 0.2576; 0.916; 0.0518; 0.8337; 0.7374; 0.5794

Pathology Classification
D; B; C; E

Tumor Site
Pelvic mass; Lymph node; Prostate; Bone; Liver; Soft tissue; Bladder; Lung; Retroperitoneal mass

CLONET Purity
0.42 1

NEPC MOLECULAR SUBTYPE
[Assigned by visual inspection]
Group 1; Group 2; Group 3
