# Supplementary material for: The long noncoding RNA landscape of neuroendocrine prostate cancer and its clinical implications
Source: Gigascience. 2018 May 10;7(6):giy050. doi: 10.1093/gigascience/giy050 (PMC6007253; doi:10.1093/gigascience/giy050)
Supplement: Supplement Files [file giy050_supplement_files.zip › SF8.pdf]

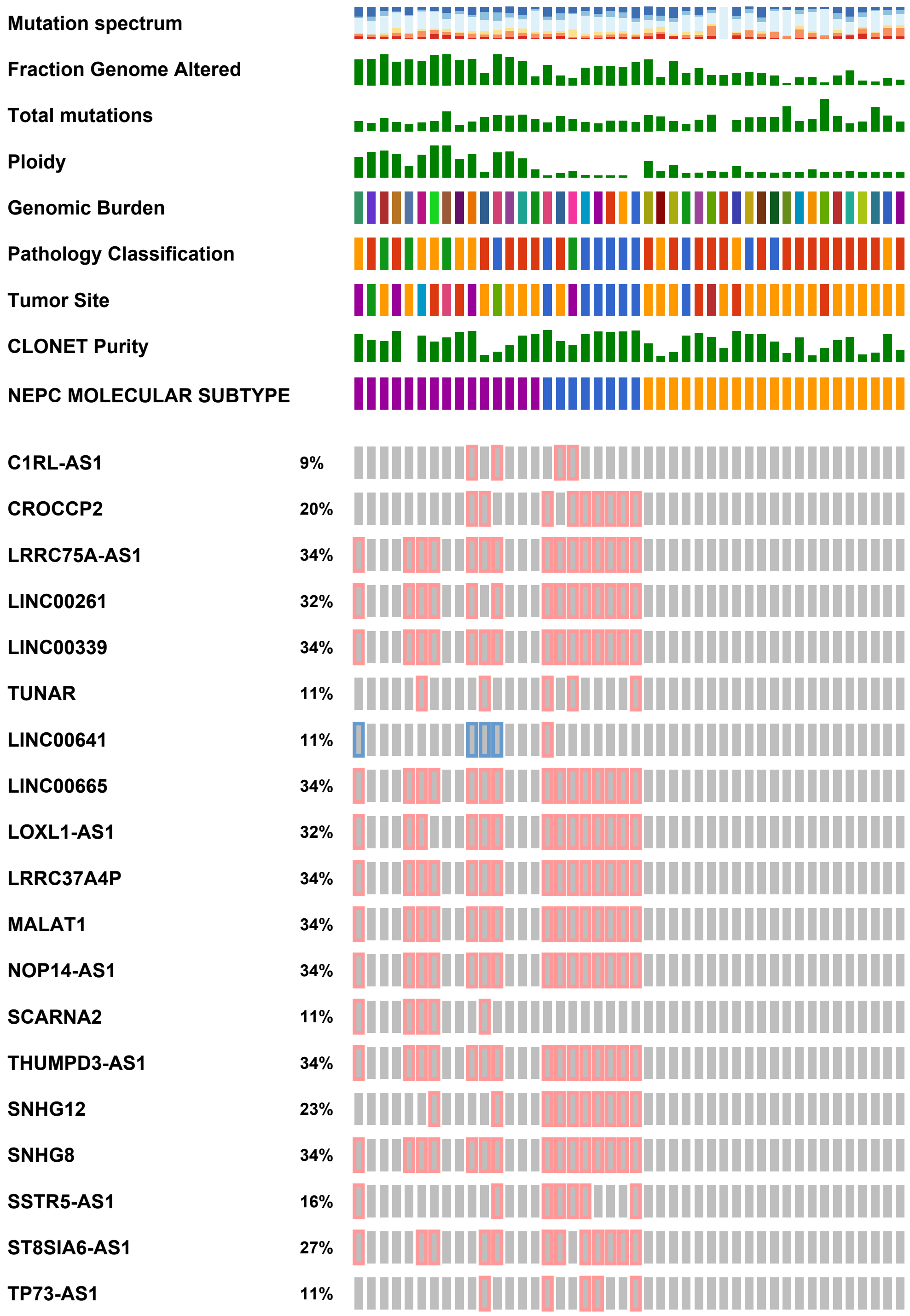

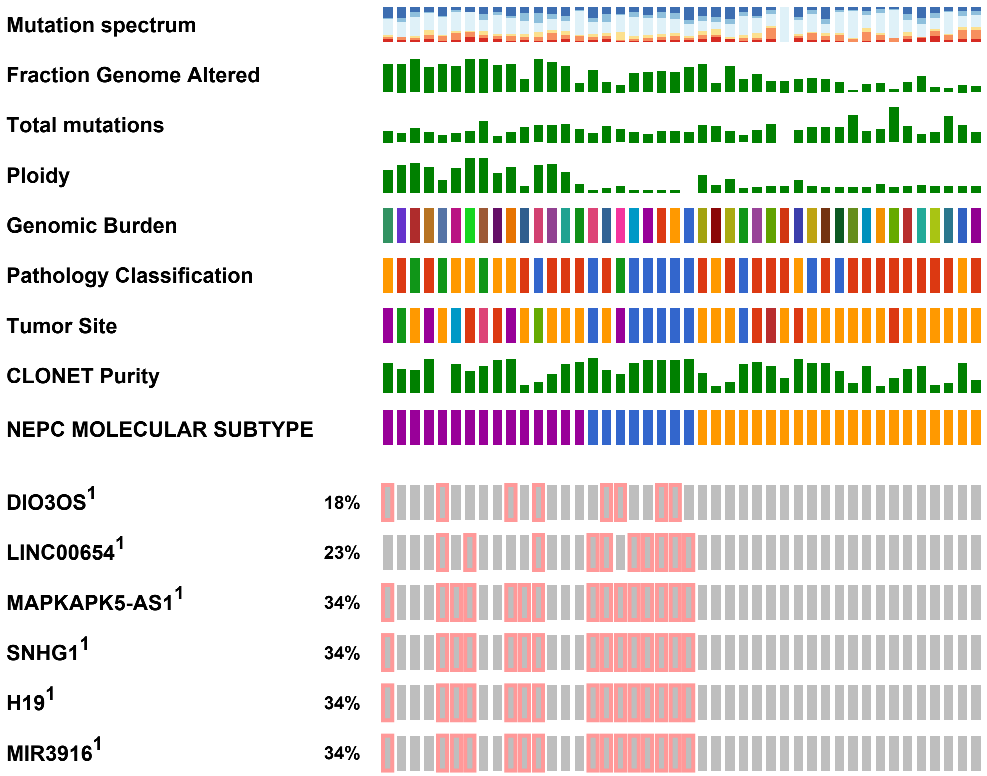

	TOTALS	PERCENTAGE %
NEPC lncRNA	122 (74 UP / 48 DOWN)	100
extNEPC Detectable (UP)	58	78
extNEPC Detectable AND Altered[2]	25	43
extNEPC Detectable AND Unaltered	33	57

[1]Overlapped NEPC lncRNA with NEtD lncRNA

[2]Displayed in plot above
