# Supplementary material for: The long noncoding RNA landscape of neuroendocrine prostate cancer and its clinical implications
Source: Gigascience. 2018 May 10;7(6):giy050. doi: 10.1093/gigascience/giy050 (PMC6007253; doi:10.1093/gigascience/giy050)
Supplement: Supplement Files [file giy050_supplement_files.zip › SF9.pdf]

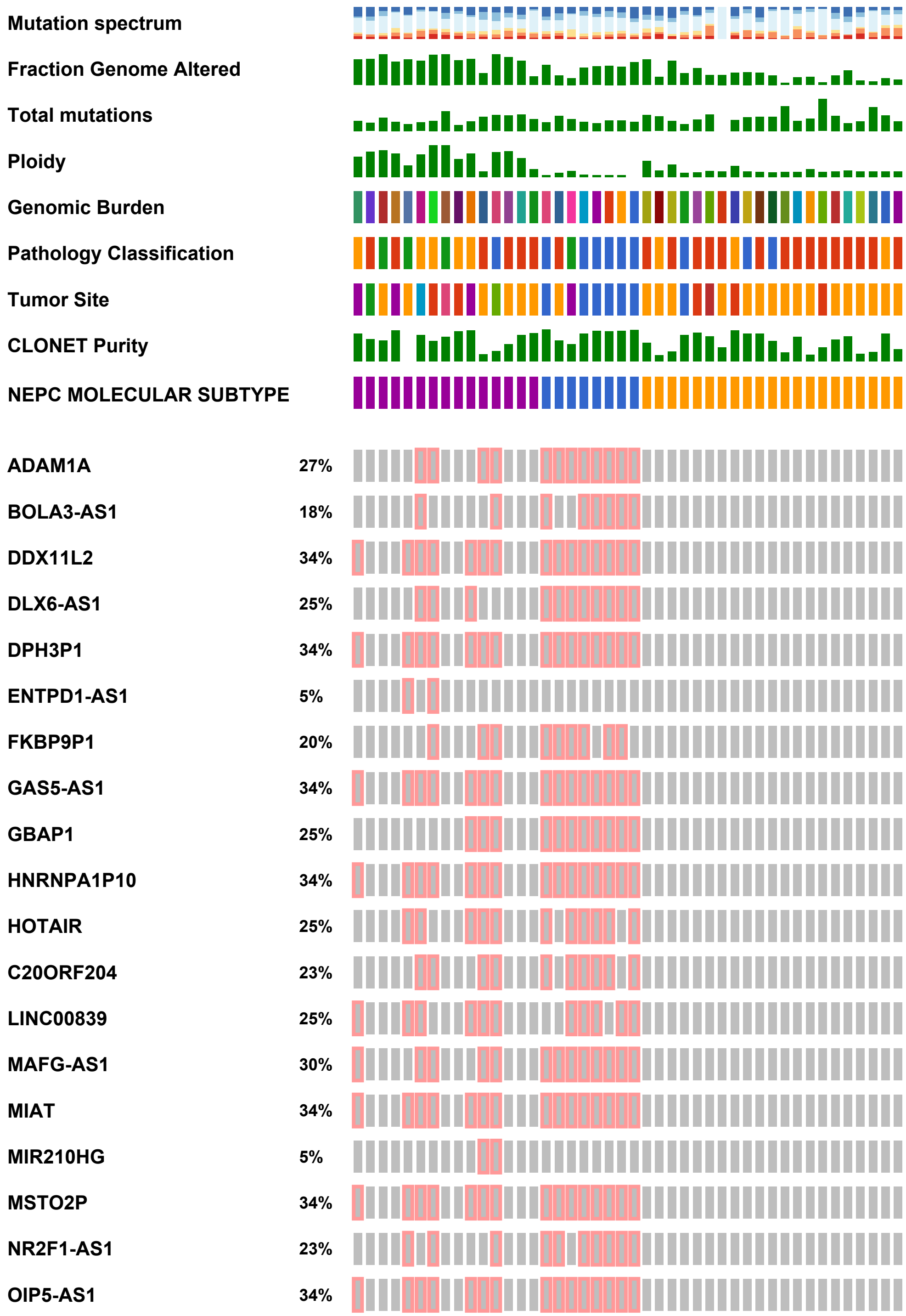

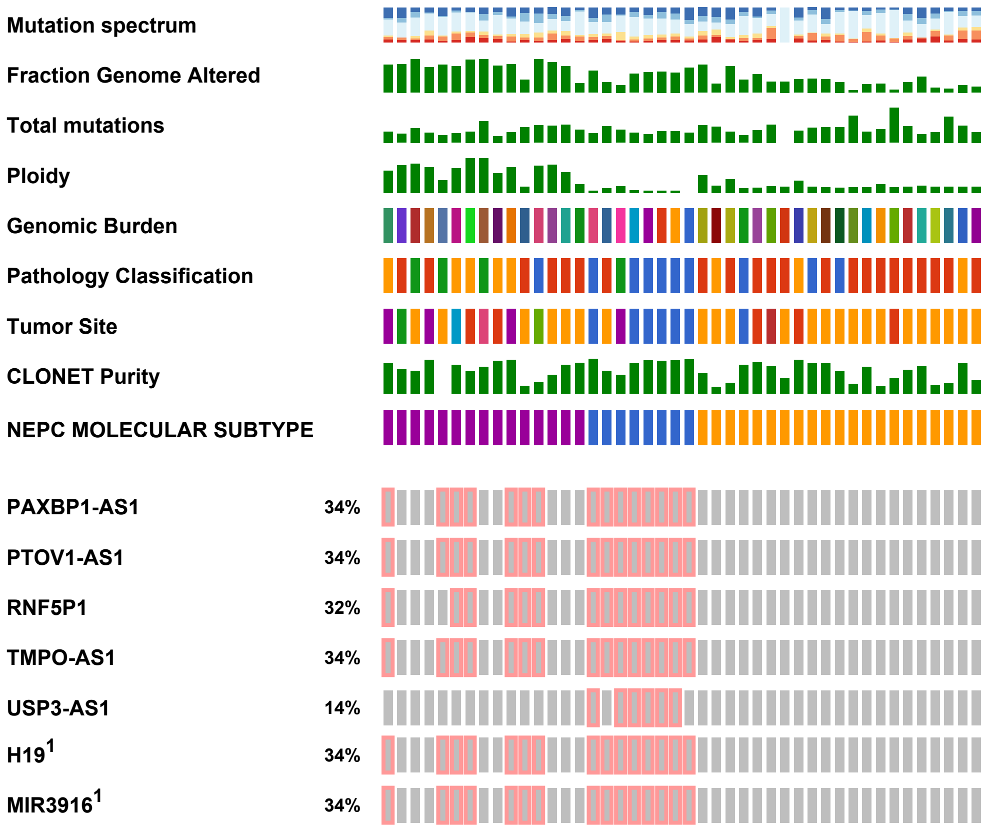

	TOTALS	PERCENTAGE %
NEtD lncRNA - Class II	222	100
extNEPC Detectable	128	58
extNEPC Detectable AND Altered[2]	26	20
extNEPC Detectable AND Unaltered	102	80

[1]Overlapped NEtD lncRNA with NEPC lncRNA

[2]Displayed in plot above
