# Supplementary figures and images for: The long noncoding RNA landscape of neuroendocrine prostate cancer and its clinical implications
Source: Gigascience. 2018 May 10;7(6):giy050. doi: 10.1093/gigascience/giy050 (PMC6007253; doi:10.1093/gigascience/giy050)

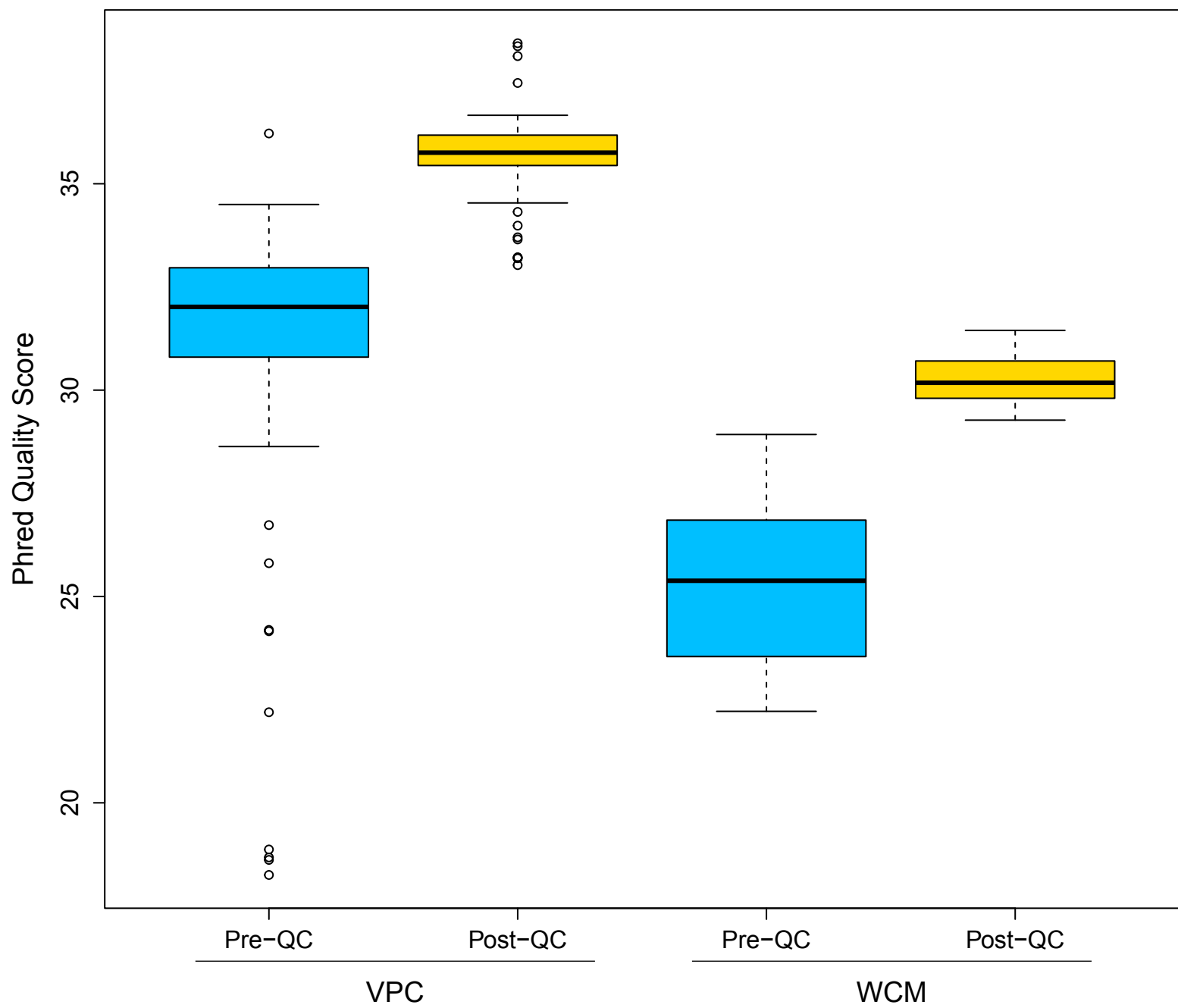

Supplement: Supplement Files [file giy050_supplement_files.zip › SF2.pdf]

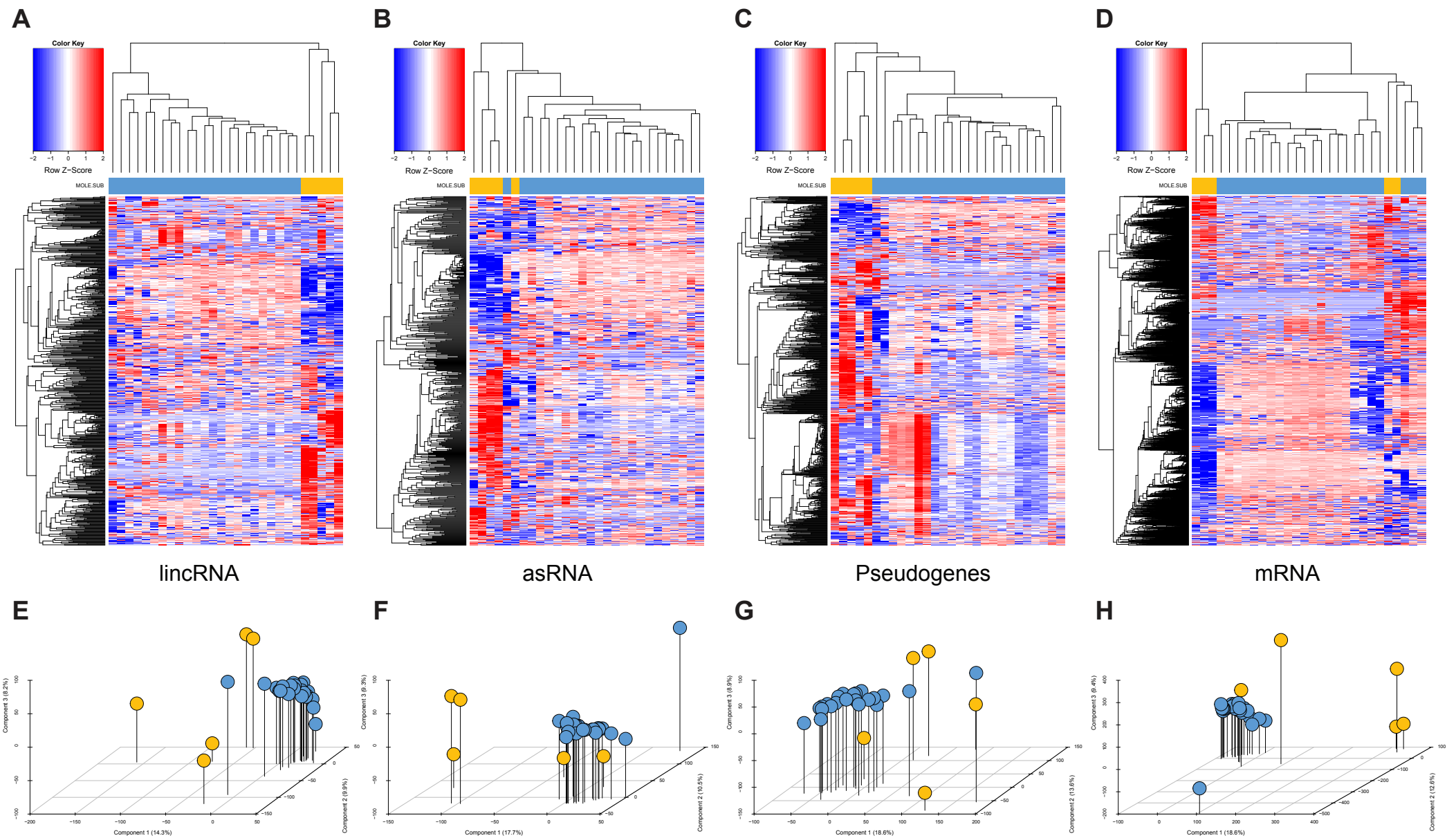

Supplement: Supplement Files [file giy050_supplement_files.zip › SF3.pdf]

**A**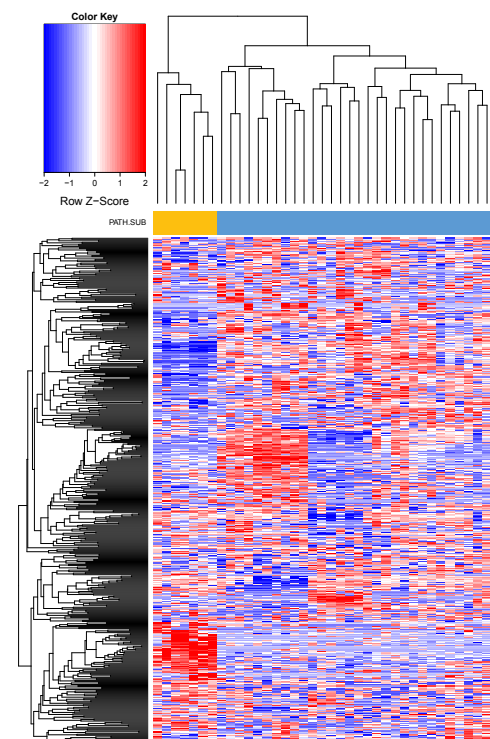

lincRNA

**B**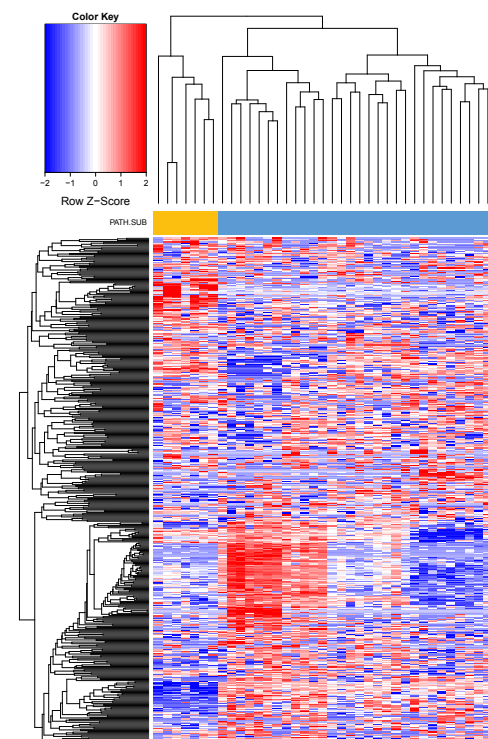

asRNA

**C**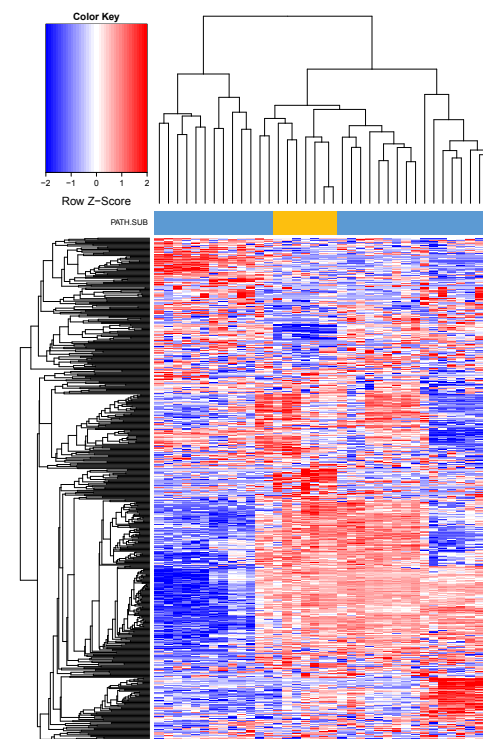

Pseudogenes

**D**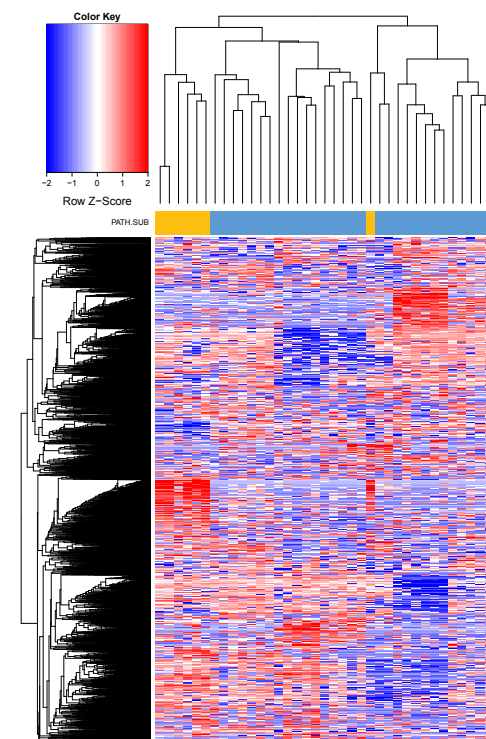

mRNA

**E**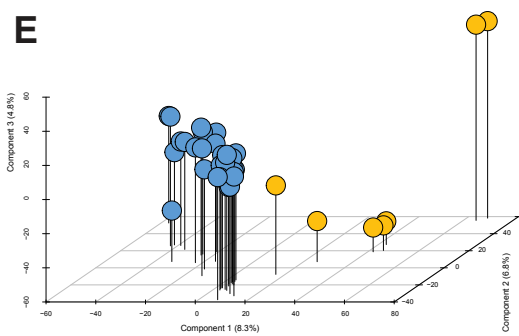**F**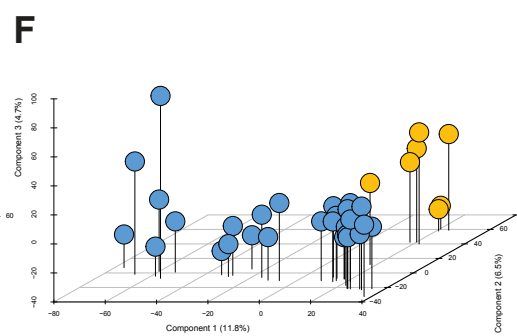**G**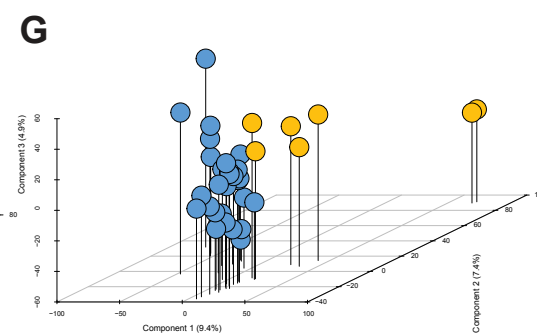**H**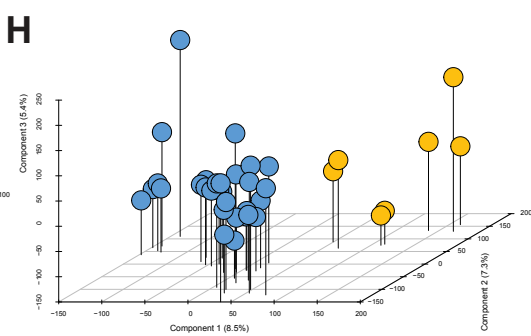

Supplement: Supplement Files [file giy050_supplement_files.zip › SF4.pdf]

**A**

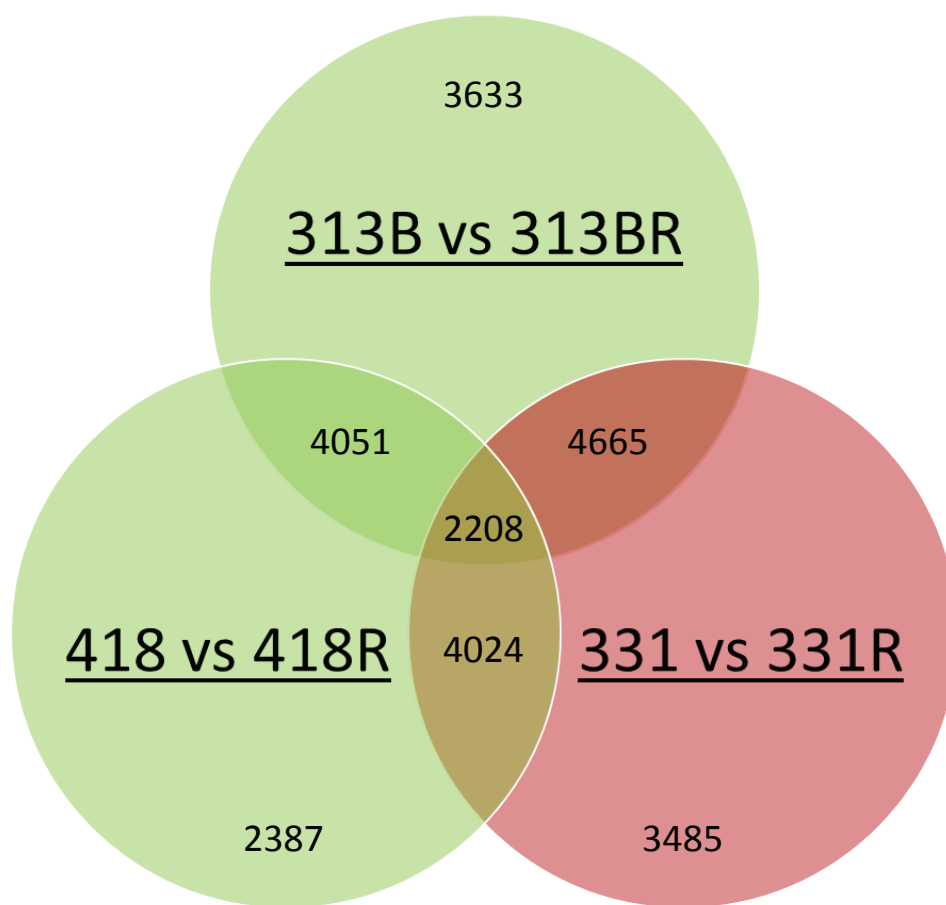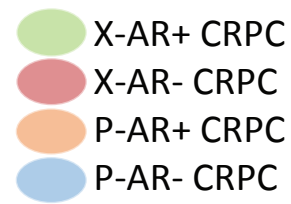

**B**

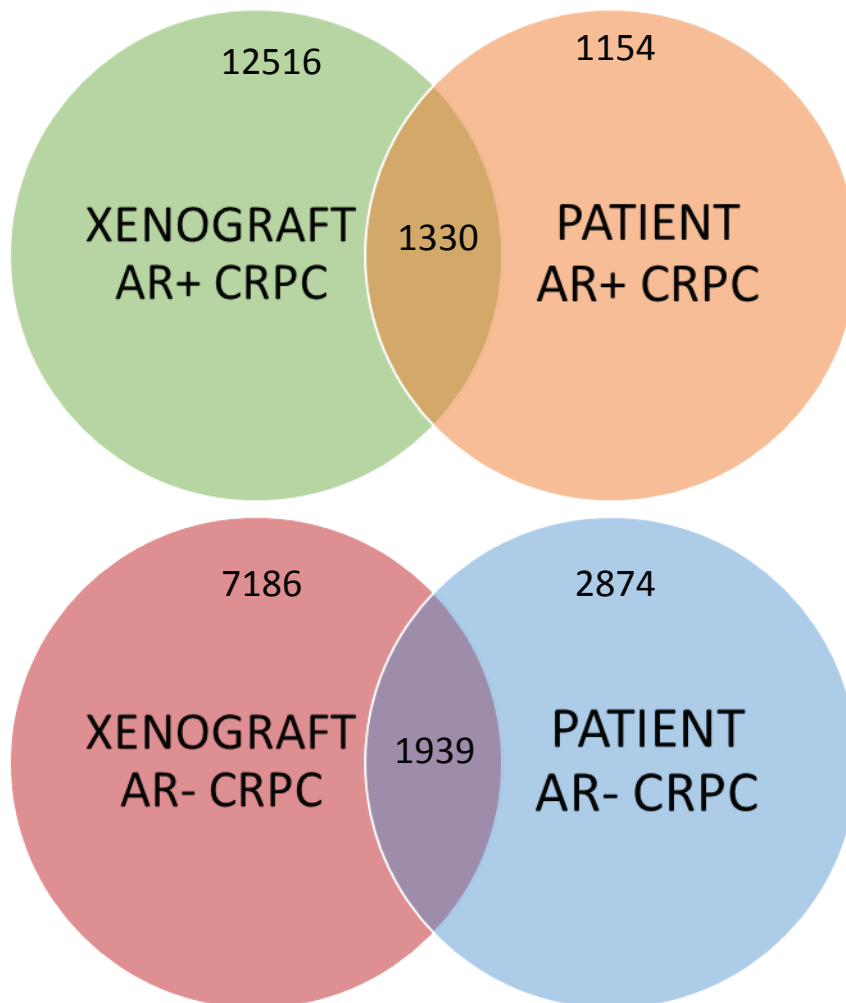

Supplement: Supplement Files [file giy050_supplement_files.zip › SF5.pdf]

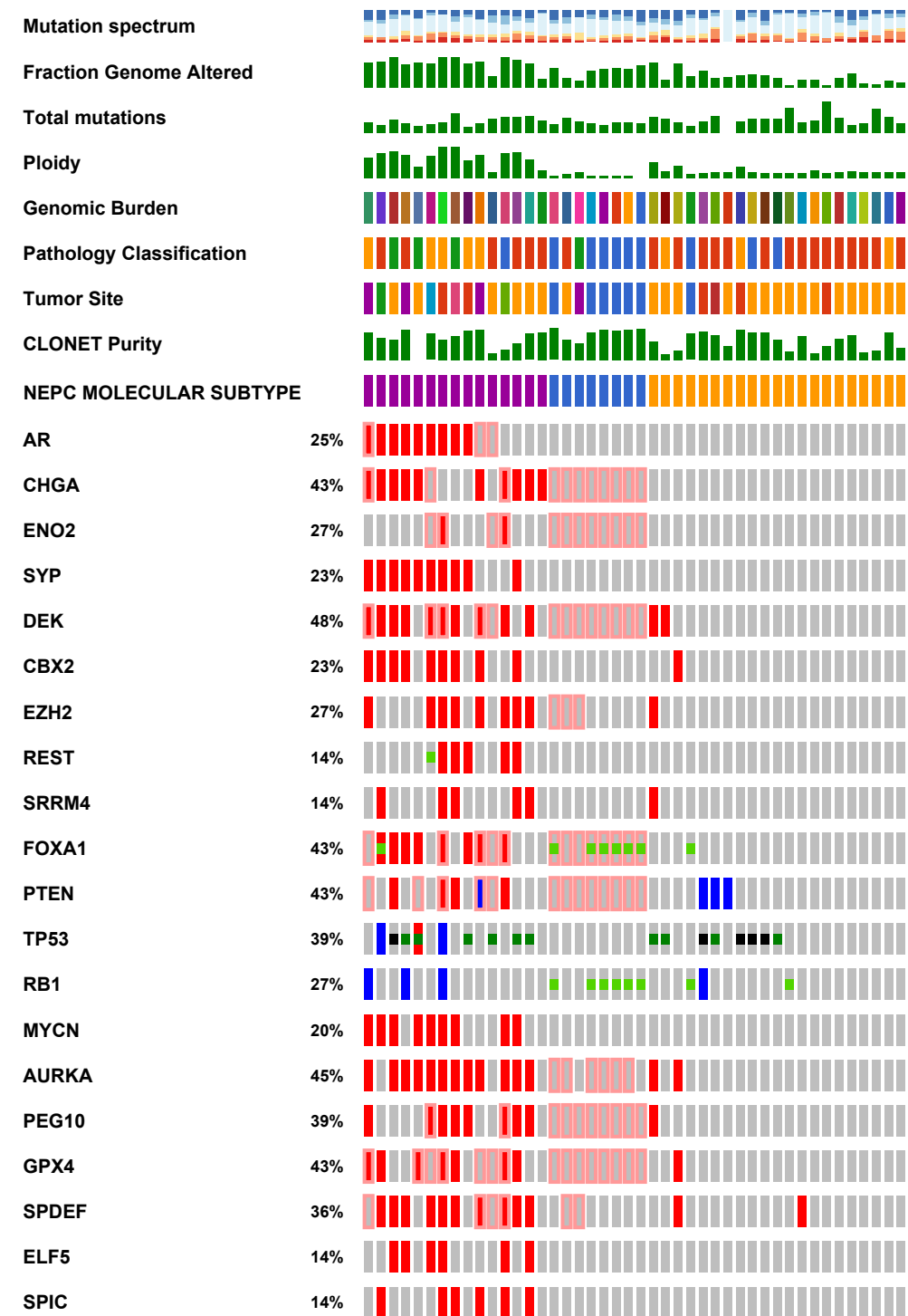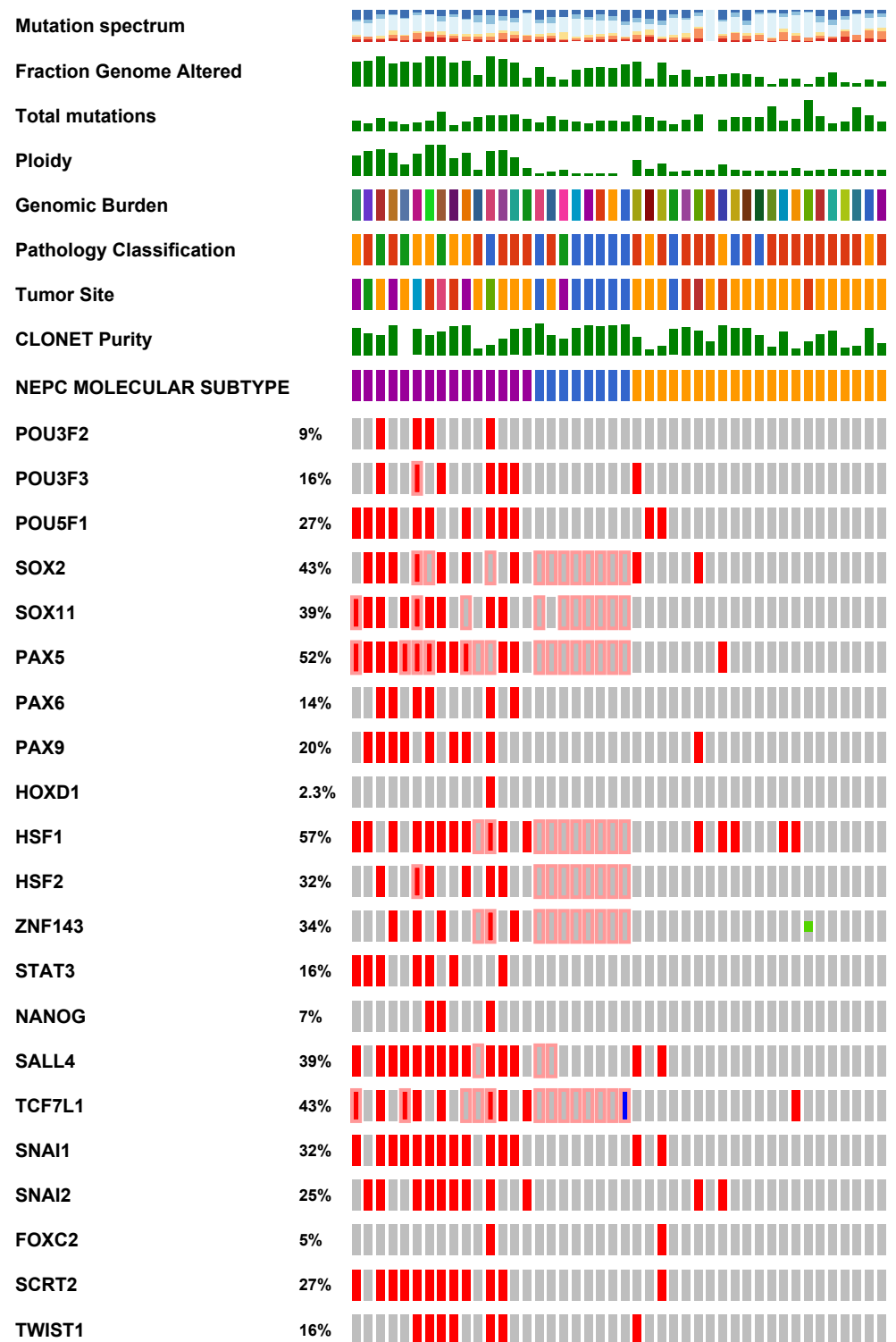

Supplement: Supplement Files [file giy050_supplement_files.zip › SF7.pdf]
