# Supplementary material for: The long noncoding RNA landscape of neuroendocrine prostate cancer and its clinical implications
Source: Gigascience. 2018 May 10;7(6):giy050. doi: 10.1093/gigascience/giy050 (PMC6007253; doi:10.1093/gigascience/giy050)
Supplement: Supplement Files [file SF14.pdf › SF14.pdf]

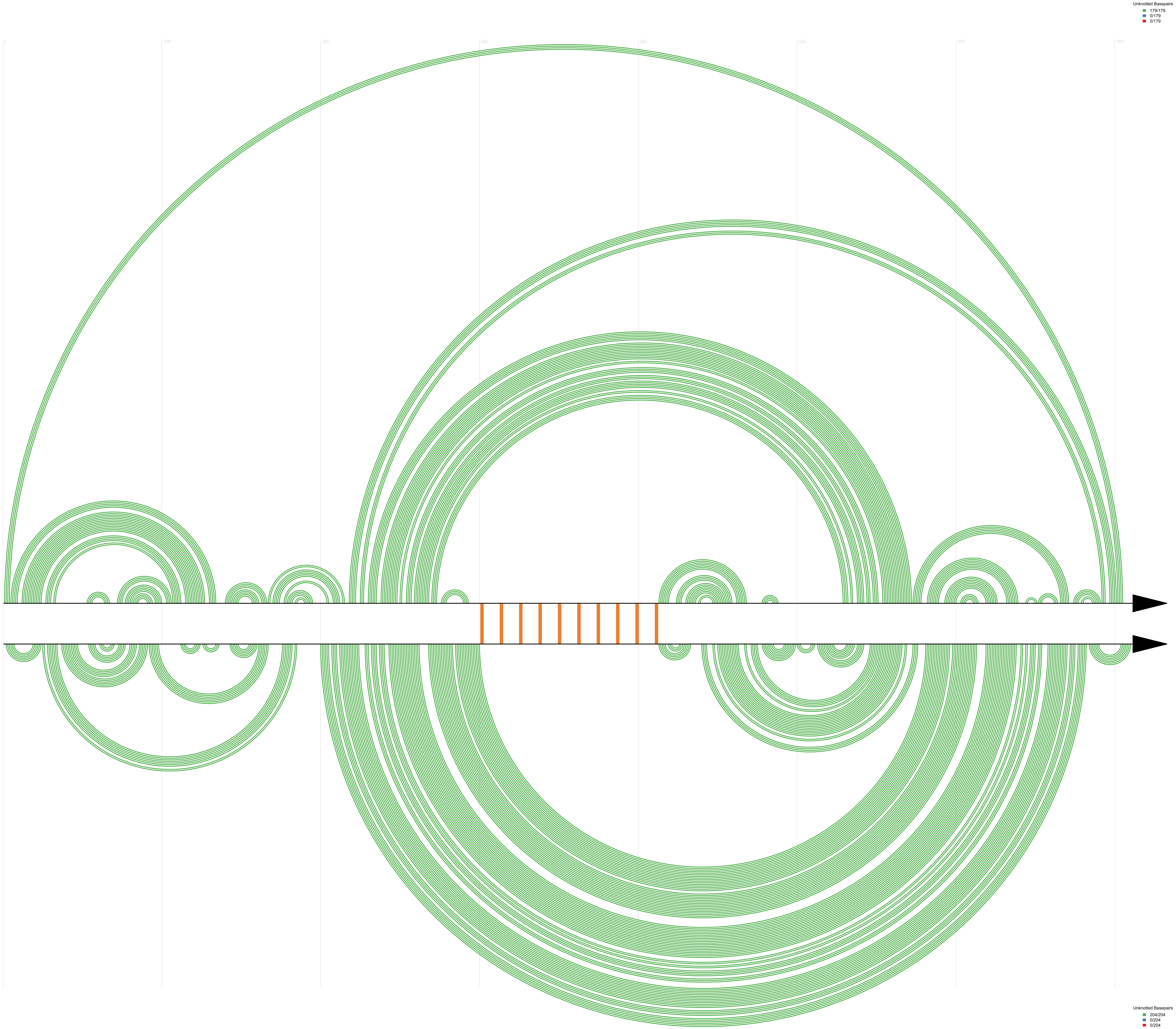

Unknotted Basepairs
179/179
0/179
0/179
0
100
200
300
400
500
600
700
Unknotted Basepairs
204/204
0/204
0/204

www.e-rna.org
