# Supplementary material for: The long noncoding RNA landscape of neuroendocrine prostate cancer and its clinical implications
Source: Gigascience. 2018 May 10;7(6):giy050. doi: 10.1093/gigascience/giy050 (PMC6007253; doi:10.1093/gigascience/giy050)
Supplement: Response_to_Reviewer_Comments_Original_Submission.pdf [file giy050_response_to_reviewer_comments_original_submission.pdf]

Author's Response To Reviewer Comments

Close

Response to reviewers: our responses are indicated by BOLD text below each of the reviewers' comments. We would also like to note that in response to the reviewer's comments and to strengthen our manuscript as a whole, we have added a large amount of new data (1 Main Figure, 7 Supplementary Figures and 20 Supplementary Tables).

Reviewer #1:

Neuroendocrine prostate cancer (NEPC) is an aggressive disease. Since the recent FDA approval of potent AR targeted therapies for the treatment of metastatic prostate cancer, the incidence of AR-negative NEPC will escalate as a mechanism of resistance. Genomic and transcriptomic profiling have been done to characterize NEPC tumors, yet the landscape of lnRNA has never been characterized. In this article, Ramnarine et al. leverage their well-characterized xenograft model, as well as patient samples, to identify lnRNA deregulated in NEPC. They further define five expression patterns associated with the disease progression from adenocarcinoma to CRPC and NEPC. They further associated some of the lnRNA expression with treatment-associated metastasis.

The manuscript focuses on lnRNA, an aspect unexplored in NEPC. The data presented are interesting but some of the data needs to be strengthened.

We agree with each of Review #1's points and acknowledge that it strengthens our work. We have carried out the requested revisions, explained each one in detail below, and referenced manuscript amended lines. We have also referenced new figures/tables necessary for these revisions. We thank the reviewer for the time taken in assessing our research and for their constructive feedback.

1. The authors use a large amount of RNAseq data and implemented a pipeline to analyze the expression of long non-coding RNA. All their evidences described in this paper are based upon in sillico analyses. The authors must validate their finding. Q-RT-PCR experiment should be carried out to validate top candidates in each class defined by the authors in the xenografts model. In addition, as proof of principle, validation in a few clinical samples by RT-Q-PCR or by RNAish in fixed material will further strengthen the manuscript.

As requested from the Reviewer, we performed qRT-PCR and validated 8 of our NEtD lncRNA candidates, choosing two from within each of the four NEtD transcriptome defining classes. Each of these candidates was validated in the appropriate time points of our NEtD PDX xenograft model (corresponding to how they were discovered). We also validated an additional 3 lncRNAs from our NEPC lncRNA patient signature. Each of these candidates was validated in NEPC clinical samples relative to an AD clinical sample. All of these lncRNAs were among our top candidates (n=100 – Figure 1B). Please see supplementary Table 25 and Supplementary Figure 12 for corresponding qRT-PCR primer sequences and expression fold changes, respectively. Manuscript and method revisions for these experiments are on lines 478-480 and 696-722, respectively.

2. The authors define five patterns of expression. They named class I "AR regulated" and class II "Neuronal". NEPC is characterized by the loss of AR and AR signaling, and it's expected that AR target genes and lnRNA will go down. However, this nomenclature is misleading. The authors do not prove

that lnRNAs falling into class I are indeed regulated by AR. Different mechanisms or pathways might be involved, in addition to AR signaling. Does the list of lnRNA in class I significantly overlap with lnRNA regulated by AR? The authors should leverage existing RNAseq data sets while looking at AR regulated transcripts. Answering these questions will strongly strengthen the data.
The reviewer has raised a very important point that we agree can be misleading to the reader regarding our results for NEtD lncRNA Class I (AR-regulated) and II (neuronal). To address this we have re-named these classes to "deactivated" (Class I) and "activated" (Class II). This nomenclature aligns with the rationale of Class III, IV and V (describing their expression patterns) and not their biological relationship to NEtD. We should note that despite these changes, we still believe transcripts belonging to Class I (deactivated) contain AR-regulated transcripts and Class II (activated) contain neuronal lncRNAs, just not in their entirety. The reviewer has asked if the lncRNAs in this class significantly overlap with lncRNA known to be regulated by the AR. However, due to the lack of existing genome-wide studies on lncRNAs regulated by the AR and expressed in neuronal tissue, identifying significant overlap of our lncRNAs in these classes would not be feasible. That said, we have added to our manuscript the handful of previously identified lncRNAs (AR regulated) that do overlap in Class I (see manuscript line 390-400). We would like to note, with respect to mRNA genes (protein coding), there are a number of studies that identify genome-wide AR-regulated mRNA transcripts (via ChIP-sequencing). However, after investigating these more deeply, they do not contain non-coding regions in their profiling and analysis.

More importantly, the reviewer mentions that different mechanisms or pathways might be involved in Class I that relate to the loss of the AR and AR signaling, aside from direct AR-regulated lncRNAs. Conversely, the same can be said about our Class II (neuronal) lncRNAs and other non-neuronal mechanisms being activated. We couldn't agree more. To investigate this and identify functional roles for each of our NEtD lncRNA classes, we have added a significant component to our manuscript performing transcription factor (TF) motif sequence analysis (see new result sections titled "NEtD lncRNAs are enriched with distinct transcription factor binding motifs" lines 286-375 and "NEtD lncRNAs contain NEPC-related Transcription Factor Binding Sites" lines 377-438). In general, this analysis identifies putative (with experimental based evidence in other systems) transcription factor binding sites (TFBS) for our lncRNAs. These TFs could be regulating our NEtD lncRNA or the inverse regulation could be occurring. Regardless, the analysis identifies mechanistic relationships to our NEtD lncRNA Classes. We utilize the best in field TFBS database Matbase, in combination with algorithms MatInspector and Overrepresented TFBS by Genomatix (see methods section lines 770-801). We carried out two approaches using Genomatix software: (1) TFBS enrichment analysis (Supplementary Table 5-19) to identify enriched TFBS within each NEtD class and (2) TFBS identification for well-known TFs or known TFs in NEPC (Supplementary Tables 20-24). This was also used to test our hypotheses about the presence/absence of specific TF families within each NEtD classes (i.e. Androgen response element (ARE) sites within NEtD class I). This additional research provides evidence for the roles of our lncRNA in NEtD and addresses the reviewer's requested revision comment #2 (manuscript lines 385-400), comment #3 (manuscript lines 402-409), and comment #4 (manuscript lines 411-426).

This analysis resulted in 20 new supplementary tables of data, providing a rich public resource of motif analysis for our identified NEtD lncRNA and increased depth to our study.

3. The same logic applies to class II "Neuronal". The authors do not provide evidence that these lnRNAs are part of any Neuronal pathways or signatures. Is the list of lnRNA in class II enriched in signatures of neuronal tissues and/or of other neuroendocrine cancers (neuroblastoma, Small Cell Lung Cancer)?

This is a good point raised by the reviewer that we agree with. It further supports the reasoning for

renaming our first two classes and additional research we have added, described above.

In combination with this, also requested by the reviewer, we have explored our NEtD Class II (formerly named neuronal and now named activated) and Class III (persistent) lncRNAs in additional neuronal tissue/neuroendocrine cancers. Directly related to our studies clinical cohort composition, a recent and timely published study in Nature Medicine1 profiles a large 114 CRPC cohort. It is comprised of histologically characterized CRPC of adenocarcinoma (CRPC-AD) and neuroendocrine (CRPC-NEPC) prostate cancers. While the studies' authors do not categorize these CRPC samples into AR+ and AR- (which was one of our focuses), this cohort provides the best external match for validation of our up-regulated NEtD lncRNAs (Class II, III, and IV). We also validate our up-regulated lncRNAs from our NEPC lncRNA signature in this cohort. We observe a high frequency of patients (up to 34%) with up-regulation of some NEPC lncRNA or NEtD lncRNA (see Supplementary Figures 6-11). We describe these results in a new section entitled "NEPC and NEtD lncRNAs identify putative molecular NEPC subtypes" lines 440-462 of our manuscript, which provide evidence for our lncRNAs in other neuroendocrine cancers. More importantly this further substantiates the clinical relevance of our identified lncRNAs.

4. The authors stated that class III, IV, and V are candidates for mechanistic involvement in the transdifferentiation process. It's now established that transdifferentiation from a prostate epithlium toward NEPC involves cellular reprogramming. This is exemplified by recent reports highlighting the role of protein involved in stemness (eg. Sox2, Sox11) in transdifferentiation toward NEPC. Can the authors find any links between these lists of lnRNA and stemness?

We agree with the reviewer's statements that transdifferentiation of prostate epithelium cells to neuroendocrine cells requires cellular reprogramming and we also believe a high degree of cellular plasticity. To support the involvement of our NEtD lncRNA (Classes III and IV) in this process we extended our TFBS analysis (described above) to specifically focus on TFs known to be involved in treatment induced NEPC and/or stemness. These included specific TFs such as BRN2, REST, and STAT3 and entire families of TFs such as ETS, glucocorticoid response elements (GREF) (i.e. AR binding sites), HOX, SOX, and pluripotency/stem cell factors (STEM). Please see manuscript lines 411-426, method section lines 770-801, and supplementary table 22).

We would also like to note we completely removed all analysis and associated reference to NEtD Class V – Reactivated transcripts from our manuscript. We did this for the following reasons:
A. Aside from significant differential expression in our clinical cohorts, we identified no significant results in downstream analysis from these transcripts
B. The significant differential expression results we did obtain (n=19) were the fewest among all other NEtD Classes suggesting that we did not have appropriate clinical cohorts to identify transcripts with this expression pattern and/or their biologically they were not relevant to the NEtD process
C. Most of these transcripts (aside from 2) were from BAC clone regions which are mostly uncharacterized/invalidated transcripts and therefore contain a higher frequency of false positives
D. To decrease study complexity and increase readability of our manuscript, especially in the context of the above reasons

5. In page 6, the authors stated that « These lncRNAs would be candidates for the mechanism driving the deregulation of known protein coding genes (PEG10, N-Myc, SOX2, SRRM4, REST, etc…) in NEPC/NEtD ». The authors can't exclude the possibility that these regulators, as transcription or splicing factors, might be responsible in establishing these lnRNA expression signatures.

We thank the reviewer for pointing this out and agree with this statement. Therefore, we have

revised the manuscript accordingly (please see manuscript lines 182-183).

6. The authors should make their figures friendlier to the reader (eg. enhancing their legends and adding the cohort's name in the Hierchachical clustering).

We have modified figures 1, 2, and 3 to include additional labels or increase their legends. To further enhance the readability of our figures we have split figure 1 into two figures and figure 2 into two figures. See figures 1-4 for these changes.

Reviewer #2:

The authors defined a landscape of lncRNAs in in Neuroendocrine Prostate Cancer via analyzing a large collection of RNA-Seq data from NEPC progression PDX models and patient cohorts. The dataset and the results are valuable for further research investigating the role of lncRNAs in NEPC. However, I have several major concerns.

Most of the reviewer's points we agree with and most importantly strengthens our work (as outlined below in our response). For the one we disagree with (comment #2), we have explained our reasoning in detail. For all others, we have carried out the requested revisions, explained each one in detail below, and referenced manuscript amended lines. We have also referenced new figures/tables necessary for these revisions. We thank the reviewer for the time taken in assessing our research, comprehensive review (especial with respect to their requested minor revisions), and their constructive feedback.

Major comments:
1. A major weakness of this manuscript is the lack of functional/mechanistic validation. Given the less stringent approach prone to high false positive rate (see below), many of the proposed lncRNAs might be artifacts of the analysis. Hence, validation of the results is important to demonstrate the usefulness of the analysis and the proposed catalog of NEPC lncRNAs. Furthermore, the authors should interpret/discuss more about the relevance of cancer-related lncRNAs discovered in each analysis.

While we admit that our manuscript lacks functional/mechanistic validation, we feel this is outside the scope of this study. As outlined in the last paragraph of our introduction, we sought to characterize the NEtD/NEPC lncRNA landscape and identify any clinicopathological associations related to NEPC. Neither of these questions have been answered comprehensively to date. This research was performed in silico, amalgamating a large number of experimentally derived resources from our previous studies and collaborators. Preliminary experimental functionalization of our candidate NEtD/NEPC lncRNA in vitro is currently underway.

That being said, we have added two parts to our manuscript that we hope address the reviewer's request here for validation and functional exploration.

The first is qRT-PCR, which we have outlined in response to Reviewer #1's comment #1 (see above). In brief, we have validated expression (or lack thereof), for 8 of our NEtD lncRNA (2 within each class) and 3 of our NEPC lncRNA by qRT-PCR (see Supplementary Figure 12 and Supplementary Table 25). References in our manuscript and methods are outlined on lines 478-480 and 696-722, respectively. We validated the 8 NEtD lncRNA within our NEtD PDX xenograft model at the appropriate time points and we validated the 3 NEPC lncRNA within our clinical samples. The selection of lncRNAs was from within our top candidates (see workflow in Figure 1C and methods section line 830-844 for identification description).

Second, also outlined in our response to Reviewer #1's comment #2, #3, and #4, we have added a significant amount of research relating to the identification of functional roles for our NEtD lncRNA. Please see explanation above for comment #2, #3, and #4. More specifically, the identification of STAT3, BRN2, REST, and the ETS, GREF, HOX, SOX, and STEM families of TFBS within our lncRNA provide strong functional evidence for their involvement in NEtD (see the method section lines 770-801, manuscript lines 286-438 and supplementary tables 5-24).

We address the reviewer's comment of false positive rates within our analysis in response to comment #3 below.

Lastly, we agree with the reviewer that we failed to highlight cancer-related lncRNAs discovered in each analysis and discuss their relevance. In fact, a number of our NEtD/NEPC lncRNA are associated with cancer and we neglected to discuss these. We have thoroughly revised our manuscript in each section to address this concern. Please see manuscript lines 197-219, 259-281, 390-400, 418-426, and 560-625.

2. It appeared that isoform level analysis was performed to find DE lncRNAs. Although differential expression can be detected at isoform level, this is particularly difficult for lncRNAs given their low expression and poor annotation. For example, the lncRNA MIAT locus is annotated with over 20 transcripts and most of them only slightly differ in the transcript structure indicating misannotation, a common issue with inadequate and uneven RNA-Seq sequencing coverage. As a result, the lists of signature lncRNAs proposed by the authors here contained significant duplication as multiple isoforms from the same locus are presented. The authors should consider reanalysis and/or removal of such repetition.

We agree with the reviewer's comment that lncRNAs are known to have relatively low expression and poor annotation. We also agree it is a common issue with RNA-sequencing to have inadequate and/or uneven sequencing coverage across transcripts. However, we do not agree that "significant duplication" and "repetition" of multiple isoforms from the same locus exist in our data. Collectively for ease of reading, we will call this misannotation.

In general, our position is that there could exist a subset of lncRNA isoforms that are misannotated and due to this they would be "artificial transcripts". We feel the risk of keeping these artificial transcripts does not outweigh the power in accurate mapping/identification (tophat algorithm) and quantification (cufflinks algorithm) achieved. This would be lost if isoform level sequence analysis was not performed. Combined with this, our study design prevents these artificial transcripts from persisting in downstream analysis.

More specifically, these are our reasons for implementing an isoform level lncRNA sequence analysis pipeline and for refuting the reviewer's suggestion to remove transcripts that are a potential consequence of misannotation:
A. Similar to mRNA/protein coding genes, variants or isoforms for a given lncRNA biologically exist. Well-known cancer lncRNA oncogenes/tumor suppressors such ANRIL2, MEG33, and SOX2OT4 are prominent examples of this. ANRIL, MEG3, and SOX2OT have 17, 27, and 59 isoforms, respectively. In many cases these isoforms are tissue and cell type specific. Similar to protein coding genes, RNA processing via the spliceosome complex carries out alternative splicing for lncRNAs due to many of them being multi-exon transcripts.
B. Similar to mRNA/protein coding genes, alternative isoforms for a given transcript can exist simultaneously in some biological context. A great example of this is SOX2OT, which has two of its isoforms both co-up-regulated with SOX2 and OCT4 in esophageal squamous cell carcinoma5. This

could be occurring with H19 in our data, which has multiple isoforms present in our NEPC lncRNA patient signature.
C. The tuxedo pipeline is designed for transcript detection at the isoform level6 and we specifically chose it for this reason. Performing sequence analysis at the gene level compromises accurate transcript quantification overall7,8. Most sequence analysis algorithms use optimization approaches, meaning a computationally "forced" solution can result. In the case of sequence analysis this means reads could be mapped to transcripts (most parsimonious solution) they do not belong too if isoform or mort impactful, novel transcript level detection is not considered.
D. We used only "annotated"/known transcripts from the Ensembl database, which incorporates the gold standard non-coding RNA reference from the ENCODE project 9.
E. We cannot systematically and comprehensively identify misannotated isoforms (if they are present) from correctly annotated isoforms. Therefore, each transcripts' expression would need to be a summarization (average or summation) of all isoforms for that particular transcript. You can imagine for large transcripts, which is the case for lncRNAs, this would significantly skew that transcript's expression (normalization considers transcript length) relative to another transcript. Since lncRNA are known to have low expression, this change would have a great influence on identifying differentially expressed lncRNAs.
F. A number of levels of downstream analysis after our initial sequence analysis in our study would prevent misannotated isoforms from persisting. i.e. Would not validate by qRT-PCR or would not be expressed in a second cohort (VPC vs. WCM).
G. Identification of the exact isoforms involved in NEtD/NEPC is required for the following downstream analysis in this study:
i. Our analysis identifying clinicopathological associations in our GRID cohorts (MCI and MCII) required isoform level expression. We first needed to map our sequenced lncRNA regions to the microarray probe set regions (PSRs) that these cohorts were profiled on. This remapping of PSRs required isoform level detail for accurate expression estimates
ii. Our analysis predicting lncRNA target interactions required the exact structure and sequence (at the exon level) for determining binding targets. This was necessary because a prerequisite to prediction is first resolving the secondary structure of our lncRNA. This requires isoform level detail. Resorting to gene level analysis (vs. isoform level) would cause increased false positives for these predicted targets, since many of these exons incorporated in the prediction would not be expressed in our samples.

Our sequencing pipeline also includes a method called cuffmerge, which addresses the concern of inadequate and/or uneven sequencing coverage. We acknowledge this does occur very frequently and most research neglect to correct for this. In brief, the cuffmerge algorithm fills in the gaps in sequencing that can occur because of poor quality of reads or inadequate sequencing coverage in some areas. i.e. A sample with low sequencing coverage genome-wide may have inadequate coverage for a lowly expressed transcript, such as a lncRNA. Cuffmerge amalgamates all sample transcriptome assemblies and forms a "merged" cohort-specific annotation. Samples are then re-mapped (cufflinks/cuffquant) to this more "complete" transcriptome annotation. To accomplish this, isoform level detection is a requirement.

Taken together, removing isoform level sequence analysis would significantly compromise our entire study. We intentionally selected this method for increased comprehensive transcriptome characterization. Due to this being the first landscape study profiling lncRNA in NEtD/NEPC, we believe this to be critical.

However, we did go back to our annotation of 50,000 lncRNAs and performed a deeper analysis to remove any potential redundant isoforms. To accomplish this we systemically confirmed each isoform for their genomic location and length (based on their ensemble transcript IDs – ENST#).

What we noticed was approximately 13K lncRNA were outside the annotation build GRCh37.v75. One of the advantages of the tuxedo pipeline is the identification of novel transcripts and/or isoforms. Likely these transcripts were within this novel category however, each would need to be PCR validated. Since novel transcript discovery is outside the scope of this study we removed all of these transcripts. We should note this modification (potential novel transcript discovery) is different from the concern the reviewer is raising here (isoform level vs. gene level analysis).

We can now confidently say that all transcripts referenced in our data are correctly annotated and present within hg19 (Ensembl GRCh37 – gtf version 75).

3. Using a p-value < 0.05 as a criterion for DE analysis could potentially introduce many false positives, given a huge number of genes being tested. Have the authors considered using FDR to control for false positive rate? The fact that "only 9.3% (n=330) were commonly deregulated in both CPRC subtypes" could be an indicator that many of the discovered lncRNAs were indeed false positives. While it might not be possible to stringently control for FDR, the issue with false positive should be carefully evaluated and minimized.

We completely agree a p-value < 0.05 as a criterion for DE analysis could introduce many false positives. However, for the following two reasons we intentionally did not apply a FDR correction and feel we have minimized the FDR:

A. Small sample sizes: The first and most important reason we did not apply FDRs in our DE cohort analysis was because of our CRPC/NEPC small sample sizes. Specifically in our VPC cohort we analyzed five AR+ CRPC and five AR- CRPC (NEPC) clinical samples. In our WCM cohort there were seven NEPC clinical samples. NEPC is a very rare disease and obtaining biopsies has been a challenge since most patient's present disease at the time of death and autopsy. For these reasons identifying statistically significant changes in such a small group of patients (without FDR correction) is already a challenge. We knew this would present a problem statistically for our project and so our study (see "B") was designed strategically to minimize the FDR.

B. Study design: To correct and minimize FDR we have carefully designed a workflow (see Figure 1B) that has prevented the persistence of candidates that we discovered by chance. For example, all NEtD lncRNA initially discovered from our NEtD PDX xenograft model were cross-validated (and integrated) with our internal clinical samples (VPC and WCM cohorts) and required each to have significant expression alterations (p-value<0.05). This was also done to remove any model-bias and/or mouse sequenced read contamination. Following this, top candidates were assessed by clinicopathological analysis in our external clinical samples (MCI and MCII) where again significant p-values were required. In fact, it should be noted that VPC/WCM were sequenced whereas the MCI/MCII cohorts were profiled using microarrays. Observing consistent results across different technologies, platforms, models, and clinical cohorts further strengthens the robust nature of our final candidates. Similar logic to our workflow for the discovery of our NEPC lncRNA patient signature (VPC+WCM -> JHMI -> MCI -> MCII).

We did redo all of our DE analysis (specifically in our patient cohorts) and as per the reviewer's request we have applied FDR correction to our p-values. Specifically, we have performed multiple test corrections (Bonferroni and Hochber) to all of our DE analysis (see Supplementary Table 3). Based on these results, as expected we do lose quite a large amount of NEtD lncRNA (1331/2147) after FDR correction. We believe these lost NEtD lncRNA are still biological relevant to our results and have clinical significance. Especially since 19 of 25 (predictive of rapid metastasis) and both SSTR5-AS1 and LINC00514 (probability of poor outcome) are lost after FDR correction and were statistically significant in downstream analysis in larger cohorts (MCI and MCII) for predicting rapid metastasis and poor survival outcome, respectively.

If the reviewer and editor insist prior to publication we will include the FDR analysis into our publication. However, we would request to include this as an isolated aspect of our research and a supplementary table. Furthermore, we would request this not be incorporated into our workflow (Figure 1B) since FDR has been considered downstream. However, for the reasons elaborated above, we feel applying a statistical FDR correction is not appropriate at the discovery stage of this work, considering our study design and small samples sizes.

4. It is fair to say that the set of 216 lncRNAs identified by the authors has some potential for classifying NEPC from AD. However, the size of the validation cohort was small. It would be very interesting to see whether this signature holds in additional validation datasets. The authors should also consider showing all the heatmaps together in one figure, from discovery to validation cohorts to help readers better relate and interpret the results. Are the pattern of expression within genes consistent across cohort? Furthermore, is this possible to construct a classification model using the lncRNAs' expression (eg. using a linear model)? Do any lncRNAs in the signature have known biological functions in cancers and/or prostate cancer?

We agree with the reviewer that it would be interesting to validate our signature in additional datasets. However, we had a number of criteria when we selected our cohorts for the NEPC signature analysis, combined with the fact that there are few publicly available NEPC cohorts. That said, the biggest factor was mapability of our lncRNA of interests. The JHMI validation cohort was selected because it was an independent/external cohort, profiled with a different technology than our discovery cohorts (sequencing vs. microarray), and had a chip (Affymetrix Human Exon 1.0 ST GeneChips) that allowed for remapping to our NEPC lncRNA sequenced reads. All other NEPC cohorts considered were either not publicly (or through collaborators) available, not profiled by a technology that allowed for mapping of our NEPC lncRNA reads, or both reasons. Again it should be noted that the clear separation of NEPC and AD in the JHMI cohort for our NEPC lncRNA (Figure 2), supports the strong robust nature of our signature. Considering the different technologies, platforms, institutes, samples collection, methods for RNA extraction, profiling library preparation, and clinical cohort composition.

While we didn't have appropriate cohorts to validate our NEPC lncRNA signature verbatim (for reasons above), we did validate components of our signature. We selected lncRNA that we expect to be active in NEPC and confirmed their expression in a relatively large CRPC cohort (n=114 – 44 NEPC). From this analysis we saw that 34% of this cohort had at least one of our NEPC lncRNA up-regulated (see Supplementary Figure 7-11). We describe these results in a new section entitled "NEPC and NEtD lncRNAs identify putative molecular NEPC subtypes" lines 440-462 of our manuscript, which provide evidence for our signature being present in other neuroendocrine cancers. More importantly this further substantiates the clinical relevance of our identified lncRNAs. We hope this satisfies the reviewer's request for validation. In our opinion, given the resources available, it is the best that can be done at this time.

We have put all three clustering figures (discovery cohorts and validation cohort) within one figure to help the reader interpret our results, as requested by the reviewer (please see Figure 2).

The reviewer inquired if patterns of mRNA/protein coding gene expression across our cohorts are similar? Please see Supplementary Figure 3-4 (mRNA plots) for this result. We observe that the expression of protein coding genes in our VPC and WCM cohorts (similar to our three lncRNA subclasses) exhibit a similar pattern and furthermore ability to segregate NEPC and AD samples. We observe these similar patterns by two methods of comparison: unsupervised hierarchical clustering and principle component analysis.

The reviewer has also asked about the possibility to construct a classification model using these lncRNAs (i.e. linear regression). We would like to remind the reviewer that the primary purpose of identifying a NEPC lncRNA signature was to support our hypothesis that lncRNA transcripts are involved in NEPC at a molecular and cellular level. We feel constructing a statistical model for our lncRNA expression is outside the scope of our study. More importantly, our cohorts are not appropriate for this due to their heterogeneity (JHMI) and small samples sizes (VPC and WCM).

Lastly, the reviewer has inquired if any of our NEPC lncRNA have biological functions previously linked to prostate or other cancers. Since our signature was designed in an unbiased manner (with respect to lncRNA selection), we did not explore each of the lncRNA independently. We thank the reviewer for bringing this to our attention because 97 of our top 100 have been studied to some extent previously, with 53/97 identified in cancers, and 16/53 in prostate cancer specifically. We have added this depth to our results and discussed in manuscript lines 197-219 (as mentioned above with the request to discuss cancer lncRNAs)

5. Please revise the title of the section "Neuroendocrine prostate cancer long non-coding RNAs are involved in treatment-associated metastasis". Although the authors observed some correlation between SSTR5-AS1 and LINC00514 and metastasis, there were no validation data to support that these lncRNAs are driver of metastasis. Furthermore, the number of samples in KM analysis for MCII was small (< 40). I wonder how the KM curves for those two lncRNAs look in the other cohorts (eg. MCI)?

We completely agree with the reviewer that we do not have data supporting that these lncRNAs are drivers of metastasis. We apologize for misleading the reader as this was not what we were intending to prove. The goal of this component of our study was to investigate the predictive/prognostic abilities of our identified lncRNAs and more generally to determine if any clinicopathological associations were present. We have revised the result section title to reflect this (see manuscript lines 464-523).

At the request of the reviewer, we have generated KM curves for our two lncRNAs in another cohort (MCI). See below (Panels A-B represent non-ADT samples and Panels C-D represent ADT samples). In the context of ADT, we see a significant separation of probability of poor survival (as defined by the metastatic end point) for tumors with low vs. high (compared to the cohort median) SSTR5-AS1 expression (Panel C). In the context of no ADT, we do not see a significant separation (Panel A). For LINC00514, we do not see a significant separation in either ADT treated or non-ADT treated, for outcome (Panel B and D). However, in the context of ADT (Panel D) there is a trend toward separation, although statistically insignificant. While this is an interesting result we cannot include this data in our publication. By definition in the original study, MCI and MCII are observational studies. MCI by design, is a “case-control study” and served as the discovery arm for MCII, which was a “case cohort” and served as the validation cohort10,11. Case-control studies have artificial insertion of test samples (in this case metastatic samples) for the purposes of signature training. For this reason, statistically it would be incorrect to test probability of survival and generate KM plots in MCI. This was the primary reason why all of our survival analyses were performed on MCII, which is a statistically valid cohort for this purpose and with respect to observational studies. We include the KM plots here since they were requested and serve for interest purposes only. They neither support nor contradict our results statistically.

6. I found it quite difficult and confusing to understand what comparisons were performed and how many lncRNAs were obtained for each comparison, how many are shared between comparisons. This could be vastly improved by: (i) clarifying the workflow in Fig. 1C by using more descriptive wordings (eg. what are PDX lncRNA, PDX+WCM lncRNAs, XXX lncRNA, "top-ranked"?) and describing

what happens between the boxes (filter, combination, intersection, overlap, etc?), and (ii) including the Venn diagrams (some are found in the supplementary figures) showing the overlapping lncRNAs found between comparisons. Fig 1A,B,C and all descriptive results and number of lncRNAs found should be presented in a single figure. Similarly, this applies to Fig 2B,C. Eg., Fig. 2B,C should include the number of lncRNAs discovered for each comparison and intersection.

We completely understand why the reviewer found that section of our manuscript confusing. We have improved this section by: (1) splitting figures 1 and 2 into two additional figures – allowing for more descriptive results to be added, (2) splitting this result section into two result sections based on the analysis performed, (3) adding a new table to outline all of our cohort comparisons, group sizes, fold changes, and p-values, and (4) improving our workflow figure to add more details of what action occurred at every step of our study. Please see figures 1-4 for these new illustrations.

7. The text and legends in many figures are too small and difficult to read.

This was a comment made by Reviewer #1 as well. We apologize for making our data poorly visually assessable. As mentioned above to Reviewer #1, we have modified our figures to include additional labels or increased their legends. To further enhance the readability of our figures we have subdivided figures 1 and 2. See figures 1-4 for these changes. Thank you for raising this concern.

Minor comments:
1. It is not clear whether the overlapping lncRNAs in the Venn diagrams were DE in the same direction (ie. were lncRNAs upregulated in one dataset but downregulated in the other considered among the overlapping lncRNAs?)

No, if directionality of regulation were contradictory between datasets then these lncRNAs were not overlapping with one another.

2. Please interpret the results in this sentence "Prominent examples observed in our NEtD model (Figure 2D), WCM cohort (Figure 2E), and VPC cohort (Figure 2F) illustrate each of these NEtD defining transcript classes". What are the lncRNAs and how are they clinically/biologically relevant?

Elaboration of this statement has been made. These lncRNAs are clinically relevant due to their elevated expression levels in our clinical cohorts. Further discussion of these examples has been made in manuscript lines 259-281.

3. Page 6, line 4: Please clarify "three classes were capable of distinctly distinguishing NEPC and AD clinical samples". What does it mean? What were the criteria? What/how many lncRNAs were included in the classification? How were they chosen? Is there any biological insight underlying this or is this a technical artifact?

We have further elaborated on this statement, including the criteria used, number of lncRNAs included, method of selection, and insight into the biology of these classes. Please see manuscript lines 148-155.

4. Fig. 1A,B. Since Fig. 1 is already a color figure, it is better to present the Venn diagram using more distinguishable colors. Moreover, the space in the Venn diagram is large enough to place the transcript classes without using too many color legends.

We have split this figure (as mentioned above) into two, one for observational results and one for our NEPC lncRNA signature discovery and validation. Since we have removed the hierarchical

clustering colour plots), we have decided to kept the venn as black and grey tones, combined with brown tones to highlight the lncRNA classes investigated more deeply in this study. To further enhance readability, we have increased the legends, size of figure overall, and unified the grey and brown tones so that each transcript type has a unique colour.

5. Fig. 2A. What does the y axis present? Fig. 2D. One bar for FENDRR is missing. Fig. 2E. Last two plots are missing.

The y-axis in 2A represents tumour volume (red line) and PSA (blue line) as denoted in the legend. As mentioned previously, we have split figure 2 into two figures, allowing us to enhance this legend and increase readability.

In figure 2D the last bar for FENDRR is intentionally missing. The last bar (black) of each triplet/lncRNA represents the fold change from the AD initial time point to the NEPC terminal point, as outlined in the figure legend. For this particular transcript (transiently expressed) there is no difference in expression between these time points, therefore there is no bar. Instead the expression is up-regulated from the AD to pre-NEPC time point (white with black outline bar) and then down-regulated from the pre-NEPC to NEPC time point (gray bar). Figure D-F will be a figure on its own so that we can increase the legend for these expression plots.

In figure 2E, the last two plots are intentionally missing. This is because for the WCM cohort we did not have NHT clinical patients and therefore could not clinically validated transient and reactivated transcripts in this particular cohort. We will revise this figure with large “X” marks in these spaces and also explain the missing data in the figure legend to avoid this confusion.

6. Fig 3B,C: Please include the number of patients at risk at each break of time, clarify the legend (0 and 1 is not descriptive), and change the p-value to decimal to match with text in results section.

All KM plots have been recalculated and plotted to now include patients at risk, more descriptive legends, and reformatting of the p-value.

7. Page 4, line 24: Instead of putting 'cured' in quote, the authors should rephrase to clarify.

Manuscript lines 60 has been modified to clarify the statement ‘cured’

8. Page 4, line 57: The author should consider using "nucleotides" in lieu of bp as lncRNA is single stranded.

All references to bp for our lncRNAs have been changed to nucleotides or nt for short, as requested.

9. Page 8, line 23: Typo in "preform"

This typo has been corrected.

10. Page 9, line 23: Please consider changing "TADA3s deregulation" to "deregulation of TADA3" to preserve gene name.

Gene names have been modified throughout the text to preserve correction transcript nomenclature

11. It is safe to use "lncRNAs" in plural form.

Thank you for notifying us with this error. The plural form for lncRNA has been revised throughout the manuscript

REFERENCES
1 Beltran, H. et al. Divergent clonal evolution of castration-resistant neuroendocrine prostate cancer. Nature medicine 22, 298-305, doi:10.1038/nm.4045 (2016).
2 Sarkar, D. et al. Multiple Isoforms of ANRIL in Melanoma Cells: Structural Complexity Suggests Variations in Processing. Int J Mol Sci 18, doi:E1378 (2017).
3 Zhang, X. et al. Maternally expressed gene 3 (MEG3) noncoding ribonucleic acid: isoform structure, expression, and functions. Endocrinology 151, 939-947, doi:10.1210/en.2009-0657 (2010).
4 Saghaeian Jazi, M., Samaei, N. M., Ghanei, M., Shadmehr, M. B. & Mowla, S. J. Identification of new SOX2OT transcript variants highly expressed in human cancer cell lines and down regulated in stem cell differentiation. Mol Biol Rep 43, 65-72, doi:10.1007 (2016).
5 Shahryari, A. et al. Two novel splice variants of SOX2OT, SOX2OT-S1, and SOX2OT-S2 are coupregulated with SOX2 and OCT4 in esophageal squamous cell carcinoma. Stem Cells 32, 126-134, doi:10.1002/stem.1542 (2014).
6 Trapnell, C. et al. Differential gene and transcript expression analysis of RNA-seq experiments with TopHat and Cufflinks. Nature protocols 7, 562-578, doi:10.1038/nprot.2012.016 (2012).
7 Trapnell, C. et al. Transcript assembly and quantification by RNA-Seq reveals unannotated transcripts and isoform switching during cell differentiation. Nat Biotechnol 28, 511-515, doi:10.1038/nbt.1621 (2010).
8 Garber, M., Grabherr, M. G., Guttman, M. & Trapnell, C. Computational methods for transcriptome annotation and quantification using RNA-seq. Nat Methods 8, 469-477, doi:10.1038/nmeth.1613 (2011).
9 Consortium, E. P. An integrated encyclopedia of DNA elements in the human genome. Nature 489, 57-74, doi:10.1038/nature11247 (2012).
10 Erho, N. et al. Discovery and validation of a prostate cancer genomic classifier that predicts early metastasis following radical prostatectomy. PloS one 8, e66855, doi:10.1371/journal.pone.0066855 (2013).
11 Karnes, R. J. et al. Validation of a genomic classifier that predicts metastasis following radical prostatectomy in an at risk patient population. The Journal of urology 190, 2047-2053, doi:10.1016/j.juro.2013.06.017 (2013).

Close
