# Supplementary material for: The long noncoding RNA landscape of neuroendocrine prostate cancer and its clinical implications
Source: Gigascience. 2018 May 10;7(6):giy050. doi: 10.1093/gigascience/giy050 (PMC6007253; doi:10.1093/gigascience/giy050)
Supplement: Response_to_Reviewer_Comments_Revision_1.pdf [file giy050_response_to_reviewer_comments_revision_1.pdf]

Author's Response To Reviewer Comments

Close

Please see attached letter for formatted version of response letter below.

Response to reviewers: our responses are indicated by BOLD text below each of the reviewers' comments.

Reviewer #1:

The authors have carried out all my requested revisions. The added data strengthen the manuscript and the new figures make it clearer for the reader.

Thank you for going through our manuscript once more and for the previous feedback. The new data added could not have been accomplished without your review.

Reviewer #2:

We would like to thank the reviewer for their time going over our work once more. The ongoing feedback continues to improve our manuscript. Please see below for our reply to each of your comments below.

I thank the authors for the enormous amount of work to address the concerns raised by me and another reviewer. The manuscript has been significantly strengthened. However, I still have several comments.

1. The authors have acknowledged that the small sample size has significantly affected the statistical power to detect differentially expressed genes. However, small sample size should not be used as a reason for not performing rigid statistical analysis. The authors could try various pre-filtering approaches to improve FDR estimate (or not). However, because the authors made several discovery using the whole list of DE lncRNAs, they should choose a reasonable FDR cutoff for nominating DE lncRNAs, and provide appropriate discussion regarding the chosen FDR cutoff to ensure transparency and assist any follow-up studies.

We completely agree with the authors comment that small size should not be used as a reason for not performing rigid statistical analysis. We apologize for the confusion in our previous response if that message was relayed. With this in mind, as mentioned in our first response-to-reviewer letter, we have reanalyzed and applied FDR corrections to all patient group-wise comparisons for identifying differential expression in our NEPC lncRNAs (DE lncRNAs). More specifically, we ran a Student t-test between all of our clinical groups within each cohort (Supplementary Table 3 – Columns B-E) and to minimize FDR we performed a Bonferroni and Hochberg multiple test correction to the resulting p-values. Statistically significant lncRNAs were identified with t-test p-values < 0.05 (Supplementary Table 3 – Columns H) and with FDR p-values < 0.05 (Supplementary Table 3 – Columns I). This was at the request of the reviewer.

However, we would like to mention this was only one of two approaches for minimizing FDR in the identification of NEPC/NEtD lncRNAs in our study
1. As an isolated piece of analysis (as described above) we have FDR corrected all p-values from our DE lncRNA analysis for our clinical cohorts. This is to statistically minimize all potential false positives

in this analysis.
2. With studies containing small sample sizes, FDR correction can result in the loss of true positives and so we applied a “filter-down” approach as an alternative to minimizing FDR biologically (See Figure 1C – Model Driven Discovery and Figure 3A-C). In brief, this begins with the use of our NEPC xenograft model and then moving through our four clinical cohorts (NEPC model -> VPC+WCM cohorts -> MCI cohort -> MCII cohort). At each step the appropriate statistical test was applied. This work-flow should prevent any false positives that could occur and minimize the FDR, had we solely relied on non-FDR corrected lncRNAs identified from our DE analysis (t-test p-value < 0.05 on VPC+WCM cohorts).

To further address the reviewers concern here, we modified our methods section to clarify this analysis (See statistical analysis in Methods, lines 816-821). This outlines when FDR correction was applied, our study work-flow, and references to FDR corrected DE NEPC lncRNAs.

Without compromising our study design, we hope this addresses the reviewers FDR concerns and assures them that careful thought has been made to reduce this effect to the best of our abilities and resources.

2. Fig. 2. Are the genes in the heatmap shown using the same order? Based on the fact that they are clustered independently in each heatmap, I suspect they are not in the same order, and therefore may mislead readers when they want to compare the heatmaps side by side. I recommend clustering the genes using the discovery dataset, and use the same gene order to generate the heatmaps for validation datasets. If the lncRNA signature is valid, we should see similar pattern between cohorts.

The lncRNAs in the heatmaps of Figure 2 are not ordered the same and this is because of our discovery and validation method. To identify the NEPC lncRNAs signature, we first performed unsupervised hierarchical clustering (UHC) on the lncRNAs with the top 5% of standard deviations of the expression within the VPC and WCM (discovery) cohorts, independently. This produced 265 and 490 lncRNAs for VPC and WCM, respectively. To minimize potential outliers and redundancy in the data we removed all uncharacterized lncRNAs and took the intersection of these two lists, respectively. This produced our 122 NEPC lncRNA signature. We then tested this signature on an independent cohort, profiled on a different platform, and from a different research center to illustrate and validate it robustness. It is very important to note that with the UHC method, patients (columns) and lncRNAs (rows) of the resulting heatmap are ordered and grouped (clustered) based on their dendrograms (appearing on left and top of the heatmaps). The construction of the dendrograms is the core of our signatures discovery. Dendrograms are determined by your calculation of ‘distance’ (relatedness between items) and ‘linkage’ (how to group related items together). This results in the separation (or not) of patients/lncRNAs and removing this would not allow us to unbiasedly determine how our signature is preforming (is there separation of AD and NEPC phenotypes or not). Setting the order of the lncRNAs would mean removing the dendrograms, biasedly grouping items, and therefor removing our method of discovery, testing and validation. We would also like to note that our signature is not just expression values (heatmap) for our lncRNAs but instead is the mathematical combination of each of this lncRNAs (defined by the dendrograms) and represented in the UHC plots.

That being said, since we can’t remove the clustering component (order of rows/lncRNAs) of our signature, we instead reconstructed the UHC plots with rows (lncRNAs) labeled so that the reader can identify the similar blocks/patterns of expression across all 3 cohorts. To aid in readability with our figure and each of the specific 122-lncRNA names, we extracted these labels, retained the order from the generated plots, and created a new table (see supplementary table 3). Also see manuscript lines 890-891 for this reference.

Without compromising our methodology again, we hope this alternative has resolved the reviewers concern and gives the reader the ability to compare the results in Figure 2.

3. In Fig. S7-S11, the legend is inadequate to interpret the data and potentially creates confusion in the results section. Please provide the full legend for the figures. Eg. are all lncRNAs in a class/group shown or only a subset? What does "**" indicate? The authors should also refer to Fig. S6 in the legend text or create separate oncoPrint legend.

We apologize for the lack of clarity and description presented in these figures and associated legends. For supplementary figures 6-11, we have expanded our figure legends to include:
1. Reference to the oncoprint plot legend shown in supplementary figure 6 to be used for supplementary figures 7-11
2. Description of the number of class NEPC/NEtD lncRNAs included in each oncoprint plot
3. Description of ** NEPC/NEtD lncRNAs (relabeled with 1 in superscript)
We also added a table within figures 7-11 to list:
1. The total number of class NEPC/NEtD lncRNAs identified in study
2. The total number of class NEPC/NEtD lncRNAs detectable/testable in extNEPC cohort
3. The list of class NEPC/NEtD lncRNAs that are detectable AND altered/unaltered in extNEPC cohort
These changes should provide clarity and inclusivity to the validation in the extNEPC cohort. Please see manuscript lines 970-991 and supplementary Figure 6-11 for these modifications.

4. In "From the 58 NEPC and 243 NEtD lncRNAs represented in the extNEPC exome sequencing profiling, 43% (25/58) and 27% (66/243) showed altered expression in up to 34% of NEPC patients,", the authors should show the range rather than specifying "up to 34%". Is this erichment significant?

We have modified this result resection to include the specific range of alterations observed in supplementary figures 7-11. Please see manuscript line 446-447 for this changes. We cannot comment if the range of alterations present in the extNEPC for our NEPC/NEtD lncRNAs is a significant enrichment (much like you could for gene set expression in pathway analysis), since there isn't an appropriate test for this. However, we can calculate the significance of our identified NEPC/NEtD lncRNA overlapping in the extNEPC cohort, with an overlapping lncRNA defined as having at least 1 patients with an alteration present in the extNEPC cohort. With this statistical calculation we can assign significance to the NEPC lncRNAs and NEtD Class I-IV lncRNAs validating in the extNEPC cohort. Please see manuscript lines 445-447 for the changes to this result section and figure legend, respectively. We would also like to note that the direction of expression (up-regulation) for the lncRNAs tested and shown as altered in the extNEPC are the same in our study, further corroborating the lncRNAs we have identified.

5. In "the lncRNAs resulted in identifying three distinct subsets of NEPC patients within the extNEPC cohort.", the authors should clarify what "the lncRNAs" are (all lncRNAs identified or a subset?). More importantly, this claim need to be supported by statistical analysis, and without validation, the authors should also revise the title of the section "NEPC and NEtD lncRNAs identify putative molecular NEPC subtypes" to indicate this is a correlation study in a single cohort.

We have further elaborated on our identification of the putative molecular subtype by our lncRNAs and specify the subset of NEPC and NEtD lncRNAs that define these 3 groups. Please see manuscript lines 449-451 for this changes. With respect to supporting this claim by statistical analysis, we agree having statistical analysis would further support our claim. However, it is important to note NEPC is a very rare disease and sample collection is challenging – especially at various metastatic sites where this disease normally presents. In fact, the extNEPC study is the first of its kind to provide the

spectrum of the disease across different metastatic sites each with different pathological classification. Since our observed lncRNA-based subtype appears to group based on the combination of tumour site, pathological classification, mutational frequency, and ploidy levels, validating this claim would require numerous samples representing different profiles for each of these clinical and genomic features. The magnitude of collecting those samples for a rare disease is not feasible. However, we do take this concern seriously and to make this result more transparent we have modified the result section to emphasis that this is observational result only, the lack of statistical support/feasibility, and renamed the result section accordingly as requested by the reviewer. Furthermore we have clarified this as an observational result that requires corroboration in a larger cohort with a vast range of metastatic sites and NEPC pathologies. Please see manuscript 458-462 for these modifications.

6. The authors should also provide statistical significance for "mutations in TP53 and RB1 did not appear to be associated with one group over another".

TP53 and RB1 genes contained either a copy number aberration and/or mutation in most patients and spread evenly across all 3 groups. We apologize if this statement suggested an anti-correlation to the three subtypes/groups. With no alterations in these genes associated with groups 1-3 we did not test for significance since alterations were spread evenly across a majority of the cohort (see supplementary figure 7). These alterations are known to be a defining characteristic for NEPC patients so we felt it important to note their status and confirmation of their presence globally within the extNEPC cohort. We have clarified this statement, the visual inspection of the oncoprint plots, and this observational result. Please see manuscript lines 456-459.

Close
