# Supplementary material for: The long noncoding RNA landscape of neuroendocrine prostate cancer and its clinical implications
Source: Gigascience. 2018 May 10;7(6):giy050. doi: 10.1093/gigascience/giy050 (PMC6007253; doi:10.1093/gigascience/giy050)
Supplement: Response_to_Reviewer_Comments_Revision_2.pdf [file giy050_response_to_reviewer_comments_revision_2.pdf]

Author's Response To Reviewer Comments

Close

Hello Dr. Edmunds,
All data issues have now been resolved. Please see attached for all final versions of data and our manuscript.
Regards
Rohan

Close
