# Supplementary material for: The long noncoding RNA landscape of neuroendocrine prostate cancer and its clinical implications
Source: Gigascience. 2018 May 10;7(6):giy050. doi: 10.1093/gigascience/giy050 (PMC6007253; doi:10.1093/gigascience/giy050)
Supplement: Reviewer_1_Report_(Original_Submission) -- Etienne Dardenne — 6/19/2017 Reviewed [file giy050_reviewer_1_report_(original_submission).pdf]

Reviewer Report

Title: The Long Noncoding RNA Landscape of Neuroendocrine Prostate Cancer and its Clinical Implications

Version: **Original Submission** **Date:** 6/19/2017

Reviewer name: **Etienne Dardenne**

Reviewer Comments to Author:

Neuroendocrine prostate cancer (NEPC) is an aggressive disease. Since the recent FDA approval of potent AR targeted therapies for the treatment of metastatic prostate cancer, the incidence of AR-negative NEPC will escalate as a mechanism of resistance. Genomic and transcriptomic profiling have been done to characterize NEPC tumors, yet the landscape of lnRNA has never been characterized.
In this article, Ramnarine et al. leverage their well-characterized xenograft model, as well as patient samples, to identify lnRNA deregulated in NEPC. They further define five expression patterns associated with the disease progression from adenocarcinoma to CRPC and NEPC. They further associated some of the lnRNA expression with treatment-associated metastasis.

The manuscript focuses on lnRNA, an aspect unexplored in NEPC. The data presented are interesting but some of the data needs to be strengthened.

1) The authors use a large amount of RNAseq data and implemented a pipeline to analyze the expression of long non-coding RNA. All their evidences described in this paper are based upon in sillico analyses. The authors must validate their finding. Q-RT-PCR experiment should be carried out to validate top candidates in each class defined by the authors in the xenografts model. In addition, as proof of principle, validation in a few clinical samples by RT-Q-PCR or by RNAish in fixed material will further strengthen the manuscript.

2) The authors define five patterns of expression. They named class I "AR regulated" and class II "Neuronal". NEPC is characterized by the loss of AR and AR signaling, and it's expected that AR target genes and lnRNA will go down. However, this nomenclature is misleading. The authors do not prove that lnRNAs falling into class I are indeed regulated by AR. Different mechanisms or pathways might be involved, in addition to AR signaling. Does the list of lnRNA in class I significantly overlap with lnRNA regulated by AR? The authors should leverage existing RNAseq data sets while looking at AR regulated transcripts. Answering these questions will strongly strengthen the data.

3) The same logic applies to class II "Neuronal". The authors do not provide evidence that these lnRNAs are part of any Neuronal pathways or signatures. Is the list of lnRNA in class II enriched in signatures of neuronal tissues and/or of other neuroendocrine cancers (neuroblastoma, Small Cell Lung Cancer)?

4) The authors stated that class III, IV, and V are candidates for mechanistic involvement in the transdifferentiation process. It's now established that transdifferentiation from a prostate epithlium toward NEPC involves cellular reprogramming. This is exemplified by recent reports highlighting the role of protein involved in stemness (eg. Sox2, Sox11) in transdifferentiation toward NEPC. Can the authors find any links between these lists of lnRNA and stemness?

5) In page 6, the authors stated that « These lncRNAs would be candidates for the mechanism driving the deregulation of known protein coding genes (PEG10, N-Myc, SOX2, SRRM4, REST, etc...) in NEPC/NEtD ». The authors can't exclude the possibility that these regulators, as transcription or splicing factors, might be responsible in establishing these lnRNA expression signatures.

6) The authors should make their figures friendlier to the reader (eg. enhancing their legends and adding the cohort's name in the Hierchachical clustering).

Methods

Are the methods appropriate to the aims of the study, are they well described, and are necessary controls included? Yes

Conclusions

Are the conclusions adequately supported by the data shown? Yes

Reporting Standards

Does the manuscript adhere to the journal's guidelines on minimum standards of reporting? Yes

Choose an item.

Statistics

Are you able to assess all statistics in the manuscript, including the appropriateness of statistical tests used? No, and I do not feel adequately qualified to assess the statistics.

Quality of Written English

Please indicate the quality of language in the manuscript: Acceptable

Declaration of Competing Interests

Please complete a declaration of competing interests, considering the following questions:

- Have you in the past five years received reimbursements, fees, funding, or salary from an organisation that may in any way gain or lose financially from the publication of this manuscript, either now or in the future?
- Do you hold any stocks or shares in an organisation that may in any way gain or lose financially from the publication of this manuscript, either now or in the future?
- Do you hold or are you currently applying for any patents relating to the content of the manuscript?
- Have you received reimbursements, fees, funding, or salary from an organization that holds or has applied for patents relating to the content of the manuscript?
- Do you have any other financial competing interests?

- Do you have any non-financial competing interests in relation to this paper?

If you can answer no to all of the above, write 'I declare that I have no competing interests' below. If your reply is yes to any, please give details below.

No competing interests to declare

I agree to the open peer review policy of the journal. I understand that my name will be included on my report to the authors and, if the manuscript is accepted for publication, my named report including any attachments I upload will be posted on the website along with the authors' responses. I agree for my report to be made available under an Open Access Creative Commons CC-BY license (http://creativecommons.org/licenses/by/4.0/). I understand that any comments which I do not wish to be included in my named report can be included as confidential comments to the editors, which will not be published.

I agree to the open peer review policy of the journal

To further support our reviewers, we have joined with Publons, where you can gain additional credit to further highlight your hard work (see: https://publons.com/journal/530/gigascience). On publication of this paper, your review will be automatically added to Publons, you can then choose whether or not to claim your Publons credit. I understand this statement.

Yes Choose an item.
