# Supplementary material for: The long noncoding RNA landscape of neuroendocrine prostate cancer and its clinical implications
Source: Gigascience. 2018 May 10;7(6):giy050. doi: 10.1093/gigascience/giy050 (PMC6007253; doi:10.1093/gigascience/giy050)
Supplement: Reviewer_2_Report_(Original_Submission) -- Ha Dang — 6/30/2017 Reviewed [file giy050_reviewer_2_report_(original_submission).pdf]

Reviewer Report

Title: The Long Noncoding RNA Landscape of Neuroendocrine Prostate Cancer and its Clinical Implications

Version: Original Submission Date: 6/30/2017

Reviewer name: Ha Dang

Reviewer Comments to Author:

The authors defined a landscape of lncRNAs in in Neuroendocrine Prostate Cancer via analyzing a large collection of RNA-Seq data from NEPC progression PDX models and patient cohorts. The dataset and the results are valuable for further research investigating the role of lncRNAs in NEPC. However, I have several major concerns.

(1) A major weakness of this manuscript is the lack of functional/mechanistic validation. Given the less stringent approach prone to high false positive rate (see below), many of the proposed lncRNAs might be artifacts of the analysis. Hence, validation of the results is important to demonstrate the usefulness of the analysis and the proposed catalog of NEPC lncRNAs. Furthermore, the authors should interpret/discuss more about the relevance of cancer-related lncRNAs discovered in each analysis.

(2) It appeared that isoform level analysis was performed to find DE lncRNAs. Although differential expression can be detected at isoform level, this is particularly difficult for lncRNAs given their low expression and poor annotation. For example, the lncRNA MIAT locus is annotated with over 20 transcripts and most of them only slightly differ in the transcript structure indicating misannotation, a common issue with inadequate and uneven RNA-Seq sequencing coverage. As a result, the lists of signature lncRNAs proposed by the authors here contained significant duplication as multiple isoforms from the same locus are presented. The authors should consider reanalysis and/or removal of such repetition.

(3) Using a p-value < 0.05 as a criterion for DE analysis could potentially introduce many false positives, given a huge number of genes being tested. Have the authors considered using FDR to control for false positive rate? The fact that "only 9.3% (n=330) were commonly deregulated in both CPRC subtypes" could be an indicator that many of the discovered lncRNAs were indeed false positives. While it might not be possible to stringently control for FDR, the issue with false positive should be carefully evaluated and minimized.

(4) It is fair to say that the set of 216 lncRNAs identified by the authors has some potential for classifying NEPC from AD. However, the size of the validation cohort was small. It would be very interesting to see whether this signature holds in additional validation datasets. The authors should also consider showing all the heatmaps together in one figure, from discovery to validation cohorts to help readers better relate and interpret the results. Are the pattern of expression within genes consistent across cohort? Furthermore, is this possible to construct a classification model using the lncRNAs' expression (eg. using a linear model)? Do any lncRNAs in the signature have known biological functions in cancers and/or prostate cancer?

(5) Please revise the title of the section "Neuroendocrine prostate cancer long non-coding RNAs are involved in treatment-associated metastasis". Although the authors observed some correlation between SSTR5-AS1 and LINC00514 and metastasis, there were no validation data to support that these lncRNAs are driver of metastasis. Furthermore, the number of samples in KM analysis for MCII was small (< 40). I wonder how the KM curves for those two lncRNAs look in the other cohorts (eg. MCI)?

(6) I found it quite difficult and confusing to understand what comparisons were performed and how many lncRNAs were obtained for each comparison, how many are shared between comparisons. This could be

vastly improved by: (i) clarifying the workflow in Fig. 1C by using more descriptive wordings (eg. what are PDX lncRNA, PDX+WCM lncRNAs, XXX lncRNA, "top-ranked"?) and describing what happens between the boxes (filter, combination, intersection, overlap, etc?), and (ii) including the Venn diagrams (some are found in the supplementary figures) showing the overlapping lncRNAs found between comparisons. Fig 1A,B,C and all descriptive results and number of lncRNAs found should be presented in a single figure. Similarly, this applies to Fig 2B,C. Eg., Fig. 2B,C should include the number of lncRNAs discovered for each comparison and intersection.

(7) The text and legends in many figures are too small and difficult to read.

**** Minor comments:
It is not clear whether the overlapping lncRNAs in the Venn diagrams were DE in the same direction (ie. were lncRNAs upregulated in one dataset but downregulated in the other considered among the overlapping lncRNAs?)

Please interpret the results in this sentence "Prominent examples observed in our NEtD model (Figure 2D), WCM cohort (Figure 2E), and VPC cohort (Figure 2F) illustrate each of these NEtD defining transcript classes". What are the lncRNAs and how are they clinically/biologically relevant?

Page 6, line 4: Please clarify "three classes were capable of distinctly distinguishing NEPC and AD clinical samples". What does it mean? What were the criteria? What/how many lncRNAs were included in the classification? How were they chosen? Is there any biological insight underlying this or is this a technical artifact?

Fig. 1A,B. Since Fig. 1 is already a color figure, it is better to present the Venn diagram using more distinguishable colors. Moreover, the space in the Venn diagram is large enough to place the transcript classes without using too many color legends.

Fig. 2A. What does the y axis present? Fig. 2D. One bar for FENDRR is missing. Fig. 2E. Last two plots are missing.

Fig 3B,C: Please include the number of patients at risk at each break of time, clarify the legend (0 and 1 is not descriptive), and change the p-value to decimal to match with text in results section.

Page 4, line 24: Instead of putting 'cured' in quote, the authors should rephrase to clarify.

Page 4, line 57: The author should consider using "nucleotides" in lieu of bp as lncRNA is single stranded.

Page 8, line 23: Typo in "preform"

Page 9, line 23: Please consider changing "TADA3s deregulation" to "deregulation of TADA3" to preserve gene name.

It is safe to use "lncRNAs" in plural form.

Methods

Are the methods appropriate to the aims of the study, are they well described, and are necessary controls included? No

Conclusions

Are the conclusions adequately supported by the data shown? No

Reporting Standards

Does the manuscript adhere to the journal's guidelines on minimum standards of reporting? No

Choose an item.

Statistics

Are you able to assess all statistics in the manuscript, including the appropriateness of statistical tests used? Yes, and I have assessed the statistics in my report.

Quality of Written English

Please indicate the quality of language in the manuscript: Needs some language corrections before being published

Declaration of Competing Interests

Please complete a declaration of competing interests, considering the following questions:

- Have you in the past five years received reimbursements, fees, funding, or salary from an organisation that may in any way gain or lose financially from the publication of this manuscript, either now or in the future?
- Do you hold any stocks or shares in an organisation that may in any way gain or lose financially from the publication of this manuscript, either now or in the future?
- Do you hold or are you currently applying for any patents relating to the content of the manuscript?
- Have you received reimbursements, fees, funding, or salary from an organization that holds or has applied for patents relating to the content of the manuscript?
- Do you have any other financial competing interests?
- Do you have any non-financial competing interests in relation to this paper?

If you can answer no to all of the above, write 'I declare that I have no competing interests' below. If your reply is yes to any, please give details below.

I declare that I have no competing interests

I agree to the open peer review policy of the journal. I understand that my name will be included on my report to the authors and, if the manuscript is accepted for publication, my named report including any attachments I upload will be posted on the website along with the authors' responses. I agree for my report to be made available under an Open Access Creative Commons CC-BY license

(http://creativecommons.org/licenses/by/4.0/). I understand that any comments which I do not wish to be included in my named report can be included as confidential comments to the editors, which will not be published.

I agree to the open peer review policy of the journal

To further support our reviewers, we have joined with Publons, where you can gain additional credit to further highlight your hard work (see: https://publons.com/journal/530/gigascience). On publication of this paper, your review will be automatically added to Publons, you can then choose whether or not to claim your Publons credit. I understand this statement.

Yes Choose an item.
