# Supplementary material for: The long noncoding RNA landscape of neuroendocrine prostate cancer and its clinical implications
Source: Gigascience. 2018 May 10;7(6):giy050. doi: 10.1093/gigascience/giy050 (PMC6007253; doi:10.1093/gigascience/giy050)
Supplement: Reviewer_2_Report_(Revision_1).pdf [file giy050_reviewer_2_report_(revision_1).pdf]

Reviewer Report

Title: he Long Noncoding RNA Landscape of Neuroendocrine Prostate Cancer and its Clinical Implications

Version: Revision 1 Date: 1/28/2018

Reviewer name: Ha Dang

Reviewer Comments to Author:

I thank the authors for the enormous amount of work to address the concerns raised by me and another reviewer. The manuscript has been significantly strengthened. However, I still have several comments.

The authors have acknowledged that the small sample size has significantly affected the statistical power to detect differentially expressed genes. However, small sample size should not be used as a reason for not performing rigid statistical analysis. The authors could try various pre-filtering approaches to improve FDR estimate (or not). However, because the authors made several discovery using the whole list of DE lncRNAs, they should choose a reasonable FDR cutoff for nominating DE lncRNAs, and provide appropriate discussion regarding the chosen FDR cutoff to ensure transparency and assist any follow-up studies.

Fig. 2. Are the genes in the heatmap shown using the same order? Based on the fact that they are clustered independently in each heatmap, I suspect they are not in the same order, and therefore may mislead readers when they want to compare the heatmaps side by side. I recommend clustering the genes using the discovery dataset, and use the same gene order to generate the heatmaps for validation datasets. If the lncRNA signature is valid, we should see similar pattern between cohorts.

In Fig. S7-S11, the legend is inadequate to interpret the data and potentially creates confusion in the results section. Please provide the full legend for the figures. Eg. are all lncRNAs in a class/group shown or only a subset? What does "**" indicate? The authors should also refer to Fig. S6 in the legend text or create separate oncoPrint legend.

In "From the 58 NEPC and 243 NEtD lncRNAs represented in the extNEPC exome sequencing profiling, 43% (25/58) and 27% (66/243) showed altered expression in up to 34% of NEPC patients,", the authors should show the range rather than specifying "up to 34%". Is this erichment significant?

In "the lncRNAs resulted in identifying three distinct subsets of NEPC patients within the extNEPC cohort.", the authors should clarify what "the lncRNAs" are (all lncRNAs identified or a subset?). More importantly, this claim need to be supported by statistical analysis, and without validation, the authors should also revise the title of the section "NEPC and NEtD lncRNAs identify putative molecular NEPC subtypes" to indicate this is a correlation study in a single cohort.

The authors should also provide statistical significance for "mutations in TP53 and RB1 did not appear to be associated with one group over another".

Methods

Are the methods appropriate to the aims of the study, are they well described, and are necessary controls included? No

Conclusions

Are the conclusions adequately supported by the data shown? No

Reporting Standards

Does the manuscript adhere to the journal's guidelines on minimum standards of reporting? Yes

Choose an item.

Statistics

Are you able to assess all statistics in the manuscript, including the appropriateness of statistical tests used? Yes, and I have assessed the statistics in my report.

Quality of Written English

Please indicate the quality of language in the manuscript: Acceptable

Declaration of Competing Interests

Please complete a declaration of competing interests, considering the following questions:

- Have you in the past five years received reimbursements, fees, funding, or salary from an organisation that may in any way gain or lose financially from the publication of this manuscript, either now or in the future?
- Do you hold any stocks or shares in an organisation that may in any way gain or lose financially from the publication of this manuscript, either now or in the future?
- Do you hold or are you currently applying for any patents relating to the content of the manuscript?
- Have you received reimbursements, fees, funding, or salary from an organization that holds or has applied for patents relating to the content of the manuscript?
- Do you have any other financial competing interests?
- Do you have any non-financial competing interests in relation to this paper?

If you can answer no to all of the above, write 'I declare that I have no competing interests' below. If your reply is yes to any, please give details below.

I declare that I have no competing interests

I agree to the open peer review policy of the journal. I understand that my name will be included on my report to the authors and, if the manuscript is accepted for publication, my named report including any attachments I upload will be posted on the website along with the authors' responses. I agree for my

report to be made available under an Open Access Creative Commons CC-BY license (http://creativecommons.org/licenses/by/4.0/). I understand that any comments which I do not wish to be included in my named report can be included as confidential comments to the editors, which will not be published.

I agree to the open peer review policy of the journal

To further support our reviewers, we have joined with Publons, where you can gain additional credit to further highlight your hard work (see: https://publons.com/journal/530/gigascience). On publication of this paper, your review will be automatically added to Publons, you can then choose whether or not to claim your Publons credit. I understand this statement.

Yes Choose an item.
